# Total Synthesis of the Diterpenes (+)-Randainin D and (+)-Barekoxide via Photoredox-Catalyzed Deoxygenative Allylation

**DOI:** 10.1021/jacs.4c02224

**Published:** 2024-04-15

**Authors:** Oleksandr Vyhivskyi, Olivier Baudoin

**Affiliations:** Department of Chemistry, University of Basel, St. Johanns-Ring 19, CH-4056 Basel, Switzerland

## Abstract

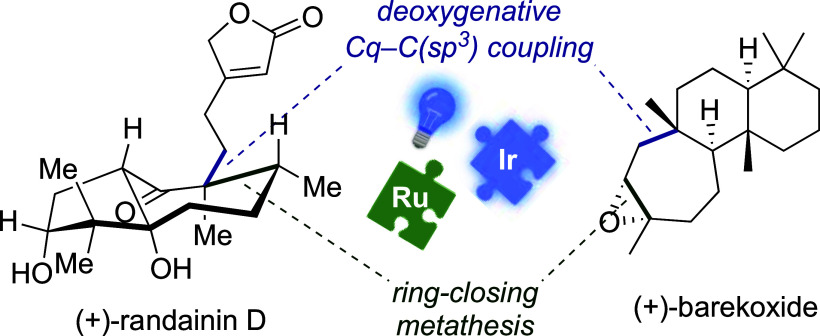

We report the first enantioselective total synthesis of diterpenoid randainin D, which possesses a hydroazulenone core with a β-substituted butenolide moiety on the cycloheptane ring. The *trans*-5/7 ring system was formed via a highly challenging ring-closing metathesis delivering the tetrasubstituted cycloheptenone. The butenolide moiety was installed via a novel deoxygenative allylation under Ir-photoredox catalysis, employing methyl oxalate as a red/ox tag. Moreover, the developed allylation was successfully utilized in the 7-step total synthesis of (+)-barekoxide. This study suggests that this deoxygenative allylation method is a promising strategy for the formation of Cq–C(sp^3^) bonds (Cq = quaternary center) in the context of natural product synthesis.

## Introduction

Terpenoids are the vastest family of natural products, accounting for over 80,000 unique compounds.^1^ The terpenome ubiquity and diversity are accompanied by a wide range of bioactivities, including anticancer, anti-inflammatory, anticoagulative, sedative, and antioxidative effects.^2^ The interesting biological properties of terpenoids, combined with the complexity of their structures, have led to a myriad of total syntheses that both contribute to solving current synthetic issues in organic chemistry and to advance the search for new therapeutic agents.

**Figure 1:**
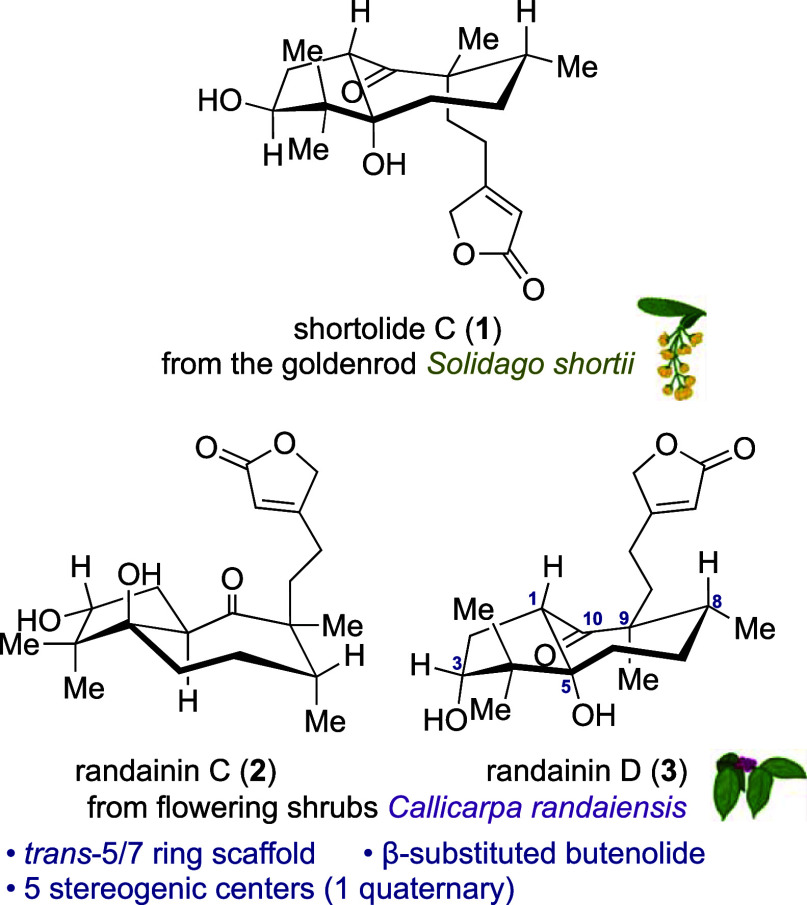
Structures of shortolide C (**1**) and randainins C (**2**) and D (**3**).

Shortolide C (**1**)^3^ and randainins C (**2**) and D (**3**)^4^ (Figure 1) are structurally unique diterpenoids due to the simultaneous presence of the *trans*-hydroazulenone core and the C9-butenolide moiety. Bioassays showed that **1** is a moderate antibacterial agent against *Staphylococcus aureus*, whereas **3** inhibits elastase release and superoxide-anion generation. Neutrophil elastase (NE) is a protease enzyme involved in numerous pathologies including rheumatoid arthritis, cystic fibrosis, and chronic obstructive pulmonary disease.^5^ Recent studies suggested the potential of NE inhibitors in the treatment of COVID-19.^6^ Considering the therapeutic potential of randainin D (**3**), together with its original structure, we aimed to (i) achieve its first total synthesis and (ii) establish a general strategy toward the formation of *trans*-cycloheptane-containing polycyclic terpenes.

**Figure 2:**
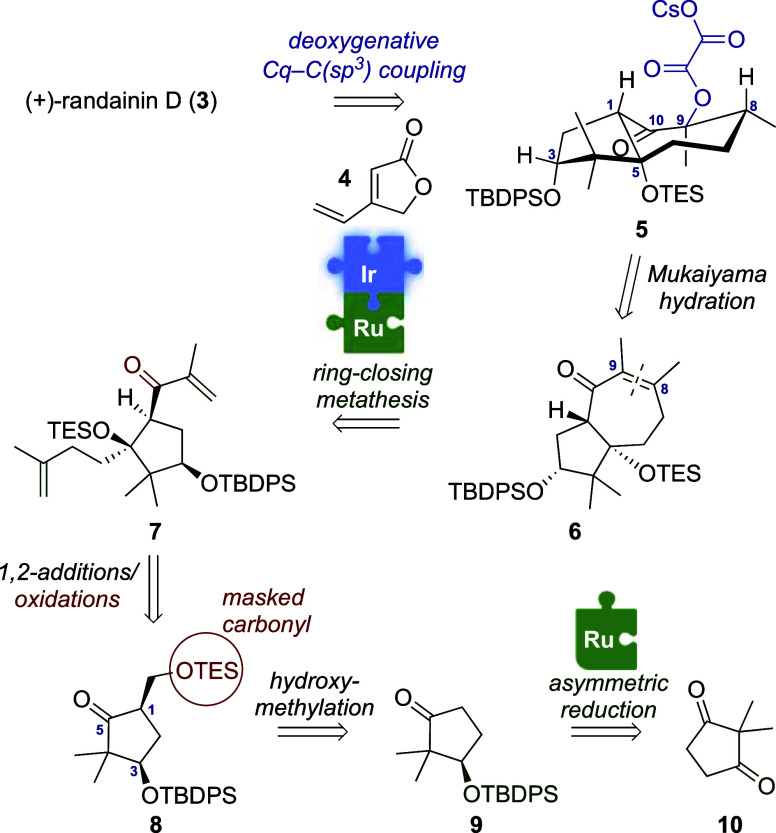
Retrosynthetic analysis of randainin D (**3**).

In the past decade, photoredox catalysis emerged as an indispensable tool for organic chemists.^7^ Mild reaction conditions, high functional group tolerance, and predictable reactivity allow its broad applicability in natural product synthesis.^8^ Deoxygenative strategies for the generation of carbon-centered radicals occupy a special place in this field, with the most commonly employed red/ox tags being xanthates, thiocarbamates, and esters.^9^ In 2015, Overman and MacMillan reported an efficient redox-neutral Giese-type coupling of alkali oxalates with Michael acceptors under blue-light-promoted Ir^III^ catalysis.^10^ This strategy was successfully applied in the highly diastereoselective synthesis of *trans*-clerodane, bearing a β-substituted butenolide moiety, using 4-vinylfuran-2(5*H*)-one (**4**, Figure 2) as the Michael acceptor. Inspired by this work, we report an enantioselective synthesis of (+)-randainin D and (+)-barekoxide, employing the deoxygenative strategy to form the key Cq–C(sp^3^) bonds (Cq = quaternary center) under Ir^III^-photoredox catalysis.

## Results and Discussion

Our retrosynthetic analysis is presented in Figure 2. We envisioned that **3** could be generated by deoxygenative coupling from oxalate **5**. The latter would arise from the α-hydroxyketone obtained via C9-regioselective Mukaiyama hydration of tetrasubstituted enone **6**. Inspired by the successful application of ring-closing metathesis (RCM) for the construction of the *cis*-hydroazulenone core, containing a trisubstituted enone, in the total synthesis of astellatol,^11^ we anticipated that this strategy could be used to form the *trans*-5/7 bicyclic skeleton of **6** from diene **7**. Admittedly, the tetrasubstituted enone in **6** represents a significant challenge compared with previous cases. We planned to access **7** from cyclopentanone **8** via consecutive 1,2-additions and oxidations. In turn, **8** would arise from the diastereoselective hydroxymethylation of **9**, which could be accessed by desymmetrization of commercially available 2,2-dimethylcyclopentane-1,3-dione **10**.

**Scheme 1:**
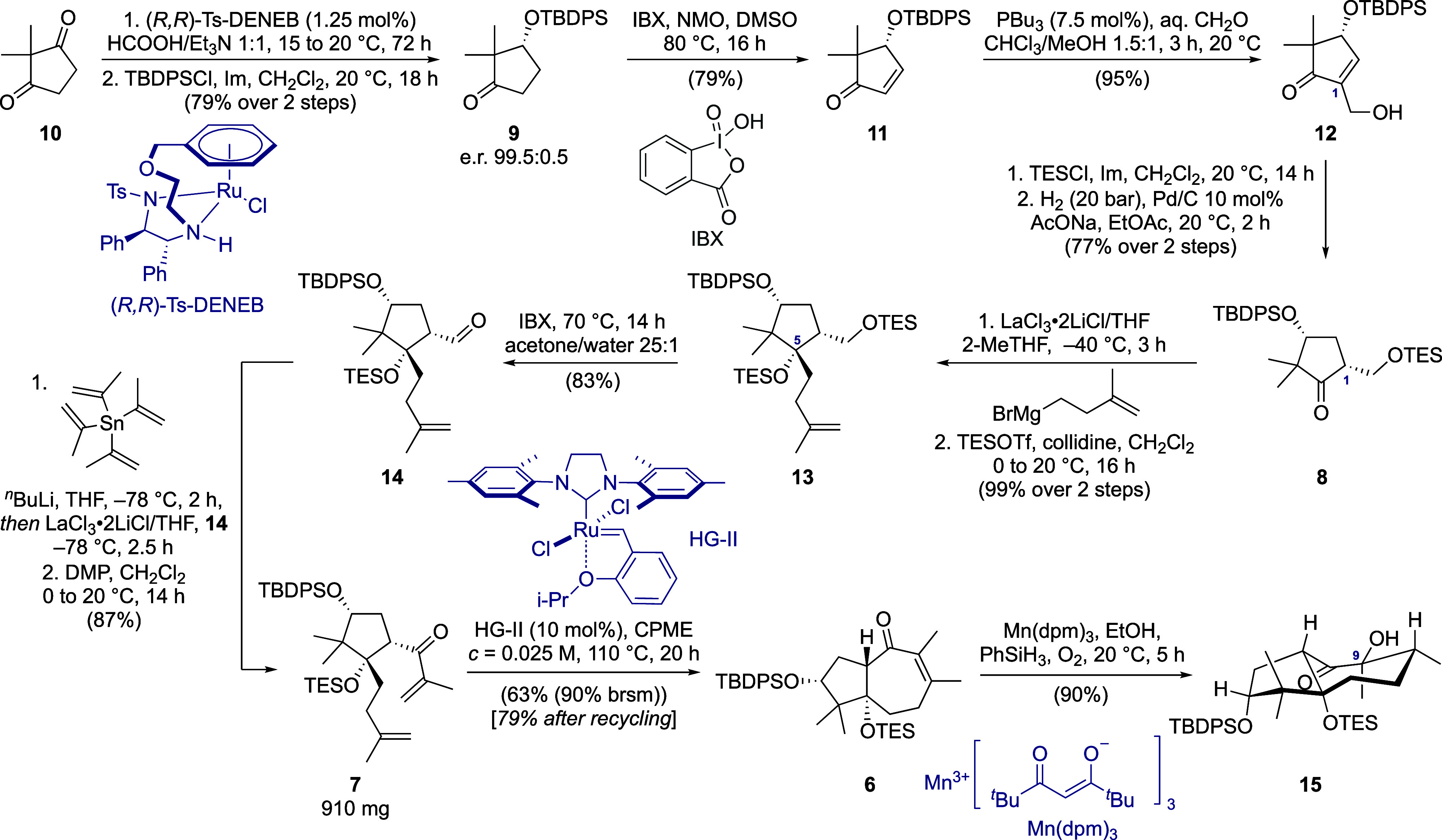
Synthesis of *trans*-Hydroazulenone **6** and α-Hydroxyketone **15**

The enantioenriched diene **7** was synthesized in high yield on the gram scale via the 11-step sequence depicted in Scheme 1. The main reported method for the desymmetrization of cyclopentadione **10** is CBS reduction. Being utilized numerous times in total syntheses,^12^ it provides the desired product in moderate yields (up to 72%) and e.r. up to 98:2. Recently, Metz and co-workers reported the asymmetric transfer hydrogenation of **10** using RuCl(*p*-cymene)[(*S*,*S*)-Ts-DPEN] as the catalyst, furnishing (*S*)-3-hydroxy-2,2-dimethylcyclopentan-1-one in 97% yield and e. r. = 95.5:4.5 on a 0.5 mmol scale.^13^ We hypothesized that the enantioselectivity could be further increased by employing the tethered Noyori–Ikariya catalyst.^14^ Indeed, using commercially available (*R*,*R*)-Ts-DENEB and formic acid as the hydrogen donor, we synthesized the desired TBDPS-protected ketone **9** on a multigram scale in 79% yield over 2 steps with excellent enantioselectivity (e. r. 99.4:0.6). The choice of the silyl protecting group was dictated by the control of diastereoselectivity in the subsequent steps of the synthesis. Our initial efforts to install the C1-side chain via alkylation/isomerization under kinetic control to minimize unnecessary red/ox manipulations were unsuccessful. Instead, the diastereoselective α-hydroxymethylation of ketone **9** to furnish **8** was accomplished through a 4-step sequence. First, **9** was subjected to single-electron transfer (SET)-based dehydrogenation mediated by the IBX/NMO complex,^15^ delivering the enone **11** in 79% yield on a 14 g scale. Subsequent Morita–Baylis–Hillman reaction of **11** with aqueous formaldehyde, catalyzed by tributyl phosphine,^16^ afforded the desired β-hydroxy ketone **12** in 95% yield on a 10 g scale. Introduction of the TES-protecting group, followed by hydrogenation, afforded **8** as a mixture of two C1-diastereomers (d. r. 9.7:1). Sodium acetate, used as a buffer base,^17^ was crucial to prevent the in situ OTES cleavage during the hydrogenation. The obtained diastereomeric mixture was separated via column chromatography, delivering pure (*R,R*)-disubstituted ketone **8** in 77% yield from alcohol **12**. Then, the C5-isopentenyl moiety was successfully installed in nearly quantitative yield upon treatment of the sterically hindered ketone **8** with freshly prepared (3-methylbut-3-en-1-yl)magnesium bromide in the presence of the LaCl_3_·2LiCl complex.^18^ Next, the one-pot deprotection of the primary TES group and oxidation in the presence of an excess of IBX in wet acetone delivered aldehyde **14** in 83% yield. Interestingly, attempts to perform this reaction using intrinsically acidic oxidants resulted in lower yields and caused epimerization of the stereocenter adjacent to the carbonyl group (see Supporting Information). Then, LaCl_3_·2LiCl-assisted 1,2-addition of *i*-C_3_H_5_Li to aldehyde **14**, followed by DMP oxidation, led to the desired diene **7** in 87% yield over 2 steps. Isopropenyllithium was generated via transmetalation from tetra(isopropenyl)stannane upon treatment of the latter with *n*-BuLi in THF at −78 °C.^19^ Attempts to generate the corresponding organolithium species through the *t*-BuLi-mediated lithiation of 2-bromo- or 2-iodoprop-1-ene resulted in poor reproducibility. When the corresponding Grignard reagent was used instead of isopropenyllithium, 1,2-addition proceeded with low yields (∼50%) and led to the formation of side products, presumably due to β-hydride transfer, despite the presence of lanthanide salts.

Having established a gram-scale, high-yielding route (11-step LLS, 32.6% overall yield) to the diene **7**, we moved to the first key step of our total synthesis, i.e., RCM. Despite the significant development in the field of RCM, its application to the synthesis of tetrasubstituted olefins remains a significant challenge.^20^ In fact, to the best of our knowledge, RCM has never been employed in total synthesis to generate a tetrasubstituted cycloheptenone.^21,22^ To our delight, when **7** was treated with 10 mol % of Hoveyda–Grubbs second-generation catalyst (HG-II) in toluene at 110 °C, the desired *trans*-hydroazulenone **6** was obtained in 36% yield, along with 48% of the recovered starting material. Several commercially available RCM catalysts, including the molybdenum-based Shrock catalyst, were evaluated, showing that the HG-II was the most efficient (see Supporting Information). Further optimization revealed a significant solvent influence on the reaction outcome. Indeed, when cyclopentyl methyl ether (CPME) was used with 10 mol % of HG-II at 110 °C, product **6** was obtained in 63% yield, together with 27% of the recovered starting material **7**. After a single recycling, the combined yield of **6** reached 79%. The remaining transformation en route to oxalate **5** was the C9-regioselective Mukaiyama hydration, which was performed in 90% yield by using Mn(dpm)_3_ and an excess of phenylsilane under an oxygen atmosphere. Finally, the cesium semioxalate moiety was introduced via the 2-step sequence reported by Overman (see the Supporting Information).^23^

**Scheme 2:**
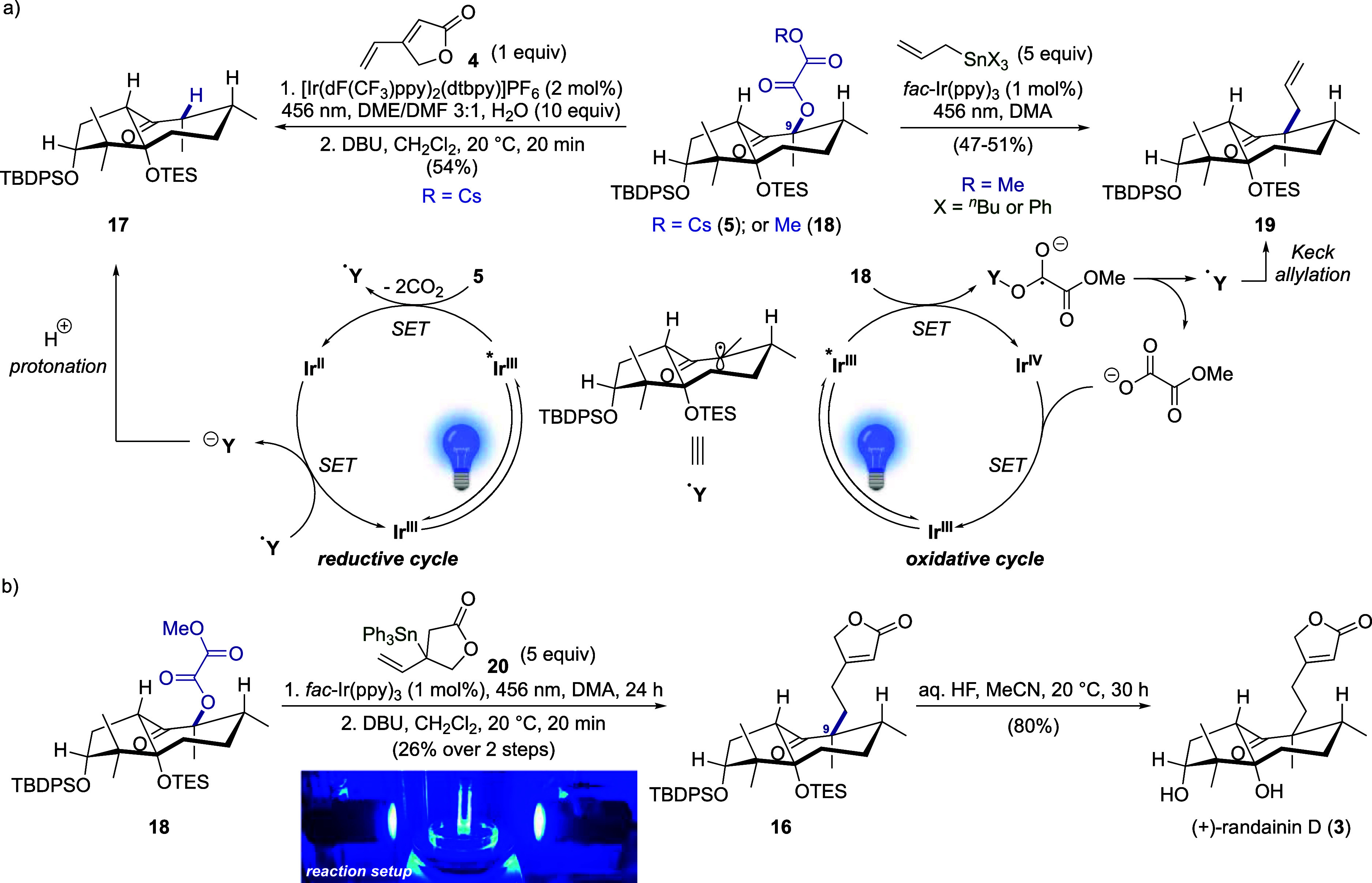
Development of the Ir^III^-Photocatalyzed Allylation and Application to the Total Synthesis of Randainin D

Unfortunately, the Giese coupling between **5** and **4** under the conditions developed by Overman and MacMillan delivered the protodeoxygenated ketone **17** as the main product in 54% yield (Scheme 2a, left). Only a trace amount of the desired butenolide **16** was observed. Attempts to conduct this transformation under water-free conditions or in the presence of other heteroleptic Ir^III^ catalysts were not fruitful. The formation of **17** can be rationalized as follows. The α-keto radical **Y**^**•**^, produced through oxidation of the oxalate by the excited photocatalyst via SET,^10^ can further engage the obtained Ir^II^ species to regenerate the ground-state Ir^III^ catalyst and form the enolate. The latter, upon protonation, leads to ketone **17**. Therefore, this Ir^III^-reductive cycle seems to be incompatible with the functionalization of α-keto radicals. On the other hand, in an Ir^III^-oxidative cycle, such an α-keto radical would not participate in the regeneration of the Ir^III^ ground state, as SET from the Ir^IV^ species would lead to the formation of a thermodynamically disfavored α-keto carbocation. Nevertheless, we envisioned that, by analogy with the Pereyre–Keck allylation,^24^ the α-keto radical generated under blue-light-promoted Ir^III^/Ir^IV^ catalysis could be trapped with an allylstannane, leading to the desired α-quaternary ketone. Allylations with allylmetal reagents proceeding via photoredox catalysis have been recently reported by groups of Reiser,^25^ Yasuda,^26^ and Woo.^27^ However, these transformations rely on the oxidation of α-halogenated aromatic ketones, producing primary α-keto radicals with a few examples of secondary carbon-centered radicals. A single precedent of deoxygenative functionalization, via a primary α-keto radical, was reported by Zeitler and co-workers,^28^ who showed that α-acetoxy aromatic ketones can be oxidized by photoexcited *fac*-Ir(ppy)_3_ in the presence of Lewis acids and trapped with substituted styrenes.

Inspired by these precedents, we focused on the use of alkyl oxalates to generate tertiary carbon-centered radicals via an Ir^III^-oxidative catalytic cycle.^29,30^ Compound **18** was prepared in 91% yield by treatment of α-hydroxyketone **15**, methyl chlorooxoacetate, and triethylamine in dichloromethane (see the Supporting Information). Irradiation of the reaction mixture containing oxalate **18**, allyltributylstannane, and *fac*-Ir(ppy)_3_ at 456 nm in DMA furnished the corresponding allylated product **19** in 47% yield (Scheme 2a, right). The yield was further increased to 51% when allyltriphenylstannane (5 equiv) was employed. The steric hindrance imposed by the two axial silyl groups efficiently shields the bottom face of radical **Y**^**•**^, which exclusively furnishes the C9-diastereomer **19** with the allyl group in the equatorial position. To apply the developed methodology for the synthesis of randainin D (**3**), we designed dihydrofuranone-based allyltin reagent **20** (see the Supporting Information). Methyl oxalate **18** and an excess of **20** were irradiated (456 nm) in the presence of *fac*-Ir(ppy)_3_ (Scheme 2b), which led to a mixture of isomeric olefins (*E*/*Z* and constitutional isomers). The crude mixture was treated with DBU in dichloromethane to promote double-bond conjugation,^31^ hence providing the desired C9-butenolide-bearing ketone **16** in 26% yield over two steps. Global deprotection of the silyl groups with aqueous HF in CH_3_CN afforded (+)-randainin D (**3**), the physicochemical properties of which fully matched those reported. Overall, randainin D was obtained in 17 steps in 4.4% yield from cyclopentanedione **10**. Of note, the current work allows confirmation of the proposed absolute configuration for randainin D, which was not determined in the isolation paper.

Encouraged by the successful Ir^III^-photocatalyzed allylation of methyl oxalate **18**, we wondered whether the developed methodology could be applied to functionalize other trisubstituted alkyl radicals. In particular, the absence of an electron-withdrawing group next to the carbon-centered radical would significantly alter the energy gap between the SOMO (radical) and HOMO (allylSnX_3_). Such a method would allow the construction of challenging allylated all-carbon quaternary centers from simple tertiary alcohols.

**Scheme 3:**
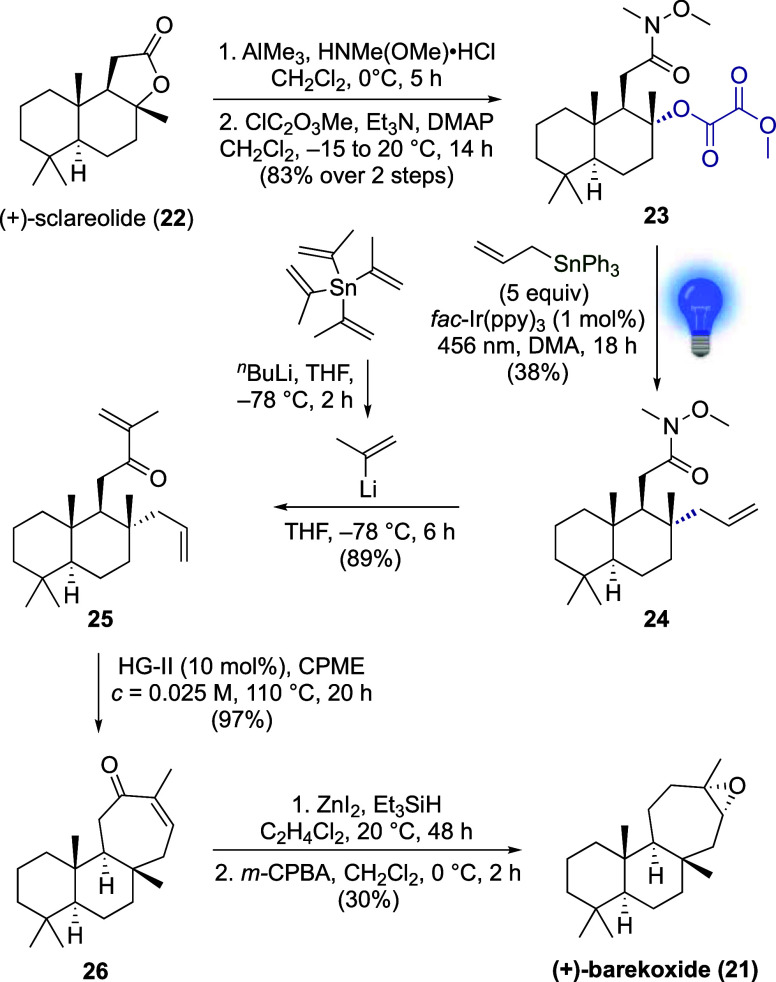
Seven-Step Synthesis of (+)-Barekoxide

The diterpene (+)-barekoxide (**21**) (Scheme 3) was isolated in 1992 from a marine sponge.^32^ In 2010, Sarpong and Davies reported the first and only enantioselective synthesis in 10 steps from (+)-sclareolide (**22**), in which the *trans*-fused cycloheptane ring was constructed via Rh^II^-catalyzed formal [4 + 3] cycloaddition, followed by hydrogenation.^33^ We hypothesized that barekoxide could be accessed via an alternative, and presumably shorter, route using the above Ir^III^-photocatalyzed allylation followed by enone RCM to close the *trans*-fused cycloheptane ring. Similar to the work by Sarpong and Davies, our synthesis started with commercially available (+)-sclareolide (**22**). Treatment of the latter with *N*-methoxymethylamine hydrochloride and AlMe_3_ provided a tertiary alcohol,^34^ which was then transformed to the corresponding methyl oxalate **23** in 83% yield over two steps, setting the ground for the Ir^III^-photocatalyzed allylation. When **23** (0.2 mmol) and an excess of allyltriphenylstannane were irradiated at 456 nm in the presence of 1 mol % of *fac*-Ir(ppy)_3_, the desired product **24** was obtained in 38% yield. Of note, the excess allyltin reagent can be recovered on chromatographic purification. The *cis*-diastereoisomer was not observed. Employing allyltributylstannane instead of allyltriphenylstannane led to a drastic yield decrease (11%, see Supporting Information). Then, the addition of in situ-generated isopropenyllithium to Weinreb amide **24** occurred in 89% yield, delivering the diene **25**. RCM was performed under the previously established conditions using 10 mol % of HG-II at 110 °C in CPME. The desired enone **26** was obtained in 97% yield, further illustrating the high efficiency of this reaction for the construction of *trans*-fused cycloheptane systems. Finally, the enone **26** was deoxygenated,^35^ producing the corresponding alkene, which, upon treatment with an excess of *m*-CPBA, led to (+)-barekoxide (**21**) (30% over 2 steps and 8.2% over 7 steps).

## Conclusions

In conclusion, we have completed the first enantioselective total synthesis of the diterpene (+)-randainin D (17 steps) and designed the shortest route (7 steps) toward (+)-barekoxide. Key to these syntheses was the combination of RCM of substituted enones with a novel Ir^III^-photocatalyzed deoxygenative allylation. This study expands the toolbox of synthetic strategies for the construction of polycyclic systems bearing *trans*-cycloheptane scaffolds including complex terpenoids. Additionally, it features an innovative approach for the late-stage incorporation of the β-substituted butenolide moiety, found in numerous natural products.

## Supporting Information Available

Additional experimental details, materials and methods, and copies of NMR spectra (PDF)
