# Supplementary material for: Total Synthesis of the Diterpenes (+)-Randainin D and (+)-Barekoxide via Photoredox-Catalyzed Deoxygenative Allylation
Source: J Am Chem Soc. 2024 Apr 15;146(16):11486–92. doi: 10.1021/jacs.4c02224 (PMC11046436; doi:10.1021/jacs.4c02224)

Supporting Information

Total Synthesis of the Diterpenes (+)-Randainin D and (+)-Barekoxide via Photoredox-Catalyzed Deoxygenative Allylation

Oleksandr Vyhivskyi, Olivier Baudoin*

Department of Chemistry, University of Basel, St. Johanns-Ring 19, CH-4056 Basel, Switzerland

*Email: olivier.baudoin@unibas.ch

Table of Contents

General information 4

List of abbreviations 5

Total synthesis of (+)-Randainin D (**3**) 7

Synthesis of (*R*)-3-((tert-butyldiphenylsilyl)oxy)-2,2-dimethylcyclopentan-1-one (**9**) 7

Synthesis of (*R*)-4-((tert-butyldiphenylsilyl)oxy)-5,5-dimethylcyclopent-2-en-1-one (**11**)... 9

Synthesis of (*R*)-4-((tert-butyldiphenylsilyl)oxy)-2-(hydroxymethyl)-5,5-dimethylcyclopent-2-en-1-one (**12**) 11

Synthesis of (3R,5R)-3-((tert-butyldiphenylsilyl)oxy)-2,2-dimethyl-5-(((triethylsilyl)oxy)methyl)cyclopentan-1-one (**8**) 13

Synthesis of tert-butyl(((1R,3S,4R)-2,2-dimethyl-3-(3-methylbut-3-en-1-yl)-3-((triethylsilyl)oxy)-4-(((triethylsilyl)oxy)methyl)cyclopentyl)oxy)diphenylsilane (**13**)...... 16

Synthesis of (1S,2S,4R)-4-((tert-butyldiphenylsilyl)oxy)-3,3-dimethyl-2-(3-methylbut-3-en-1-yl)-2-((triethylsilyl)oxy)cyclopentane-1-carbaldehyde (**14**) 18

Synthesis of 1-((1S,2S,4R)-4-((tert-butyldiphenylsilyl)oxy)-3,3-dimethyl-2-(3-methylbut-3-en-1-yl)-2-((triethylsilyl)oxy)cyclopentyl)-2-methylprop-2-en-1-one (**7**) 20

Synthesis of (2R,3aS,8aS)-2-((tert-butyldiphenylsilyl)oxy)-1,1,5,6-tetramethyl-8a-((triethylsilyl)oxy)-2,3,3a,7,8,8a-hexahydroazulen-4(1H)-one (**6**) 22

Synthesis of (2R,3aS,5S,6R,8aS)-2-((tert-butyldiphenylsilyl)oxy)-5-hydroxy-1,1,5,6-tetramethyl-8a-((triethylsilyl)oxy)octahydroazulen-4(1H)-one (**5**) 23

Iridium(III)-catalyzed deoxyfunctionalization 24

Synthesis of (2R,3aS,5S,6R,8aS)-2-((tert-butyldiphenylsilyl)oxy)-1,1,5,6-tetramethyl-4-oxo-8a-((triethylsilyl)oxy)decahydroazulen-5-yl (2-(trimethylsilyl)ethyl) oxalate **(SI-7)** 24

Synthesis of 4-vinylfuran-2(5H)-one (**4**) 25

Giese-coupling of **SI-7** and **4**: 27

Synthesis of (2R,3aS,5S,6R,8aS)-2-((tert-butyldiphenylsilyl)oxy)-1,1,5,6-tetramethyl-4-oxo-8a-((triethylsilyl)oxy)decahydroazulen-5-yl methyl oxalate (**18**) 28

Synthesis of (2R,3aS,5S,6R,8aS)-5-allyl-2-((tert-butyldiphenylsilyl)oxy)-1,1,5,6-tetramethyl-8a-((triethylsilyl)oxy)octahydroazulen-4(1H)-one (**19**) 29

Synthesis of 4-(triphenylstannyl)-4-vinyldihydrofuran-2(3H)-one (**20**) 30

Synthesis of 4-(2-(phenylselanyl)ethyl)furan-2(5H)-one (**SI-10**): 30

Synthesis of 4-(2-(phenylselanyl)ethyl)-4-(triphenylstannyl)dihydrofuran-2(3H)-one (**SI-12**) 31

Synthesis of 4-(2-((2R,3aS,5S,6R,8aS)-2-((tert-butyldiphenylsilyl)oxy)-1,1,5,6-tetramethyl-4-oxo-8a-((triethylsilyl)oxy)decahydroazulen-5-yl)ethyl)furan-2(5H)-one (**16**) 33

Synthesis of (+)-Randainin D (**3**) 35

(+)-Randainin D (**3**) analytical data comparison 36

Total synthesis of (+)-Barekoxide (**21**) 37

Synthesis of 2-((1R,2R,4aS,8aS)-2-hydroxy-2,5,5,8a-tetramethyldecahydronaphthalen-1-yl)-N-methoxy-N-methylacetamide **(SI-14)** 37

Synthesis of (1R,2R,4aS,8aS)-1-(2-(methoxy(methyl)amino)-2-oxoethyl)-2,5,5,8a-tetramethyldecahydronaphthalen-2-yl methyl oxalate **(23)** 38

Synthesis of 2-((1S,2S,4aS,8aS)-2-allyl-2,5,5,8a-tetramethyldecahydronaphthalen-1-yl)-N-methoxy-N-methylacetamide (**24**) 39

Synthesis of 1-((1S,2S,4aS,8aS)-2-allyl-2,5,5,8a-tetramethyldecahydronaphthalen-1-yl)-3-methylbut-3-en-2-one (**25**) 40

Synthesis of (4aS,6aS,11aS,11bS)-4,4,6a,9,11b-pentamethyl-1,2,3,4,4a,5,6,6a,7,11,11a,11b-dodecahydro-10H-cyclohepta[a]naphthalen-10-one (**26**) 41

Synthesis of (+)-Barekoxide (**21**) 42

(+)-Barekoxide (**21**) analytical data comparison 43

References 45

General information

General methods:

All reactions were performed under an argon atmosphere in oven-dried glassware sealed with a rubber septum unless specified otherwise. Reactions were stirred magnetically and monitored by thin-layer chromatography (TLC) and TLC-Ms. Analytical thin layer chromatography was performed using pre-coated Merck silica gel 60 F254 plates (0.25 mm) and was visualized by UV (254 nm) and stained with aqueous potassium permanganate ($KMnO_4$) [2.25 g $KMnO_4$, 300 mL H_2O, 15 g K_2CO_3, 2 mL 1M aqueous NaOH]. Concentration under reduced pressure (= *in vacuo*) was performed by rotatory evaporation at 40 °C (unless otherwise stated) at the appropriate pressure. Chromatographic purification was performed using silica gel (230-400 mesh) at 0.3 – 0.5 bar nitrogen over-pressure. Yields refer to the purified compound. Graphics for the reaction schemes and figures in the original communication and supporting information were created with BioRender.com.

Chemicals and solvents:

All chemicals and solvents were purchased from ACROS, SIGMA-ALDRICH, COMBIBLOCKS, FLUOROCHEM, FLUKA, MERCK, FISHER-SCIENTIFIC, TCI or STREM and were used as received from the commercial supplier without further purification. THF, Et_2O, DCM, MeCN, and toluene were dried on an INNOVATIVE TECHNOLOGY PURE SOLVE PS-MD-5 solvent purification system under N_2. (H_2O content < 10 ppm, Karl–Fischer titration). Anhydrous CPME, DMA, DMF, THF, Et_2O, and DCM were purchased from ACROS and degassed by argon bubbling over 60 minutes, or via freeze-pump-thaw procedure before use. *fac*-Ir(ppy$_3$)$_3$, (*R,R*)-Ts-DENEB®, and Shrock's catalyst were purchased from STREM. (Ir[dF(CF$_3$)ppy]$_2$(dtbpy))PF$_6$, Hoveyda-Grubbs catalysts® (M720, M721) and nitro-Grela catalyst were purchased from SIGMA-ALDRICH.

Instrumentation:

Melting points were obtained on a Büchi melting point M-565 and are uncorrected. IR spectra were recorded on an ATR Varian Scimitar 800 and reported in reciprocal centimeters (cm^{-1}). Nuclear magnetic resonance spectra were recorded on a Bruker Advance 400 and 500 (400 and 500 MHz). Measurements were carried out at ambient temperature (ca. 22 °C). Chemical shifts (δ) are reported in ppm with the residual solvent signal as internal standard ($CHCl_3$ at 7.26 and 77.16 ppm for ^1H and ^{13}C NMR spectroscopy, respectively; C_6H_6 at 7.16 and 128.06 ppm) unless otherwise noted. Data are reported in parts per million (ppm) as follows: chemical shift, multiplicity (s = singlet, d = doublet, t = triplet, q = quartet, quint = quintuplet, sept = septuplet, m = multiplet and brs = broad singlet), coupling constant in Hz and integration. 13C NMR spectra were recorded with broadband ^1H-decoupling. High-resolution mass spectra were recorded by Dr. M. Pfeffer (Department of Chemistry, University of Basel) on a Bruker maXis 4G QTOF ESI mass spectrometer. Optical rotations were measured on a Perkin Elmer 341 Polarimeter in a 0.7 mL micro cuvette (cell length 100mm) with NaD-Line (λ = 589 nm) at 20 or 25 °C with c = 1.00 corresponds to g/100 mL. The enantiomeric excesses were determined by employing a chiral HPLC. HPLC analyses were performed using a Shimadzu Prominence system with SIL-20A auto sample, CTO-20AC column oven, LC-20AD pump system, DGU20A3 degasser and SPD-M20A Diode Array or UV/VIS detector. The following chiral

columns from Daicel Chemical Industries were used: ODH (Chiralcel®), and IA (Chiralpak®) in 4.6 x 250 mm size. TLC-MS measurements were performed using an Advion Plate ExpressTM TLC Plate Reader and an Advion Expression LCMS (MeOH flow, ESI-Source).

Deoxygentaive photoredox transformations were performed using two 40 W blue LED lamps (Kessil PR 160 L, 456 nm). Lamps were positioned on both sides at the 10 cm distance from the catalysis tube. When specified, cooling was performed using household fan (temperature without cooling 45 °C, with cooling 38 – 40 °C).

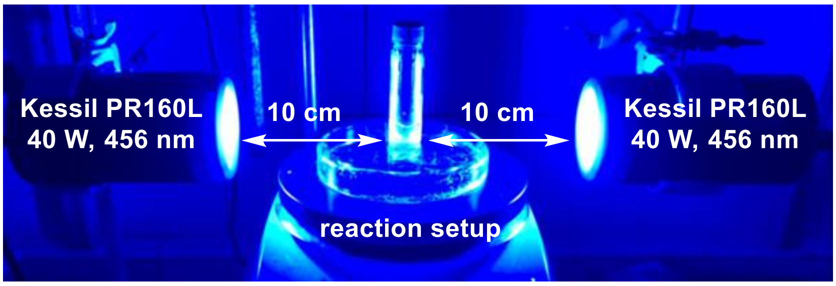

List of abbreviations

2-MeTHF – 2-methyltetrahydrofuran

AcN – acetonitrile

CPME – cyclopentyl methyl ether

DBU – 1,8-diazabicyclo(5.4.0)undec-7-ene

DCE – 1,2-dichloroethane

DCM – dichloromethane

DMA – dimethylacetamide

DMAP – 4-dimethylaminopyridine

DMF – dimethylformamide

DMP – Dess–Martin periodinane

DMSO – dimethyl sulfoxide

HPLC – high-performance liquid chromatography

IBX – 2-iodoxy benzoic acid

Im – imidazole

m-CPBA – meta-chloroperoxybenzoic acid

NBS – N-bromosuccinimide

NMO – N-methylmorpholine N-oxide

P – product

PCC – pyridinium chlorochromate

PE – petroleum ether

Ppy – 2-phenylpyridine

r.t. – room temperature (20 °C)

SM – starting material

TBDPSCl – tert-butyl(chloro)diphenylsilane

TEA – triethylamine

TESCl – chlorotriethylsilane

TESOTf – triethylsilyl trifluoromethanesulfonate

THF – tetrahydrofuran

TLC-MS – thin-layer chromatography mass spectrometry

TMS – trimethylsilyl group

Total synthesis of (+)-Randainin D (**3**)

Synthesis of (*R*)-3-((tert-butyldiphenylsilyl)oxy)-2,2-dimethylcyclopentan-1-one (**9**)

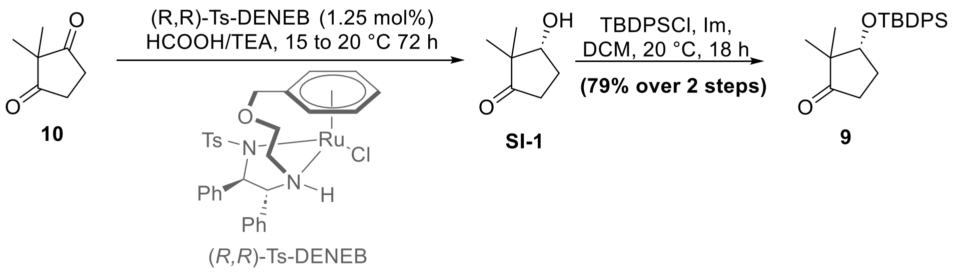

Modification of the procedure reported by Chiu et al.[1]

In the glovebox, (*R,R*)-Ts-DENEB (402 mg, 0.62 mmol, 1.25 mol%, purchased from STREM) was added to a 100 mL round-bottom flask, equipped with a stirring bar and containing commercially available ketone **10** (6.5 g, 51.5 mmol). The flask was sealed with a rubber septum and removed from the glove box. It was then placed in a 15°C cooling bath (temperature controlled with cryostat). Then, a precooled to 15 °C mixture of 9.25 mL of formic acid and 9.25 mL of triethylamine was slowly added to the reaction flask. After the addition was complete, the reaction mixture was stirred for 48 h at 15 ºC. *CAUTION*: gas evolution was observed throughout the reaction; the flask must be nicely sealed with the appropriate rubber septum. After 48 h, the reaction mixture was warmed to room temperature (25 °C) and stirred for an additional 24 h to ensure complete consumption of the starting material. Then, the mixture was diluted with 50 mL of water and extracted with EtOAc (75 mL x3). Combined organic fractions were washed two times with sat. aq. $NaHCO_3$ (100 mL), then two times with sat. aq. NH_4Cl solution (100 mL) and, finally, once with brine (100 mL). The organic extract was dried over the anhydrous Na_2SO_4 and concentrated *in vacuo*. 100 mg aliquote was taken to obtain an analytical sample of the **SI-1** (column chromatography, gradient elution with 5:1 (PE:EtOAc) to 1:1 (PE:EtOAc))

Analytical data SI-1:

^{1}H NMR (400 MHz, $CDCl_3$): δ 4.05 ("dt", *J* = 7.7, 3.6 Hz, 1H), 2.53 – 2.41 (m, 1H), 2.30 – 2.19 (m, 2H), 1.95 – 1.84 (m, 1H), 1.80 (d, *J* = 3.4 Hz, 1H), 1.03 (s, 6H). **^{13}C NMR (101 MHz, $CDCl_3$):** δ 221.2, 78.4, 50.2, 34.3, 27.8, 22.3, 16.9.
$[\alpha]_D^{20}$ = -12.0 (c = 1.44, $CHCl_3$) [reported:[2] **$[\alpha]_D^{25}$** = -12.4 (c = 1.3, $CHCl_3$)]; e.r. was measured after the protection with TBDPS, by comparing to the racemic reference, using chiral HPLC.
Analytical data are identical to those previously reported.[2,3]

The obtained residue (placed in 250 mL round-bottom flask) was placed in a 250 mL round-bottom flask, mixed with imidazole (8.84 g, 103 mmol, 2 equiv), DMAP (1.26 g, 10.3 mmol, 0.2 equiv), then dissolved in 100 mL of DCM (ca. 0.5 M), cooled down to 0 °C using an ice-water bath and treated with TBDPSCl (16.98 g, 61.8 mmol, 16.1 mL, 1.2 equiv; dropwise addition, using syringe pump, rate 1 mL/min). The reaction mixture was left to stir for 18 hours in the ice-water bath, slowly warming up to room temperature. Then, it was mixed with a 0.5 M HCl solution (100 mL), the DCM fraction was separated, and the aqueous phase was

extracted with DCM (75 mL x3). The combined organic fractions were successively washed with sat. aq. $NaHCO_3$ (200 mL) and brine (100 mL). It was then dried over anhydrous Na_2SO_4, filtered, and concentrated *in vacuo*. The obtained residue was purified using column chromatography (gradient elution 80:1 (PE:EtOAc) to 50:1 (PE:EtOAc)) to afford **9** (14.95 g, 79%, e.r. = 99.4:0.6) as a colorless oil.

Experimental Notes:

- Ketone **10** can also be prepared using the previously reported by Gribble group[4] procedure. To ensure high yield, peroxides-free dioxane must be used!

Analytical data 9:

^{1}H NMR (500 MHz, $CDCl_3$): δ 7.71 – 7.64 (m, 4H), 7.48 – 7.42 (m, 2H), 7.41 – 7.36 (m, 4H), 4.03 ("t", *J* = 6.0 Hz, 1H), 2.38 (dddd, *J* = 19.2, 9.3, 5.4, 0.6 Hz, 1H), 2.03 (ddd, *J* = 19.2, 9.0, 7.9 Hz, 1H), 1.89 – 1.72 (m, 2H), 1.08 (s, 12H), 0.91 (s, 3H). **^{13}C NMR (126 MHz, $CDCl_3$):** δ 221.2, 136.1, 136.0, 134.4, 133.6, 130.0, 129.9, 127.8, 127.8, 79.6, 50.7, 34.6, 28.1, 27.1, 22.1, 19.5, 17.9.
IR (neat): ν 2963, 2932, 1743, 1468, 1428, 1111, 1086, 703.
HRMS (ESI): calcd for $C_{23}H_{30}O_2Si$ $[M+Na]^+$: 389.1907, found: 389.1904
TLC: Rf = 0.20 (20:1 PE/Et_2O, $KMnO_4$, or UV 254 nm).
$[\alpha]_D^{25}$ = 0.84 (c = 1.9, $CHCl_3$).
e.r. = 99.4:0.6; column – ODH, eluent – 95(Heptane):5(*i*-PrOH), flow 1 mL, 20 min

<Chromatogram>

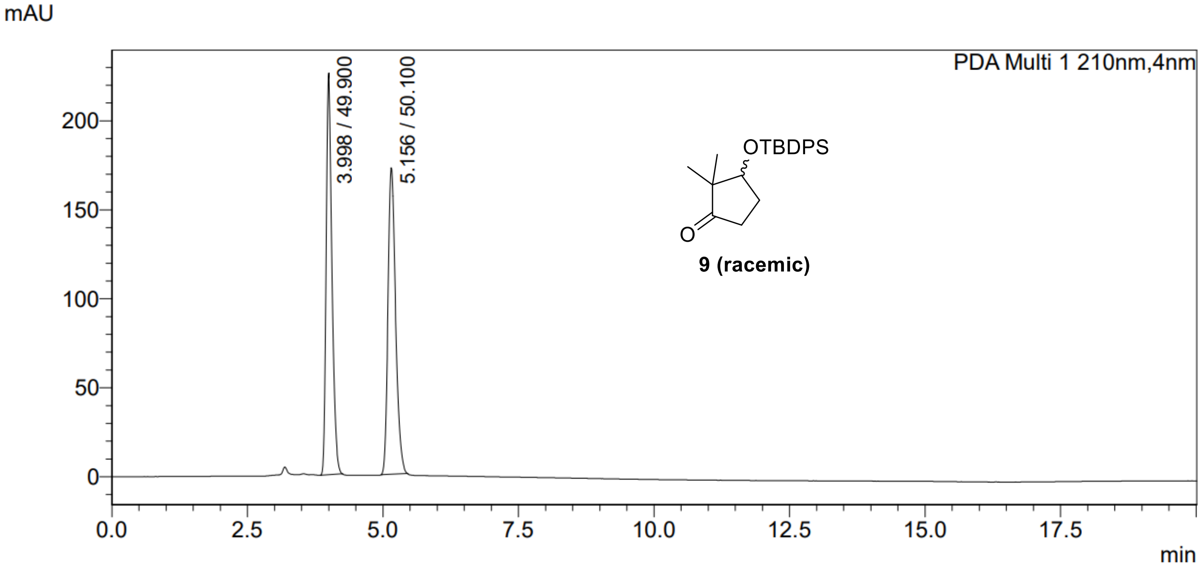

<Peak Table>

PDA Ch1 210nm

Peak#	Ret. Time	Area	Height	Area%
1	3.998	1675124	225644	49.900
2	5.156	1681864	172265	50.100
Total		3356988	397909	100.000

<Chromatogram>

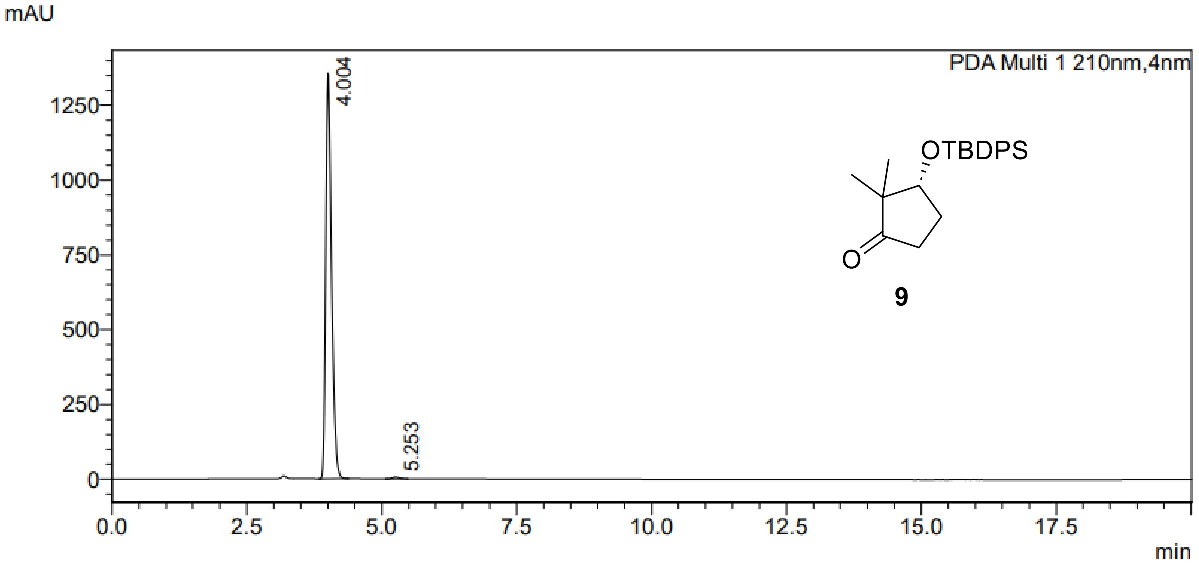

<Peak Table>

PDA Ch1 210nm

Peak#	Ret. Time	Area	Height	Area%
1	4.004	10152083	1356437	99.443
2	5.253	56903	6132	0.557
Total		10208985	1362568	100.000

Synthesis of (*R*)-4-((tert-butyldiphenylsilyl)oxy)-5,5-dimethylcyclopent-2-en-1-one (11)

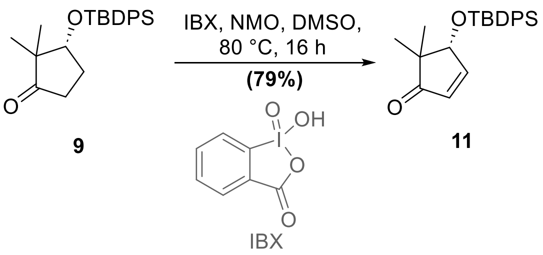

Ketone **9** (14.1 g, 38.46 mmol) was placed in a 1 L round-bottom flask equipped with a stirring bar and mixed with NMO (13.52 g, 115.38 mmol, 3.0 equiv) and 2-iodoxy benzoic acid (IBX, 32.3 g, 115.38 mmol, 3.0 equiv), then dissolved in anhydrous DMSO (385 mL, 0.1 M). The reaction vessel was heated to 80 °C. After 14 hours, the reaction was cooled to 0 °C and sat. aq. $NaHCO_3$ (300 mL) was slowly added. The formed mixture was stirred for 30 minutes and filtered over a short celite pad. The fritted glass filter was extensively washed with EtOAc. The EtOAc layer was removed from the obtained mixture using a separatory funnel. The aqueous phase was further extracted with EtOAc (100 mL x3). Combined organic fractions were successively washed with sat. aq. $NaHCO_3$ (200 mL) and with brine (200 mL), dried over anhydrous Na_2SO_4, filtered, and concentrated *in vacuo*. The obtained residue was purified using column chromatography (gradient elution 70:1 (PE:EtOAc) to 40:1 (PE:EtOAc)) to afford **11** (11.07 g, 79%, e.r. = 99.4:0.6) as a colorless solid.

Experimental Notes:

- Freshly prepared 2-iodoxy benzoic acid (≥ 99% purity) was used.[5]

^{1}H NMR (500 MHz, $CDCl_3$): δ 7.71 – 7.68 (m, 4H), 7.49 – 7.45 (m, 2H), 7.43 – 7.39 (m, 4H), 7.07 (dd, J = 5.9, 2.1 Hz, 1H), 6.04 (dd, J = 5.9, 1.4 Hz, 1H), 4.57 (dd, J = 2.1, 1.4 Hz, 1H), 1.14 (s, 3H), 1.10 (s, 9H), 0.89 (s, 3H). **^{13}C NMR (126 MHz, $CDCl_3$):** δ 211.9, 161.4, 136.1, 136.0, 133.9, 133.1, 132.0, 130.3, 130.2, 128.0, 128.0, 80.7, 49.1, 27.1, 22.5, 21.5, 19.5.
IR (neat): ν 2932, 2859, 1716, 1463, 1427, 1106, 1065, 857, 702.
HRMS (ESI): calcd for $C_{23}H_{28}O_2Si$ $[M+Na]^+$: 387.1751, found: 387.1751
TLC: Rf = 0.17 (20:1 PE/Et_2O, $KMnO_4$, or UV 254 nm).
$[\alpha]_D^{25}$ = -49.3 (c = 1.14, $CHCl_3$).
Melting point: 64 °C
e.r. = 99.4:0.6; column – IA, eluent – 99.7(Heptane):0.3(*i*-PrOH), flow 1 mL, 30 min

<Chromatogram>

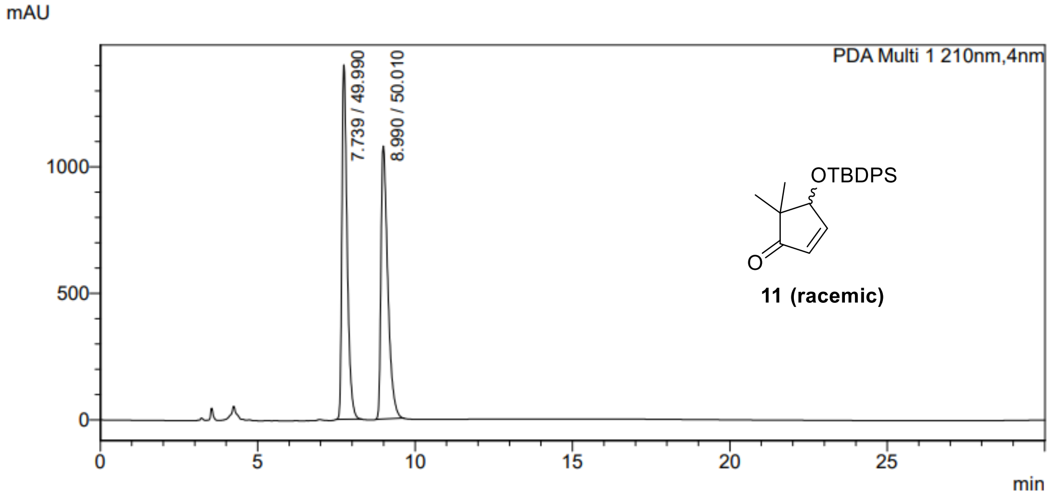

<Peak Table>

PDA Ch1 210nm

Peak#	Ret. Time	Area	Height	Area%
1	7.739	16204292	1399995	49.990
2	8.990	16210828	1078291	50.010
Total		32415120	2478287	100.000

<Chromatogram>

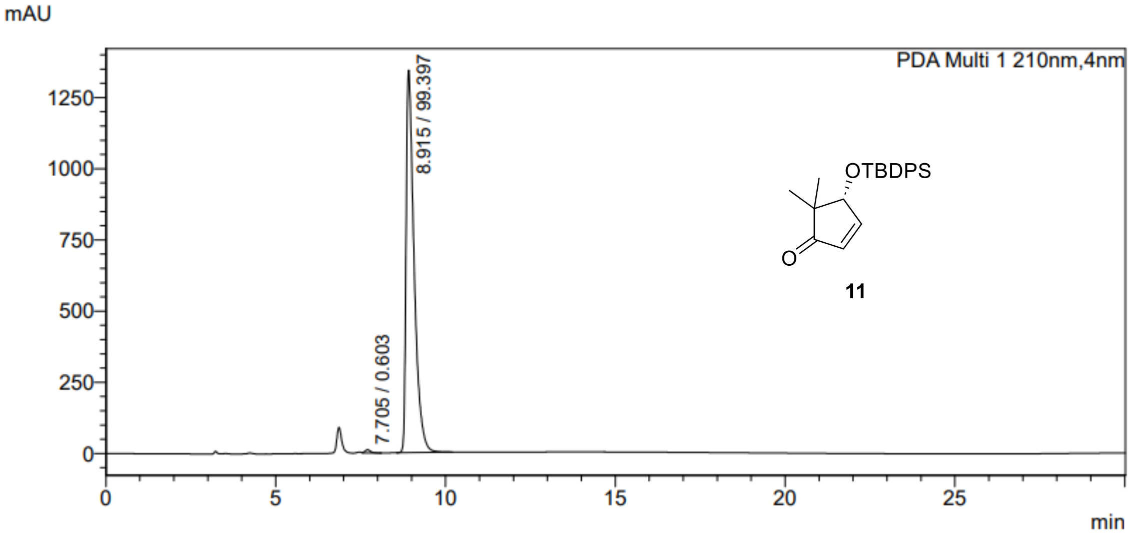

<Peak Table>

PDA Ch1 210nm

Peak#	Ret. Time	Area	Height	Area%
1	7.705	133994	11838	0.603
2	8.915	22069499	1343444	99.397
Total		22203493	1355282	100.000

Synthesis of (*R*)-4-((tert-butyldiphenylsilyl)oxy)-2-(hydroxymethyl)-5,5-dimethylcyclopent-2-en-1-one (**12**)

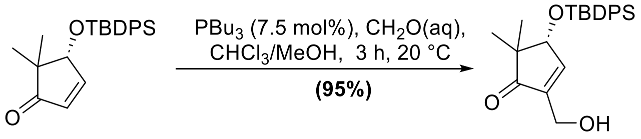

11 **12**

Enone **11** (10.3 g, 28.25 mmol, 1.00 equiv) was introduced into the 250 mL round-bottom flask and dissolved in $CHCl_3$ (34.5 mL) and MeOH (23 mL). Formaldehyde was added to the obtained mixture (37% solution in H_2O, 3.16 mL, 42.4 mmol, 1.5 equiv) at r.t. The resulting solution was degassed using three freeze-pump-thaw cycles and then cooled to 15 °C. In the glovebox, tributyl phosphine (429 mg, 2.12 mmol, 0.520 mL, 7.5 mol%) was dissolved in degassed $CHCl_3$ (30 mL) and added dropwise over 5 minutes to the stirred reaction mixture. After one hour at 15 °C, additional formaldehyde (37% solution in H_2O, 1.05 mL, 14.1 mmol, 0.5 equiv) and tributyl phosphine (143 mg, 0.71 mmol, 173 µl, 2.5 mol%) solution in 1 mL of degassed $CHCl_3$ were added to the reaction mixture. The stirring continued for 90 min at 15 °C and 30 minutes at r.t. The reaction mixture was directly dried by adding anhydrous Na_2SO_4, filtered, and concentrated *in vacuo*. The obtained residue was purified using column chromatography (gradient elution 10:1 (PE:EtOAc) to 5:1 (PE:EtOAc)) to afford **12** (10.58 g, 95%, e.r. = 99.5:0.5) as a white solid.

Experimental Notes:

- PPh_2Me resulted in a 47% yield, and 31% of SM was recovered.
- Prolongated reaction times result in lower yields due to the decomposition of the product.
- When the reaction mixture was not degassed, the desired product was generally obtained in an 80% yield.
- Higher dilution of the reaction mixture leads to decreased yields.

^{1}H NMR (500 MHz, $CDCl_3$): δ 7.71 – 7.68 (m, 4H), 7.49 – 7.45 (m, 2H), 7.43 – 7.40 (m, 4H), 6.90 (dt, *J* = 2.2, 1.4 Hz, 1H), 4.54 ("q", *J* = 1.9 Hz, 1H), 4.34 – 4.26 (m, 2H), 2.08 (t, *J* = 6.1 Hz, *OH*), 1.13 (s, 3H), 1.10 (s, 9H), 0.86 (s, 3H). **^{13}C NMR (126 MHz, $CDCl_3$):** δ 211.4, 155.0, 142.7, 136.1, 136.0, 133.8, 133.2, 130.3, 130.2, 128.0, 128.0, 79.3, 57.8, 50.1, 27.1, 22.4, 21.5, 19.5.
IR (neat): ν 2932, 2860, 1704, 1463, 1428, 1385, 1084, 703.
HRMS (ESI): calcd for $C_{24}H_{30}O_3Si$ $[M+Na]^+$: 417.1856, found: 417.1859
TLC: Rf = 0.29 (4:1 PE/EtOAc, $KMnO_4$, or UV 254 nm).
$[\alpha]_D^{25}$ = -47.6 (c = 1.06, $CHCl_3$).
Melting point: 77 °C
e.r. = 99.5:0.5; column – ODH, eluent – 99(Heptane):1(*i*-PrOH), flow 1.5 mL, 40 min

<Chromatogram>

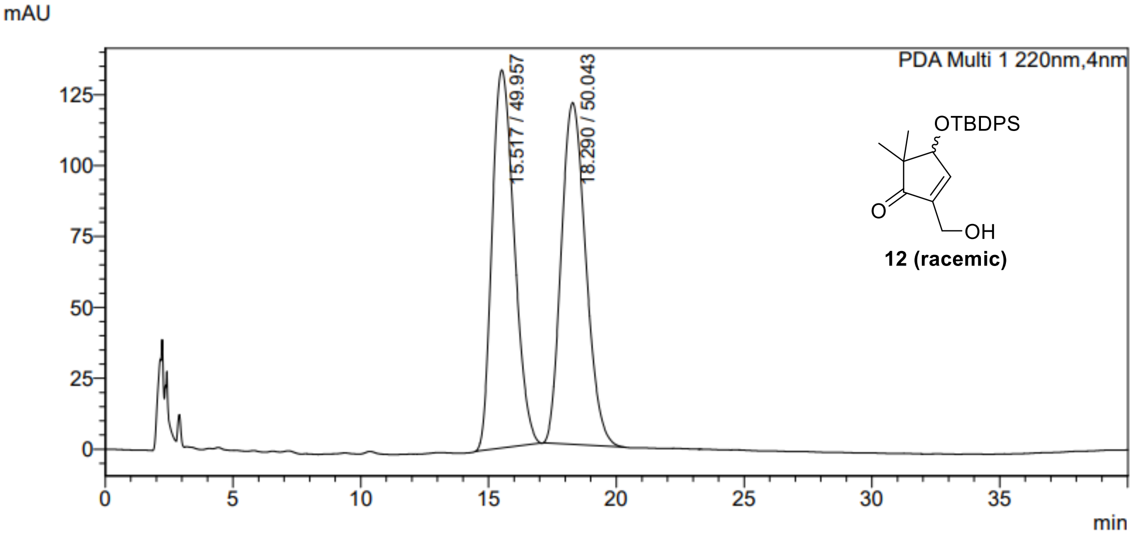

<Peak Table>

PDA Ch1 220nm

Peak#	Ret. Time	Area	Height	Area%
1	15.517	8011089	133310	49.957
2	18.290	8024908	120531	50.043
Total		16035997	253841	100.000

<Chromatogram>

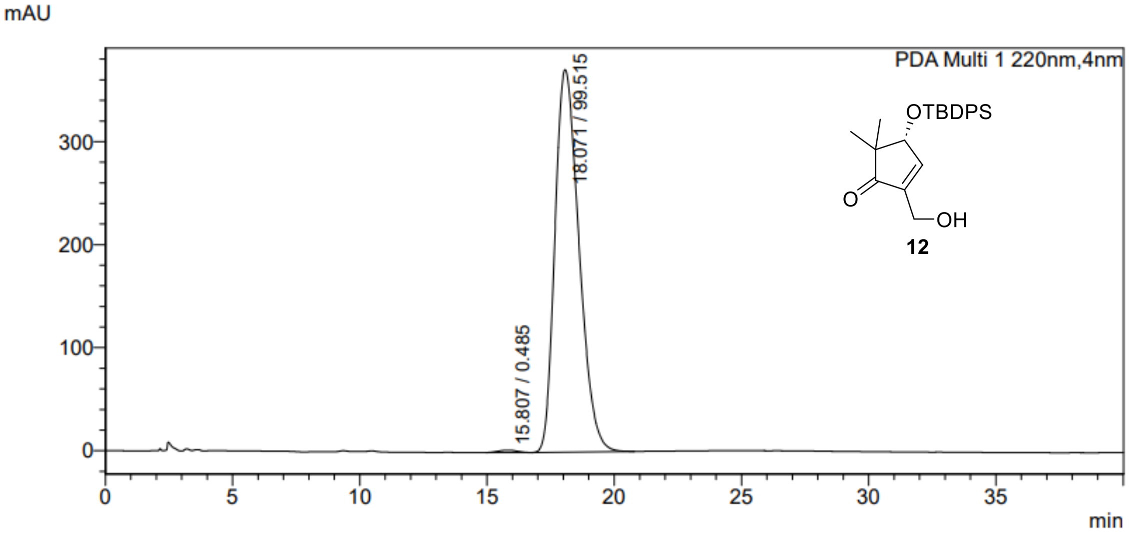

<Peak Table>

PDA Ch1 220nm

Peak#	Ret. Time	Area	Height	Area%
1	15.807	119134	2302	0.485
2	18.071	24435458	371371	99.515
Total		24554591	373673	100.000

Synthesis of (3R,5R)-3-((tert-butyldiphenylsilyl)oxy)-2,2-dimethyl-5-(((triethylsilyl)oxy)methyl)cyclopentan-1-one (**8**)

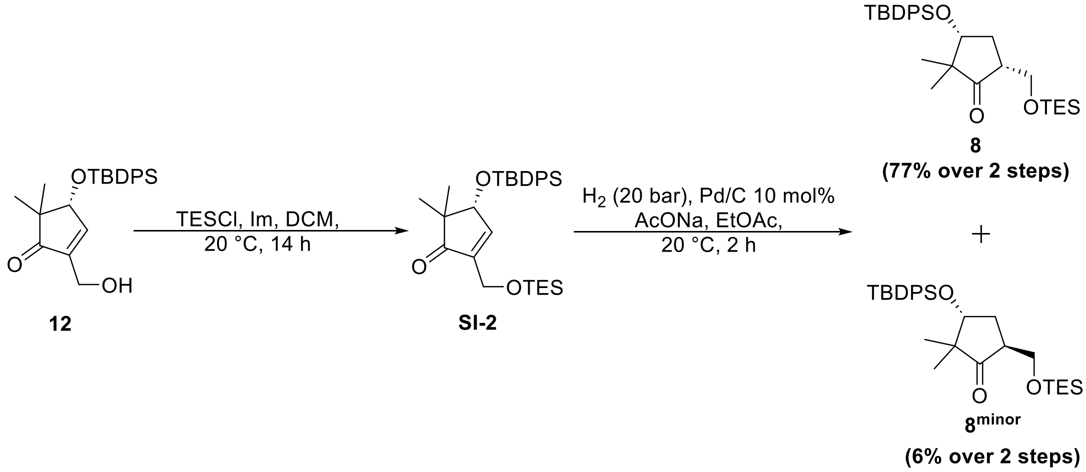

Alcohol **12** (3.4 g, 8.6 mmol, 1.00 equiv) and imidazole (1.17 g, 17.2 mmol, 2 eq) were introduced into a 100 mL round-bottom flask equipped with a stirring bar. The flask was evacuated and backfilled with Ar (3 times). Anhydrous DCM (43 mL, 0.2 M) was added, and the solution was cooled to 0 °C using a water-ice bath. TESCl (1.56 g, 10.3 mmol, 1.74 mL, 1.2 equiv) was added dropwise using a syringe pump. (rate 0.2 mL/min). The reaction mixture was left to stir for 14 hours in the ice-water bath, where it slowly warmed up to room temperature. Then, it was diluted with 50 mL of DCM, transferred to the separatory funnel, and washed once with sat. aq. $NaHCO_3$ (100 mL), then it was washed three times with sat. aq. NH_4Cl (50 mL). Combined NH_4Cl fractions were extracted once with DCM (50 mL). The obtained organic fractions were combined, washed with brine (50 mL), dried over anhydrous Na_2SO_4, filtered, and concentrated *in vacuo*. The obtained crude material **SI-2** (3.8 g, 87%) was essentially pure and directly engaged in the hydrogenation step.

Analytical data SI-2:

^{1}H NMR (500 MHz, $CDCl_3$): δ 7.71 – 7.68 (m, 4H), 7.48 – 7.44 (m, 2H), 7.42 – 7.38 (m, 4H), 6.91 (q, *J* = 2.0 Hz, 1H), 4.51 ("dt", *J* = 2.8, 2.0 Hz, 1H), 4.33 ("dt", *J* = 16.1, 1.9 Hz, 1H), 4.27 ("ddd", *J* = 16.2, 2.8, 1.9 Hz, 1H), 1.13 (s, 3H), 1.10 (s, 9H), 0.95 – 0.87 (m, 12H), 0.56 (q, *J* = 7.8 Hz, 6H).**^{13}C NMR (126 MHz, $CDCl_3$):** δ 210.1, 154.6, 144.4, 136.1, 136.0, 134.0, 133.3, 130.2, 130.1, 128.0, 127.9, 79.1, 57.7, 50.3, 27.1, 22.6, 21.5, 19.5, 6.9, 4.5
IR (neat): ν 2958, 2877, 1710, 1463, 1428, 1111, 1085, 631.
HRMS (ESI): calcd for $C_{30}H_{44}O_3Si_2$ $[M+Na]^+$: 531.2721, found: 531.2726
TLC: Rf = 0.31 (20:1 PE/Et_2O, $KMnO_4$, or UV 254 nm).
$[\alpha]_D^{25}$ = -56.7 (c = 1.5, $CHCl_3$).
e.r. = 99.5:0.5; column – ODH, eluent – 99.9(Heptane):0.1(*i*-PrOH), flow 0.5 mL, 30 min

<Chromatogram>

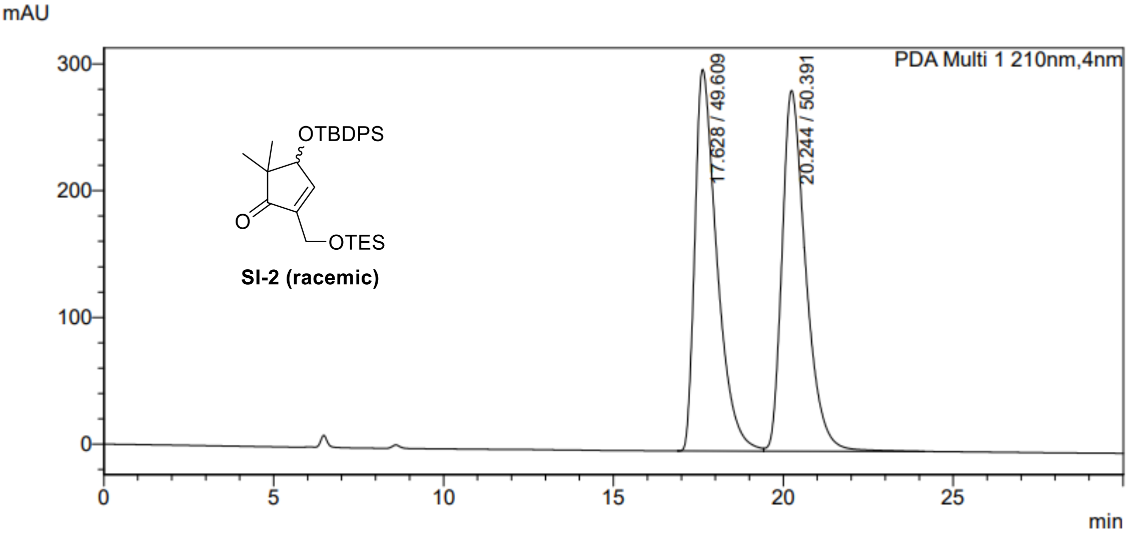

<Peak Table>

PDA Ch1 210nm

Peak#	Ret. Time	Area	Height	Area%
1	17.628	13574261	301101	49.609
2	20.244	13788174	284571	50.391
Total		27362435	585671	100.000

<Chromatogram>

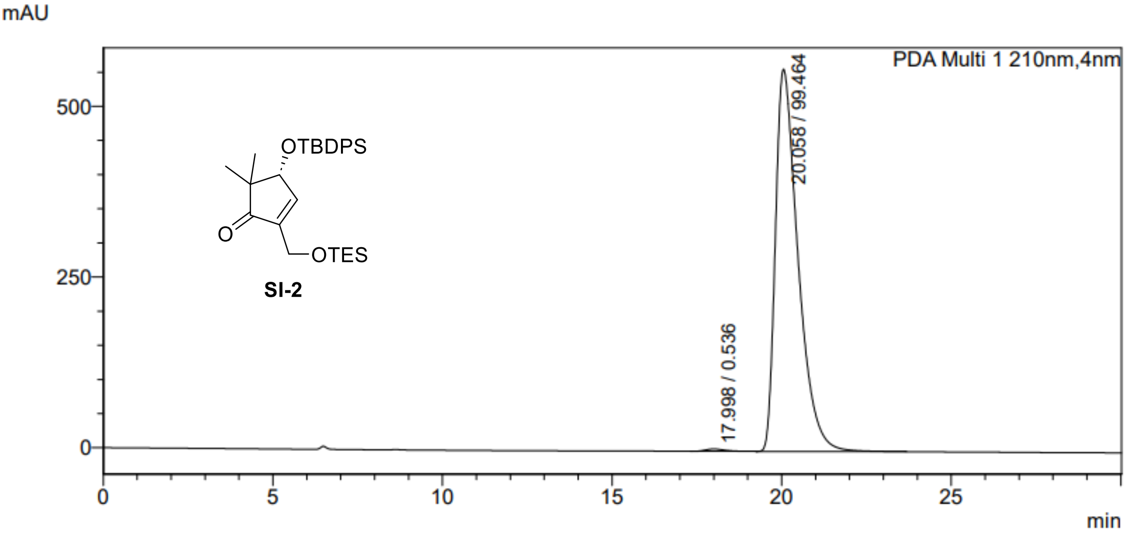

<Peak Table>

PDA Ch1 210nm

Peak#	Ret. Time	Area	Height	Area%
1	17.998	139698	3504	0.536
2	20.058	25921635	559706	99.464
Total		26061333	563210	100.000

SI-2 (3.8 g) from the previous step was placed into a 300 mL autoclave equipped with a stirring bar. Then it was mixed with 10% Pd/C (800 mg, 0.75 mmol, 10 mol%) and sodium acetate (92 mg, 1.12 mmol, 15 mol%). Then 100 mL of anhydrous EtOAc was added, and the autoclave was sealed and purged with Ar for 15 minutes. Then, the autoclave was filled with hydrogen (20 bar) and left to stir at r.t. for 2 hours. Hydrogen was released, and the autoclave was flushed with Ar for 20 minutes. The mixture was filtered over a short celite pad. The fritted glass filter with a celite layer was extensively washed with EtOAc (200 mL). The mother liquor was dried over anhydrous Na_2SO_4, filtered, and concentrated *in vacuo* to afford the desired product (3.79

g, 99%) as mixture of two diastereomers (d.r. 9.7:1, determined via ^{1}H NMR). This mixture was purified using column chromatography (slow gradient elution 100:1 (PE:EtOAc) to 50:1 (PE:EtOAc)) to afford **8** (3.4 g, 77% over 2 steps) as colorless oil and **8^{minor}** (280 mg, 6% over 2 steps) as yellowish oil.

Experimental Notes:

- In the absence of AcONa, partial cleavage of the primary OTES-group was observed.
- More concentrated EtOAc solutions for the hydrogenation step resulted in lower yields and OTES partial deprotection.
- The relative configuration of the **8^{minor}** was determined by Nuclear Overhauser Effect Spectroscopy (NOESY):

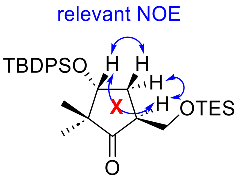

8^{minor}

Analytical data 8^{minor}:

^{1}H NMR (500 MHz, CDCl$_3$): δ 7.68 – 7.65 (m, 4H), 7.45 – 7.42 (m, 2H), 7.39 – 7.36 (m, 4H), 4.17 ("t", *J* = 4.8 Hz, 1H), 3.90 (dd, *J* = 9.7, 3.6 Hz, 1H), 3.50 (dd, *J* = 9.7, 3.2 Hz, 1H), 2.55 – 2.50 (m, 1H), 2.00 (ddd, *J* = 13.2, 7.9, 5.2 Hz, 1H), 1.88 (ddd, *J* = 13.5, 9.5, 4.5 Hz, 1H), 1.09 (s, 3H), 1.07 (s, 9H), 0.85 – 0.82 (m, 12H), 0.46 (q, *J* = 7.9 Hz, 6H). **^{13}C NMR (126 MHz, CDCl$_3$):** δ 222.1, 136.1, 136.0, 134.6, 133.9, 129.9, 129.8, 127.8, 127.7, 78.9, 61.8, 51.3, 48.1, 31.8, 27.2, 21.3, 19.6, 18.6, 6.8, 4.3.
IR (neat): ν 2957, 2876, 1742, 1463, 1428, 1383, 1107, 826, 736, 703.
HRMS (ESI): calcd for $C_{30}H_{46}O_3Si_2$ $[M+Na]^+$: 533.2878, found: 533.2878
TLC: Rf = 0.19 (40:1 PE/Et_2O, $KMnO_4$, or UV 254 nm).
$[\alpha]_D^{25}$ = -39.27 (c = 1.04, $CHCl_3$).

Analytical data 8:

^{1}H NMR (500 MHz, CDCl$_3$): δ 7.71 – 7.68 (m, 4H), 7.46 – 7.42 (m, 2H), 7.4o – 7.36 (m, 4H), 3.96 – 3.92 ("m", 1H), 3.88 (dd, *J* = 9.9, 4.1 Hz, 1H), 3.60 (dd, *J* = 10.0, 3.2 Hz, 1H), 2.08 – 1.98 (m, 2H), 1.91 – 1.84 (m, 1H), 1.09 (s, 9H), 1.01 (s, 3H), 0.94 (s, 3H), 0.93 (t, *J* = 7.9 Hz, 9H), 0.56 (q, *J* = 8.0 Hz, 6H). **^{13}C NMR (126 MHz, CDCl$_3$):** δ 220.1, 136.1, 136.1, 134.5, 133.7, 130.0, 129.8, 127.8, 127.7, 78.1, 60.7, 50.3, 49.8, 31.1, 27.1, 22.1, 19.5, 16.6, 6.9, 4.4.
IR (neat): ν 2957, 2877, 1743, 1463, 1428, 1383, 1113, 776, 741, 705.
HRMS (ESI): calcd for $C_{30}H_{46}O_3Si_2$ $[M+Na]^+$: 533.2878, found: 533.2886
TLC: Rf = 0.13 (40:1 PE/Et_2O, $KMnO_4$, or UV 254 nm).
$[\alpha]_D^{25}$ = 21.82 (c = 1.1, $CHCl_3$).

Synthesis of tert-butyl(((1R,3S,4R)-2,2-dimethyl-3-(3-methylbut-3-en-1-yl)-3-((triethylsilyl)oxy)-4-(((triethylsilyl)oxy)methyl)cyclopentyl)oxy)diphenylsilane (**13**)

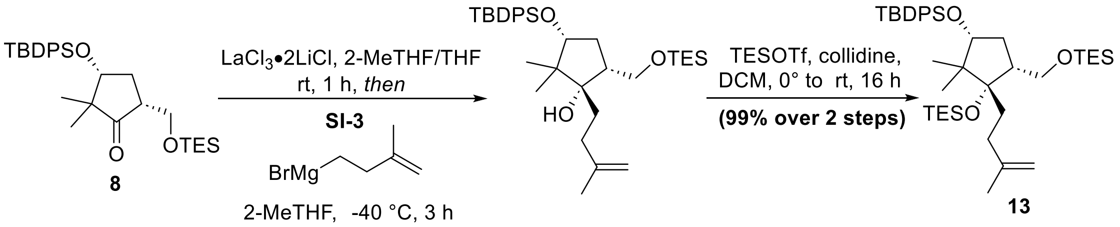

Preparation of (3-methylbut-3-en-1-yl)magnesium bromide (SI-3):

Synthesis of 4-bromo-2-methylbut-1-ene (**SI-4**):

PPh_3 (26 g, 99 mmol, 1 equiv) was placed into a 500 mL round-bottom flask equipped with a stirring bar and then dissolved in 100 mL of anhydrous THF. 3-methylbut-3-en-1-ol (10.0 mL, 98.9 mmol, 0.99 equiv) was added to the obtained solution. The reaction mixture was cooled to 0 °C, and portions of NBS (freshly recrystallized from water) (17.6 g, 99 mmol, 1 equiv.) were added. The reaction mixture was warmed to room temperature and stirred for 16 h. Then, the reaction was diluted with 200 mL of *n*-pentane. The mixture was filtered over a short silica pad. The fritted glass filter with a silica layer was extensively washed with *n*-pentane (1000 mL). The mother liquor was concentrated *in vacuo*. The residue was purified using column chromatography (elution with pentane) to afford 4-bromo-2-methylbut-1-ene (9.5 g, 64%) as a colorless oil.

^{1}H NMR (400 MHz, $CDCl_3$): δ 4.86 (s, 1H), 4.77 (s, 1H), 3.47 (t, *J* = 7.4 Hz, 2H), 2.58 (td, *J* = 7.4, 1.3 Hz, 2H), 1.75 (t, *J* = 1.2 Hz, 3H). **^{13}C NMR (101 MHz, $CDCl_3$):** δ 142.5, 112.8, 41.0, 30.9, 22.1.
Analytical data are identical to those previously reported.[6]

Magnesium turnings (632 mg, 26 mmol) were placed into a 50 mL two-necked round-bottom flask equipped with a stirring bar and a condenser. A stopcock valve was attached to the condenser, and the second neck of the flask was sealed with a rubber septum. The flask was flame-dried with a heat gun. It was cooled down, evacuated, and filled with Ar 3 times. Under the flow of Ar, iodine (25 mg, 0.1 mmol) was introduced into the flask, and the flask was heated with the heat gun until purple vapors spread throughout it. Then, it was left to stir (1000 rpm) for 15 minutes. 4-bromo-2-methylbut-1-ene (2.98 g, 20 mmol, 2.48 mL) was dissolved under Ar in 22.5 mL of the anhydrous and degassed 2-MeTHF. The obtained solution was added dropwise to the activated magnesium turnings using a syringe pump (rate 1.5 mL/min). Upon the completion of the addition, the reaction mixture was heated up to 70 °C and stirred at this temperature for 2 hours. Aliquot was titrated using Knochel's method,[7] obtained concentration of (3-methylbut-3-en-1-yl)magnesium bromide was 0.75 M (94% conversion).

Experimental Notes:

- Commercially available magnesium turnings were initially "activated" via fast treatment with 0.1 M HCl. Then, the acid was filtered off, and metallic magnesium was washed extensively with distilled water and dried on the fritted glass filter for 30 min. Then, it was

placed in the round-bottom flask and dried under vacuum (0.1 mbar) at 100 °C for 14 h. Obtained dry magnesium turnings were stored under Ar.

- (3-methylbut-3-en-1-yl)magnesium bromide was found to be poorly soluble in THF. Attempts to perform Grignard reagent synthesis under higher dilution in THF results in poor conversion. LiCl (2 eq) might be used to ensure high solubility and conversion rates during the Grignard synthesis. Application of (3-methylbut-3-en-1-yl)magnesium bromide lithium chloride complex in 1,2-addition (mediated by lanthanum chloride) generally resulted in lower yields (70-80%).
- 2-MeTHF was found to be crucial not only for the Grignard reagent synthesis but also for increasing the yield of 1,2-addition.

Preparation of 13:

A flame-dried round bottom flask (100 mL), equipped with a stirring bar, was charged with **8** (3 g, 5.87 mmol, 1 equiv) and 11 mL of the anhydrous and degassed 2-MeTHF. A commercially available 0.6 M THF solution of $LaCl_3 \cdot 2LiCl$ (10.8 mL, 6.46 mmol, 1.1 equiv) was added to this flask. The obtained reaction mixture was stirred at room temperature for one hour and then placed in a -40 °C cooling bath (temperature controlled with cryostat). Freshly prepared (3-methylbut-3-en-1-yl)magnesium bromide 0.75 M solution in 2-MeTHF (9.9 mL, 7.34 mmol) was added dropwise, using a syringe pump (rate 1 mL/min). Upon the completion of the addition, the obtained mixture was further stirred at -40 °C until the complete conversion of the starting was observed (ca 3 h, TLC control). The reaction was quenched at -40 °C with sat. aq. NH_4Cl (20 mL) was warmed to r.t. and transferred to the separatory funnel diluted with Et_2O (50 mL) and aq. HCl (20 mL, 0.5 M). The organic phase was separated, and the aqueous phase was further extracted with Et_2O (50 mL x3). Combined organic fractions were successively washed with sat. aq. $NaHCO_3$ (100 mL) and with brine (100 mL), dried over anhydrous Na_2SO_4, filtered, and concentrated *in vacuo*. The obtained residue was placed into a flame-dried round bottom flask (50 mL) equipped with a stirring bar. Anhydrous DCM (15 mL) was added to the flask, followed by *sym*-collidine (1.57 g, 12.9 mmol, 1.7 mL, 2.2 equiv). The obtained solution was cooled down to 0 °C using a water-ice bath. TESOTf (1.94 g, 7.34 mmol, 1.66 mL, 1.2 equiv) was added dropwise using a syringe pump. (rate 0.2 mL/min). The reaction mixture was left to stir for 14 hours in the ice-water bath, where it slowly warmed up to room temperature. Then it was quenched with sat. aq. $NaHCO_3$ (25 mL) and transferred to the separatory funnel. The organic phase was removed, and the aqueous was extracted with DCM (30 mL x3). The combined organic fractions were successively washed with HCl (0.5 M, 50 mL) with sat. aq. $NaHCO_3$ (100 mL), and finally, with brine (50 mL). The obtained organic fraction was dried over anhydrous Na_2SO_4, filtered, and concentrated *in vacuo*. The residue was purified using column chromatography (gradient elution 200:1 (PE:Et_2O) to 100:1 (PE:Et_2O)) to afford **13** (4.04 g, 99% over 2 steps) as a colorless oil.

*Note: The relative configuration of the alkyl chain in **13** was determined by Nuclear Overhauser Effect Spectroscopy (NOESY) after transformation into aldehyde **14**.*

^{1}H NMR (500 MHz, $CDCl_3$): δ 7.69 – 7.65 (m, 4H), 7.43 – 7.38 (m, 2H), 7.36 – 7.32 (m, 4H), 4.66 – 4.65 (m, 1H), 4.57 – 4.56 (m, 1H), 3.76 (dd, *J* = 9.9, 4.8 Hz, 1H), 3.63 (dd, *J* = 9.8, 6.8 Hz, 1H), 3.47 ("t", *J* = 9.8 Hz, 1H), 1.94 – 1.79 (m, 4H), 1.64 (brs, 3H), 1.60 – 1.55 (m, 1H),

1.53 – 1.50 (m, 1H), 1.40 (ddd, J = 13.6, 11.8, 5.1 Hz, 1H), 1.07 (s, 9H), 0.98 – 0.95 (m, 9H), 0.95 – 0.92 (m, 12H), 0.73 (s, 3H), 0.63 (qd, J = 7.9, 2.5 Hz, 6H), 0.56 (q, J = 8.1 Hz, 6H). **^{13}C NMR (126 MHz, $CDCl_3$):** δ 146.1, 136.3, 136.2, 134.9, 134.4, 129.7, 129.6, 127.6, 127.5, 109.4, 85.9, 78.6, 65.9, 50.4, 45.8, 38.3, 37.3, 32.4, 27.3, 23.0, 23.0, 19.5, 19.2, 7.6, 7.6, 6.9, 4.6.
IR (neat): ν 2956, 2878, 1462, 1428, 1239, 1108, 1079, 1008, 740, 704.
HRMS (ESI): calcd for $C_{41}H_{70}O_3Si_3$ $[M+Na]^+$: 717.4525, found: 717.4510
TLC: Rf = 0.55 (100:1 PE/Et_2O, $KMnO_4$, or UV 254 nm).
$[\alpha]_D^{25}$ = -6.1 (c = 1.0, $CHCl_3$).

Synthesis of (1S,2S,4R)-4-((tert-butyldiphenylsilyl)oxy)-3,3-dimethyl-2-(3-methylbut-3-en-1-yl)-2-((triethylsilyl)oxy)cyclopentane-1-carbaldehyde (**14**)

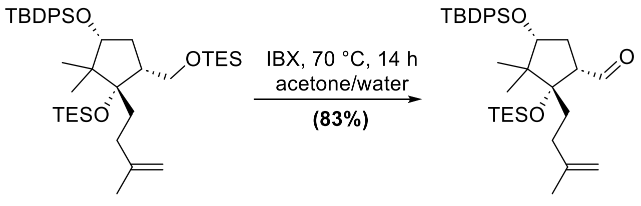

A round bottom pressure flask (50 mL) equipped with a stirring bar was charged with **13** (1 g, 1.58 mmol, 1 equiv), 15.5 mL of acetone, and distilled H_2O (0.62 mL). 2-iodoxy benzoic acid (IBX, 2.22 g, 7.91 mmol, 5.0 equiv) was added, and the flask was sealed with the screw cap and stirred at 70 °C for 14 h. Then, the solvent was removed *in vacuo*, and 50 mL of Et_2O was added to the obtained slurry. The resulting mixture was filtered over a short celite pad. The fritted glass filter was extensively washed with Et_2O (200 mL). The mother liquor was transferred to the separatory funnel and successively washed with sat. aq. $NaHCO_3$ (100 mL) and with brine (50 mL), dried over anhydrous Na_2SO_4, filtered, and concentrated *in vacuo*. The obtained residue was purified using column chromatography (gradient elution 80:1 (PE:Et_2O) to 50:1 (PE:Et_2O)) to afford **14** (760 mg, 83%) as a colorless oil.

Experimental Notes:

Initially, a condition screen was performed to find out the most suitable oxidant performing the one-pot deprotection/oxidation:

conditions	***NY, %***
DMP, DCM, H_2O	nr
PCC, DCM, 0 °C - rt	67
PCC, DCM, -15 °C to -5 °C	62
CrO_3•2Py, DCM 0 °C	78.5 (isolated)
CrO_3•2Py, DCM 0 °C	54 (850 mg)
DMP, DCM, H_2O, AcOH, 45 °C	75

Reactions were performed at 0.1 mmol scale and analyzed by ^{1}H NMR unless specified otherwise. The initial hit was observed for the chromium-containing reagents, with Collins reagent (CrO_3•2Py) being the highest-yielding one. Unfortunately, upon the scale-up, yields of the desired product decreased. When the reaction was performed on an 850 mg scale, only 54% of the desired product was observed, with clear evidence of epimerization of the obtained aldehyde at the alpha position. During the further conditions screen, we discovered that a combination of AcOH/DMP can be used to cleave and oxidize the primary OTES group. Considering the pKa of AcOH and IBX, we envisioned that the latter could be suitable for the OTES deprotection. Indeed, when treatment of **13** was performed in wet acetone at 70 °C with an excess of IBX (5 eq) on a 0.1 mmol scale, the desired aldehyde **14** was obtained in quantitative yield. The reaction was successfully scaled up afterward; however, there was a slight yield decrease.

- Freshly prepared 2-iodoxy benzoic acid (≥ 99% purity) was used.[5]
- The relative configuration of the obtained aldehyde **14** was determined by Nuclear Overhauser Effect Spectroscopy (NOESY):

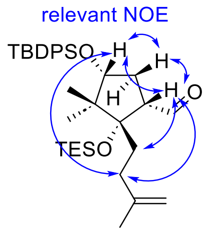

14

^{1}H NMR (500 MHz, $CDCl_3$): δ 9.66 (d, *J* = 2.8 Hz, 1H), 7.68 – 7.65 (m, 4H), 7.45 – 7.41 (m, 2H), 7.39 – 7.35 (m, 4H), 4.69 – 4.67 (m, 1H), 4.57 – 4.56 (m, 1H), 3.73 (dd, *J* = 9.8, 7.4 Hz, 1H), 2.52 (td, *J* = 8.9, 2.9 Hz, 1H), 2.15 (ddd, *J* = 13.2, 9.9, 8.6 Hz, 1H), 1.92 – 1.82 (m, 2H), 1.69 – 1.63 (m, 4H), 1.62 – 1.52 (m, 1H), 1.42 (ddd, *J* = 13.7, 11.0, 6.0 Hz, 1H), 1.09 (s, 9H), 0.98 (s, 3H), 0.94 (t, *J* = 7.9 Hz, 9H), 0.76 (s, 3H), 0.62 (qd, *J* = 7.9, 1.9 Hz, 6H). **^{13}C NMR (126 MHz, $CDCl_3$):** δ 203.1, 145.2, 136.2, 136.1, 134.4, 134.0, 129.9, 129.8, 127.7, 127.7, 109.9, 88.0, 78.0, 55.1, 50.1, 38.3, 32.3, 30.7, 27.2, 23.1, 21.4, 19.4, 18.1, 7.4, 7.4.
IR (neat): ν 2957, 2878, 1722, 1463, 1428, 1239, 1110, 1079, 1008, 741, 705.
HRMS (ESI): calcd for $C_{35}H_{54}O_3Si_2$ $[M+Na]^+$: 601.3504, found: 601.3510
TLC: Rf = 0.21 (40:1 PE/Et_2O, $KMnO_4$, or UV 254 nm).
$[\alpha]_D^{25}$ = 18.71 (c = 1.01, $CHCl_3$).

<u>Synthesis of 1-((1S,2S,4R)-4-((tert-butyldiphenylsilyl)oxy)-3,3-dimethyl-2-(3-methylbut-3-en-1-yl)-2-((triethylsilyl)oxy)cyclopentyl)-2-methylprop-2-en-1-one (7)</u>

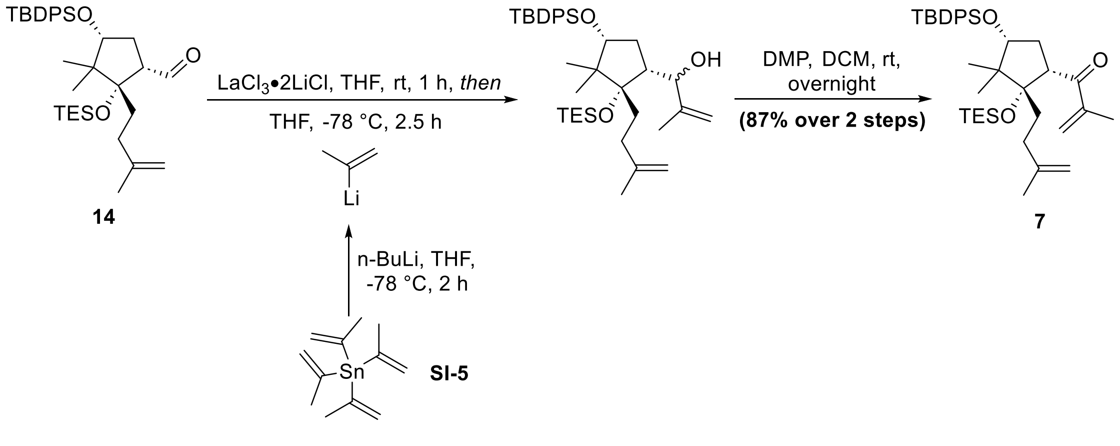

Synthesis of tetra(prop-1-en-2-yl)stannane (**SI-5**):

Magnesium turnings (2.23 mg, 91.8 mmol) were placed into a 100 mL two-necked round-bottom flask equipped with a stirring bar and a condenser. A stopcock valve was attached to the condenser, and the second neck of the flask was sealed with a rubber septum. The flask was flame-dried with a heat gun. It was cooled down, evacuated, and filled with Ar 3 times. Under the flow of Ar, iodine (25 mg, 0.1 mmol) was introduced into the flask, and the flask was heated with the heat gun until purple vapors spread throughout it. Then, it was left to stir (1000 rpm) for 15 minutes. Isopropenyl bromide (5.55 g, 45.9 mmol, 4.1 mL) was dissolved under Ar in 45 mL of the anhydrous and degassed THF. The obtained solution was added dropwise to the activated magnesium turnings using a syringe pump (rate 2 mL/min). The reaction mixture was refluxed for 90 minutes after the addition. Aliquot was titrated using Knochel's method,[7] obtained concentration of isopropenyl magnesium bromide was 0.85 M (91% conversion).

Under the atmosphere of Ar, tin tetrachloride (2.39 g, 9.2 mmol, 1.1 mL) was placed into a flame-dried 250 mL round-bottom flask equipped with a stirring bar. The flask was sealed with a rubber septum. 30 mL of anhydrous, degassed toluene was added to the flask, followed by 0.85 M THF solution of isopropenyl magnesium bromide (45 mL, 38.3 mmol) – dropwise addition, using a syringe pump (rate 2 mL/min). The obtained reaction mixture was heated up to 70 °C and stirred at this temperature for 16 h. The flask was cooled down to room temperature and quenched with aq. HCl (1 M, 100 mL) and transferred to a separatory funnel. The organic phase was separated, and the aqueous was further extracted with Et_2O (50 mL x3). Combined organic fractions were successively washed with sat. aq. $NaHCO_3$ (100 mL) and with brine (100 mL), dried over anhydrous Na_2SO_4, filtered, and concentrated *in vacuo*. The obtained residue was purified using column chromatography (elution with PE) to afford tetra(prop-1-en-2-yl)stannane (**SI-5**) (1.95 g, 75%) as a colorless oil.

^{1}H NMR (400 MHz, $CDCl_3$): δ 6.05 – 5.61 (m, 1H), 5.32 – 5.12 (m, 1H), 2.09 – 1.96 (m, 3H).
^{13}C NMR (101 MHz, $CDCl_3$): δ 147.5, 128.2, 27.1.
IR (neat): ν 2935, 291, 1837, 1449, 1435, 915.
HRMS (ESI): calcd for $C_{12}H_{20}Sn$ $[M+Ag]^+$: 390.9633, found: 390.9631
TLC: Rf = 0.9 (*n*-pentane).

Tetra(prop-1-en-2-yl)stannane (1.05 g, 3.72 mmol, 0.8 mL, 2.2 equiv) was used for the *in situ* preparation of *isopropenyl lithium*. It was placed into a flame-dried 50 mL round-bottom flask equipped with a stirring bar under an atmosphere of Ar. The flask was sealed with a rubber septum and charged with 8.5 mL of anhydrous, degassed THF. The reaction mixture was cooled down to -78 °C, and 2.5 M in hexanes *n*-BuLi (3.72 mmol, 1.5 mL, 2.2 eq) was added dropwise to the flask. It was stirred at -78 °C for 2 hours. Aldehyde **14** (980 mg, 1.69 mmol, 1 equiv) was dissolved in 5.5 mL of anhydrous, degassed THF in the flame-dried 25 mL round-bottom flask equipped with a stirring bar under an atmosphere of Ar. A commercially available 0.6 M THF solution of $LaCl_3·2LiCl$ (3.5 mL, 2.12 mmol, 1.25 equiv) was added to the aldehyde solution, and the obtained mixture was stirred for 45 minutes at room temperature. Then, it was added dropwise, using a syringe pump (rate 2 mL/min), to the flask containing *isopropenyl lithium* at -78 °C. The resulting reaction mixture was stirred for 2.5 hours at the same temperature. Then it was quenched with sat. aq. NH_4Cl (20 mL), warmed up to r.t., transferred to the separatory funnel, diluted with Et_2O (50 mL), and aq. HCl (20 mL, 1 M). The organic phase was separated, and the aqueous was further extracted with Et_2O (50 mL x3). Combined organic fractions were successively washed with sat. aq. $NaHCO_3$ (100 mL) and with brine (100 mL), dried over anhydrous Na_2SO_4, filtered, and concentrated *in vacuo*. The obtained residue was placed into a flame-dried round bottom flask (25 mL) equipped with a stirring bar. Anhydrous DCM (8.5 mL) was added to the flask. The obtained solution was cooled down to 0 °C using a water-ice bath. Commercial grade Dess-Martin periodinane (1.29 g, 3.05 mmol, 1.8 equiv) was added in one portion. The reaction mixture was left to stir for 14 hours in the ice-water bath, where it slowly warmed up to room temperature. Then it was quenched with sat. aq. $NaHCO_3$ (5 mL) and sat. aq. $Na_2S_2O_3$ (5 mL) and transferred to the separatory funnel. The organic phase was removed, and the aqueous was extracted with DCM (15 mL x3). The combined organic fractions were washed with brine (50 mL). The obtained organic fraction was dried over anhydrous Na_2SO_4, filtered, and concentrated *in vacuo*. The obtained residue was purified using column chromatography (gradient elution 100:1 (PE:Et_2O) to 80:1 (PE:Et_2O)) to afford **7** (910 mg, 87%) as a yellowish solid.

^{1}H NMR (500 MHz, $CDCl_3$): δ 7.71 – 7.65 (m, 4H), 7.45 – 7.37 (m, 2H), 7.40 – 7.33 (m, 4H), 5.69 (s, 1H), 4.66 (s, 1H), 4.54 (s, 1H), 3.66 (dd, *J* = 10.8, 7.3 Hz, 1H), 3.49 ("t", *J* = 9.2 Hz, 1H), 2.50 (ddd, *J* = 12.5, 10.8, 9.7 Hz, 1H), 1.88 (s, 3H), 1.77 – 1.71 (m, 1H), 1.71 – 1.66 (m, 2H), 1.63 (s, 3H), 1.62 – 1.57 (m, 1H), 1.52 – 1.45 (m, 1H), 1.08 (s, 9H), 1.03 (s, 3H), 0.88 (t, *J* = 7.9 Hz, 9H), 0.75 (s, 3H), 0.53 (qd, J = 7.9, 5.9 Hz, 6H). **^{13}C NMR (126 MHz, $CDCl_3$)**: δ 200.1, 146.8, 145.3, 136.3, 136.2, 134.6, 134.2, 129.8, 129.7, 127.7, 127.5, 123.8, 109.9, 87.0, 77.6, 50.2, 47.5, 39.7, 33.7, 33.6, 27.2, 22.9, 21.5, 19.4, 18.6, 18.0, 7.6, 7.5.
IR (neat): ν 2947, 1668, 1457, 1377, 1096, 1066, 734, 703.
HRMS (ESI): calcd for $C_{38}H_{58}O_3Si_2$ $[M+Na]^+$: 641.3817, found: 641.3815
TLC: Rf = 0.35 (40:1 PE/Et_2O, $KMnO_4$, or UV 254 nm).
$[\alpha]_D^{25}$ = 21.05 (c = 0.57, $CHCl_3$).
Melting point: 98 °C

Synthesis of (2R,3aS,8aS)-2-((tert-butyldiphenylsilyl)oxy)-1,1,5,6-tetramethyl-8a-((triethylsilyl)oxy)-2,3,3a,7,8,8a-hexahydroazulen-4(1H)-one (**6**)

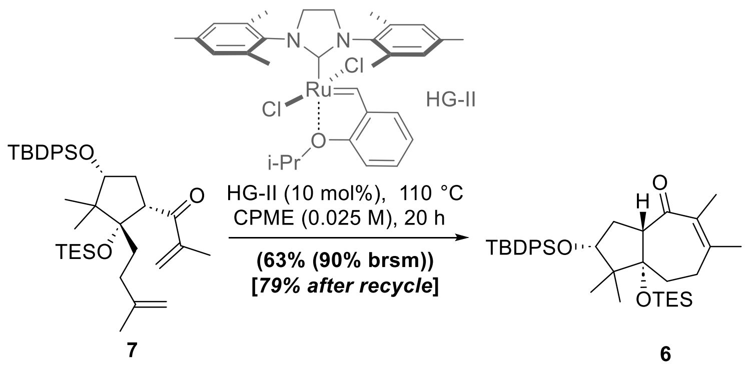

Diene **7** (130 mg, 0.21 mmol, 1 equiv) was placed into a flame-dried 25 mL round-bottom flask equipped with a stirring bar and introduced to the glove box. HG-II (13.2 mg, 0.021 mmol, 10 mol%) was added, followed by 8.5 mL of dry, degassed cyclopentyl methyl ether (CPME). The flask was sealed with the screw cap and stirred at r.t. until the catalyst was fully dissolved and then at 110 °C for 20 h. It was then cooled to room temperature and concentrated *in vacuo*. The obtained residue was purified using column chromatography (gradient elution 120:1 (PE:Et_2O) to 100:1 (PE:Et_2O) – recovered starting material was eluted, then 80:1 (PE:Et_2O) to 40:1 (PE:Et_2O). **6** (78 mg, 63%) was obtained as white solid, together with the recovered **7** (35 mg, 27%) as colorless oil.

The recovered **7** (35 mg, 0.0565 mmol, 1 equiv) was reused in the described procedure (HG II (3.6 mg, 10 mol%) and 2.3 mL of CPME) to afford 6 (20 mg, 60%) as a white solid.

Experimental Notes:

- Conditions optimization (reactions were performed at 0.05 mmol scale, and 0.025 M concentration):

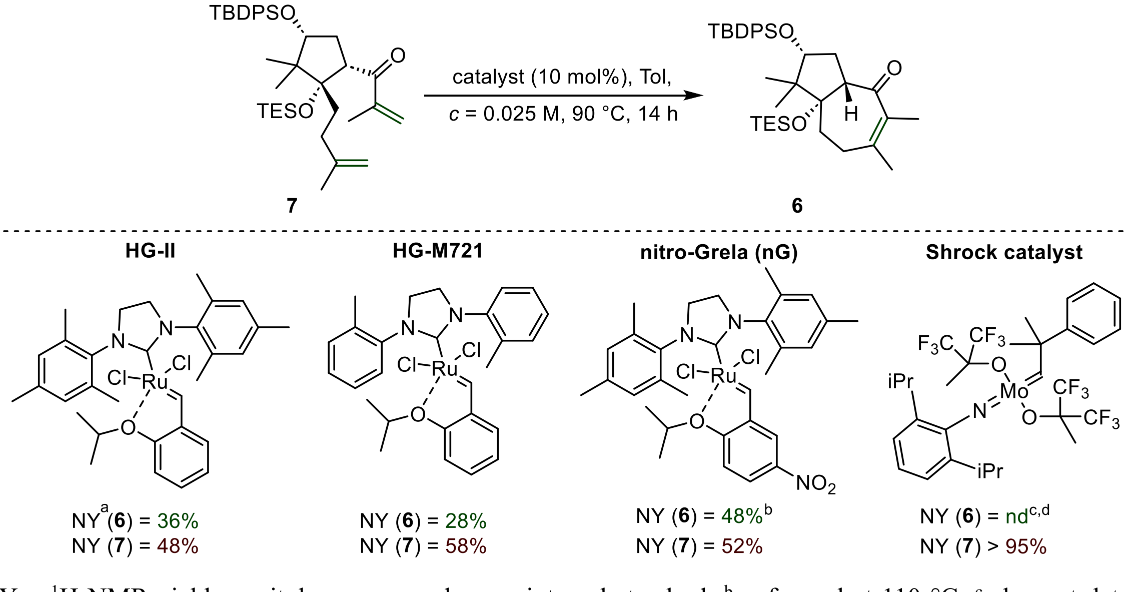

[a]NY = ^{1}H NMR yield, mesitylene was used as an internal standard; [b]performed at 110 °C; [c]nd = not detected; [d]performed at 60 °C.

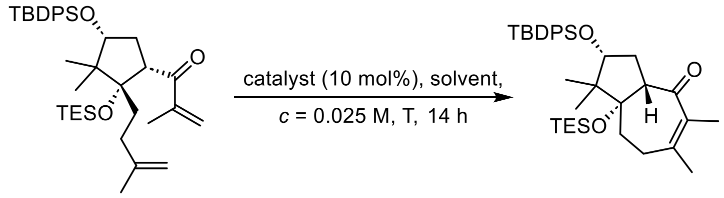

7 **6**

HG-II **nitro-Grela (nG)**

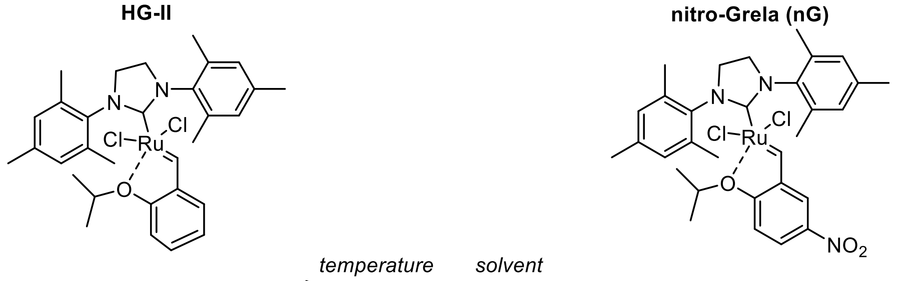

solvent		*temperature*	*solvent*		*temperature*
F_3-toluene	NY[a](**6**) = 42%; NY (**7**) = 42%	110 °C	Toluene	NY (**6**) = 48%; NY (**7**) = 52%	110 °C
CPME	NY (**6**) = 62%; NY (**7**) = 27%	110 °C	CPME	NY (**6**) = 43%; NY (**7**) = 50%	110 °C
CH_2Cl_2	*decomposition*	110 °C			
Toluene	NY (**6**) = 50%; NY (**7**) < 15%	130 °C			

[a]NY = ^{1}H NMR yield, mesitylene was used as an internal standard.

- The starting material was recovered after the first cycle as a colorless oil, whereas **7** is a white solid. ^{1}H NMR shows lower purity compared to the product obtained from **14**.
- Further recycling of starting material results in a decreased yield of **6** (generally 30-40% after the third cycle).
- Portionwise addition of the HG-II catalyst did not result in the yield improvement.

^{1}H NMR (500 MHz, CDCl$_3$): δ 7.72 – 7.67 (m, 4H), 7.43 – 7.39 (m, 2H), 7.38 – 7.34 (m, 4H), 3.80 (dd, J = 7.7, 6.6 Hz, 1H), 2.94 (dd, J = 12.4, 6.2 Hz, 1H), 2.56 (td, J = 12.7, 6.6 Hz, 1H), 2.43 ("t", J = 15.1 Hz, 1H), 2.25 (ddd, J = 17.2, 5.4, 2.2 Hz, 1H), 2.14 (ddd, J = 14.8, 5.4, 2.1 Hz, 1H), 1.97 (s, 3H), 1.95 – 1.91 (m, 1H), 1.81 (s, 3H), 1.59 – 1.55 (m, 1H), 1.09 (s, 9H), 0.93 (t, J = 7.9 Hz, 9H), 0.81 (s, 3H), 0.55 (s, 3H), 0.51 (qd, J = 7.9, 1.6 Hz, 6H). **^{13}C NMR (126 MHz, CDCl$_3$)**: δ 198.6, 153.0, 136.5, 136.4, 134.9, 134.9, 134.7, 129.6, 129.6, 127.5, 127.5, 86.7, 79.8, 56.4, 52.0, 36.5, 35.6, 34.6, 27.3, 26.8, 25.8, 19.3, 19.2, 14.5, 7.5, 7.0.
IR (neat): ν 2947, 2871, 1657, 1615, 1107, 1086, 1005, 731, 703.
HRMS (ESI): calcd for $C_{36}H_{54}O_3Si_2$ $[M+Na]^+$: 613.3504, found: 613.3512
TLC: Rf = 0.28 (20:1 PE/Et_2O, $KMnO_4$, or UV 254 nm).
$[\alpha]_D^{25}$ = -32.13 (c = 0.8, $CHCl_3$).
Melting point: 116 °C

<u>Synthesis of (2R,3aS,5S,6R,8aS)-2-((tert-butyldiphenylsilyl)oxy)-5-hydroxy-1,1,5,6-tetramethyl-8a-((triethylsilyl)oxy)octahydroazulen-4(1H)-one</u> (**5**)

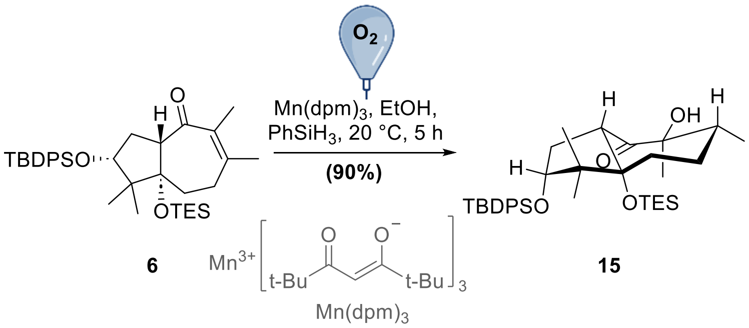

A flame-dried 25 mL round-bottom flask equipped with a stirring bar was charged with the enone **6** (57.0 mg, 0.096 mmol, 1.0 equiv), $Mn(dmp)_3$ (58.3 mg, 0.096 mmol, 1.0 equiv), and anhydrous ethanol (12 mL). $PhSiH_3$ (31.3 mg, 0.0289 mmol, 36 µL, 3.0 equiv) was dissolved in ethanol (1 mL) and added to the reaction mixture in one portion. Oxygen was bubbled through the resulting mixture for 10 minutes. Then, it was stirred under an atmosphere of oxygen (balloon) for 5 h. The reaction mixture was then concentrated *in vacuo*. The obtained residue was purified using column chromatography (gradient elution 20:1 (PE:EtOAc) to 10:1 (PE:EtOAc) to afford **15** (53 mg, 90%) as a white solid.

Note: The relative configuration of the ***15*** *was determined by Nuclear Overhauser Effect Spectroscopy (NOESY) after transformation into oxalate* ***SI-7****.*

^{1}H NMR (500 MHz, CDCl$_3$): δ 7.73 – 7.64 (m, 4H), 7.45 – 7.38 (m, 2H), 7.38 – 7.34 (m, 4H), 3.73 ("t", *J* = 7.8 Hz, 1H), 3.15 (dd, *J* = 12.8, 6.1 Hz, 1H), 2.59 (td, *J* = 12.9, 7.9 Hz, 1H), 1.93 – 1.82 (m, 1H), 1.73 (ddd, *J* = 13.4, 7.8, 6.1 Hz, 1H), 1.70 – 1.59 (m, 2H), 1.51 (s, 1H), 1.29 – 1.25 (m, 5H), 1.09 (s, 9H), 1.02 (d, *J* = 7.1 Hz, 3H), 0.99 (t, *J* = 7.9 Hz, 9H), 0.88 (s, 3H), 0.62 (qd, *J* = 7.9, 5.7 Hz, 6H), 0.53 (s, 3H). **^{13}C NMR (126 MHz, CDCl$_3$)**: δ 207.1, 136.5, 136.4, 134.6, 134.5, 129.7, 129.6, 127.6, 127.5, 90.9, 81.1, 79.1, 52.0, 50.8, 40.4, 36.1, 35.2, 28.9, 28.1, 27.3, 20.3, 19.3, 17.4, 17.0, 7.6, 7.6.
IR (neat): ν 3455, 2957, 2934, 1711, 1469, 1138, 1108, 1009, 738, 703.
HRMS (ESI): calcd for $C_{36}H_{56}O_4Si_2$ $[M+Na]^+$: 631.3609, found: 631.3608
TLC: Rf = 0.3 (9:1 PE/EtOAc, $KMnO_4$).
$[\alpha]_D^{25}$ = 6.4 (c = 0.75, $CHCl_3$).
Melting point: 158 °C

Iridium(III)-catalyzed deoxyfunctionalization

Synthesis of (2R,3aS,5S,6R,8aS)-2-((tert-butyldiphenylsilyl)oxy)-1,1,5,6-tetramethyl-4-oxo-8a-((triethylsilyl)oxy)decahydroazulen-5-yl (2-(trimethylsilyl)ethyl) oxalate **(SI-7)**

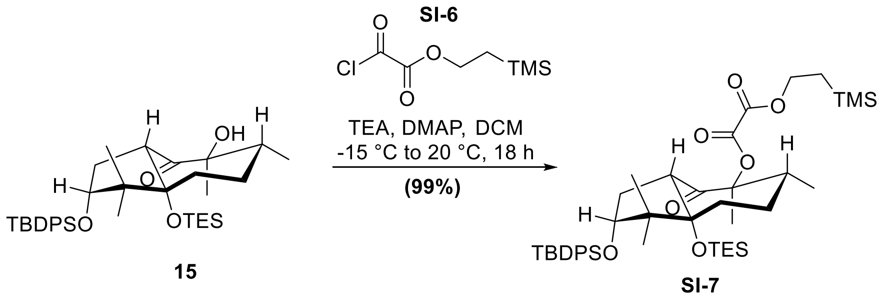

Synthesis of 2-(trimethylsilyl)ethyl 2-chloro-2-oxoacetate (**SI-6**):

To a flame-dried 25 mL round-bottom flask, equipped with the stirring bar and containing oxalyl chloride (4.95 g, 41.9 mmol, 6 mL, 1 equiv), was added 2-(trimethylsilyl)ethanol (5.31 g, 41.9 mmol, 3.98 mL, 1 equiv), dropwise, using a syringe pump (rate 0.5 mL/min) at 0 °C. The mixture was warmed to 25 °C, stirred for 14 h, concentrated *in vacuo*, and subjected to distillation to afford **SI-6** (4.0 g, 46 %) as a colorless oil.

^{1}H NMR (500 MHz, CDCl$_3$): δ 4.48 – 4.44 (m, 2H), 1.17 – 1.12 (m, 2H), 0.08 (s, 9H). **^{13}C NMR (126 MHz, CDCl$_3$):** δ 161.4, 156.0, 68.1, 17.5, -1.4.
Analytical data are identical to those previously reported.[8]

A flame-dried 10 mL round-bottom flask, equipped with a stirring bar, was charged, under an atmosphere of Ar, with the alcohol **15** (16.0 mg, 0.0263 mmol, 1.0 equiv), and anhydrous, degassed DCM (0.5 mL). The flask was then placed in a -15 °C cooling bath (temperature controlled with the cryostat). 0.5 mL of DCM solution containing triethyl amine (3.2 mg, 0.032 mmol, 4.4 µL, 1.2 equiv) and DMAP (0.32 mg, 0.00026 mmol, 10 mol%) was slowly added to the precooled flask. To the obtained reaction mixture, 1 mL of DCM solution containing 2-(trimethylsilyl)ethyl 2-chloro-2-oxoacetate (**SI-6**) (6.6 mg, 0.0315 mmol, 1.2 equiv) was added dropwise, using a syringe pump (rate 0.2 mL/min). The cryostat was turned off, and the reaction mixture was left to stir in the cooling bath for 18 h, where it slowly reached room temperature. Then it was quenched with sat. aq. $NaHCO_3$ (10 mL) and transferred to the separatory funnel. The organic phase was removed, and the aqueous was extracted with DCM (10 mL x3). The combined organic fractions were successively washed with HCl (0.5 M, 10 mL) with sat. aq. $NaHCO_3$ (20 mL), and finally with brine (20 mL). The obtained organic fraction was dried over anhydrous Na_2SO_4, filtered, and concentrated *in vacuo*. The residue was purified using column chromatography (gradient elution 80:1 (PE:EtOAc) to 50:1 (PE:EtOAc) to afford **SI-7** (20.4 mg, 99%) as a colorless oil.

Experimental Notes:

- **SI-7** was stored in the fridge under an Ar atmosphere after distillation.
- Scaling up the acylation results in a decreased yield; for instance, a 42 mg, 0.069 mmol scale of **15** results in 49 mg (91%) of **SI-7**.
- The relative configuration of the obtained aldehyde **14** was unambiguously determined by Nuclear Overhauser Effect Spectroscopy (NOESY):

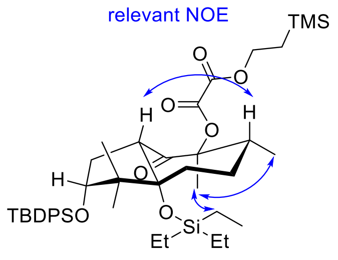

SI-7

^{1}H NMR (500 MHz, CDCl$_3$): δ 7.72 – 7.69 (m, 2H), 7.67 – 7.64 (m, 2H), 7.43 – 7.39 (m, 2H), 7.39 – 7.35 (m, 4H), 4.33 – 4.30 ("m", 2H), 3.70 ("t", J = 7.7 Hz, 1H), 3.34 (dd, J = 12.8, 6.1 Hz, 1H), 2.56 (td, J = 12.9, 7.6 Hz, 1H), 2.42 ("p", J = 7.3 Hz, 1H), 1.91 (ddd, J = 13.0, 7.8, 6.1 Hz, 1H), 1.69 – 1.62 (m, 2H), 1.53 – 1.45 (m, 4H), 1.43 – 1.38 (m, 1H), 1.08 (s, 9H), 1.08 – 1.04 (m, 5H), 0.99 (t, J = 7.9 Hz, 9H), 0.86 (s, 3H), 0.67 (qd, J = 7.8, 5.6 Hz, 6H), 0.47 (s, 3H), 0.04 (s, 9H). **^{13}C NMR (126 MHz, CDCl$_3$)**: δ 203.5, 157.9, 156.5, 136.5, 136.4, 134.6, 134.3, 129.7, 129.6, 127.6, 127.6, 90.8, 90.0, 80.6, 65.8, 54.4, 51.1, 38.5, 35.8, 35.4, 28.8, 28.0, 27.3, 20.3, 19.3, 17.6, 17.3, 16.4, 7.7, 7.6, -1.4.
IR (neat): ν 2955, 2934, 1766, 1742, 1710, 1468, 1250, 1169, 1110, 1086, 1008, 839, 737, 703.
HRMS (ESI): calcd for $C_{43}H_{68}O_7Si_3$ $[M+Na]^+$: 803.4165, found: 803.4171
TLC: Rf = 0.25 (20:1 PE/Et_2O, $KMnO_4$).
$[\alpha]_D^{25}$ = 3.29 (c = 0.7, $CHCl_3$).

<u>Synthesis of 4-vinylfuran-2(5H)-one </u>(**4**)

Modification of procedure by Pale *et al.*:[9]

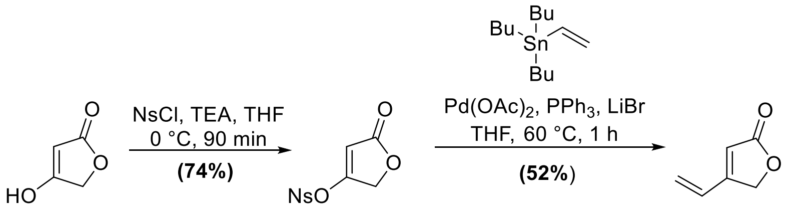

Synthesis of 5-oxo-2,5-dihydrofuran-3-yl 4-nitrobenzenesulfonate (**SI-8**):

To the flame-dried 100 mL round-bottom flask, equipped with the stirring bar and containing tetronic acid (1 g, 10 mmol, 1 equiv) in THF (20 mL) was added triethylamine (1.32 g, 13 mmol, 1.8 mL, 1.3 eq.) dropwise at 0°C under argon. After 5 min of stirring, a solution of 4-nitrobenzenesulfonyl chloride (2.44 g, 11 mmol, 1.1 eq.) in THF (12.5 mL) was added dropwise, using a syringe pump (rate 1 mL/min). The reaction mixture was stirred at 0°C for 90 min. EtOH (38 mL) was added, and the mixture was allowed to stand in the fridge at 5 °C for 16 h. Then, the mixture was filtered off, and the fritted glass filter with the product was washed with cold EtOH (15 mL) and dried to afford **SI-8** (2.1 g, 74%) as a white solid.

^{1}H NMR (400 MHz, $CDCl_3$): δ 8.52 – 8.49 (m, 2H), 8.24 – 8.21 (m, 2H), 5.91 (t, *J* = 1.6 Hz, 1H), 4.73 (d, *J* = 1.7 Hz, 2H) **^{13}C NMR (101 MHz, $CDCl_3$)**: δ 170.2, 167.4, 139.5, 130.1, 125.2, 101.8, 67.9.
Analytical data are identical to those previously reported.[10]

A flame-dried 100 mL round-bottom flask, equipped with a stirring bar containing **SI-8** (800 mg, 2.8 mmol, 1 equiv), was introduced into the glove box. $Pd(OAc)_2$ (31.5 mg, 0.14 mmol, 5 mol%), PPh_3 (73.6 mg, 0.28 mmol, 10 mol%), and LiBr (731 mg, 8.4 mmol, 3 equiv) were added. The flask was sealed with the rubber septum and removed from the glove box. 48 mL of anhydrous, degassed THF were added to the corresponding flask under Ar, followed by tributyl(vinyl)tin (934 mg, 2.94 mmol, 0.865 mL, 1.05 equiv). The reaction mixture was stirred at 60 °C for one hour, cooled down, and volatiles were evaporated *in vacuo*. The obtained residue was treated with Et_2O (60 mL) and aq. CsF (2 M, 2 equiv), and further stirred for 30 min. Then, it was filtered over a short celite pad. The fritted glass filter was extensively washed with Et_2O (100 mL). The mother liquor was concentrated *in vacuo,* and the obtained residue was purified using column chromatography (gradient elution 10:1 (PE:EtOAc) to 5:1 (PE:EtOAc) to afford **4** (160 mg, 52%) as a yellowish oil.

^{1}H NMR (500 MHz, $CDCl_3$): δ 6.70 (ddq, *J* = 17.8, 10.8, 0.7 Hz, 1H), 5.98 (tq, *J* = 1.8, 0.6 Hz, 1H), 5.63 (d, *J* = 3.9 Hz, 1H), 5.60 (d, *J* = 3.0 Hz, 1H), 4.99 (dd, *J* = 1.8, 0.8 Hz, 2H) **^{13}C NMR (126 MHz, $CDCl_3$)**: δ 173.8, 162.0, 128.1, 123.2, 116.7, 70.5.
Analytical data are identical to those previously reported.[9]

Giese-coupling of **SI-7** and **4**:

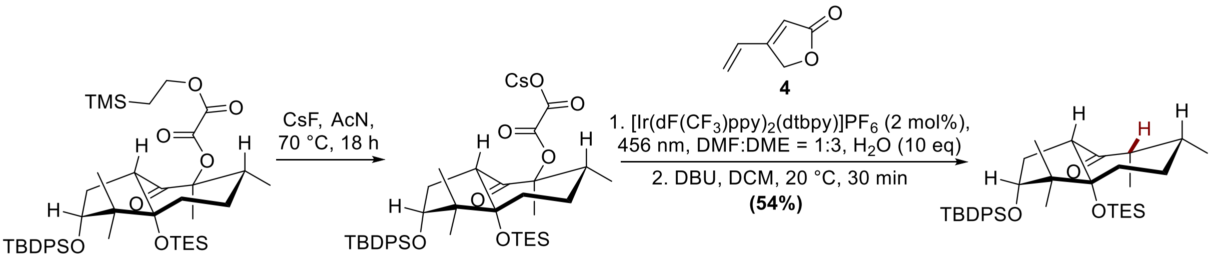

Synthesis of cesium 2-(((2R,3aS,5S,6R,8aS)-2-((tert-butyldiphenylsilyl)oxy)-1,1,5,6-tetramethyl-4-oxo-8a-((triethylsilyl)oxy)decahydroazulen-5-yl)oxy)-2-oxoacetate (**SI-9**):

The procedure for the preparation of **SI-9** is adapted from Overman *et al.*:[11]

A 10 mL flame-dried vial was charged with **SI-7** (16 mg, 0.0205 mmol, 1.0 equiv) and a stirring bar. AcN (1.0 mL) was added, followed by cesium fluoride (3.1 mg, 0.0205 mmol, 1.0 equiv). The vial was sealed with a screw cap and stirred at 70 °C for 18 h. The reaction mixture was cooled down, and volatiles were evaporated *in vacuo*. Analysis with ^{1}H NMR spectroscopy of the obtained residue showed complete conversion of starting material. Formation of carboxylate was further confirmed by negative HRMS (ESI): calcd for anion $C_{38}H_{55}O_7Si_2$ $[M]^-$: 679.3494, found: 679.3494. The obtained residue was engaged in the following step without purification.

Giese-coupling procedure is adapted from Overman, MacMillan *et al.*[12]

A 2 mL vial equipped with a stirring bar was charged with SI-9 (14 mg, 0.0172 mmol, 1.1 equiv) and transferred to the glove box. $Ir[dF(CF_3)ppy)]_2(dtbbpy)PF_6$ (0.176 mg, 0.157 µmol, 1 mol%) was added as a solution in 0.1 mL of degassed, freshly distilled DMF. Additionally, 0.3 mL of degassed, freshly distilled DME was added, followed by **4** (1.72 mg, 0.0157, 1 equiv). The vial was sealed with a rubber septum and removed from the glove box. Deionized water (2.8 mg, 0.157 mmol, 2.8 µL, 10 equiv) was added under Ar. The reaction mixture was irradiated (2 x 40 W blue LED lamps, 456 nm) for 24 h. The temperature has risen to 40 °C during the reaction (cooling with a fan). The reaction mixture was diluted with brine (10 mL) and extracted with Et_2O (3 x 5 mL). The combined organic fractions were dried over Na_2SO_4 and concentrated *in vacuo*. The residue was redissolved in DCM (3.4 mL, 0.005 M), and DBU (2.86 mg, 0.0188 mmol, 2.8 µL, 1.2 equiv) was added. The mixture was stirred at room temperature for 20 min. The reaction was diluted with DCM (5 mL) and washed with aq. HCl (0.5 M, 2x10 mL), then with sat. aq. $NaHCO_3$ (20 mL), and finally with brine (20 mL). The organic fraction was dried over anhydrous Na_2SO_4, filtered, and concentrated *in vacuo*. The residue was purified using column chromatography (gradient elution 100:1 (PE:EtOAc) to 80:1 (PE:EtOAc) to afford **17** (5.5 mg, 54%) as a white solid. The desired Giese-coupling product was not isolated. Only traces were found using TLC-MS evaluation of the thin-layer chromatography plate of the reaction mixture.

Experimental Notes:

- The relative configuration of the obtained ketone **17** was determined by Nuclear Overhauser Effect Spectroscopy (NOESY):

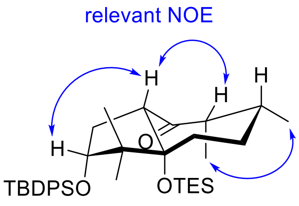

17

- *Conditions screen*:

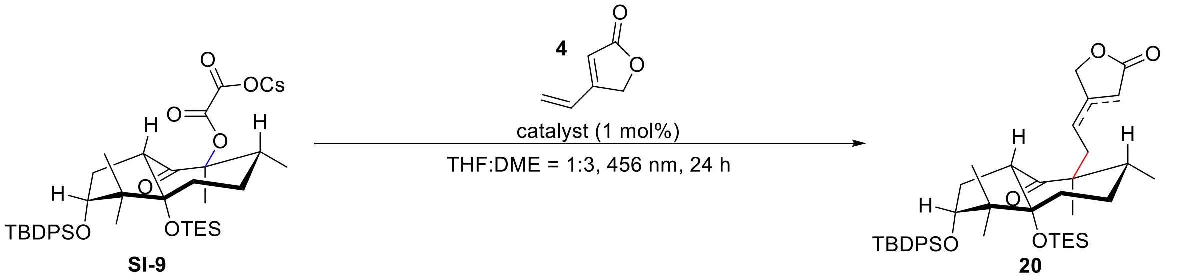

SI-9 **20**

Ir[dF(CF₃)ppy]₂(dtbpy))PF₆	Ir[dFCF₃ppy]₂(bpy)PF₆	Ir[(dtbbpy)(ppy)₂]PF₆	Ir(ppy)₃
P = trace	P = trace	P = trace	P = trace

Only a trace amount of the desired product was observed in ^{1}H NMR (mesitylene was used as an internal standard). The main product, in all cases, was ketone **17.**

Analytical data 17:

^{1}H NMR (500 MHz, CDCl₃): δ 7.73 – 7.65 (m, 4H), 7.44 – 7.38 (m, 2H), 7.38 – 7.32 (m, 4H), 3.68 ("t", *J* = 7.8 Hz, 1H), 2.97 (dd, *J* = 12.8, 6.1 Hz, 1H), 2.61 (td, *J* = 12.9, 7.9 Hz, 1H), 2.29 – 2.15 (m, 2H), 1.73 – 1.59 (m, 3H), 1.49 – 1.39 (m, 1H), 1.19 – 1.11 (m, 1H), 1.08 (s, 9H), 1.04 (d, *J* = 6.7 Hz, 3H), 1.02 – 0.94 (m, 12H), 0.86 (s, 3H), 0.63 (qd, *J* = 7.9, 3.5 Hz, 6H), 0.52 (s, 3H). **^{13}C NMR (126 MHz, CDCl₃)**: δ 209.7, 136.5, 136.4, 134.7, 134.6, 129.7, 129.6, 127.6, 127.5, 89.4, 81.0, 55.1, 50.9, 50.8, 36.5, 35.2, 34.3, 29.0, 27.3, 27.2, 21.6, 20.2, 19.3, 8.8, 7.7, 7.6.
IR (neat): ν 2957, 2927, 1704, 1466, 1111, 1075, 739, 704
HRMS (ESI): calcd for $C_{36}H_{56}O_3Si_2$ $[M+Na]^+$: 615.3660, found: 615.3666
TLC: Rf = 0.39 (20:1 PE/Et_2O, $KMnO_4$).
[α]$_D^{25}$ = 6.50 (c = 0.2, $CHCl_3$).
Melting point: 137 °C

<u>Synthesis of (2R,3aS,5S,6R,8aS)-2-((tert-butyldiphenylsilyl)oxy)-1,1,5,6-tetramethyl-4-oxo-8a-((triethylsilyl)oxy)decahydroazulen-5-yl methyl oxalate (**18**)</u>

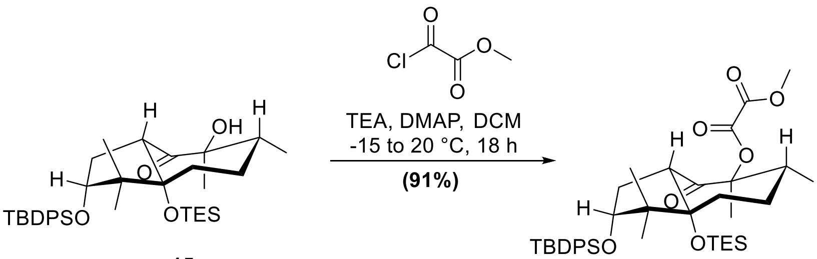

15 **18**

A flame-dried 10 mL round-bottom flask, equipped with a stirring bar, was charged, under an atmosphere of Ar, with the alcohol **15** (47.0 mg, 0.077 mmol, 1.0 equiv), and anhydrous,

degassed DCM (2 mL). The flask was then placed in a -15 °C cooling bath (temperature controlled with the cryostat). 1 mL of DCM solution containing triethyl amine (11.7 mg, 0.116 mmol, 16.3 µL, 1.5 equiv) and DMAP (0.94 mg, 0.0077 mmol, 10 mol%) was slowly added to the precooled flask. To the obtained reaction mixture, 1 mL of DCM solution containing monomethyl oxalyl chloride (11.8 mg, 0.0965 mmol, 8.9 µL, 1.25 eq) was added dropwise, using a syringe pump (rate 0.2 mL/min). The cryostat was turned off, and the reaction mixture was left to stir in the cooling bath for 18 h, where it slowly reached room temperature. Then it was quenched with sat. aq. $NaHCO_3$ (10 mL) and transferred to the separatory funnel. The organic phase was removed, and the aqueous was extracted with DCM (10 mL x3). The combined organic fractions were successively washed with HCl (0.5 M, 10 mL) with sat. aq. $NaHCO_3$ (20 mL), and finally with brine (20 mL). The obtained organic fraction was dried over anhydrous Na_2SO_4, filtered, and concentrated *in vacuo*. The residue was purified using column chromatography (gradient elution 40:1 (PE:EtOAc) to 20:1 (PE:EtOAc) to afford **18** (49 mg, 91%) as a colorless oil.

^{1}H NMR (500 MHz, $CDCl_3$): δ 7.73 – 7.62 (m, 4H), 7.44 – 7.39 (m, 2H), 7.39 – 7.33 (m, 4H), 3.83 (s, 3H), 3.70 ("t", *J* = 7.7 Hz, 1H), 3.33 (dd, *J* = 12.8, 6.1 Hz, 1H), 2.56 (td, *J* = 12.9, 7.7 Hz, 1H), 2.42 ("p", *J* = 7.3 Hz, 1H), 1.90 (ddd, *J* = 12.9, 7.8, 6.1 Hz, 1H), 1.71 – 1.59 (m, 2H), 1.48 (s, 3H), 1.43 – 1.38 (m, 2H), 1.08 (s, 9H), 1.05 (d, *J* = 7.0 Hz, 3H), 0.99 (t, *J* = 7.9 Hz, 9H), 0.86 (s, 3H), 0.67 (qd, *J* = 7.9, 5.8 Hz, 6H), 0.47 (s, 3H).
^{13}C NMR (126 MHz, $CDCl_3$): δ 203.4, 158.1, 156.0, 136.5, 136.4, 134.5, 134.3, 129.7, 129.6, 127.6, 127.6, 91.0, 90.0, 80.6, 54.4, 53.7, 51.1, 38.5, 35.9, 35.4, 28.8, 27.9, 27.3, 20.3, 19.3, 17.6, 16.4, 7.7, 7.6.
IR (neat): ν 2956, 2950, 1770, 1746, 1709, 1468, 1326, 1207, 1109, 1086, 1008, 737, 703.
HRMS (ESI): calcd for $C_{39}H_{58}O_7Si_2$ $[M+Na]^+$: 717.3613, found: 717.3621
TLC: Rf = 0.36 (9:1 PE/EtOAc, $KMnO_4$).
$[\alpha]_D^{25}$ = 5.23 (c = 0.46, $CHCl_3$).

Synthesis of (2R,3aS,5S,6R,8aS)-5-allyl-2-((tert-butyldiphenylsilyl)oxy)-1,1,5,6-tetramethyl-8a-((triethylsilyl)oxy)octahydroazulen-4(1H)-one (**19**)

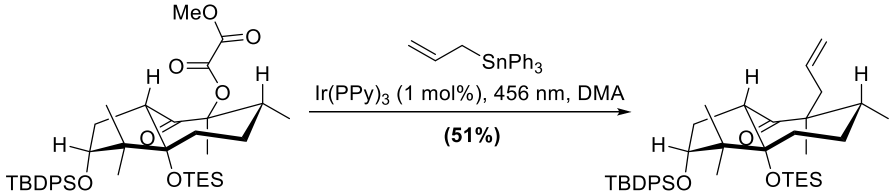

18 **19**

A 2 mL vial equipped with a stirring bar was charged with **18** (14 mg, 0.0201 mmol, 1 equiv) and transferred to the glove box. Allyltriphenylstannane (39.4 mg, 0.1 mmol, 5 equiv) was added, followed by Ir(ppy)$_3$ (0.132 mg, 0.2 µmol, 1 mol%) as a solution in 0.2 mL of degassed, anhydrous DMA. The vial was sealed with a screw cap and removed from the glove box. The reaction mixture was irradiated (2 x 40 W blue LED lamps, 456 nm) for 18 h. The temperature has risen to 45 °C during the reaction. The reaction mixture was transferred to the separatory funnel and diluted with sat. aq. NH_4Cl (5 mL), and extracted with Et_2O (4x5 mL). The combined organic fractions were washed two times with brine, dried over Na_2SO_4, and concentrated *in vacuo*. The residue was subjected to column chromatography (gradient elution 200:1:1

(PE:Et_2O:TEA) to 200:2:1 (PE:Et_2O:TEA)) to deliver the desired product **19** (6.5 mg, 51%) as a colorless oil.

Experimental Notes:

- Using 5 equiv of allyltributylstannane or 3 equiv of allyltriphenylstannane resulted in a decreased yield (0.02 mmol scale, 47%, or 35-40% respectively)
- Excess of allyltriphenylstannane can be regenerated via column chromatography during the gradient elution, and an analytical sample suitable for further deoxygenative functionalization can be obtained via sequential recrystallization from ethanol.
- The relative configuration of the obtained ketone **19** was determined by Nuclear Overhauser Effect Spectroscopy (NOESY):

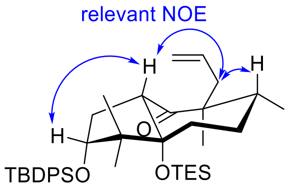

19

^{1}H NMR (500 MHz, $CDCl_3$): δ 7.73 – 7.69 (m, 2H), 7.69 – 7.66 (m, 2H), 7.44 – 7.39 (m, 2H), 7.38 – 7.34 (m, 4H), 5.48 (ddt, *J* = 17.5, 10.1, 7.5 Hz, 1H), 4.91 (dd, *J* = 10.1, 2.2 Hz, 1H), 4.87 – 4.79 (m, 1H), 3.68 ("t", *J* = 7.8 Hz, 1H), 2.90 (dd, *J* = 13.0, 6.0 Hz, 1H), 2.60 (td, *J* = 12.9, 7.8 Hz, 1H), 2.11 (dd, *J* = 13.9, 7.7 Hz, 1H), 1.98 (dd, *J* = 13.9, 7.4 Hz, 1H), 1.79 ("t", *J* = 7.2 Hz, 1H), 1.68 – 1.58 (m, 3H), 1.48 – 1.38 (m, 1H), 1.22 – 1.14 (m, 1H), 1.08 (s, 9H), 0.99 (t, *J* = 7.9 Hz, 9H), 0.94 (s, 3H), 0.93 (d, *J* = 7.0 Hz, 3H), 0.84 (s, 3H), 0.68 – 0.57 (m, 6H), 0.49 (s, 3H).**^{13}C NMR (126 MHz, $CDCl_3$)**: δ 210.5, 136.5, 136.4, 134.7, 134.6, 134.0, 129.7, 129.6, 127.6, 127.5, 117.6, 90.0, 81.2, 54.9, 53.4, 50.6, 44.1, 39.3, 36.4, 35.1, 29.0, 28.9, 27.3, 20.3, 19.3, 18.4, 15.3, 7.7, 7.5.
IR (neat): ν 2957, 2877, 1703, 1468, 1428, 1110, 1083, 1010, 737, 703.
HRMS (ESI): calcd for $C_{39}H_{60}O_3Si_2$ $[M+Na]^+$: 655.3973, found: 655.3978
TLC: Rf = 0.35 (100:1 PE/Et_2O, $KMnO_4$).
$[\alpha]_D^{25}$ = 34.47 (c = 0.47, $CHCl_3$).

<u>Synthesis of 4-(triphenylstannyl)-4-vinyldihydrofuran-2(3H)-one (**20**)</u>

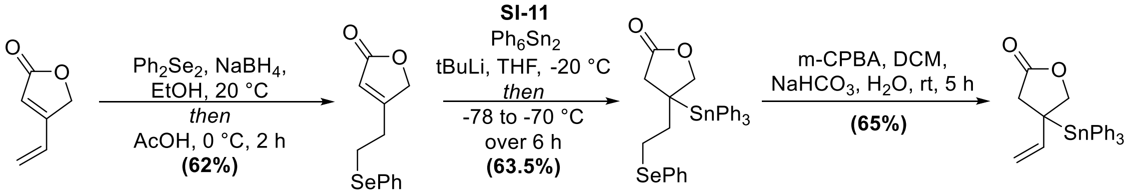

4 **SI-10** **SI-12** **20**

<u>Synthesis of 4-(2-(phenylselanyl)ethyl)furan-2(5H)-one (**SI-10**):</u>

The procedure for the preparation of **SI-10** is adapted from Miyashita *et al.*:[13]

A flame-dried 25 mL round-bottom flask, equipped with a stirring bar, was charged, under an atmosphere of Ar, with the diphenyl diselenide (480 mg, 1.53 mmol, 0.65 equiv) and absolute ethanol (6.5 mL). Sodium borohydride (116 mg, 3.07 mmol, 1.3 equiv) was added portion-wise at r.t. The obtained solution was stirred at r.t. for 30 mins (until discolored) under an Ar atmosphere. The obtained reaction mixture was cooled to 0 °C using an ice-water bath, and glacial acetic acid (326 mg, 5.43 mmol, 0.31 mL, 2.3 equiv) was slowly added. **4** (260 mg, 2.36 mmol, 1 equiv) was dissolved in absolute ethanol (1.5 mL) and added to the reaction flask using a syringe pump (rate 0.2 mL/min). The obtained reaction mixture was stirred for an additional 2 h at 0 °C. Then it was quenched with sat. aq. NH_4Cl (30 mL), diluted with Et_2O (30 mL) and transferred to the separatory funnel. The organic phase was removed, and the aqueous was extracted with Et_2O (15 mL x3). The combined organic fractions were successively washed with sat. aq. $NaHCO_3$ (30 mL), and with brine (30 mL). The obtained organic fraction was dried over anhydrous Na_2SO_4, filtered, and concentrated *in vacuo*. The residue was purified using column chromatography (gradient elution 10:1 (PE:EtOAc) to 4:1 (PE:EtOAc) to afford **SI-10** (390 mg, 62%) as a pale yellow oil.

^{1}H NMR (500 MHz, $CDCl_3$): δ 7.54 – 7.49 (m, 2H), 7.31 – 7.29 (m, 3H), 5.89 ("p", *J* = 1.7 Hz, 1H), 4.73 ("dt", *J* = 1.8, 0.7 Hz, 2H), 3.07 (t, *J* = 7.4 Hz, 2H), 2.80 – 2.77 (m, 2H). **^{13}C NMR (126 MHz, $CDCl_3$)**: δ 173.7, 168.5, 133.7, 129.5, 128.7, 127.9, 116.6, 72.9, 29.5, 24.0.
IR (neat): ν 2928, 1779, 1747, 1637, 1478, 1438, 1171, 1028, 889, 739, 692.
HRMS (ESI): calcd for $C_{12}H_{12}O_2Se$ $[M+Na]^+$: 290.9895, found: 290.9900
TLC: Rf = 0.16 (4:1 PE/EtOAc, $KMnO_4$, or UV 254 nm).

Synthesis of 4-(2-(phenylselanyl)ethyl)-4-(triphenylstannyl)dihydrofuran-2(3H)-one (**SI-12**)

The procedure for the preparation of **SI-11** is adapted from Tagliavini *et al.*:[14]

A flame-dried 100 mL round-bottom flask, equipped with a stirring bar, was charged under an atmosphere of Ar, with zinc (0.68 g, 10.4 mmol, 2.01 equiv), sat. aq. NH_4Cl (20 mL), and peroxide-free THF (20 mL). Triphenyltin chloride (2 g, 5.19 mmol, 1 equiv) was added portion-wise. The obtained reaction mixture was vigorously stirred 14 h at r.t.. Volatiles were removed *in vacuo*; the remaining slurry was diluted with Et2O (20 mL) and transferred to the separatory funnel. The organic phase was removed, and the aqueous was extracted with Et_2O (15 mL x3). The combined organic fractions were washed with brine (20 mL). The obtained organic fraction was dried over anhydrous Na_2SO_4, filtered, and concentrated *in vacuo*. The residue was purified using column chromatography (gradient elution 200:1:1 (PE:EtOAc:TEA) to 200:2:1 (PE:EtOAc:TEA) to afford **SI-11** (920 mg, 51%) as a white solid.

^{1}H NMR (500 MHz, C_6D_6): δ 7.73 – 7.61 (m, 2H), 7.13 – 7.05 (m, 3H). **^{13}C NMR (126 MHz, C_6D_6):** δ 139.5, 137.9, 129.2.
Analytical data are in accordance with those previously reported.[15]

A flame-dried 25 mL round-bottom flask, equipped with a stirring bar, was charged, under an atmosphere of Ar, with **SI-11** (1.02 g, 1.46 mmol, 1.3 equiv) and anhydrous, degassed THF (5 mL). The flask was placed in a -20 °C cooling bath (temperature controlled with the cryostat). 1.7 M pentane solution of *t*-BuLi (0.83 mL, 1.4 mmol, 1.2 equiv) was added dropwise, and the resulting solution was stirred at -20 °C for 40 min. Then, the reaction mixture was cooled to -78 °C (temperature controlled with the cryostat), and 5 mL of THF solution containing **SI-10** (300 mg, 1.12 mmol, 1 equiv) was added dropwise using a syringe pump (rate 0.2 mL/min). The cryostat temperature was set to -70 °C, and the reaction mixture was stirred for 6 hours. Then it was quenched with sat. aq. NH_4Cl (10 mL) and aq. HCl (0.5 M, 10 mL), diluted with Et_2O (30 mL) and transferred to the separatory funnel. The organic phase was removed, and the

aqueous was extracted with Et_2O (15 mL x3). The combined organic fractions were successively washed with sat. aq. $NaHCO_3$ (30 mL), and with brine (30 mL). The obtained organic fraction was dried over anhydrous Na_2SO_4, filtered, and concentrated *in vacuo*. The residue was purified using column chromatography (gradient elution 30:1 (PE:EtOAc) to 15:1 (PE:EtOAc) to afford **SI-12** (441 mg, 63.5%) as a yellow oil.

^{1}H NMR (500 MHz, $CDCl_3$): δ 7.52 – 7.38 (m, 17H), 7.26 – 7.22 (m, 3H), 7.20 – 7.16 (m, 2H), 4.69 – 4.59 (m, 1H), 4.48 – 4.40 (m, 1H), 2.90 – 2.78 (m, 4H), 2.58 – 2.50 (m, 1H), 2.30 – 2.14 (m, 2H).**^{13}C NMR (126 MHz, $CDCl_3$)**: δ 176.8, 137.2, 136.0, 133.1, 129.9, 129.3, 129.3, 129.2, 127.4, 77.2, 39.7, 39.0, 38.6, 24.4.
IR (neat): ν 3065, 3048, 1773, 1578, 1478, 1428, 1161, 1073, 996, 729, 698.
HRMS (ESI): calcd for $C_{30}H_{28}O_2SeSn$ $[M+Na]^+$: 641.0164, found: 641.0161
TLC: Rf = 0.21 (9:1 PE/EtOAc, $KMnO_4$, or UV 254 nm).

A flame-dried 25 mL round-bottom flask, equipped with a stirring bar, was charged with **SI-12** (330 mg, 0.534 mmol, 1 equiv), $NaHCO_3$ (224 mg, 2.67 mmol, 5 equiv), DCM (4.4 mL) and deionized water (0.44 mL). *m*-CPBA (92 mg, 0.534 mmol, 1 equiv) was added to the vigorously stirred reaction mixture in one portion. After 6 h, the reaction mixture was quenched with sat. aq. $Na_2S_2O_3$ (20 mL) and transferred to the separatory funnel. The organic phase was removed, and the aqueous was extracted with Et_2O (10 mL x3). The combined organic fractions were successively washed with sat. aq. $NaHCO_3$ (20 mL), and with brine (20 mL). The obtained organic fraction was dried over anhydrous Na_2SO_4, filtered, and concentrated *in vacuo*. The residue was purified using column chromatography (gradient elution 30:1 (PE:EtOAc) to 15:1 (PE:EtOAc) to afford **20** (160 mg, 65%) as a yellowish solid.

Experimental Notes:

- Commercially available *m*-CPBA was washed with a phosphate buffer (pH 7.5) and dried under reduced pressure, resulting in high reagent purity (>99%). It can be stored in a plastic bottle at -30 °C for several months.

Analytical data 20:
^{1}H NMR (500 MHz, $CDCl_3$): δ 7.58 – 7.47 (m, 6H), 7.46 – 7.41 (m, 9H), 6.12 (dd, *J* = 17.3, 10.6 Hz, 1H), 5.08 (d, *J* = 10.6 Hz, 1H), 4.97 (d, *J* = 17.3 Hz, 1H), 4.70 – 4.56 (m, 2H), 2.84 (s, 2H).**^{13}C NMR (126 MHz, $CDCl_3$)**: δ 176.3, 140.8, 137.4, 135.8, 130.0, 129.2, 112.7, 76.3, 41.0, 37.4.
IR (neat): ν 3065, 1780, 1760, 1625, 1481, 1427, 1169, 1074, 1019, 984, 730, 700.
HRMS (ESI): calcd for $C_{24}H_{22}O_2Sn$ $[M+Na]^+$: 485.0534, found: 485.0537
TLC: Rf = 0.33 (9:1 PE/EtOAc, $KMnO_4$, or UV 254 nm).

Synthesis of 4-(2-((2R,3aS,5S,6R,8aS)-2-((tert-butyldiphenylsilyl)oxy)-1,1,5,6-tetramethyl-4-oxo-8a-((triethylsilyl)oxy)decahydroazulen-5-yl)ethyl)furan-2(5H)-one **(16)**

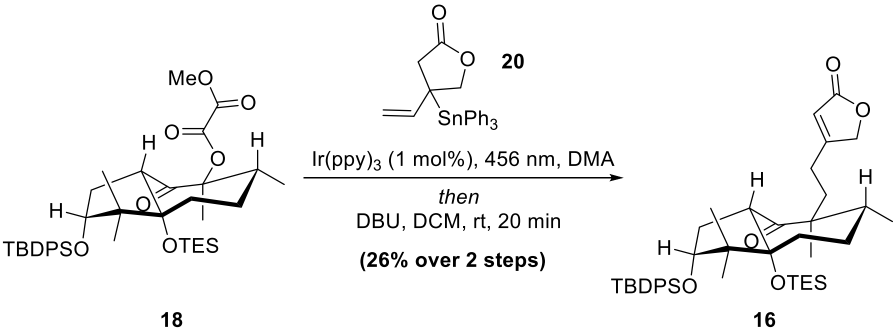

A 10 mL vial equipped with a stirring bar was charged with **18** (27 mg, 0.0388 mmol, 1 equiv) and **20** (90 mg, 0.194 mmol, 5 equiv) and transferred to the glove box. $Ir(ppy)_3$ (0.132 mg, 0.2 µmol, 1 mol%) was added as a solution in 0.4 mL of degassed, anhydrous DMA. The vial was sealed with a screw cap and removed from the glove box. The reaction mixture was irradiated (2 x 40 W blue LED lamps, 456 nm) for 18 h. The temperature has risen to 45 °C during the reaction. The reaction mixture was diluted with sat. aq. NH_4Cl (5 mL) and extracted with Et_2O (4x5 mL) (using a syringe). The combined organic fractions were transferred to a separatory funnel, washed two times with brine, dried over Na_2SO_4, and concentrated *in vacuo*. The residue was redissolved in DCM (7.7 mL, ~ 0.005 M), and DBU (29.6 mg, 0.194 mmol, 29 µL, 5 equiv) was added. The mixture was stirred at room temperature for 20 min. The reaction was diluted with DCM (15 mL) and washed with aq. HCl (0.5 M, 2x10 mL), then with sat. aq. $NaHCO_3$ (20 mL), and finally with brine (20 mL). The organic fraction was dried over anhydrous Na_2SO_4, filtered, and concentrated *in vacuo*. The residue was purified using column chromatography (gradient elution 100:1:1 (PE:EtOAc:TEA) to 100:5:1 (PE:EtOAc:TEA)). Organic fractions containing the desired product **16** (confirmed with TLC MS) were taken together, concentrated, and subjected to preparative HPLC (elution with 85:15 (Hex:EtOAc)) to deliver the desired product **16** (7 mg, 26% over 2 steps) as a colorless oil.

Experimental Notes:

- Using 5 equiv of **20** resulted in a moderate yield increase (26%, compared to 20% when 3 equiv of **20** were used)
- A remarkable yield decrease was observed during the synthesis of **21**, compared to the allylation with allyltriphenylstannane. This is due to the decomposition of **20** over the course of the reaction. Despite the significant excess of **20**, it was not detected in the reaction mixture after 18 hours. Instead, the side product **SI-13** was isolated in 54% yield. This can be explained via the mechanistic hypothesis presented below:

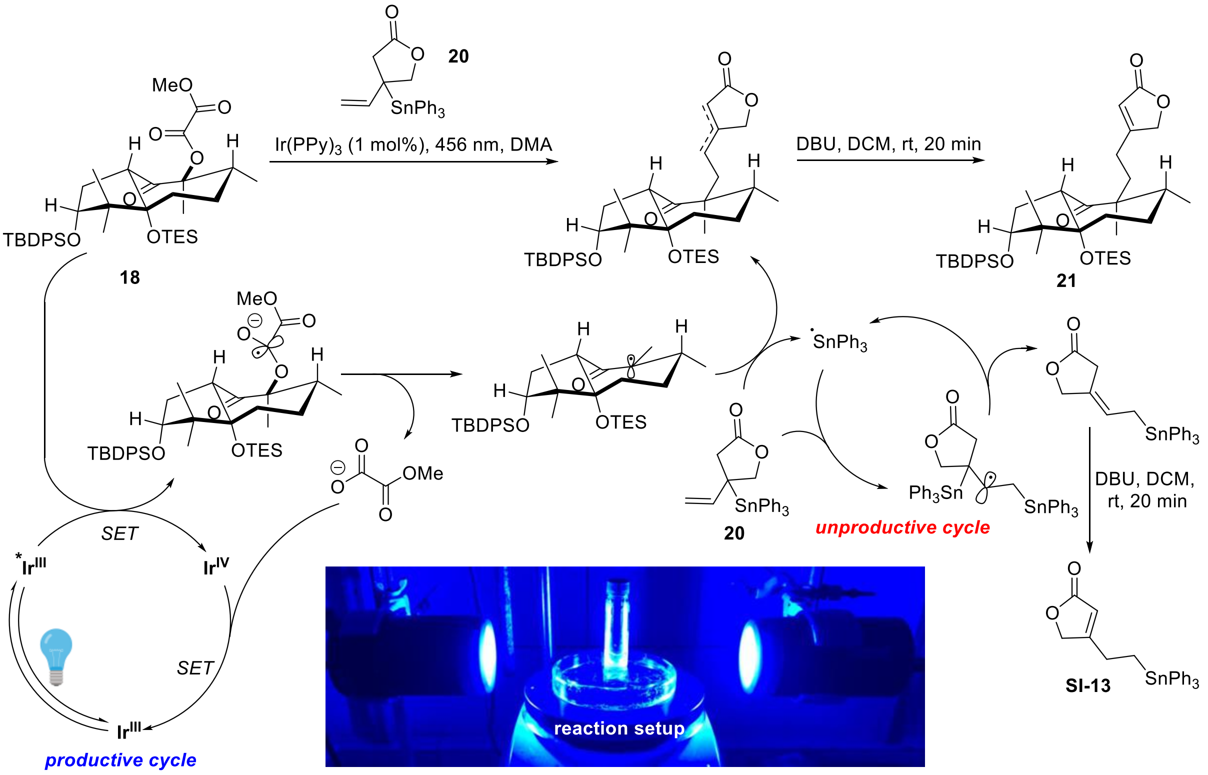

Analytical data SI-13:

^{1}H NMR (500 MHz, CDCl$_3$): δ 7.59 – 7.45 (m, 6H), 7.43 – 7.36 (m, 9H), 5.84 ("p", *J* = 1.7 Hz, 1H), 4.61 ("d", *J* = 1.9 Hz, 2H), 2.78 – 2.65 (m, 2H), 1.75 – 1.60 (m, 2H). **^{13}C NMR (126 MHz, CDCl$_3$)**: δ 174.0, 172.7, 137.5, 137.0, 129.5, 129.0, 115.3, 72.8, 25.8, 7.9.
IR (neat): ν 3063, 3046, 1778, 1748, 1637, 1428, 1171, 1075, 1036, 888, 731, 700.
HRMS (ESI): calcd for $C_{24}H_{22}O_{22}Sn$ [M+Na]$^+$: 485.0538, found: 485.0538
TLC: Rf = 0.30 (4:1 PE/EtOAc, KMnO$_4$).
Melting point: 112 °C

- The relative configuration of the obtained butenolide **16** was assigned by comparison with **19**. Nuclear Overhauser Effect Spectroscopy (NOESY) was not informative due to the massive signal overlap in the aliphatic region. Nevertheless, several relevant cross-peaks were used to confirm the relative configuration of **16**:

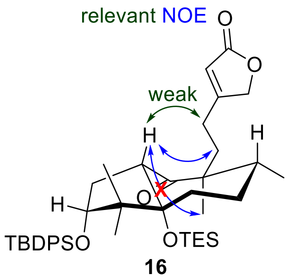

16

Analytical data 16:

^{1}H NMR (500 MHz, CDCl$_3$): δ 7.71 – 7.69 (m, 2H), 7.68 – 7.66 (m, 2H), 7.44 – 7.40 (m, 2H), 7.38 – 7.35 (m, 4H), 5.72 ("p", *J* = 1.7 Hz, 1H), 4.66 – 4.56 (m, 2H), 3.70 ("t", *J* = 7.8 Hz, 1H), 2.94 (dd, *J* = 12.9, 6.0 Hz, 1H), 2.63 (td, *J* = 12.9, 7.9 Hz, 1H), 2.19 (ddd, *J* = 17.0, 12.4, 4.6 Hz, 1H), 1.91 – 1.84 (m, 1H), 1.77 – 1.69 (m, 2H), 1.67 – 1.55 (m, 3H), 1.47 – 1.37 (m, 2H),

1.30 – 1.20 (m, 2H), 1.08 (s, 9H), 1.01 (t, J = 7.9 Hz, 9H), 0.98 (s, 3H), 0.95 (d, J = 7.0 Hz, 3H), 0.86 (s, 3H), 0.70 – 0.56 (m, 9H), 0.50 (s, 3H).**^{13}C NMR (126 MHz, $CDCl_3$)**: δ 210.2, 173.9, 170.1, 136.5, 136.4, 134.5, 134.4, 129.8, 129.7, 127.6, 127.6, 115.2, 90.3, 81.0, 73.1, 54.0, 52.4, 50.6, 41.1, 36.3, 35.7, 35.5, 29.0, 28.9, 27.3, 23.8, 20.4, 19.3, 18.4, 14.1, 7.6, 7.5.
IR (neat): ν 2957, 2932, 2877, 1780, 1750, 1700, 1639, 1469, 1110, 1084, 1011, 739, 704.
HRMS (ESI): calcd for $C_{42}H_{62}O_5Si_2$ $[M+Na]^+$: 725.4028, found: 725.4029
TLC: Rf = 0.40 (4:1 PE/EtOAc, $KMnO_4$).
$[\alpha]_D^{25}$ = 36.5 (c = 0.4, $CHCl_3$).

Synthesis of (+)-Randainin D (**3**)

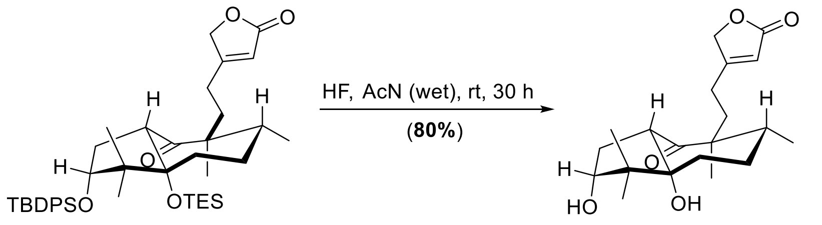

16
(+)-randainin D (**3**)

A 2 mL plastic Eppendorf tube equipped with a stirring bar was charged with **16** (4 mg, 0.0057 mmol, 1 equiv), AcN (1 mL), and 48% aq. HF (10 µL, 0.0284 mmol, 50 equiv). The reaction mixture was stirred for 15 h when an additional 48% aq. HF (10 µL, 0.0284 mmol, 50 equiv) was added. After an extra 15 hours, the reaction mixture was quenched with sat. aq. $NaHCO_3$ (0.5 mL) and volatiles were removed *in vacuo*. The residue was dissolved in 10 mL of EtOAc and washed with brine (5 mL x2). Combined aqueous fractions were extracted with EtOAc (5 mL), and the combined organic fractions were dried over anhydrous Na_2SO_4, filtered, and concentrated *in vacuo*. The residue was purified using column chromatography (gradient elution 5:1 (PE:EtOAc) to 3:1 (PE:EtOAc)) to afford (+)-Randainin D (**3**) (1.6 mg, 80%) as a white solid.

^{1}H NMR (500 MHz, $CDCl_3$): δ 5.78 ("p", J = 1.7 Hz, 1H), 4.69 ("d", J = 1.8 Hz, 2H), 3.87 (dd, J = 5.9, 3.5 Hz, 1H), 3.64 (dd, J = 10.6, 5.8 Hz, 1H), 3.47 (d, J = 2.0 Hz, 1H), 2.37 (dd, J = 15.2, 5.9 Hz, 1H), 2.20 – 2.17 (m, 2H), 2.13 (d, J = 3.9 Hz, 1H), 2.07 – 2.04 (m, 1H), 2.03 – 2.01 (m, 1H), 1.92 – 1.86 (m, 1H), 1.83 – 1.76 (m, 2H), 1.65 – 1.58 (m, 2H), 1.37 (dt, J = 14.4, 3.6 Hz, 1H), 1.06 (s, 3H), 1.05 (s, 3H), 1.00 (d, J = 6.9 Hz, 3H), 0.88 (s, 3H). **^{13}C NMR (126 MHz, $CDCl_3$ at 77.16 ppm)**: δ 213.4, 173.9, 170.1, 115.4, 85.5, 82.4, 73.2, 55.2, 53.2, 50.8, 38.5, 36.3, 34.8, 32.9, 27.4, 23.6, 23.5, 18.8, 16.3, 15.2.
HRMS (ESI): calcd for $C_{20}H_{30}O_5$ $[M+Na]^+$: 373.1985, found: 373.1987.
$[\alpha]_D^{20}$ = 41.25 (c = 0.16, MeOH)

(+)-Randainin D (**3**) analytical data comparison

Table 1. Analytical data comparison of synthetic and natural (+)-Randainin D (**3**):

**^{1}H NMR ($CDCl_3$)**	
Synthetic	***Natural***
5.78 ("p", J = 1.7 Hz, 1H)	5.78 (s, 1H)
4.69 ("d", J = 1.8 Hz, 2H)	4.69 (s, 2H)
3.87 (dd, J = 5.9, 3.5 Hz, 1H)	3.86 (d, J = 5.6, 1H)
3.64 (dd, J = 10.6, 5.8 Hz, 1H)	3.63 (dd, J = 10.3, 5.7 Hz, 1H)
**3.47 (d, J = 2.0 Hz, 1H) *OH***	-
2.37 (dd, J = 15.2, 5.9 Hz, 1H)	2.36 (dd, J = 15.1, 5.6 Hz, 1H)
2.20 – 2.17 (m, 2H) [**2.18 (m, 2H)**]	2.18 (t, J = 7.9, 2H)
**2.11 (d, J = 3.9 Hz, 1H) *OH***	-
2.07 – 2.04 (m, 1H) [**2.05 (m, 1H)**]	2.04 (m, 1H)
2.03 – 2.01 (m, 1H) [**2.02 (m, 1H)**]	2.02 (m, 1H)
1.94 – 1.86 (m, 1H) [**1.89 (m, 1 H)**]	1.89 (m, 1H)
1.83 – 1.76 (m, 2H)	1.79 (dd, J = 14.8, 3.2 Hz, 1H)<br>1.78 (d, J = 14.3 Hz, 1 H)
1.65 – 1.58 (m, 1H) [**1.61 (m, 1 H)**]	1.61 (t, J = 14.3)
1.37 (dt, J = 14.4, 3.6 Hz, 1H)	1.37 (d, J = 14.8, 1H)
1.06 (s, 3H)	1.06 (s, 3H)
1.05 (s, 3H)	1.05 (s, 3H)
1.00 (d, J = 6.9 Hz, 3H)	1.00 (d, J = 6.8 Hz, 3H)
0.88 (s, 3H)	0.88 (s, 3H)
**^{13}C NMR ($CDCl_3$ at 77.00 ppm)**	
213.3	213.4
173.8	173.8
169.9	170.0
115.3	115.3
85.3	85.5
82.2	82.2
73.0	73.0
55.0	55.1
53.0	53.0
50.6	50.6
38.4	38.3
36.2	36.2
34.7	34.7
32.7	32.7
27.2	27.2
23.5	23.5
23.4	23.3
18.6	18.6
16.1	16.1
15.0	15.0
**$[\alpha]_D^{20}$**	
41.25 (c = 0.16, MeOH)	26 (c = 0.1, MeOH)

Note: ^{13}C NMR shifts were referenced in the original study of Shen et al. [16] *against the residual $CDCl_3$ peak at 77.00 ppm. For comparison to the original data of natural (+)-Randainin D, chemical shifts for the synthetic (+)-Randainin D (3) in Table 1 are referenced accordingly. Analytical data provided in the synthetic part are referenced against the residual $CDCl_3$ peak at 77.16 ppm.*

Total synthesis of (+)-barekoxide (**21**)

Synthesis of 2-((1R,2R,4aS,8aS)-2-hydroxy-2,5,5,8a-tetramethyldecahydronaphthalen-1-yl)-N-methoxy-N-methylacetamide **(SI-14)**

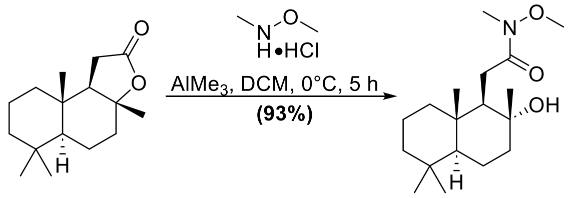

(+)-sclareolide (**22**) **SI-14**

The procedure for the preparation of **SI-14** is adapted from Nagamitsu *et al.*:[17]

A flame-dried 50 mL round-bottom flask equipped with a stirring bar was charged under an Ar atmosphere with the N,O-dimethylhydroxylamine hydrochloride (0.78 g, 8.0 mmol, 2 equiv), anhydrous, degassed DCM (4.0 mL), and cooled down to 0°C, using an ice-water bath. 1 M solution of Me_3Al in heptane (8.4 mL, 8.4 mmol, 2.1 equiv) was added dropwise using a syringe pump (rate 1 mL/min). After stirring for 2 h at 0°C, a solution of (+)-sclareolide (**22**) (1 g, 4.0 mmol, 1 equiv) in anhydrous, degassed DCM (4.0 mL) was added dropwise using a syringe pump (rate 1 mL/min). After stirring for an additional 5 h at 0°C, the reaction mixture was quenched with 10% aq H_2SO_4 (30 mL). The organic layer was removed from the obtained mixture using a separatory funnel. The aqueous phase was further extracted with DCM (20 mL x3). Combined organic fractions were successively washed with sat. aq. $NaHCO_3$ (50 mL) and with brine (50 mL), dried over anhydrous Na_2SO_4, filtered, and concentrated *in vacuo*. The obtained residue was purified using column chromatography (gradient elution 3:1 (PE:EtOAc) to 1:2 (PE:EtOAc)) to afford **SI-14** (1.16 g, 93%) as a white solid.

^{1}H NMR (500 MHz, $CDCl_3$): δ 3.71 (s, 3H), 3.17 (s, 3H), 2.56 (d, *J* = 17.1 Hz, 1H), 2.49 (s, 1H), 2.44 (dd, *J* = 16.7, 6.4 Hz, 1H), 1.66 (dq, *J* = 13.8, 3.5 Hz, 1H), 1.61 – 1.52 (m, 1H), 1.52 – 1.47 (m, 1H), 1.45 – 1.37 (m, 2H), 1.37 – 1.32 (m, 1H), 1.25 (qd, *J* = 13.5, 3.2 Hz, 1H), 1.16 – 1.10 (m, 4H), 1.01 (dd, *J* = 12.3, 2.3 Hz, 1H), 0.96 (td, J = 12.8, 3.9 Hz, 1H), 0.86 (s, 3H), 0.81 (s, 3H), 0.78 (s, 3H). **^{13}C NMR (126 MHz, $CDCl_3$):** δ 176.4, 73.0, 61.4, 56.4, 56.0, 44.6, 41.9, 39.3, 38.7, 33.5, 33.4, 32.7, 27.0, 23.4, 21.5, 20.7, 18.6, 15.9.
IR (neat): ν 3409, 2932, 2870, 1775, 1641, 1461, 1423, 1387, 1169, 1007.
HRMS (ESI): calcd for $C_{18}H_{33}NO_3$ $[M+Na]^+$: 334.2353, found: 334.2355
TLC: Rf = 0.1 (1.5:1 PE/EtOAc, $KMnO_4$).
$[\alpha]_D^{25}$ = 34.36 (c = 1.1, $CHCl_3$). [reported:[18] **$[\alpha]_D^{25}$** = 39.3 (c = 0.98, $CHCl_3$)]

Synthesis of (1R,2R,4aS,8aS)-1-(2-(methoxy(methyl)amino)-2-oxoethyl)-2,5,5,8a-tetramethyldecahydronaphthalen-2-yl methyl oxalate **(23)**

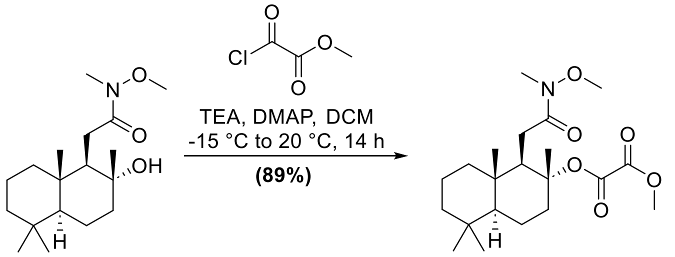

A flame-dried 25 mL round-bottom flask, equipped with a stirring bar, was charged, under an atmosphere of Ar, with the alcohol **SI-14** (249 mg, 0.8 mmol, 1.0 equiv), and anhydrous, degassed DCM (2 mL). The flask was then placed in an ice-water bath. 2 mL of DCM solution containing triethyl amine (138 mg, 1.36 mmol, 191 µL, 1.7 equiv) and DMAP (9.77 mg, 0.08 mmol, 10 mol%) was slowly added to the precooled flask. To the obtained reaction mixture, 4 mL of DCM solution containing monomethyl oxalyl chloride (118 mg, 0.96 mmol, 89 µL, 1.2 eq) was added dropwise, using a syringe pump (rate 0.2 mL/min). The reaction mixture was left to stir in the cooling bath for 14 h, where it slowly reached room temperature. Then it was quenched with sat. aq. $NaHCO_3$ (20 mL) and transferred to the separatory funnel. The organic phase was removed, and the aqueous was extracted with DCM (15 mL x3). The combined organic fractions were successively washed with HCl (0.5 M, 20 mL) with sat. aq. $NaHCO_3$ (40 mL), and finally with brine (40 mL). The obtained organic fraction was dried over anhydrous Na_2SO_4, filtered, and concentrated *in vacuo*. The residue was purified using column chromatography (gradient elution 4:1 (PE:EtOAc) to 1.5:1 (PE:EtOAc) to afford **23** (282 mg, 89%) as a white solid.

^{1}H NMR (500 MHz, $CDCl_3$): δ 3.78 (s, 3H), 3.70 (s, 3H), 3.16 (s, 3H), 2.72 (dt, *J* = 12.6, 3.4 Hz, 1H), 2.61 – 2.56 (m, 2H), 2.41 (dd, *J* = 17.9, 6.4 Hz, 1H), 1.89 (td, *J* = 12.6, 1.1 Hz, 1H), 1.76 – 1.71 (m, 1H), 1.60 (d, *J* = 0.9 Hz, 3H), 1.57 – 1.50 (m, 1H), 1.44 – 1.38 (m, 2H), 1.37 – 1.24 (m, 2H), 1.20 – 1.11 (m, 3H), 0.86 (s, 6H), 0.78 (s, 3H).**^{13}C NMR (126 MHz, $CDCl_3$):** 174.1, 159.0, 156.4, 91.7, 61.2, 55.2, 53.2, 53.1, 41.7, 39.0, 38.7, 38.5, 33.4, 33.2, 32.6, 27.2, 21.5, 20.7, 20.1, 18.3, 16.0.
IR (neat): ν 2960, 2926, 1750, 1727, 1662, 1430, 1389, 1347, 1202, 1165, 1120, 1007, 835.
HRMS (ESI): calcd for $C_{21}H_{35}NO_6$ $[M+Na]^+$: 420.2357, found: 420.2359
TLC: Rf = 0.23 (1.5:1 PE/EtOAc, $KMnO_4$).
$[\alpha]_D^{25}$ = -20.769 (c = 0.65, $CHCl_3$).
Melting point: 101.7 °C

Synthesis of 2-((1S,2S,4aS,8aS)-2-allyl-2,5,5,8a-tetramethyldecahydronaphthalen-1-yl)-N-methoxy-N-methylacetamide (**24**)

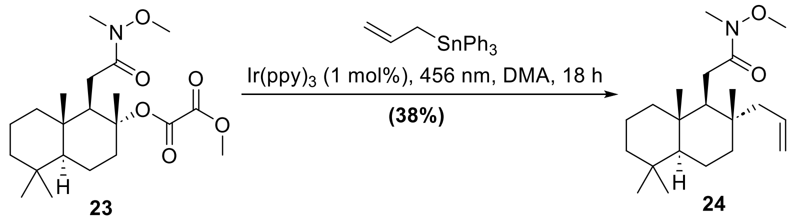

A 2 mL vial equipped with a stirring bar was charged with **23** (79.5 mg, 0.2 mmol, 1 equiv) and transferred to the glove box. Allyltriphenylstannane (391 mg, 1 mmol, 5 equiv) was added, followed by Ir(ppy)$_3$ (1.31 mg, 0.002 mmol, 1 mol%) as a solution in 2 mL of degassed, anhydrous DMA. The vial was sealed with a screw cap and removed from the glove box. The reaction mixture was irradiated (2 x 40 W blue LED lamps, 456 nm) for 18 h. The temperature has risen to 45 °C during the reaction. The reaction mixture was transferred to the separatory funnel and diluted with sat. aq. NH_4Cl (5 mL), and extracted with Et_2O (4x10 mL). The combined organic fractions were washed two times with brine, dried over Na_2SO_4, and concentrated *in vacuo*. The residue was subjected to column chromatography (gradient elution 100:1:1 (PE:EtOAc:TEA) to 100:5:1 (PE:EtOAc:TEA)). Organic fractions containing the desired product **24** (confirmed with TLC MS) were taken together, concentrated, and subjected to preparative HPLC (elution with 95:5 (Hex:EtOAc)) to deliver the desired product **24** (25.5 mg, 38%) as a colorless oil.

Experimental Notes:

- Anhydrous degassed DMF can be used instead of DMA, resulting in 24 mg yield of **24** (36% yield).
- When reaction was performed at 30 °C, the desired product was isolated in 27% yield (18 mg). Using 5 equiv of allyltributylstannane at 30 °C resulted in 10.5% yield (7 mg).
- Excess of allyltriphenylstannane can be recovered during the gradient elution, and an analytical sample suitable for further deoxygenative functionalization can be obtained via sequential recrystallization from ethanol.
- We did not succeed to scale up the reaction. To complete the total synthesis, two allylations of **23** were set up in parallel to provide 51 mg of the desired product **24**.
- It was not possible to determine the relative configuration of the obtained product **24** by Nuclear Overhauser Effect Spectroscopy (NOESY) due to the signals overlapping in the aliphatic region. The product **24** was transformed to the corresponding ketone **25**, which allowed us to determine the *trans*-relationships between the allyl group and the carbonyl chain.

^{1}H NMR (500 MHz, $CDCl_3$): δ 5.85 (dddd, J = 16.8, 10.1, 8.2, 6.5 Hz, 1H), 5.00 (ddt, J = 10.1, 2.5, 0.9 Hz, 1H), 4.93 (ddt, J = 16.9, 2.6, 1.4 Hz, 1H), 3.71 (s, 3H), 3.17 (s, 3H), 2.42 (dd, J = 18.1, 5.5 Hz, 1H), 2.29 (dd, J = 18.1, 3.9 Hz, 1H), 1.94 – 1.89 (m, 2H), 1.81 (ddt, J = 13.6, 8.2, 1.1 Hz, 1H), 1.58 – 1.49 (m, 2H), 1.48 – 1.45 (m, 1H), 1.40 – 1.28 (m, 5H), 1.15 (td, J = 13.3, 4.1 Hz, 1H), 1.03 – 0.94 (m, 2H), 0.88 (d, J = 0.8 Hz, 3H), 0.85 (s, 3H), 0.85 (s, 3H), 0.79 (s, 3H). **^{13}C NMR (126 MHz, $CDCl_3$)**: δ 175.6, 135.5, 117.3, 61.4, 56.1, 52.4, 48.8, 41.9, 39.5, 39.2, 38.9, 37.7, 33.39, 33.36, 32.9, 27.8, 21.7, 20.9, 18.8, 18.4, 16.5.
IR (neat): ν 2929, 2870, 1776, 1670, 1416, 1385, 1176, 1110, 1007, 910.

HRMS (ESI): calcd for $C_{21}H_{37}NO_2$ $[M+Na]^+$: 358.2717, found: 358.2724
TLC: Rf = 0.38 (10:1 PE/EtOAc, $KMnO_4$).
$[\alpha]_D^{25}$ = -16.33 (c = 0.6, $CHCl_3$).

Synthesis of 1-((1S,2S,4aS,8aS)-2-allyl-2,5,5,8a-tetramethyldecahydronaphthalen-1-yl)-3-methylbut-3-en-2-one (**25**)

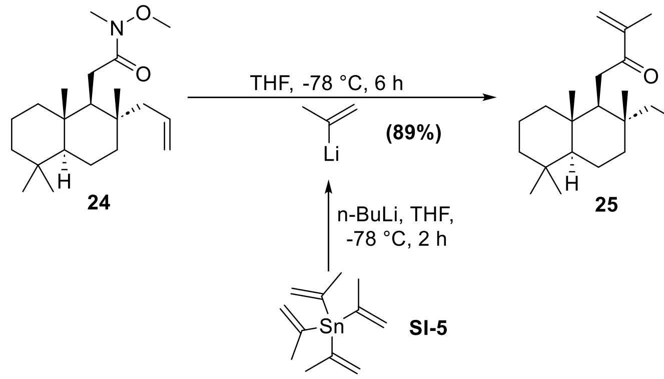

Tetra(prop-1-en-2-yl)stannane (**SI-5**) (93 mg, 0.328 mmol, 71 µL, 2.2 equiv) was used for the *in situ* preparation of *isopropenyl lithium*. It was placed into a flame-dried 10 mL round-bottom flask equipped with a stirring bar under an atmosphere of Ar. The flask was sealed with a rubber septum and charged with 1 mL of anhydrous, degassed THF. The reaction mixture was cooled down to -78 °C, and 2.5 M in hexanes *n*-BuLi (0.328 mmol, 0.13 mL, 2.2 eq) was added dropwise to the flask. It was stirred at -78 °C for 2 hours. Weinreb amide **24** (50 mg, 0.149 mmol, 1 equiv) was dissolved in 1 mL of anhydrous, degassed THF under an atmosphere of Ar. Then, it was added dropwise, using a syringe pump (rate 0.1 mL/min), to the flask containing *isopropenyl lithium* at -78 °C. The resulting reaction mixture was stirred for 6 hours at the same temperature. Then it was quenched with sat. aq. NH_4Cl (5 mL), warmed to r.t., transferred to the separatory funnel, diluted with Et_2O (10 mL), and aq. HCl (5 mL, 1 M). The organic phase was separated, and the aqueous was further extracted with Et_2O (10 mL x3). Combined organic fractions were successively washed with sat. aq. $NaHCO_3$ (20 mL) and with brine (20 mL), dried over anhydrous Na_2SO_4, filtered, and concentrated *in vacuo*. The obtained residue was purified using column chromatography (gradient elution 200:1 (PE:Et_2O) to 10:1 (PE:Et_2O)) to afford **25** (42 mg, 89%) as a white solid.

Experimental Notes:

- The relative configuration of the obtained ketone 27 was determined by Nuclear Overhauser Effect Spectroscopy (NOESY):

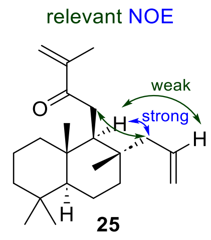

^{1}H NMR (500 MHz, CDCl$_3$): δ 5.99 (s, 1H), 5.80 (dddd, *J* = 16.8, 10.1, 8.2, 6.6 Hz, 1H), 5.71 (dt, *J* = 2.2, 1.1 Hz, 1H), 4.99 (dd, *J* = 10.1, 2.6 Hz, 1H), 4.90 (ddt, *J* = 17.0, 2.7, 1.4 Hz, 1H), 2.70 (dd, *J* = 18.6, 5.2 Hz, 1H), 2.52 (dd, *J* = 18.7, 3.6 Hz, 1H), 1.98 (dd, *J* = 5.2, 3.6 Hz, 1H), 1.89 (dd, *J* = 1.4, 0.8 Hz, 3H), 1.82 (dd, *J* = 13.7, 6.6 Hz, 1H), 1.71 (dd, *J* = 13.6, 8.2 Hz, 1H), 1.56 – 1.52 (m, 2H), 1.50 – 1.46 (m, 1H), 1.40 – 1.29 (m, 4H), 1.17 – 1.11 (m, 2H), 0.97 – 0.94 (m, 1H), 0.91 – 0.89 (m, 1H), 0.87 (s, 3H), 0.86 (s, 3H), 0.85 (s, 3H), 0.79 (s, 3H). **^{13}C NMR (126 MHz, CDCl$_3$)**: δ 201.4, 145.0, 135.3, 123.3, 117.3, 56.1, 51.9, 48.9, 42.0, 39.6, 39.5, 38.9, 37.6, 34.1, 33.38, 33.37, 21.7, 20.9, 18.8, 18.41, 18.39, 16.5.
IR (neat): ν 2957, 2926, 1676, 1636, 1451, 1387, 1370, 1091, 913.
HRMS (ESI): calcd for $C_{22}H_{36}O$ $[M+Na]^+$: 339.2658, found: 339.2653
TLC: Rf = 0.46 (100:1 PE/Et_2O, $KMnO_4$, or UV 254 nm).
$[\alpha]_D^{25}$ = -18.8 (c = 0.25, $CHCl_3$).
Melting point: 119 °C

Synthesis of (4aS,6aS,11aS,11bS)-4,4,6a,9,11b-pentamethyl-1,2,3,4,4a,5,6,6a,7,11,11a,11b-dodecahydro-10H-cyclohepta[a]naphthalen-10-one (**26**)

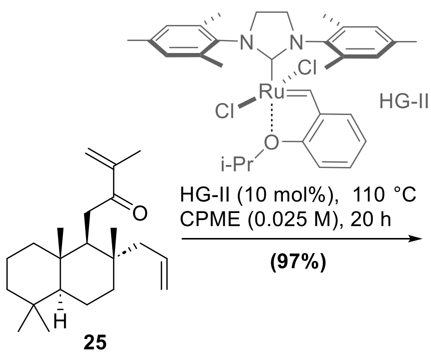

Diene **25** (38 mg, 0.12 mmol, 1 equiv) was placed into a flame-dried 10 mL round-bottom flask equipped with a stirring bar and introduced to the glove box. HG-II (7.52 mg, 0.012 mmol, 10 mol%) was added, followed by 4.8 mL of dry, degassed cyclopentyl methyl ether (CPME). The flask was sealed with the screw cap and stirred at r.t. until the catalyst was fully dissolved and then at 110 °C for 24 h. It was then cooled to room temperature and concentrated *in vacuo*. The obtained residue was purified using column chromatography (50:1 (PE:Et_2O)), to afford **26** (33.5 mg, 97%) as a white solid.

^{1}H NMR (500 MHz, CDCl$_3$): δ 6.21 (dddd, *J* = 8.0, 6.4, 3.1, 1.5 Hz, 1H), 2.50 (dd, *J* = 16.5, 1.6 Hz, 1H), 2.27 (dd, *J* = 16.5, 10.0 Hz, 1H), 2.06 (ddq, *J* = 15.2, 6.5, 1.7 Hz, 1H), 1.94 (ddd, *J* = 15.3, 7.5, 0.9 Hz, 1H), 1.82 (td, *J* = 1.6, 0.8 Hz, 3H), 1.71 (dtd, *J* = 12.7, 3.5, 1.4 Hz, 1H), 1.65 – 1.56 (m, 2H), 1.56 – 1.52 (m, 1H), 1.50 – 1.37 (m, 3H), 1.38 – 1.29 (m, 2H), 1.12 (td, *J* = 13.5, 4.7 Hz, 1H), 1.00 (d, *J* = 0.8 Hz, 3H), 0.86 – 0.82 (m, 7H), 0.81 (s, 3H), 0.79 – 0.73 (m, 1H). **^{13}C NMR (126 MHz, CDCl$_3$)**: δ 207.8, 139.1, 135.9, 56.8, 54.4, 44.4, 42.14, 42.13, 40.3, 39.9, 38.4, 36.9, 33.6, 33.4, 23.1, 21.4, 19.6, 19.3, 18.5, 16.6.
IR (neat): ν 2954, 2918, 1663, 1451, 1434, 1385, 1357, 1076, 997.
HRMS (ESI): calcd for $C_{20}H_{32}O$ $[M+Na]^+$: 311.2345, found: 311.2343
TLC: Rf = 0.17 (20:1 PE/Et_2O, $KMnO_4$, or UV 254 nm).
$[\alpha]_D^{25}$ = 128.64 (c = 0.22, $CHCl_3$).
Melting point: 78 °C

Synthesis of (+)-barekoxide (**21**)

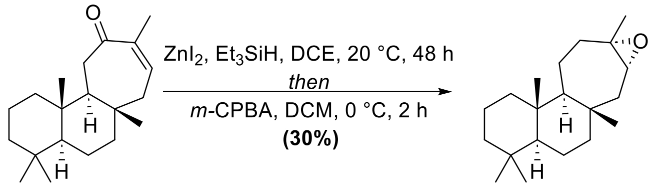

26

(+)-barekoxide (21)

The procedure for the reduction is adapted from Li *et al.*:[19]

A flame-dried 2 mL vial equipped with a stirring bar was charged with **26** (14 mg, 0.0485 mmol, 1 equiv), and introduced to the glove box. ZnI_2 (31 mg, 0.097 mmol, 2 equiv) was added, followed by anhydrous, degassed DCE (0.3 mL) and Et_3SiH (56.4 mg, 0.485 mmol, 78 µL, 10 equiv). The vial was stirred for 24 h, when another portion of Et_3SiH (56.4 mg, 0.485 mmol, 78 µL, 10 equiv) was added and stirring continued for additional 24 h. Volatiles were removed *in vacuo*, the obtained slurry was sonicated (10 min) with 1 ml of PE and filtered over a short silica gel pad. The fritted glass filter with silica gel was washed with PE (50 mL). The obtained PE fraction was concentrated *in vacuo,* the residue was placed into a flame-dried 10 mL round-bottom flask equipped with a stirring bar, dissolved in 2 ml of dry DCM, and cooled to 0 °C using an ice-water bath. *m*-CPBA (16.7 mg, 0.097 mmol, 2 equiv) was dissolved in 2 mL of dry DCM and added dropwise, using a syringe pump (rate 0.2 mL/min). The reaction mixture was then stirred for 2 h at 0 °C, quenched with 4 mL of sat. aq. $Na_2S_2O_3$, transferred to the separatory funnel, and diluted with sat. aq. $NaHCO_3$ (10 mL). Organic phase was removed, and aqueous was extracted with Et_2O (5 mL x3). Combined organic fractions were washed with brine (20 mL), dried over anhydrous Na_2SO_4, filtered, concentrated *in vacuo*, and subjected to preparative HPLC (elution with 98:2 (Hex:EtOAc)) to deliver (+)-barekoxide (**21**) (4.2 mg, 30% over 2 steps) as a white solid.

^{1}H NMR (400 MHz, C_6D_6): δ 2.60 (t, *J* = 7.2 Hz, 1H), 1.89 (dd, *J* = 13.9, 7.7 Hz, 1H), 1.71 (dd, *J* = 14.0, 6.6 Hz, 1H), 1.61 – 1.48 (m, 3H), 1.43 (t, *J* = 12.9 Hz, 1H), 1.39 – 1.25 (m, 6H), 1.22 (s, 3H), 1.19 – 0.95 (m, 4H), 0.86 (s, 3H), 0.84 (s, 3H), 0.81 (s, 3H), 0.73 (s, 3H), 0.61 – 0.58 (m, 1H), 0.57 – 0.52 (m, 1H).
^{1}H NMR (600 MHz, C_6D_6): δ 2.60 (t, *J* = 7.2 Hz, 1H), 1.89 (dd, *J* = 14.0, 7.8 Hz, 1H), 1.71 (dd, *J* = 14.0, 6.6 Hz, 1H), 1.62 – 1.56 (m, 1H), 1.56 – 1.49 (m, 2H), 1.43 (brt, *J* = 13.1 Hz, 1H), 1.39 – 1.31 (m, 3H), 1.30 – 1.23 (m, 3H), 1.22 (s, 3H), 1.18 – 1.11 (m, 1H), 1.11 – 1.06 (m, 1H), 1.03 (td, *J* = 13.2, 4.1 Hz, 1H) 0.86 (s, 3H), 0.85 (s, 3H), 0.80 (s, 3H), 0.73 (s, 3H), 0.62 – 0.57 (m, 2H), 0.58 – 0.53 (m, 1H)
^{13}C NMR (151 MHz, C_6D_6): δ 64.5, 60.0, 59.9, 56.3, 48.0, 44.2, 42.1, 40.3, 39.0, 37.8, 36.7, 33.60, 33.59, 22.7, 21.8, 20.4, 19.8, 19.2, 18.9, 16.2.
HRMS (ESI): calcd for $C_{20}H_{34}O$ $[M+H]^+$: 291.2682, found: 291.2674.
$[\alpha]_D^{25}$ = 1.37 (c = 0.2, $CHCl_3$)

(+)-Barekoxide (**21**) analytical data comparison

Table 2. Analytical data comparison of synthetic and natural (+)-Barekoxide (**21**)

**^{1}H NMR (C_6D_6)**			
Synthetic (600 MHz)	Natural (500 MHz)[20]	Sarpong, Davies *et al* (400 MHz)[21]	Jefford *et al (500 MHZ)*[22] *Semisynthesis*
2.60 (t, J = 7.2 Hz, 1H)	2.58 (t, J = 7.1 Hz, 1H)	2.59 (t, J = 7.3 Hz, 1H)	2.59 (t, J = 7.5 Hz, 1H)
1.89 (dd, J = 14.0, 7.8 Hz, 1H)	1.88 (dd, J = 14.0, 7.3 Hz, 1H)	1.88 (dd, J = 13.7, 7.6 Hz, 1H)	1.88 (dd, J = 14.0, 8.0 Hz, 1H)
1.71 (dd, J = 14.0, 6.6 Hz, 1H)	1.70 (dd, J = 14.0, 6.7 Hz, 1H)	1.70 (dd, J = 14.0, 6.4 Hz, 1H)	1.71 (dd, J = 13.5, 6.0 Hz, 1H)
1.62 – 1.56 (m, 1H), 1.56 – 1.49 (m, 2H)	1.58 (dd, J = 7.9, 3.1 Hz, 1H)	1.61 – 1.22 (m, 10H)	1.58 (m, 1H), 1.55 (m, 1H), 1.55 (m, 1H), 1.50 (m, 1H)
	1.55 (m, 1H), 1.51 (m, 1H)		
1.43 (brt, J = 13.1 Hz, 1H)	1.42 (dd, J = 14.0, 7.0 Hz, 1H)		1.42 (brt, J = 13.0 Hz)
1.39 – 1.31 (m, 3H), 1.30 – 1.23 (m, 3H)	1.38 (m, 1H), 1.36 (m, 1H), 1.34 (m, 1H), 1.33 (m, 1H), 1.28 (m, 1H), 1.25 (m, 1H)		1.38 (m, 1H), 1.35 (m, 1H), 1.30 (m, 1H), 1.29 (m, 1H), 1.25 (m, 1H), 1.22 (m, 1H)
1.22 (s, 3H)	1.22 (s, 3H)	1.21 (s, 3H)	1.22 (s)
1.18 – 1.11 (m, 1H), 1.11 – 1.06 (m, 1H)	1.20 (m, 1H), 1.12 (m, 1H) 1.08 (m, 1H)	1.19 – 0.95 (m, 3H)	1.15 (m, 1H), 1.10 (m, 1H), 1.02 (m, 1H)
1.03 (td, J = 13.2, 4.1 Hz, 1H)	1.02 (dd, J = 12.8, 3.9 Hz, 1H)		
0.86 (s, 3H)	0.86 (s, 3H)	0.85 (s, 3H)	0.86 (s, 3H)
0.85 (s, 3H)	0.84 (s, 3H)	0.83 (s, 3H)	0.84 (s, 3H)
0.80 (s, 3H)	0.80 (s, 3H)	0.80 (s, 3H)	0.80 (s, 3H)
0.73 (s, 3H),	0.72 (s, 3H)	0.72 (s, 3H)	0.70 (s, 3H)
0.62 – 0.57 (m, 2H)	0.60 (m, 1H)	0.61 – 0.47 (m, 3H)	0.60 (m, 1H)
0.58 – 0.53 (m, 1H)	0.58 (m, 1H)		0.58 (m, 1H)
**^{13}C NMR (C_6D_6)**			
Synthetic (151 MHz)	Natural (126 MHz)	Sarpong, Davies *et al* (101 MHz)	**Not given**
64.5	64.4	64.5	
60.0	60.0	60.0	
59.9	59.8	59.9	
56.3	56.2	56.2	
48.0	47.9	48.0	
44.2	44.2	44.2	
42.1	42.1	42.1	
40.3	40.2	40.2	
39.0	38.9	38.9	
37.8	37.7	37.7	
36.7	36.6	36.6	
33.60	33.5	33.6	
33.59	33.5	33.6	
22.7	22.7	22.7	
21.8	21.7	21.8	

20.4	20.3	20.4	
19.8	19.8	19.8	
19.2	19.1	19.1	
18.9	18.8	18.8	
16.2	16.2	16.2	
**$[\alpha]_D$**			
1.37 (c = 0.2, $CHCl_3$) (25 °C)	13.9 (solvent and concentration are not specified)[22]	1.73 (c = 0.59, $CHCl_3$) (25 °C)	5.2 (c = 0.256, $CHCl_3$) (22 °C)

References

(1) Kuang, L.; Liu, L. L.; Chiu, P. Formal Total Synthesis of (+)-Cortistatins A and J. *Chemistry – A European Journal* **2015**, *21* (41), 14287–14291. https://doi.org/10.1002/chem.201502890.

(2) Hanessian, S.; Auzzas, L. Alternative and Expedient Asymmetric Syntheses of L-(+)-Noviose. *Org Lett* **2008**, *10* (2), 261–264. https://doi.org/10.1021/ol702655c.

(3) Kreutziger, J.; Jäger, A.; Metz, P. Asymmetric Transfer Hydrogenation of Prochiral Cyclic 1,3-Diketones. *Arkivoc* **2022**, *2023* (5), 43–53. https://doi.org/10.24820/ark.5550190.p011.915.

(4) Lopchuk, J. M.; Green, I. L.; Badenock, J. C.; Gribble, G. W. A Short, Protecting Group-Free Total Synthesis of Bruceollines D, E, and J. *Org Lett* **2013**, *15* (17), 4485–4487. https://doi.org/10.1021/ol402042f.

(5) Frigerio, M.; Santagostino, M.; Sputore, S. A User-Friendly Entry to 2-Iodoxybenzoic Acid (IBX). *J Org Chem* **1999**, *64* (12), 4537–4538. https://doi.org/10.1021/jo9824596.

(6) Giacomina, F.; Alexakis, A. Construction of Enantioenriched Cyclic Compounds by Asymmetric Allylic Alkylation and Ring-Closing Metathesis. *European J Org Chem* **2013**, *2013* (29), 6710–6721. https://doi.org/10.1002/ejoc.201300971.

(7) Krasovskiy, A.; Knochel, P. Convenient Titration Method for Organometallic Zinc, Magnesium, and Lanthanide Reagents. *Synthesis (Stuttg)* **2006**, *2006* (05), 0890–0891. https://doi.org/10.1055/s-2006-926345.

(8) McQueney, M. S.; Lee, S. L.; Swartz, W. H.; Ammon, H. L.; Mariano, P. S.; Dunaway-Mariano, D. Evidence for an Intramolecular, Stepwise Reaction Pathway for PEP Phosphomutase Catalyzed Phosphorus-Carbon Bond Formation. *J Org Chem* **1991**, *56* (25), 7121–7130. https://doi.org/10.1021/jo00025a031.

(9) Cheval, N. P.; Dikova, A.; Blanc, A.; Weibel, J.; Pale, P. Vinyl Nosylates: An Ideal Partner for Palladium-Catalyzed Cross-Coupling Reactions. *Chemistry – A European Journal* **2013**, *19* (27), 8765–8768. https://doi.org/10.1002/chem.201300127.

(10) Dikova, A.; Cheval, N. P.; Blanc, A.; Weibel, J.; Pale, P. Handy Protocols Using Vinyl Nosylates in Suzuki–Miyaura Cross-Coupling Reactions. *Adv Synth Catal* **2015**, *357* (18), 4093–4100. https://doi.org/10.1002/adsc.201500682.

(11) Weires, N. A.; Slutskyy, Y.; Overman, L. E. Facile Preparation of Spirolactones by an Alkoxycarbonyl Radical Cyclization–Cross-Coupling Cascade. *Angewandte Chemie International Edition* **2019**, *58* (25), 8561–8565. https://doi.org/10.1002/anie.201903353.

(12) Nawrat, C. C.; Jamison, C. R.; Slutskyy, Y.; MacMillan, D. W. C.; Overman, L. E. Oxalates as Activating Groups for Alcohols in Visible Light Photoredox Catalysis: Formation of Quaternary Centers by Redox-Neutral Fragment Coupling. *J Am Chem Soc* **2015**, *137* (35), 11270–11273. https://doi.org/10.1021/jacs.5b07678.

(13) Miyashita, M.; Yoshikoshi, A. Facile and Highly Efficient Conjugate Addition of Benzeneselenol to α,β-Unsaturated Carbonyl Compounds. *Synthesis (Stuttg)* **1980**, *1980* (08), 664–666. https://doi.org/10.1055/s-1980-29167.

(14) von Gyldenfeldt, F.; Marton, D.; Tagliavini, G. Wurtz-Type Reductive Coupling Reaction of Allyl Bromides and Haloorganotins in Cosolvent/H2O(NH4Cl)/Zn Media as a Route to Allylstannanes and Hexaaryldistannanes. *Organometallics* **1994**, *13* (3), 906–913. https://doi.org/10.1021/om00015a025.

(15) Schneider, H.; Krahfuß, M. J.; Radius, U. To Rearrange or Not to Rearrange: Reactivity of NHCs towards Chloro- and Hydrostannanes *R* $_2$ SnCl $_2$ (*R* = Me, Ph) and Ph $_3$ SnH. *Z Anorg Allg Chem* **2016**, *642* (22), 1282–1286. https://doi.org/10.1002/zaac.201600271.

(16) Cheng, H. H.; Cheng, Y. Bin; Hwang, T. L.; Kuo, Y. H.; Chen, C. H.; Shen, Y. C. Randainins A-D, Based on Unique Diterpenoid Architectures, from Callicarpa Randaiensis. *J Nat Prod* **2015**, *78* (8), 1823–1828. https://doi.org/10.1021/acs.jnatprod.5b00012.

(17) Ohtawa, M.; Hishinuma, Y.; Takagi, E.; Yamada, T.; Ito, F.; Arima, S.; Uchida, R.; Kim, Y.-P.; Ōmura, S.; Tomoda, H.; Nagamitsu, T. Synthesis and Structural Revision of Cyslabdan. *Chem Pharm Bull (Tokyo)* **2016**, *64* (9), 1370–1377. https://doi.org/10.1248/cpb.c16-00382.

(18) Kumar, C. N. S. S. P.; Chein, R.-J. Synthesis of Labdane Diterpenes Galanal A and B from (+)-Sclareolide. *Org Lett* **2014**, *16* (11), 2990–2992. https://doi.org/10.1021/ol501121v.

(19) Li, Z.; Deng, G.; Li, Y.-C. Zinc(II) Iodide-Triethylsilane: A Novel Mild Reduction System for Direct Deoxygenation of Aryl Aldehydes, Ketones, and α,β-Unsaturated Enones. *Synlett* **2008**, *2008* (19), 3053–3057. https://doi.org/10.1055/s-0028-1083627.

(20) Rudi, A.; Kashman, Y. Chelodane, Barekoxide, and Zaatirin--Three New Diterpenoids from the Marine Sponge Chelonaplysilla Erecta. *J Nat Prod* **1992**, *55* (10), 1408–1414. https://doi.org/10.1021/np50088a004.

(21) Lian, Y.; Miller, L. C.; Born, S.; Sarpong, R.; Davies, H. M. L. Catalyst-Controlled Formal [4 + 3] Cycloaddition Applied to the Total Synthesis of (+)-Barekoxide and (−)-Barekol. *J Am Chem Soc* **2010**, *132* (35), 12422–12425. https://doi.org/10.1021/ja103916t.

(22) Kuniyoshi, M.; Marma, M. S.; Higa, T.; Bernardinelli, G.; Jefford, C. W. 3-Bromobarekoxide, an Unusual Diterpene from Laurencia Luzonensis. *Chemical Communications* **2000**, No. 13, 1155–1156. https://doi.org/10.1039/b002530p.

^{1}H NMR (400 MHz, $CDCl_3$)

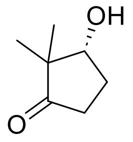

SI-1

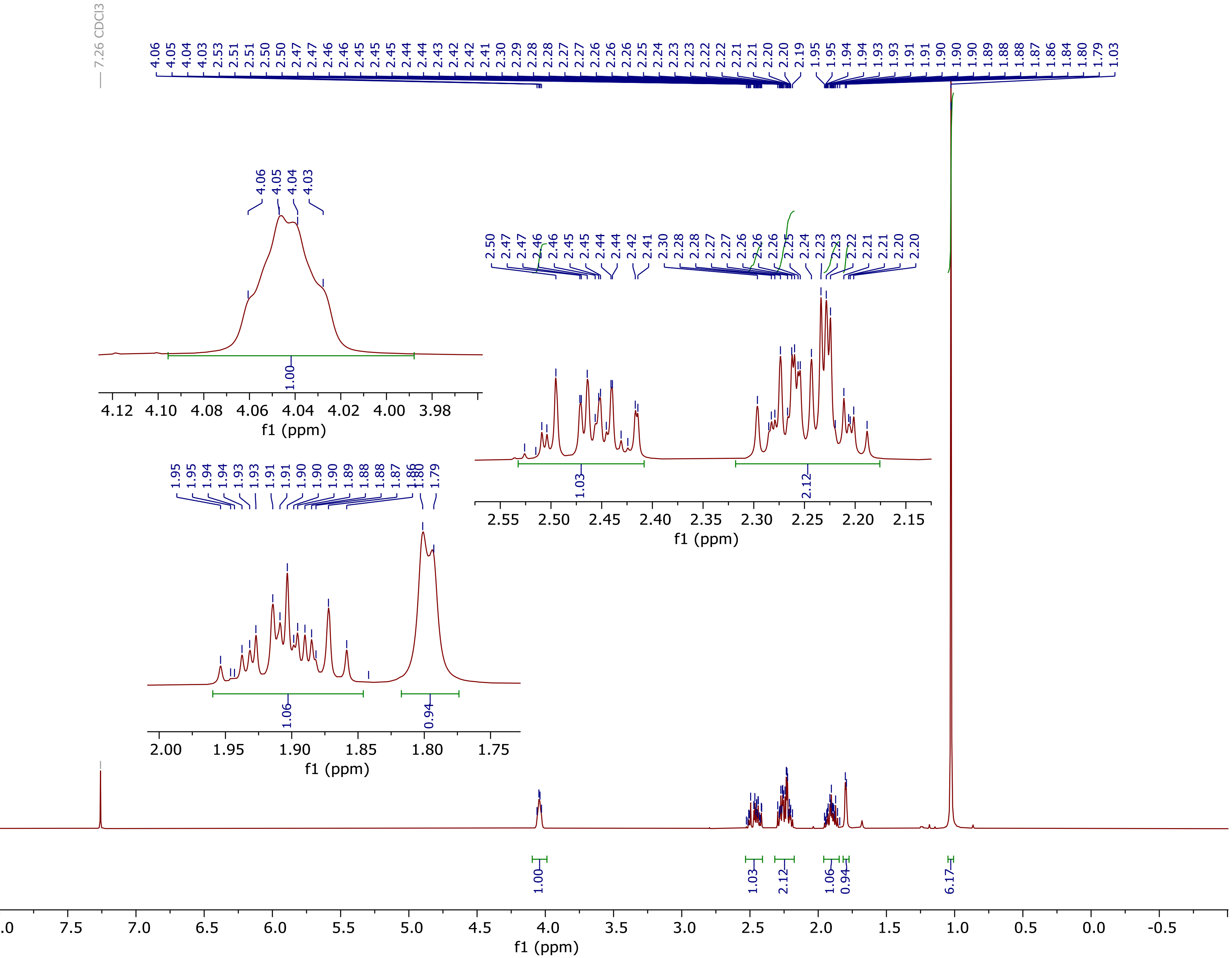

^{13}C NMR (101 MHz, $CDCl_3$)

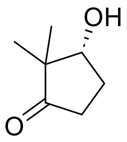

SI-1

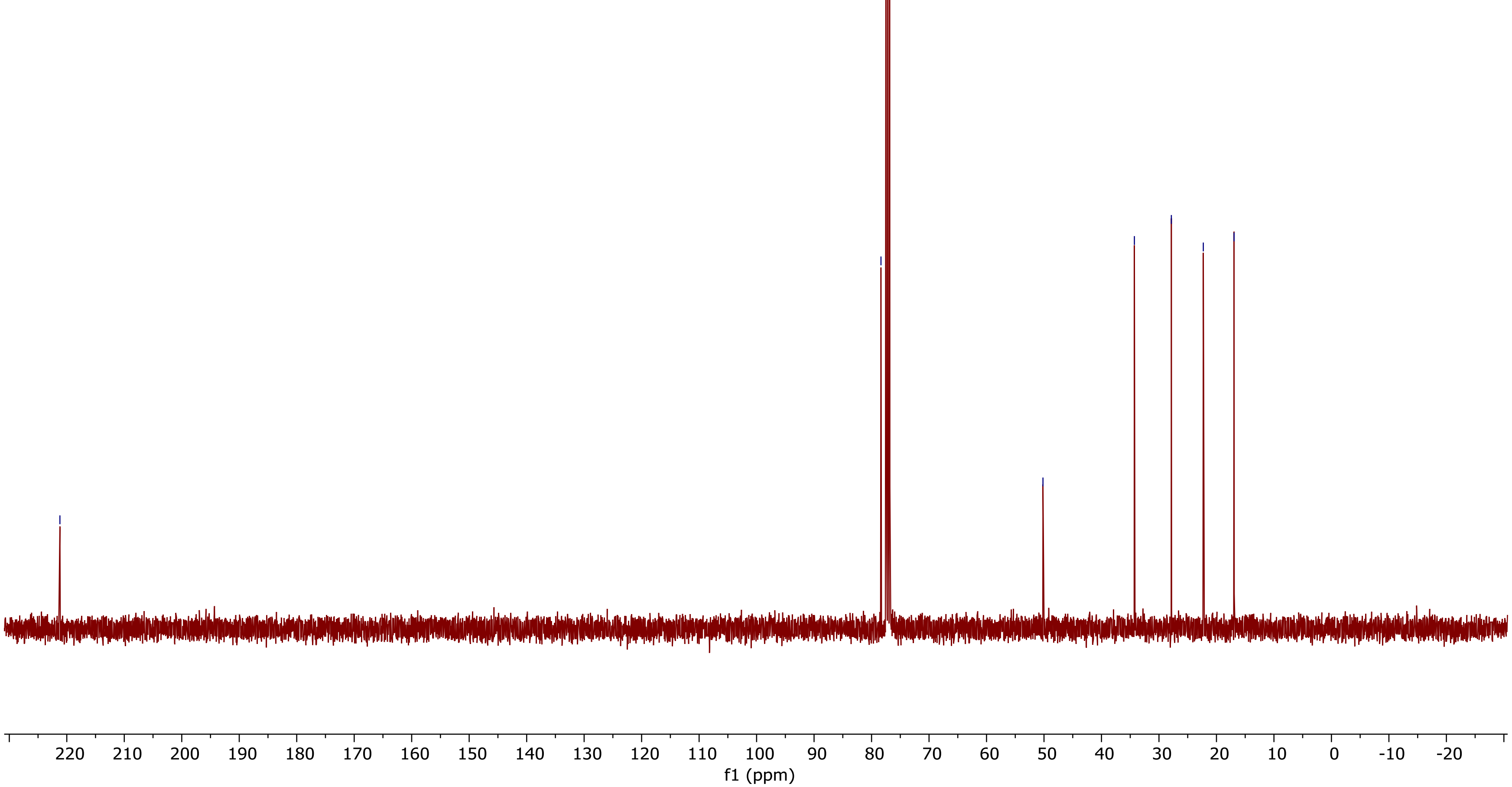

^{1}H NMR (500 MHz, $CDCl_3$)

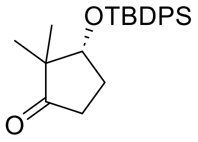

9

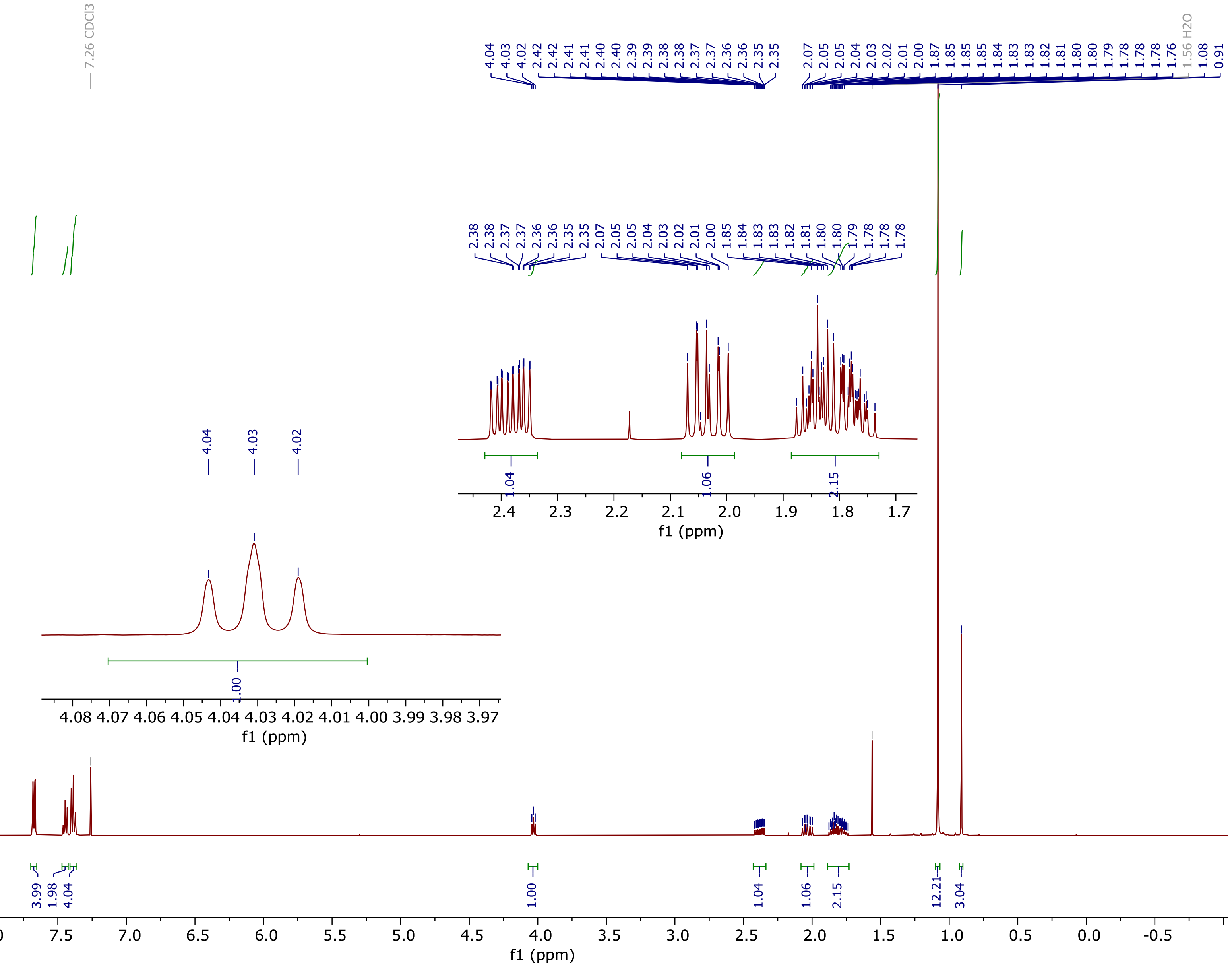

^{13}C NMR (126 MHz, $CDCl_3$)

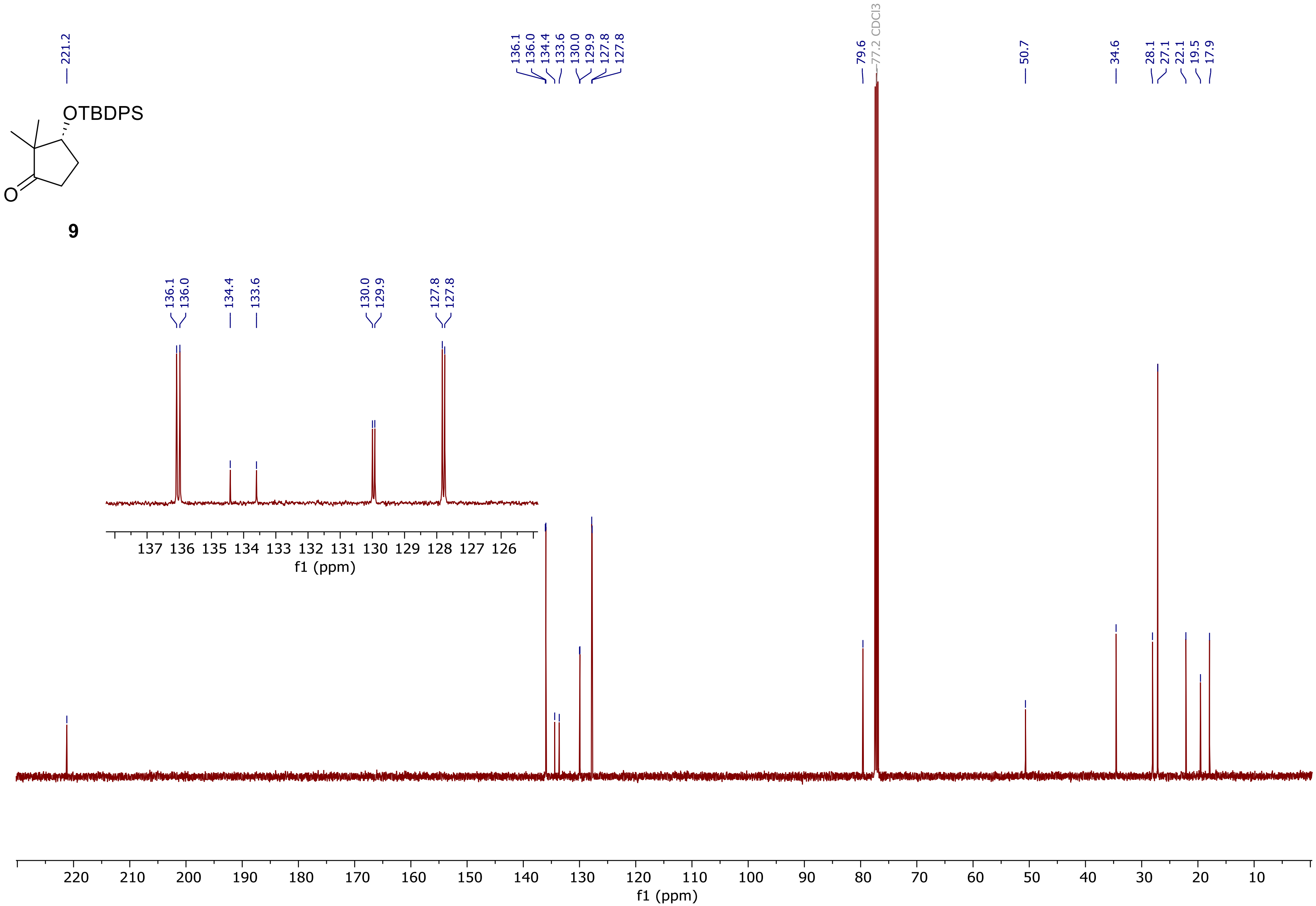

^{1}H NMR (500 MHz, $CDCl_3$)

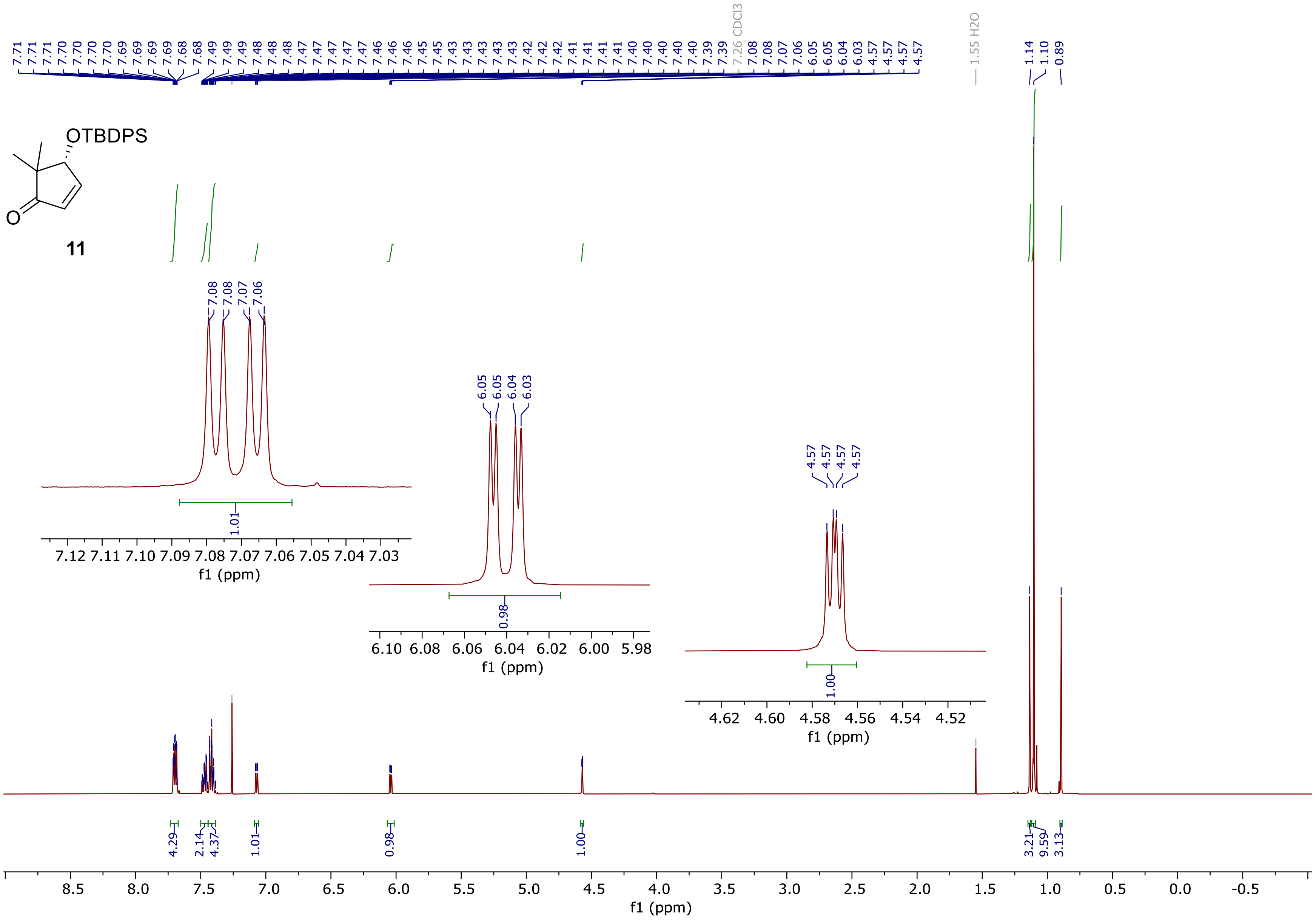

^{13}C NMR (126 MHz, CDCl$_3$)

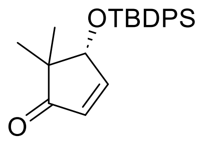

11

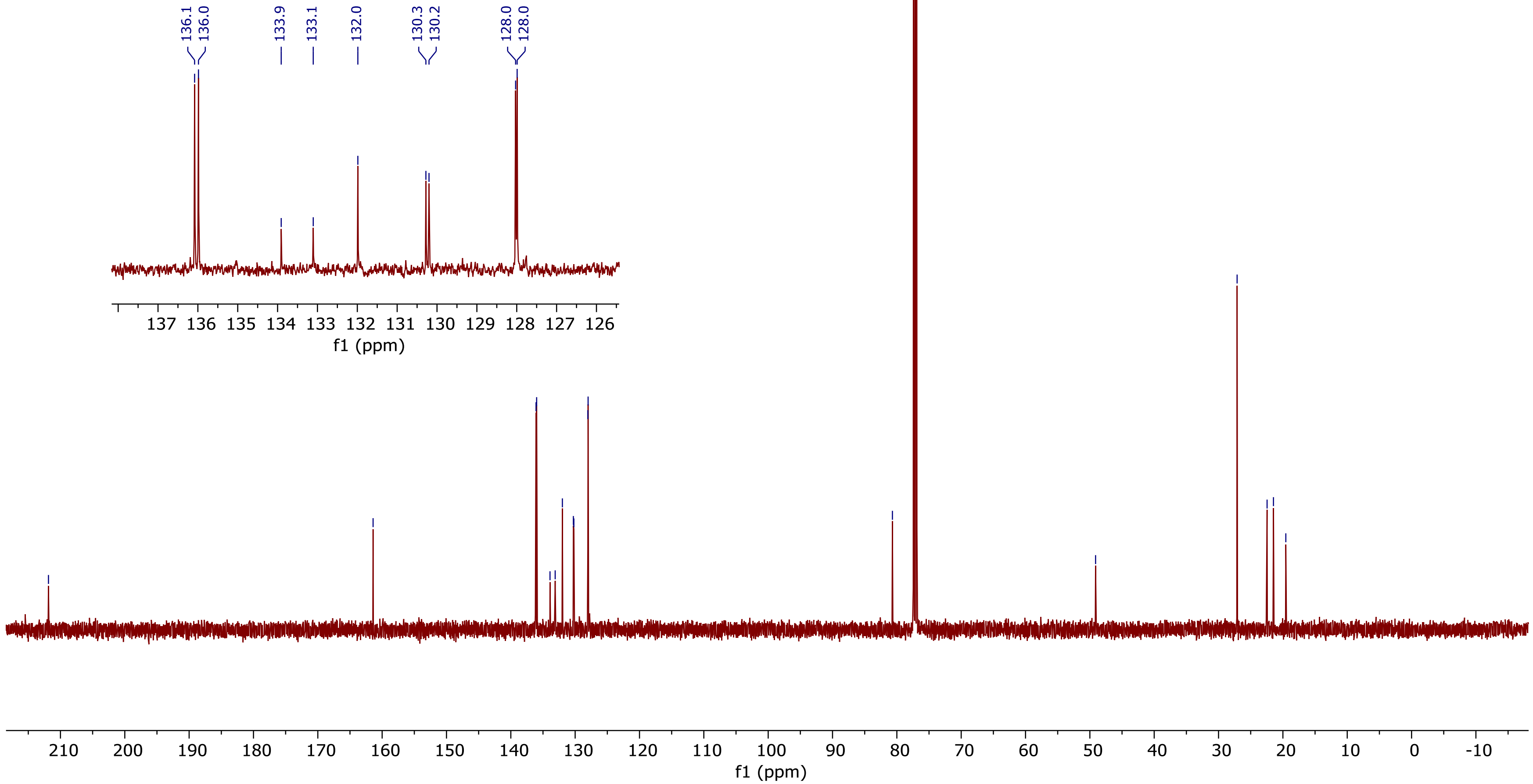

^{1}H NMR (500 MHz, $CDCl_3$)

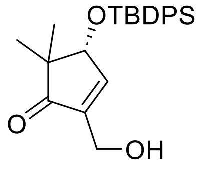

12

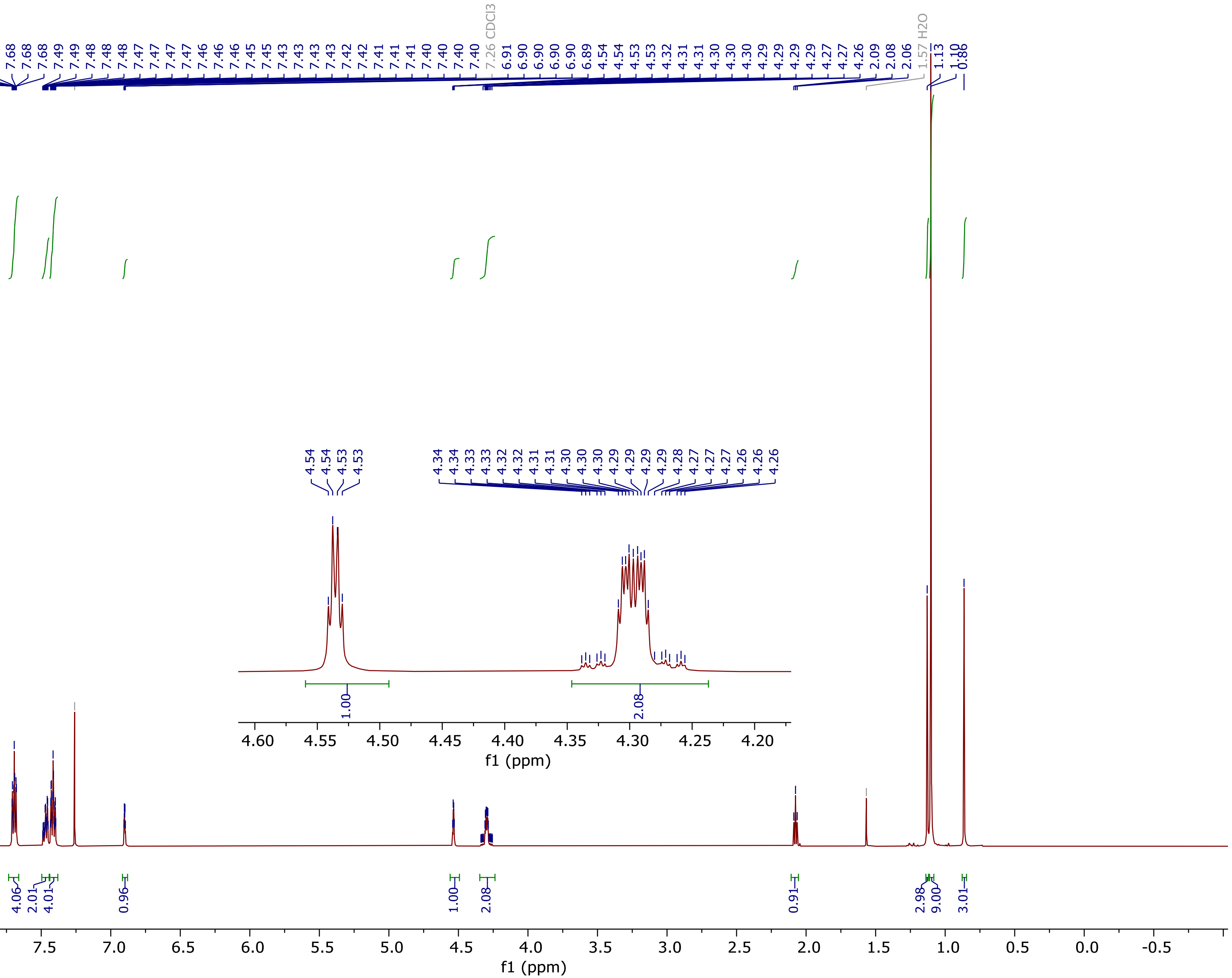

^{13}C NMR (126 MHz, $CDCl_3$)

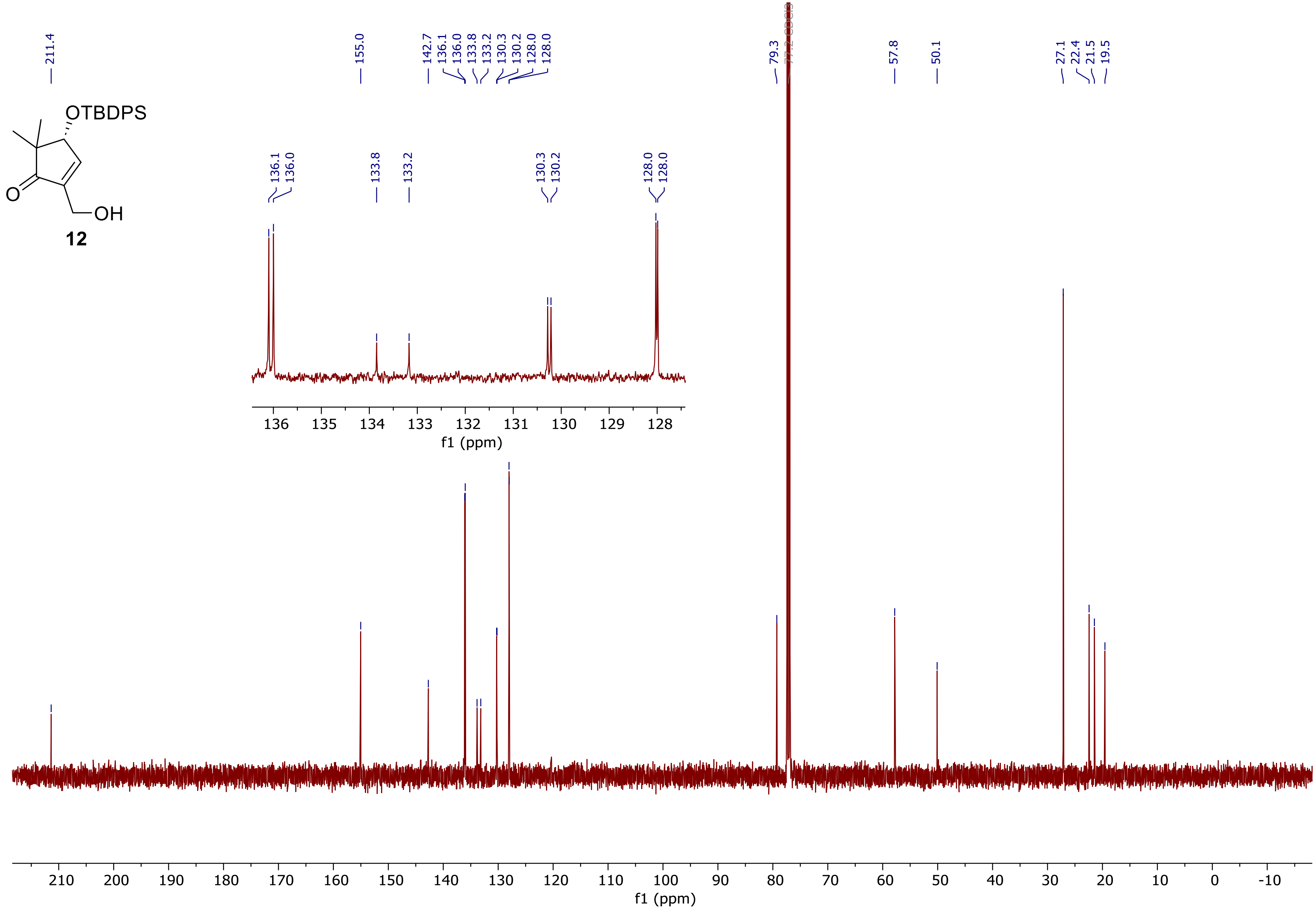

^{1}H NMR (500 MHz, $CDCl_3$)

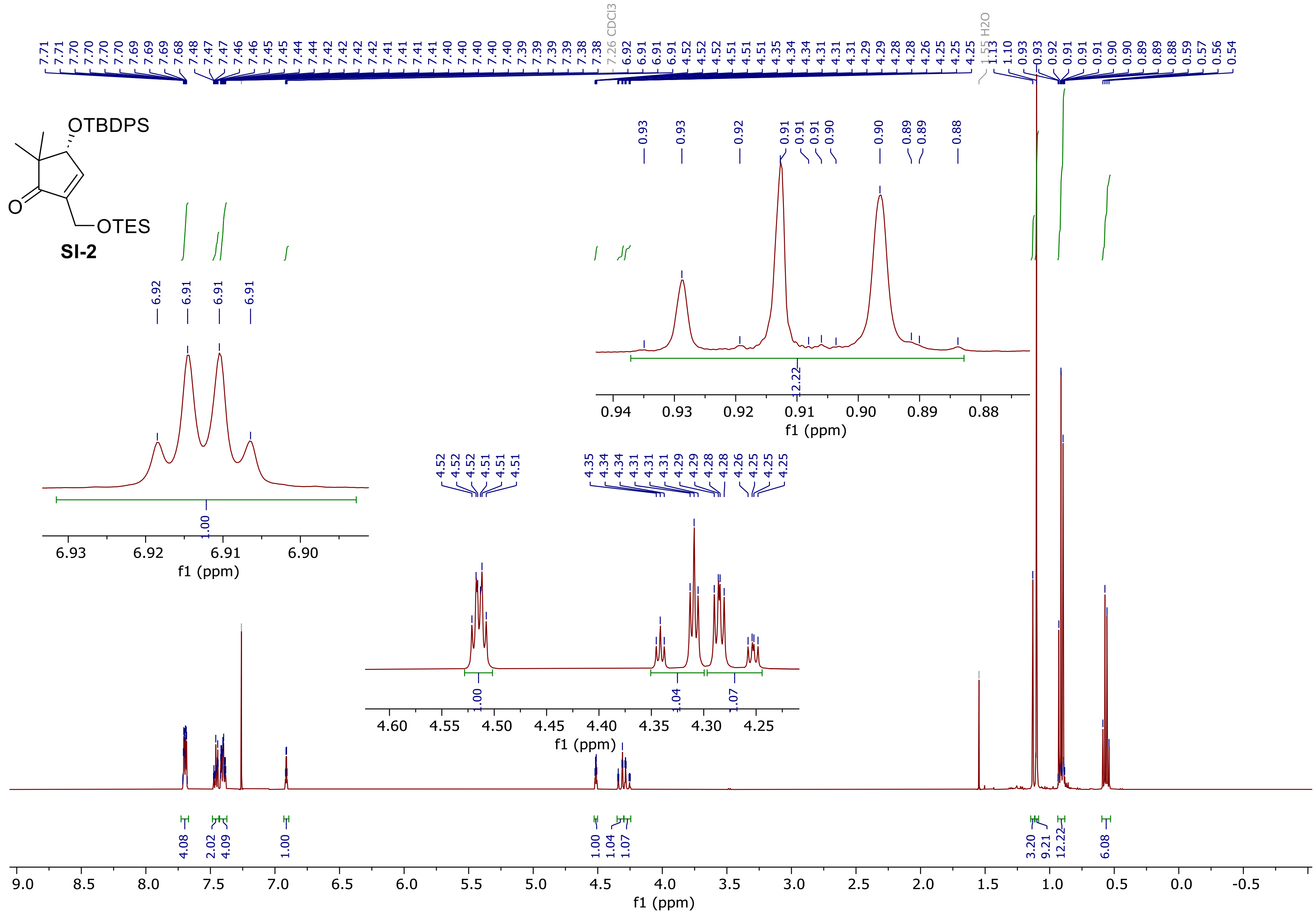

^{13}C NMR (126 MHz, $CDCl_3$)

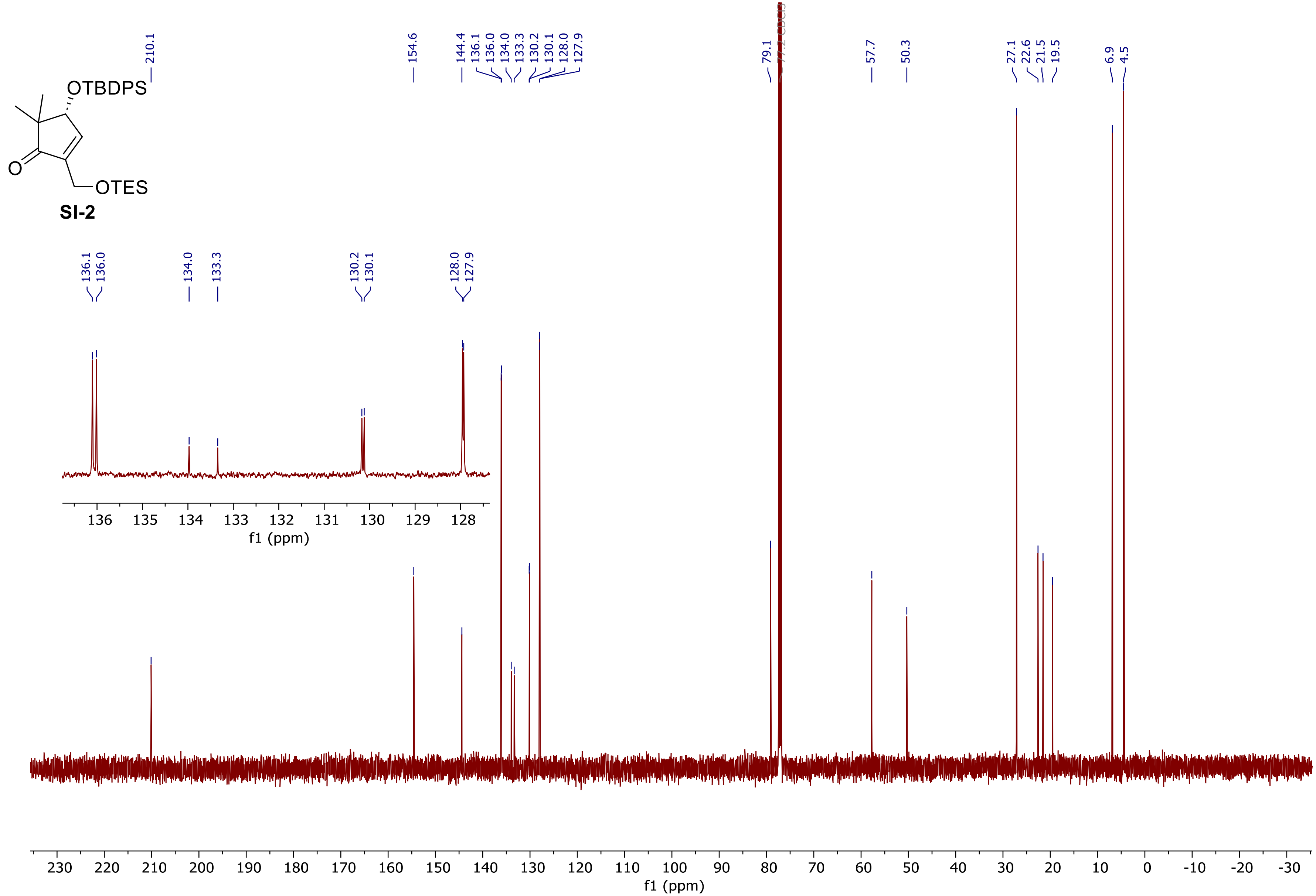

^{1}H NMR (500 MHz, $CDCl_3$)

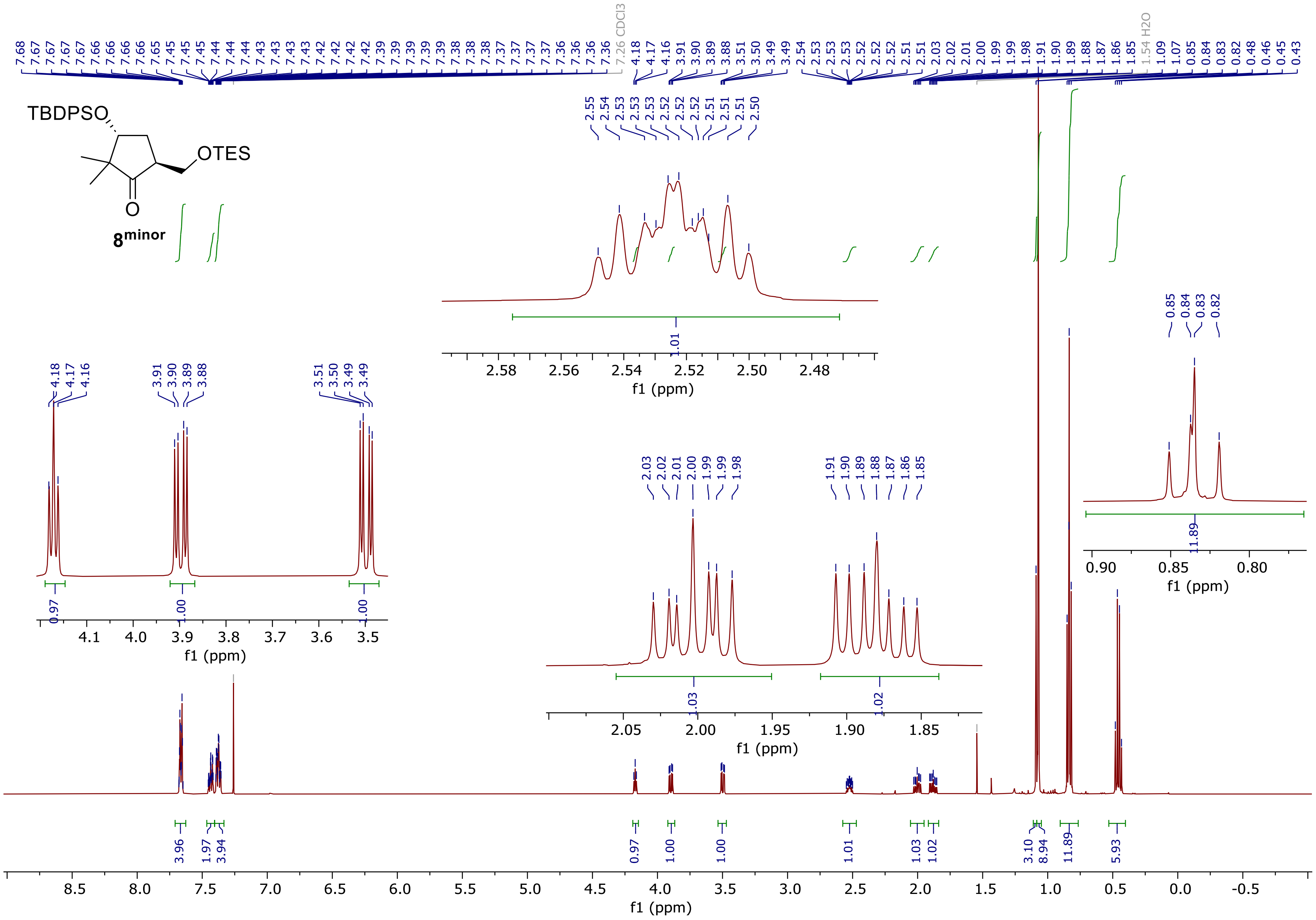

^{13}C NMR (126 MHz, $CDCl_3$)

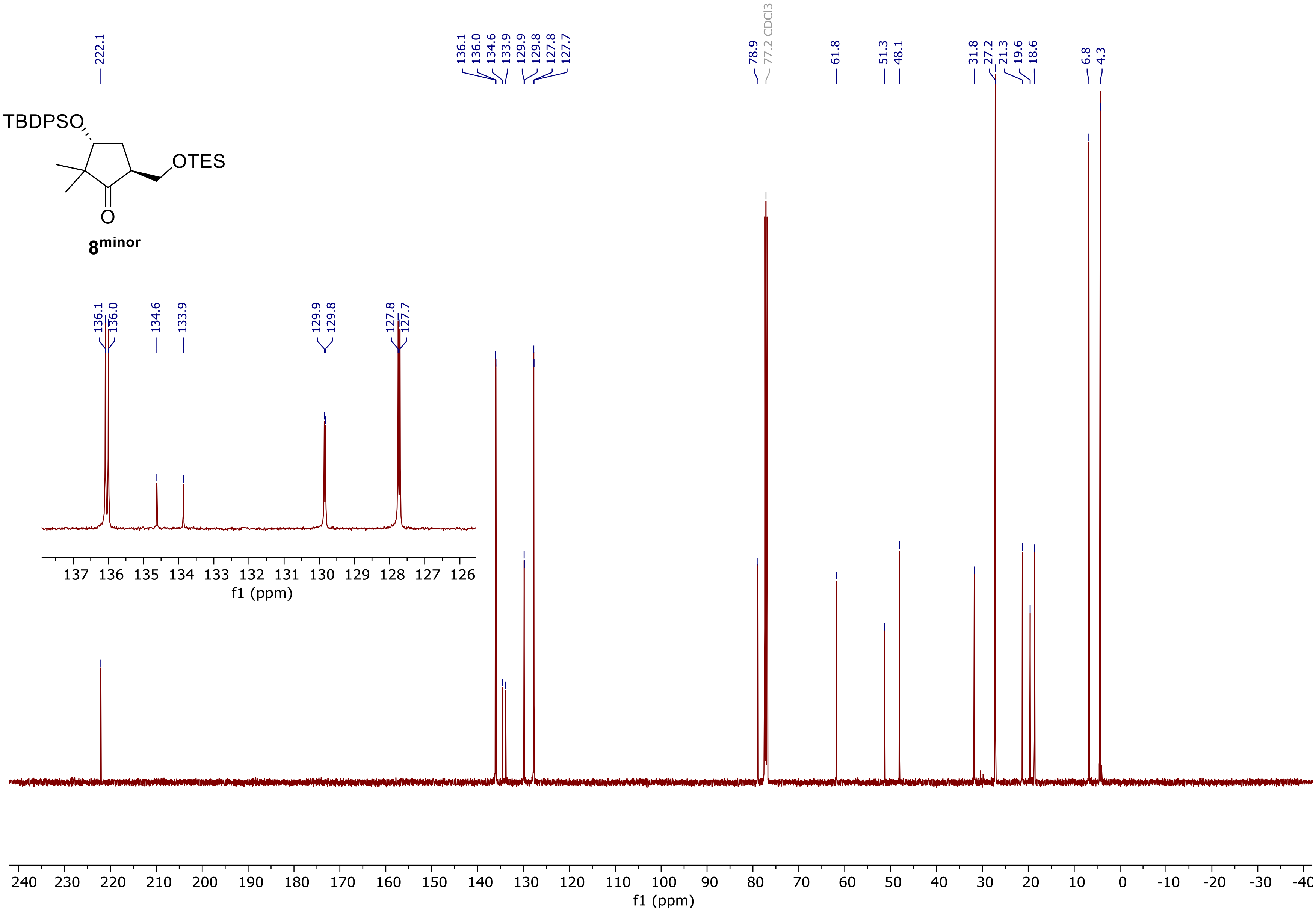

NOESY (500 MHz, $CDCl_3$)

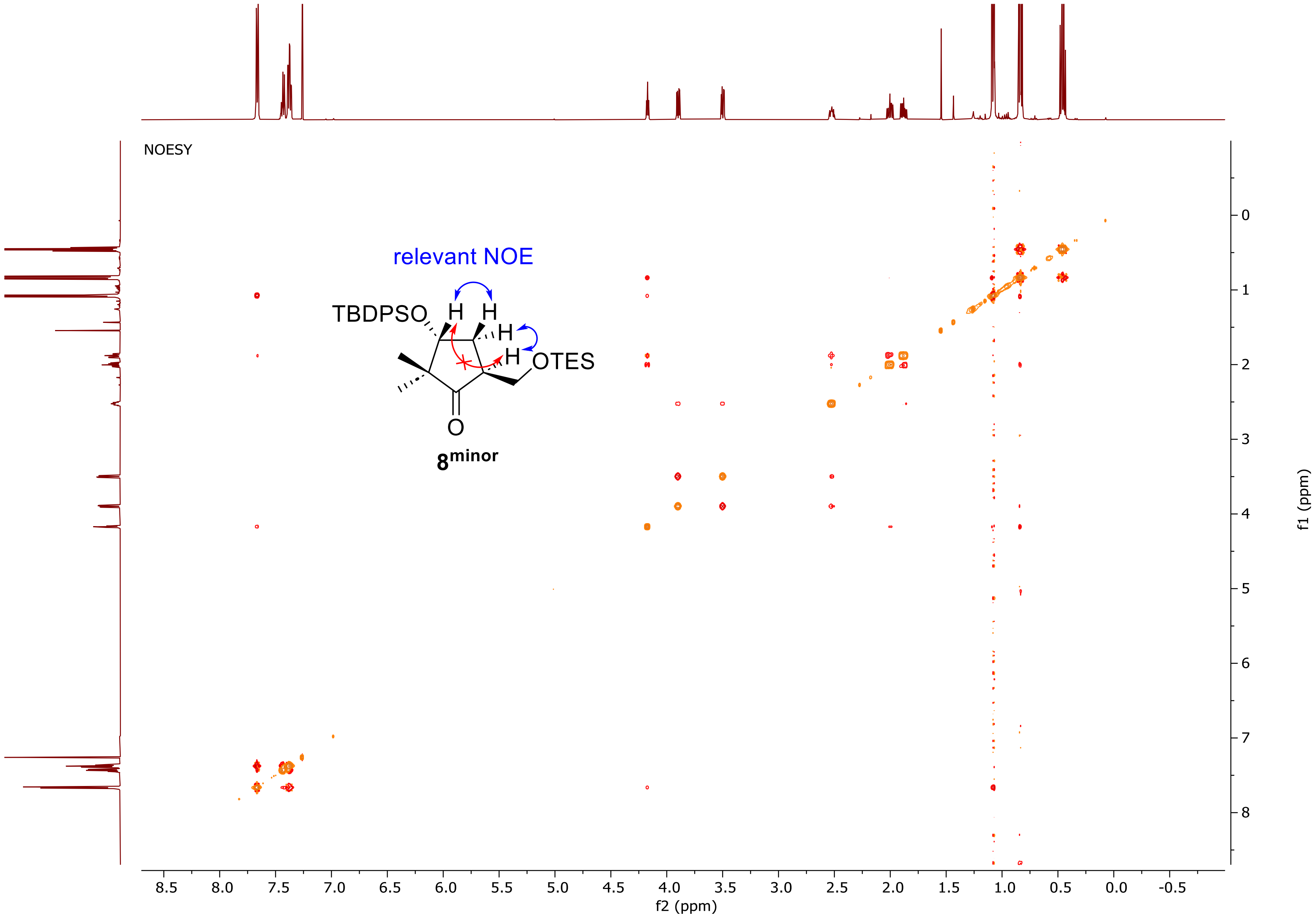

^{1}H NMR (500 MHz, $CDCl_3$)

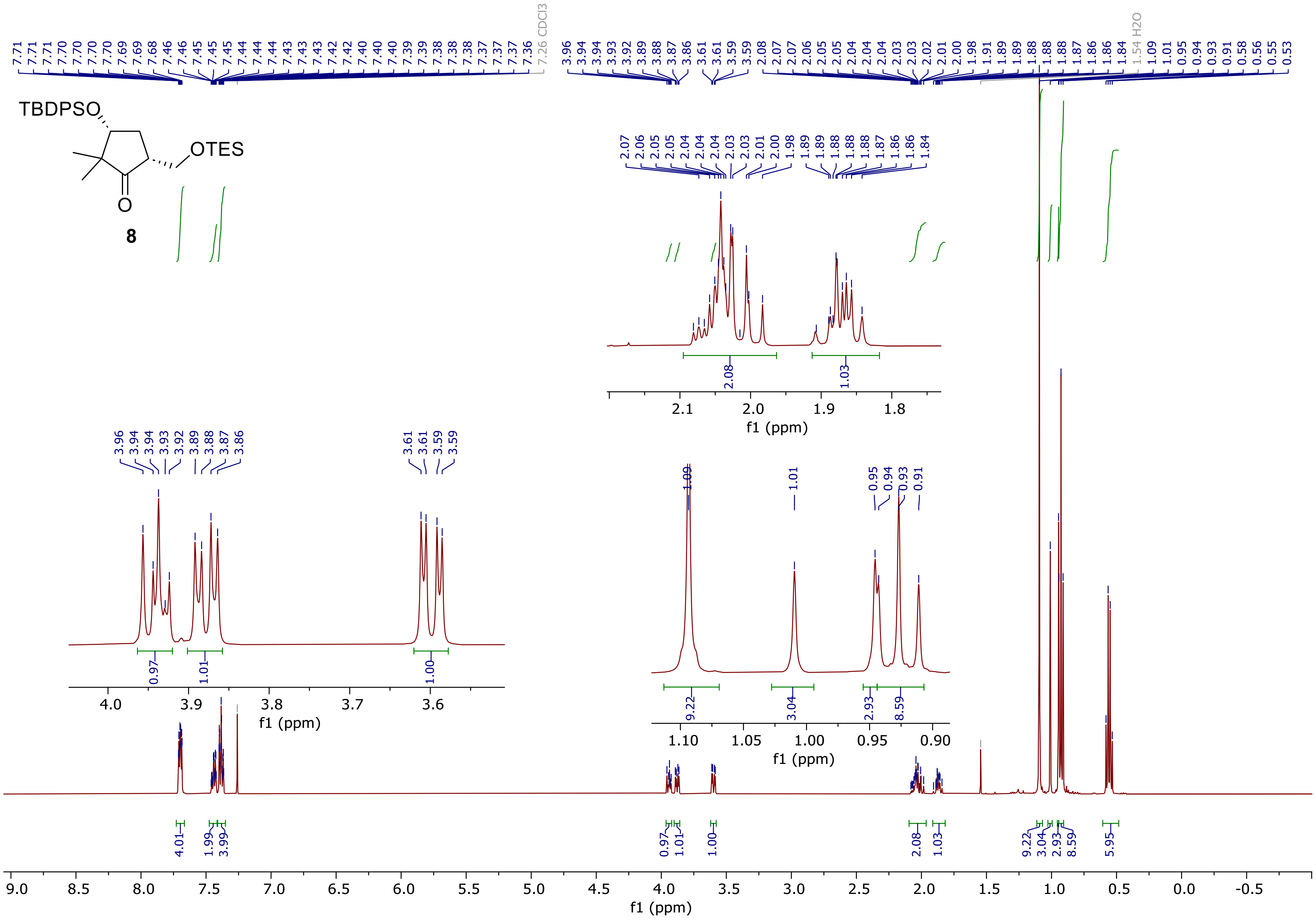

^{13}C NMR (126 MHz, $CDCl_3$)

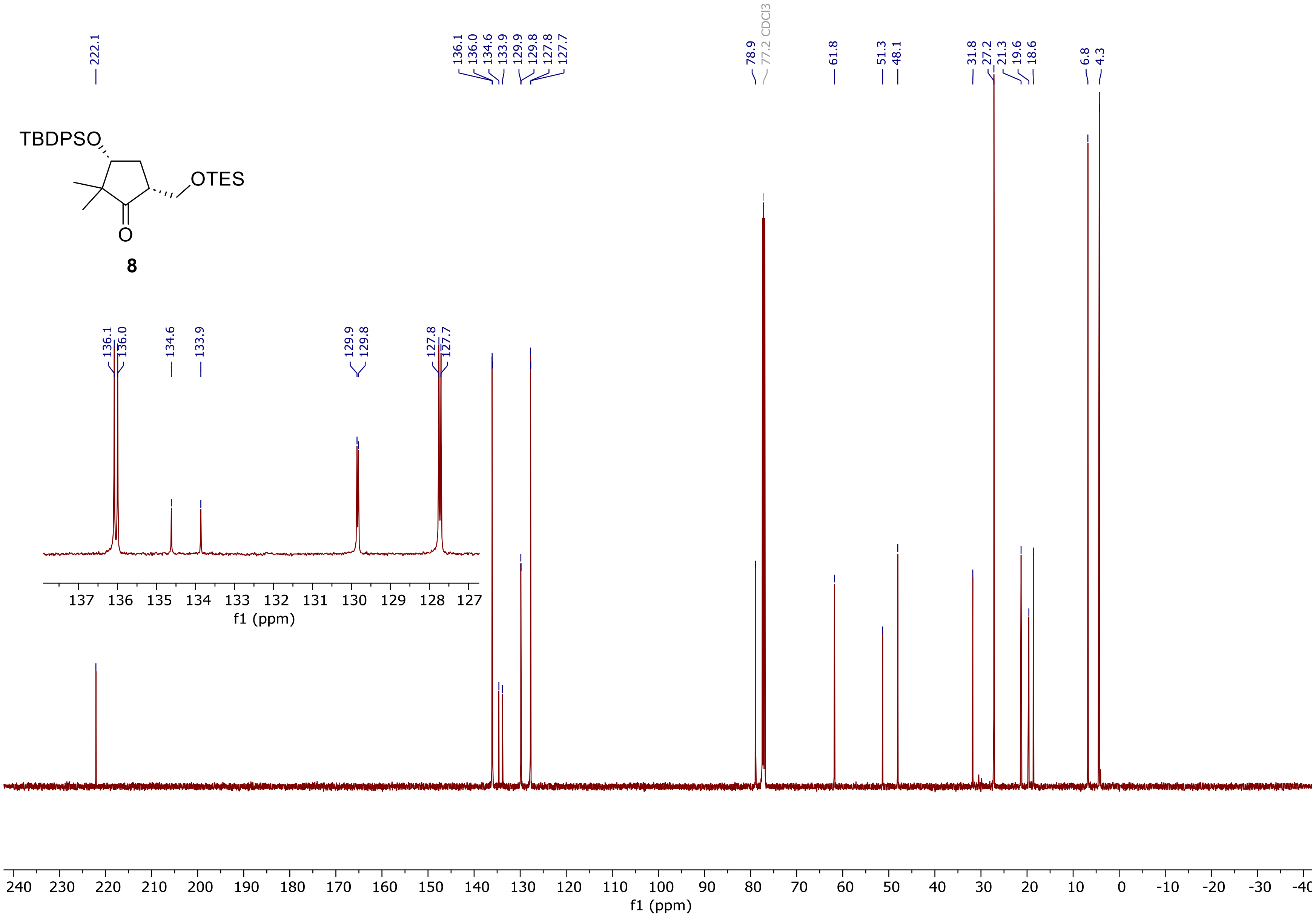

NOESY (500 MHz, $CDCl_3$)

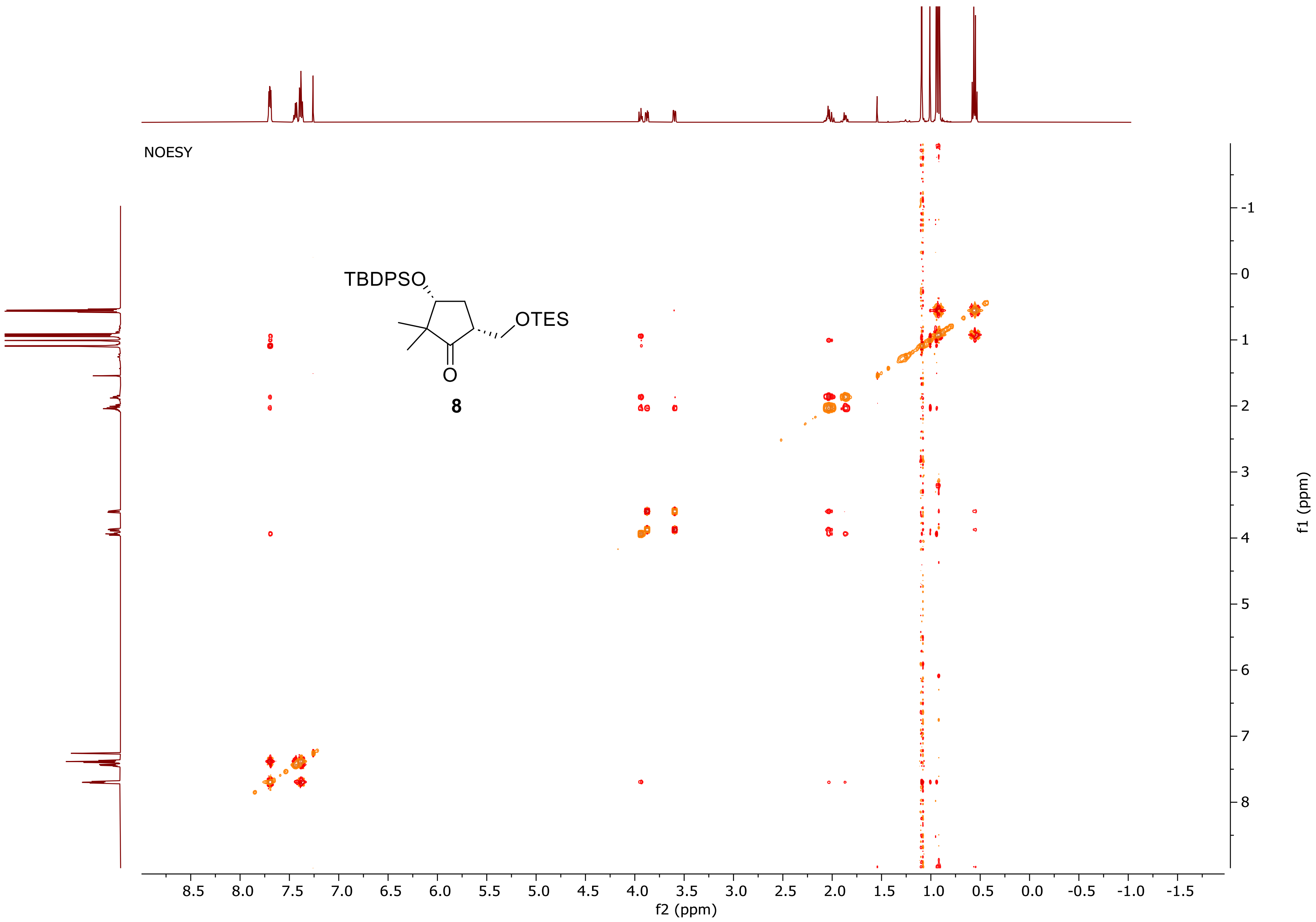

^{1}H NMR (400 MHz, $CDCl_3$)

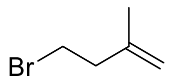

SI-4

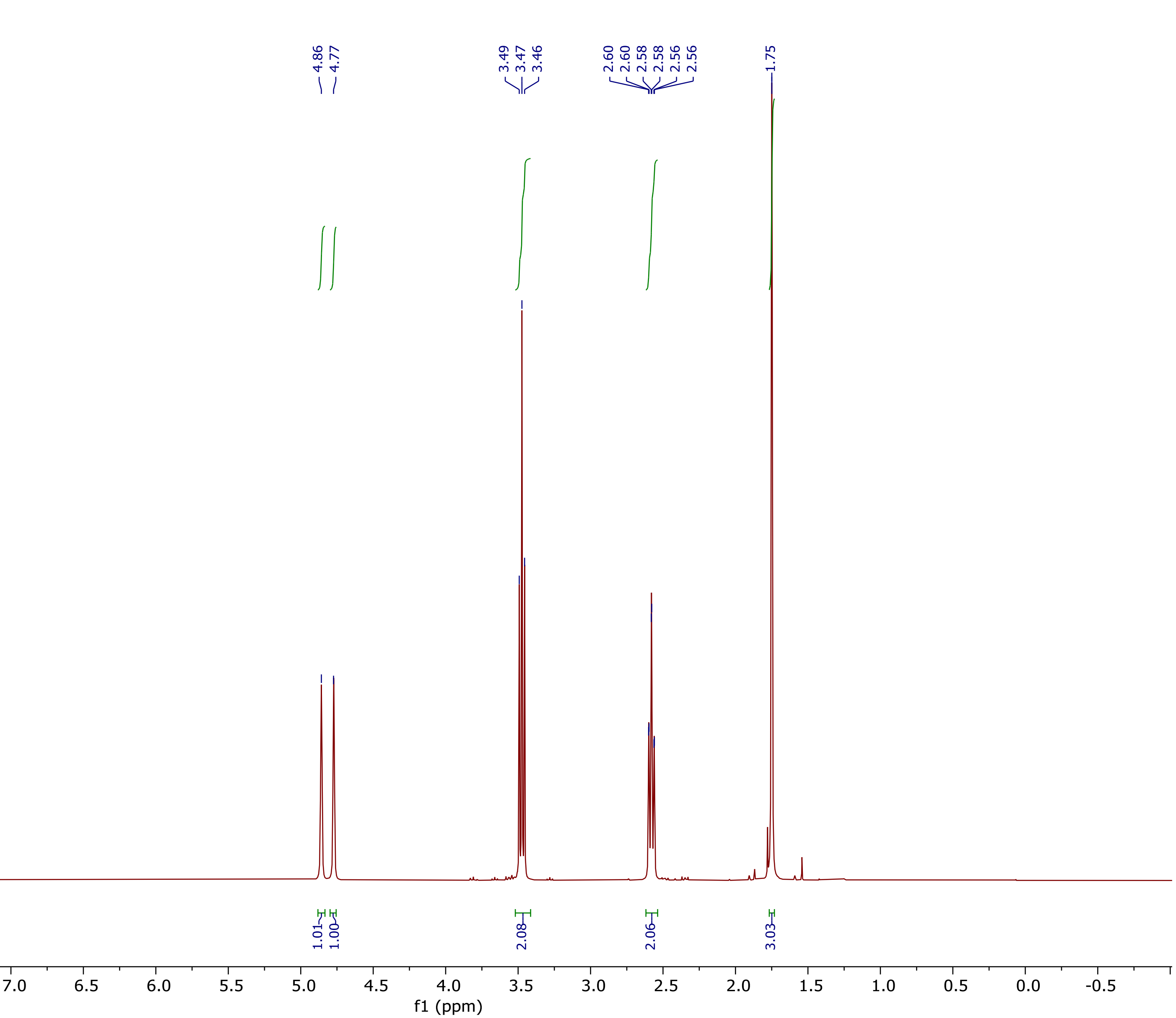

^{13}C NMR (101 MHz, $CDCl_3$)

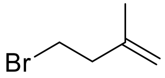

SI-4

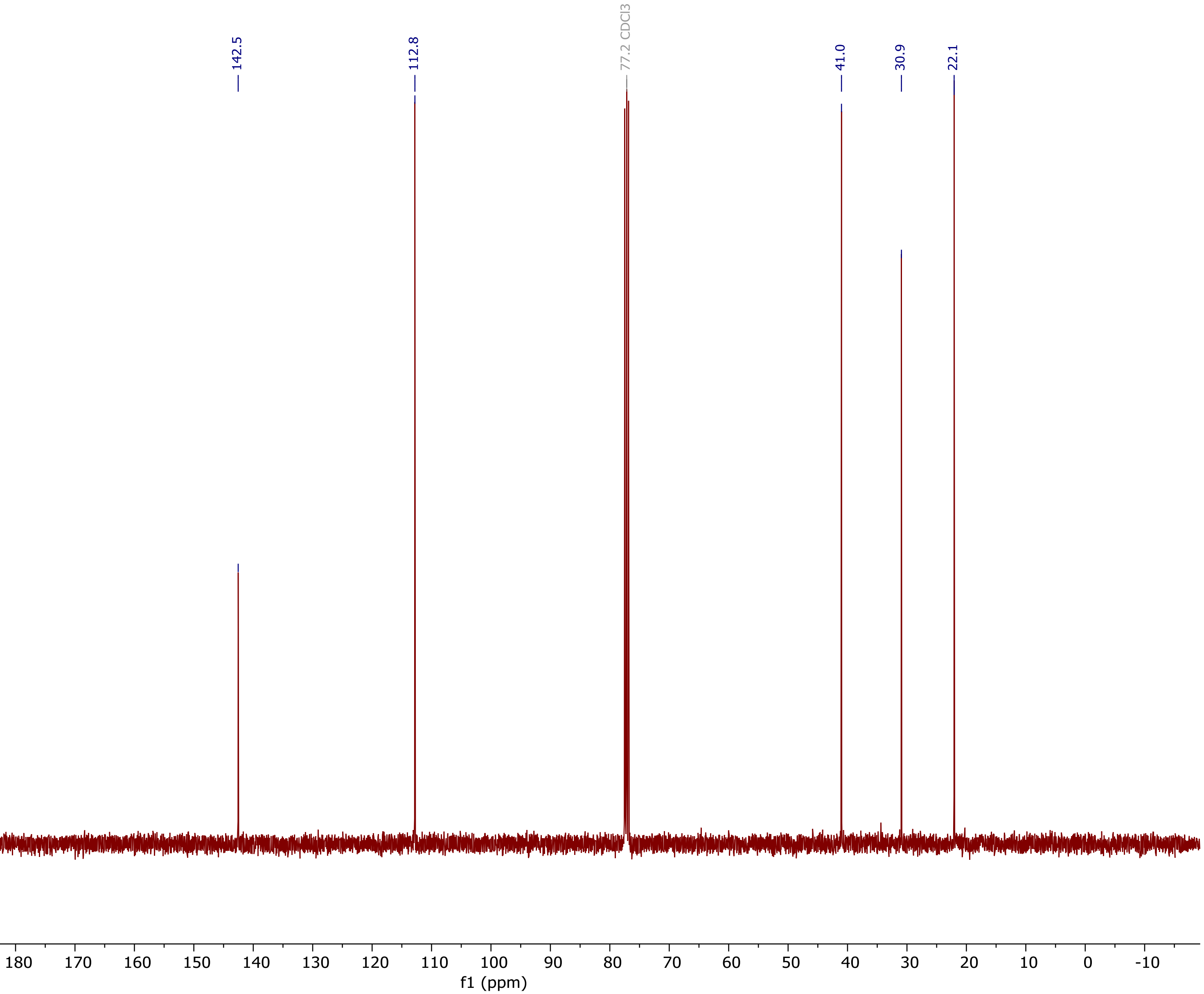

^{1}H NMR (500 MHz, $CDCl_3$)

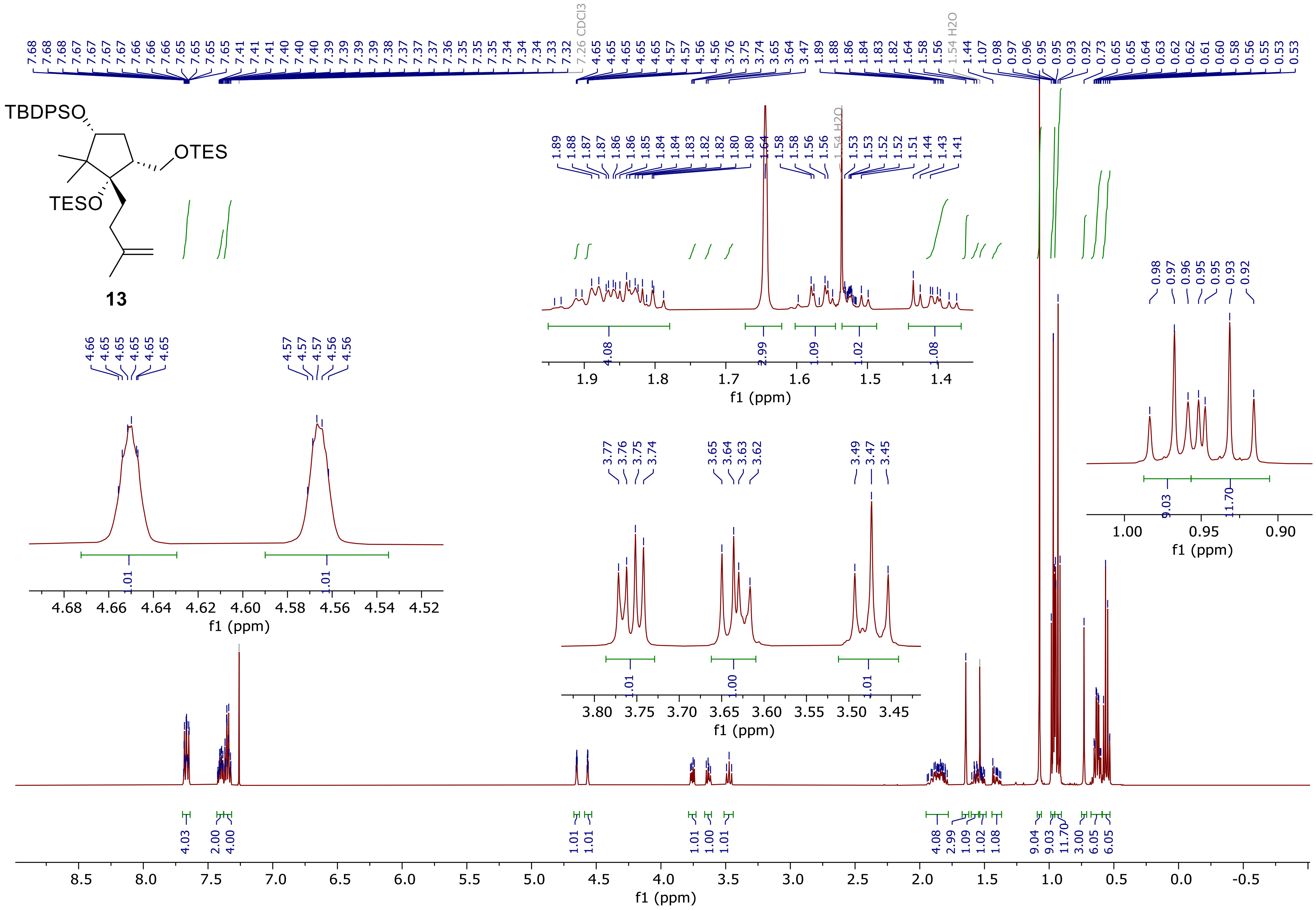

^{13}C NMR (126 MHz, $CDCl_3$)

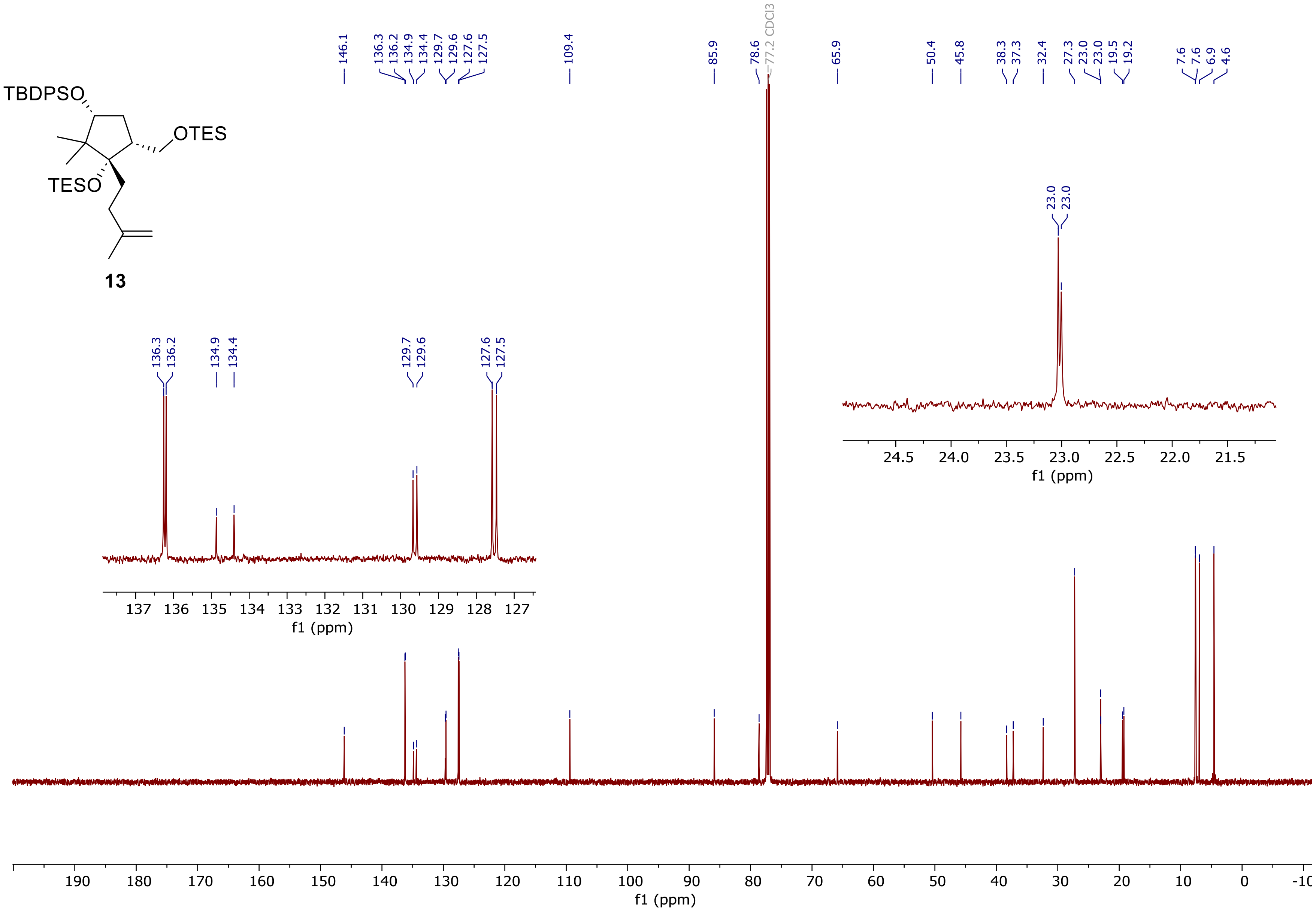

^{1}H NMR (500 MHz, $CDCl_3$)

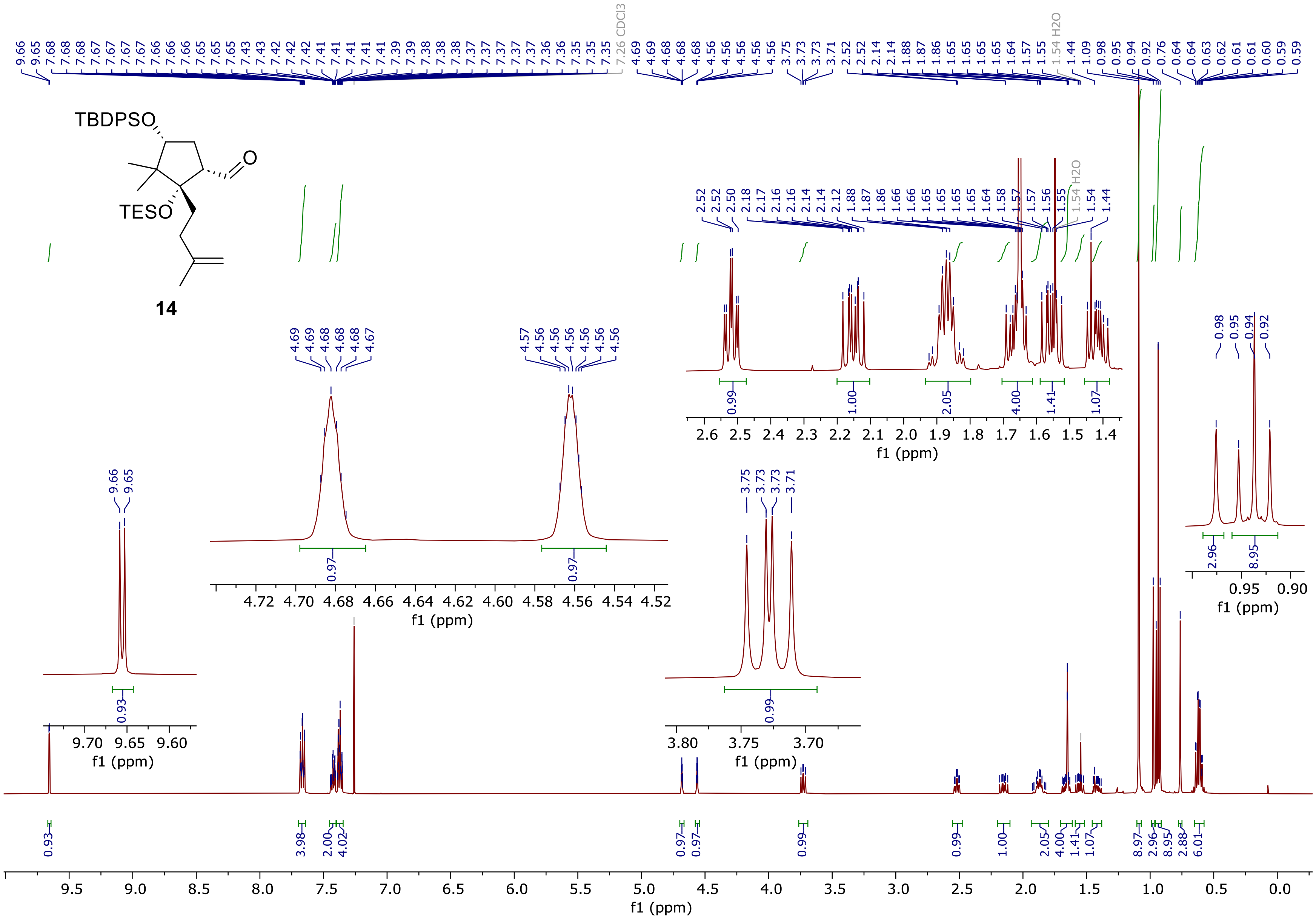

^{13}C NMR (126 MHz, $CDCl_3$)

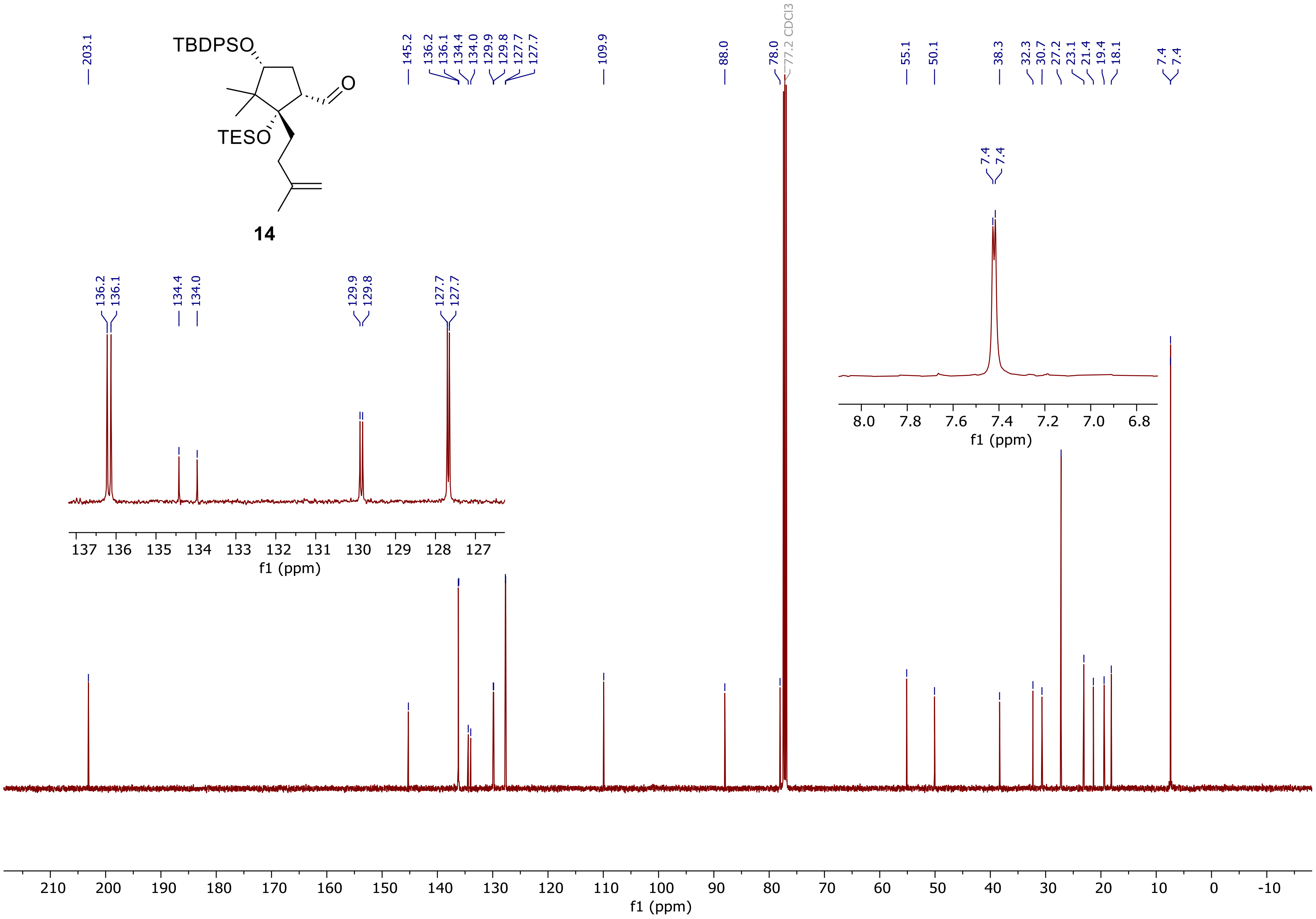

COSY (500 MHz, $CDCl_3$)

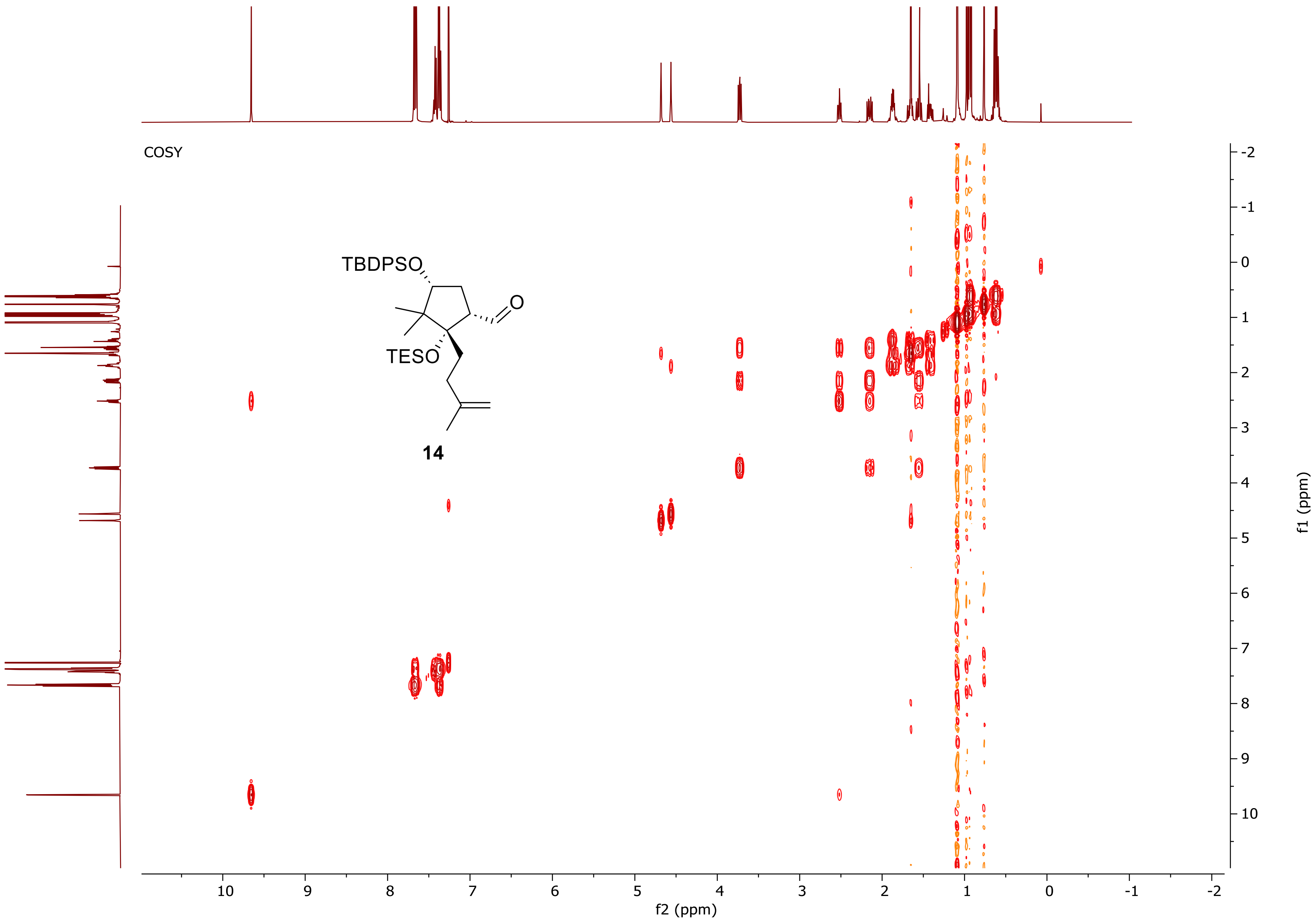

NOESY (500 MHz, $CDCl_3$)

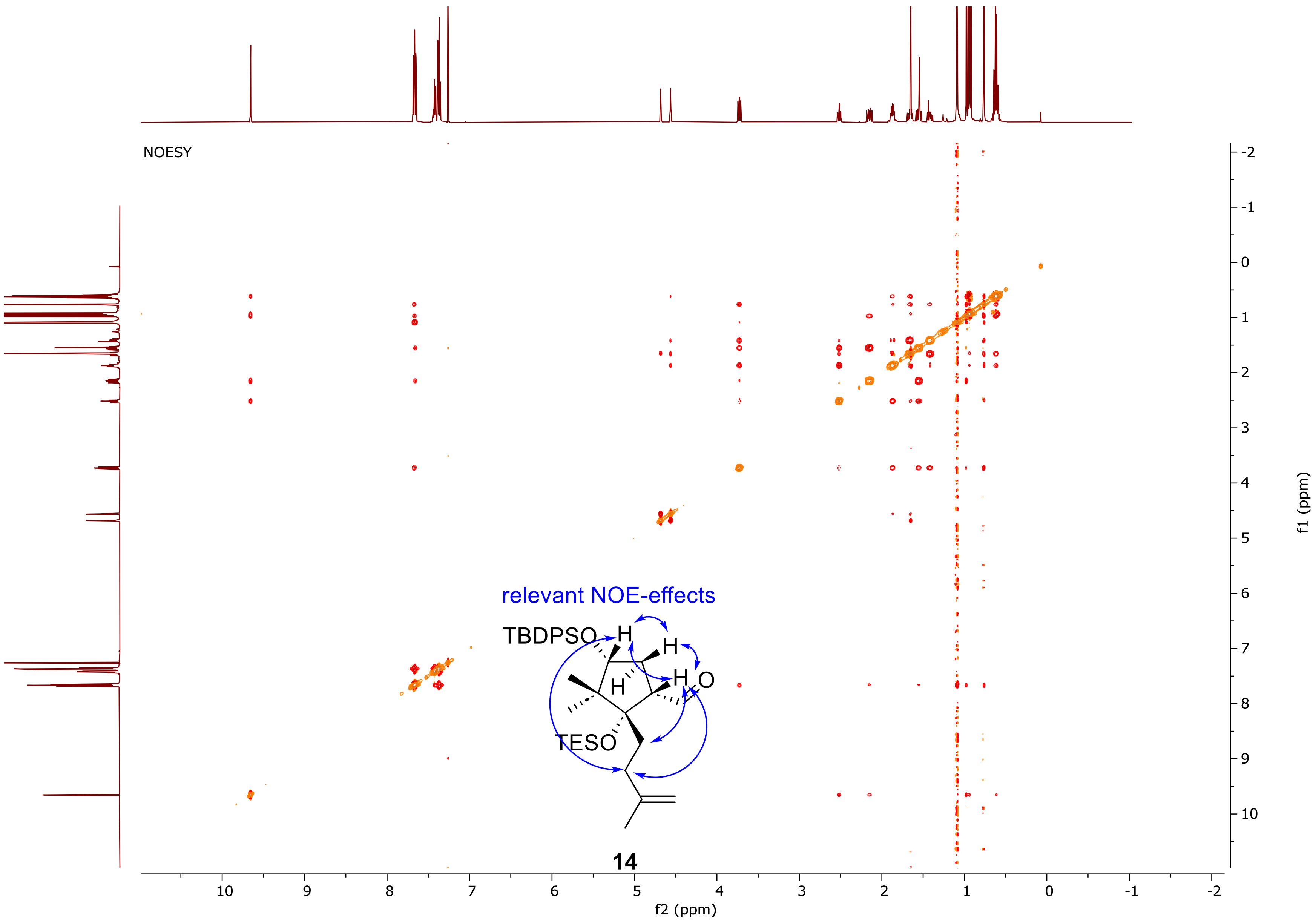

^1H NMR (400 MHz, $CDCl_3$)

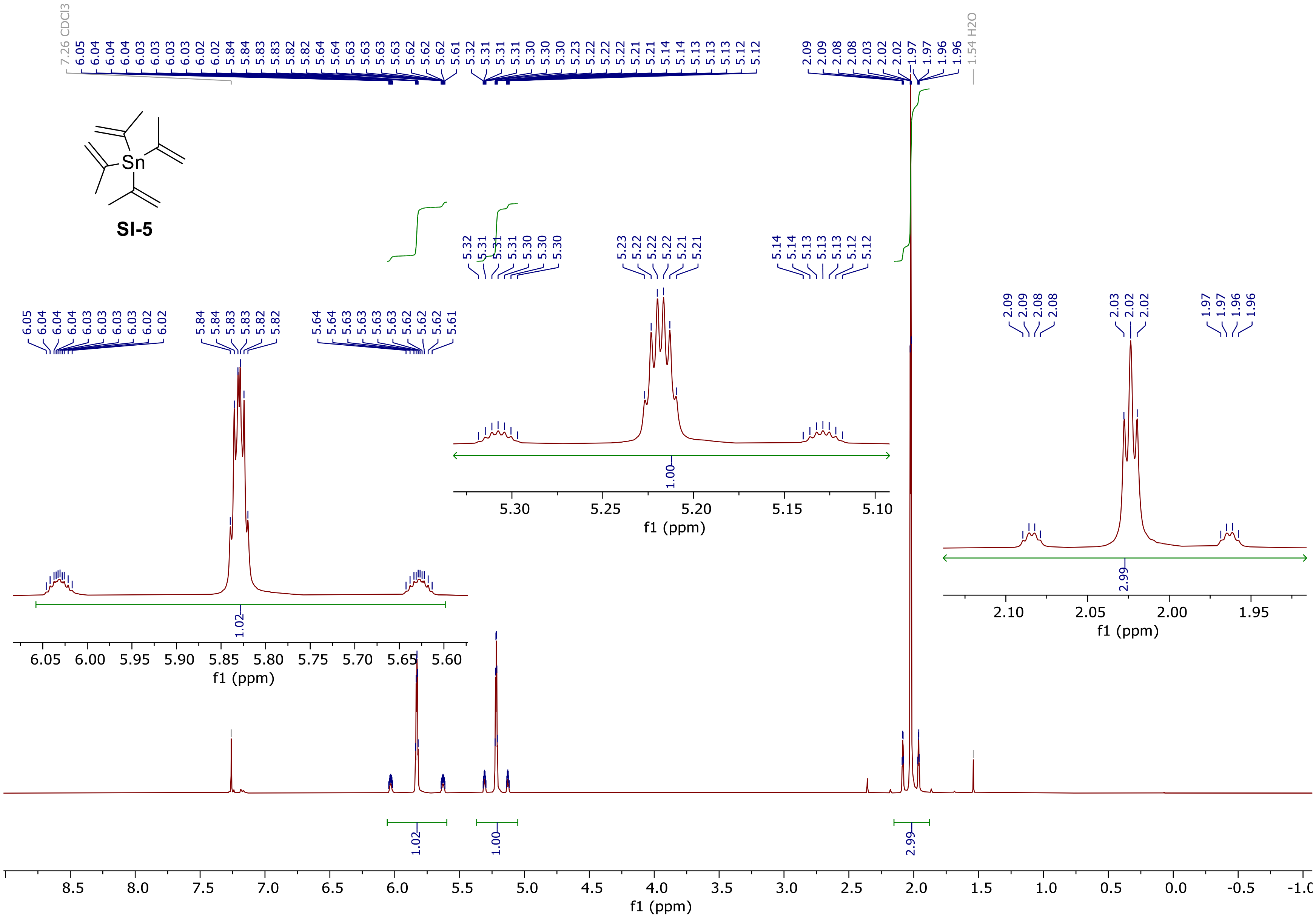

SI-5

^{13}C NMR (101 MHz, $CDCl_3$)

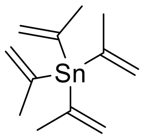

SI-5

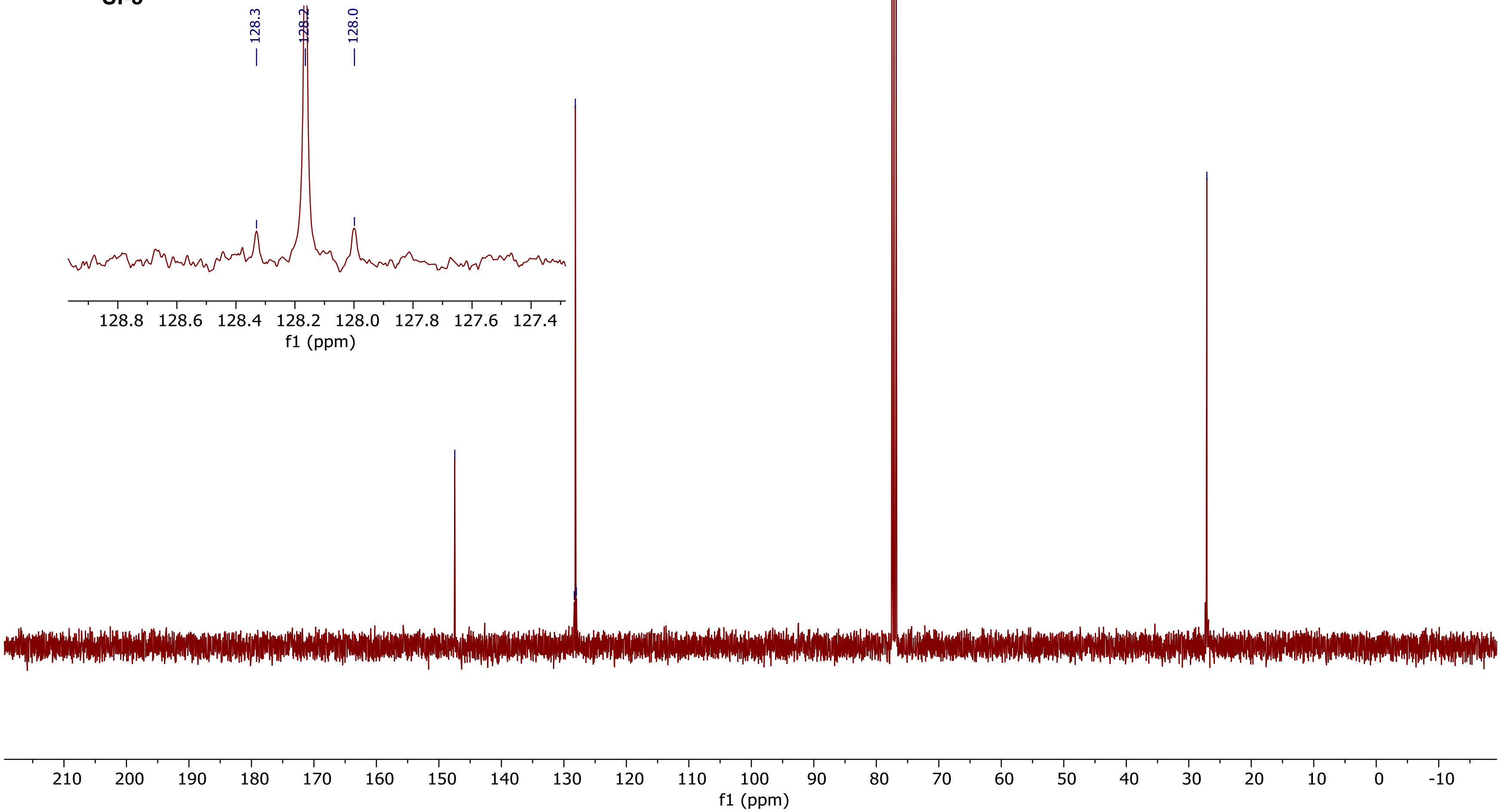

^1H NMR (500 MHz, $CDCl_3$)

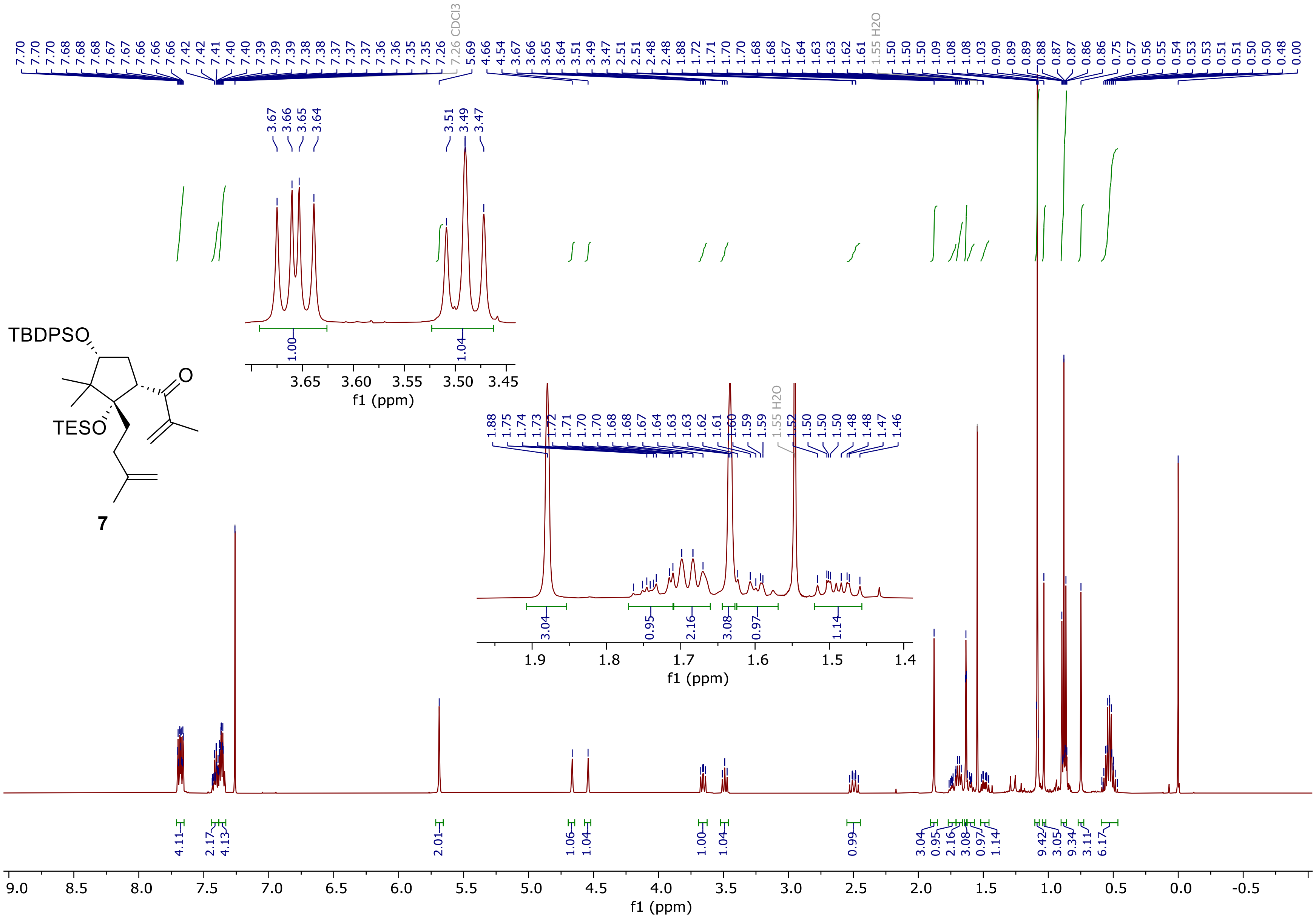

^{13}C NMR (126 MHz, $CDCl_3$)

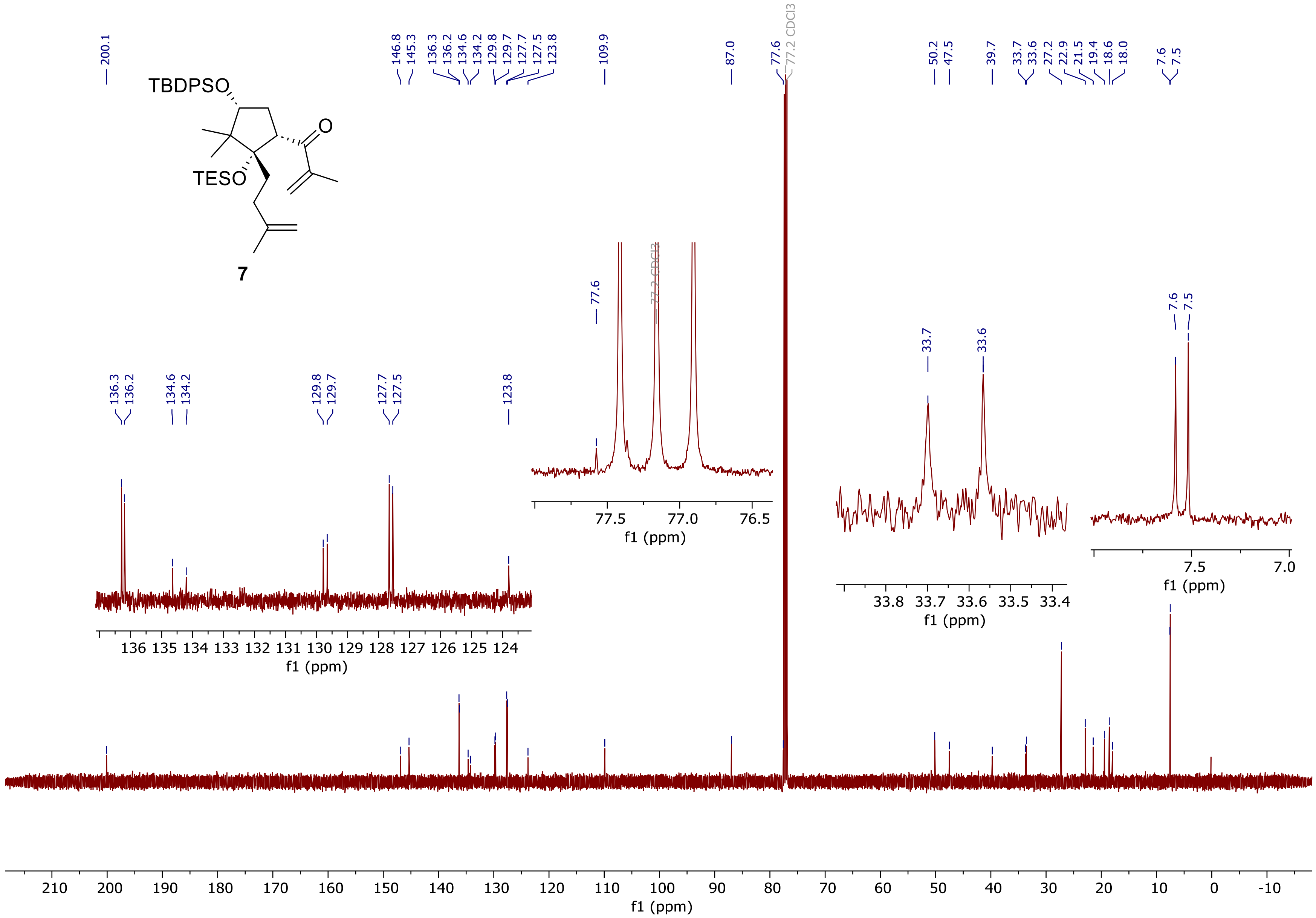

^{1}H NMR (500 MHz, $CDCl_3$)

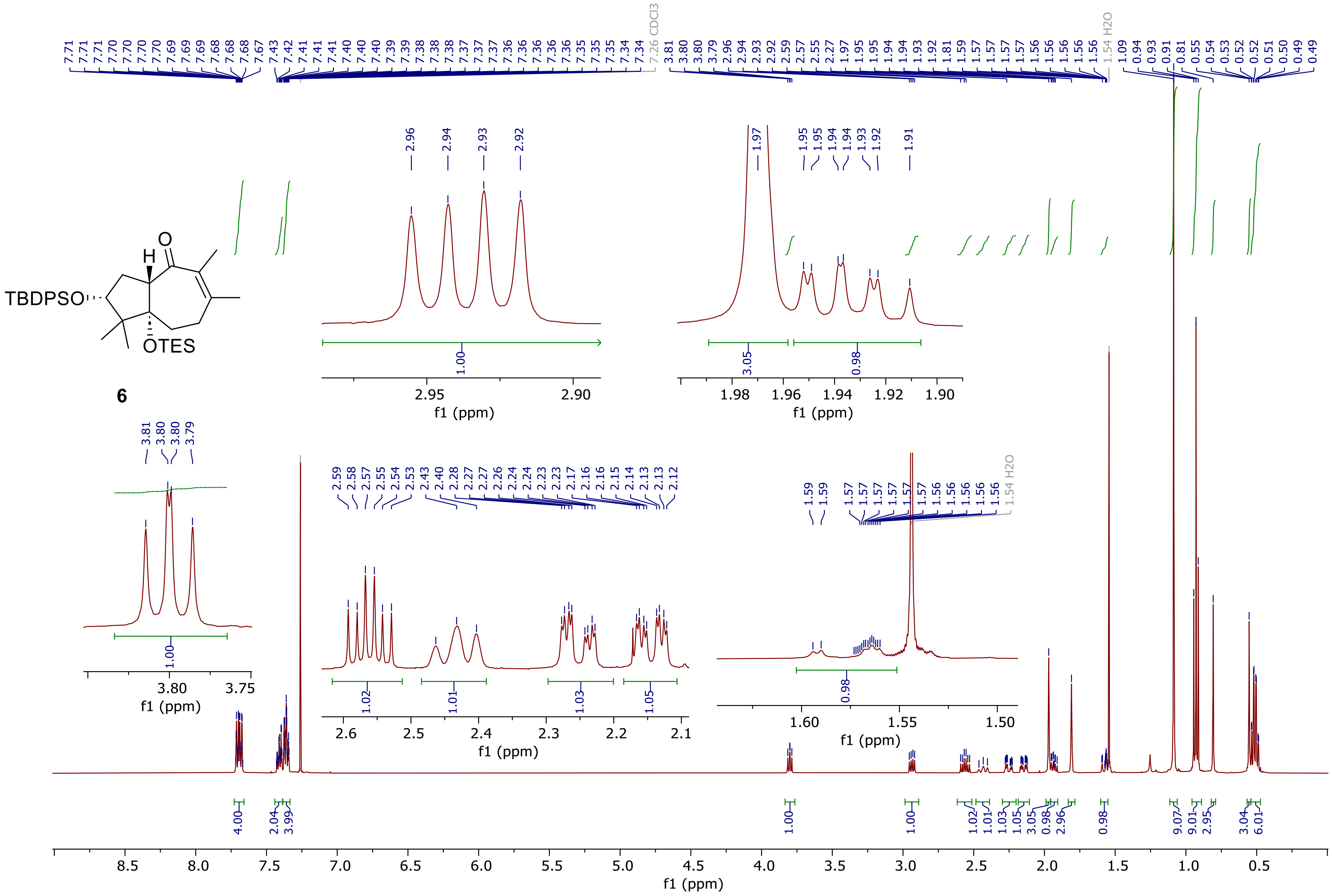

6

^{13}C NMR (126 MHz, $CDCl_3$)

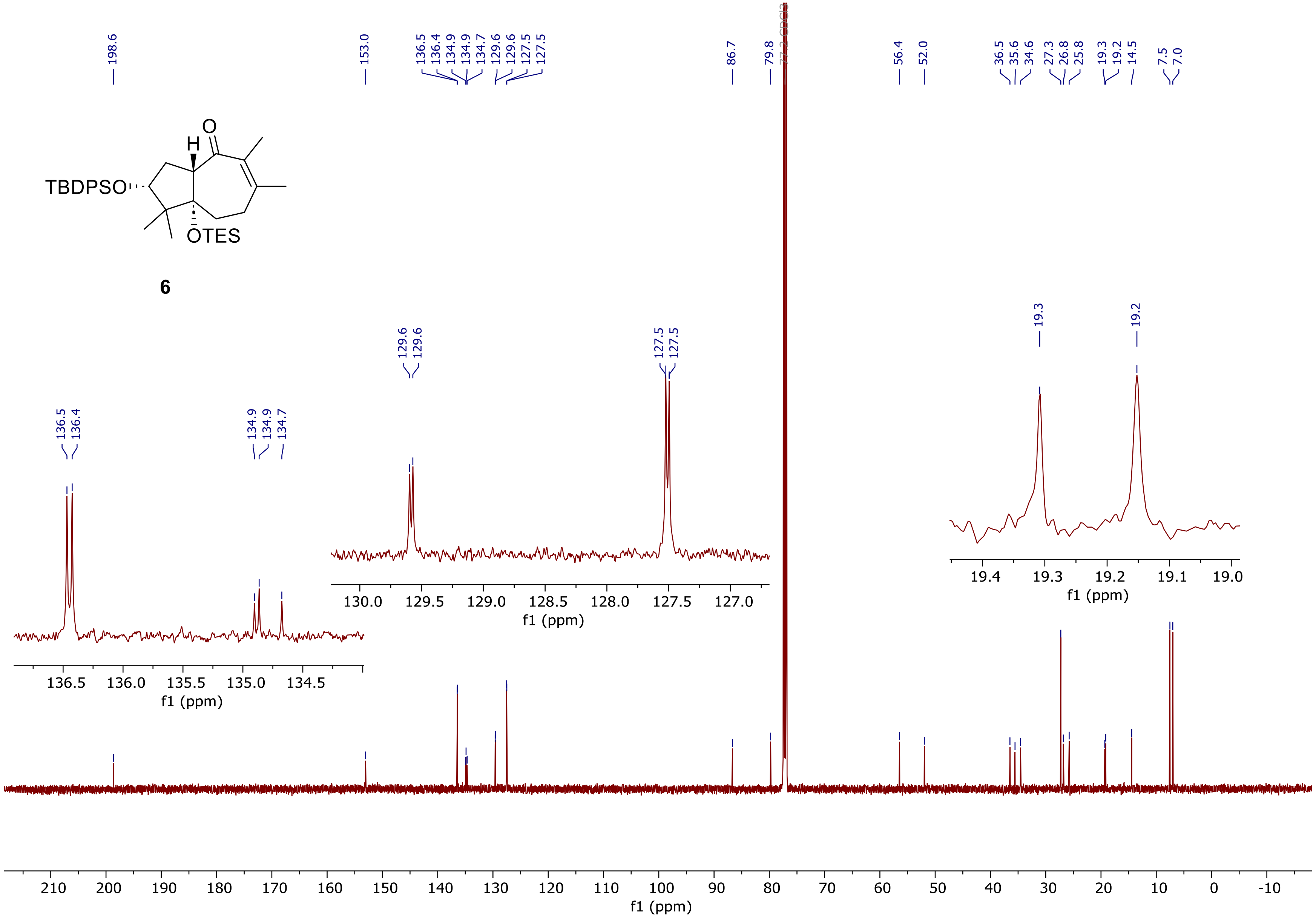

^{1}H NMR (500 MHz, $CDCl_3$)

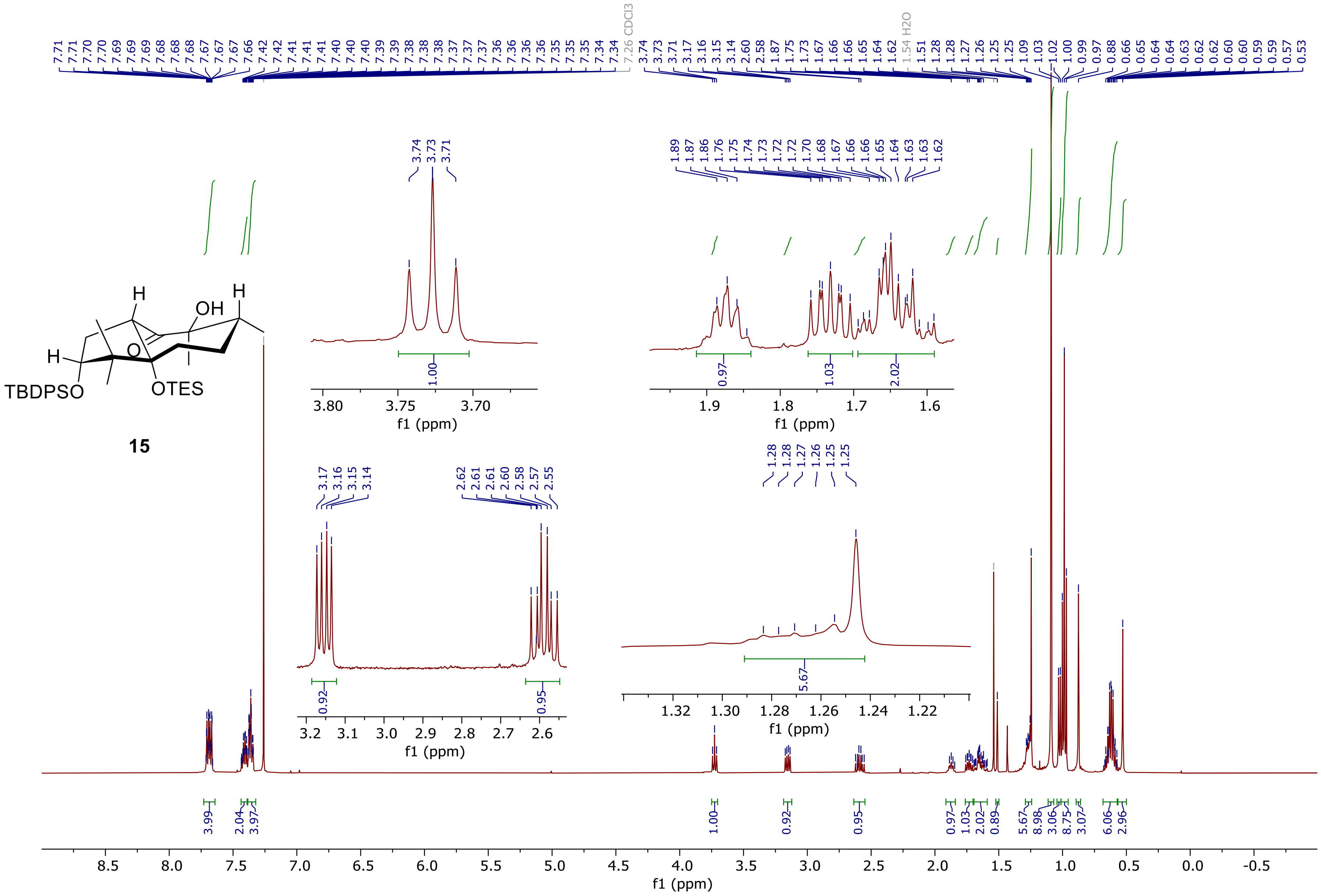

^{13}C NMR (126 MHz, $CDCl_3$)

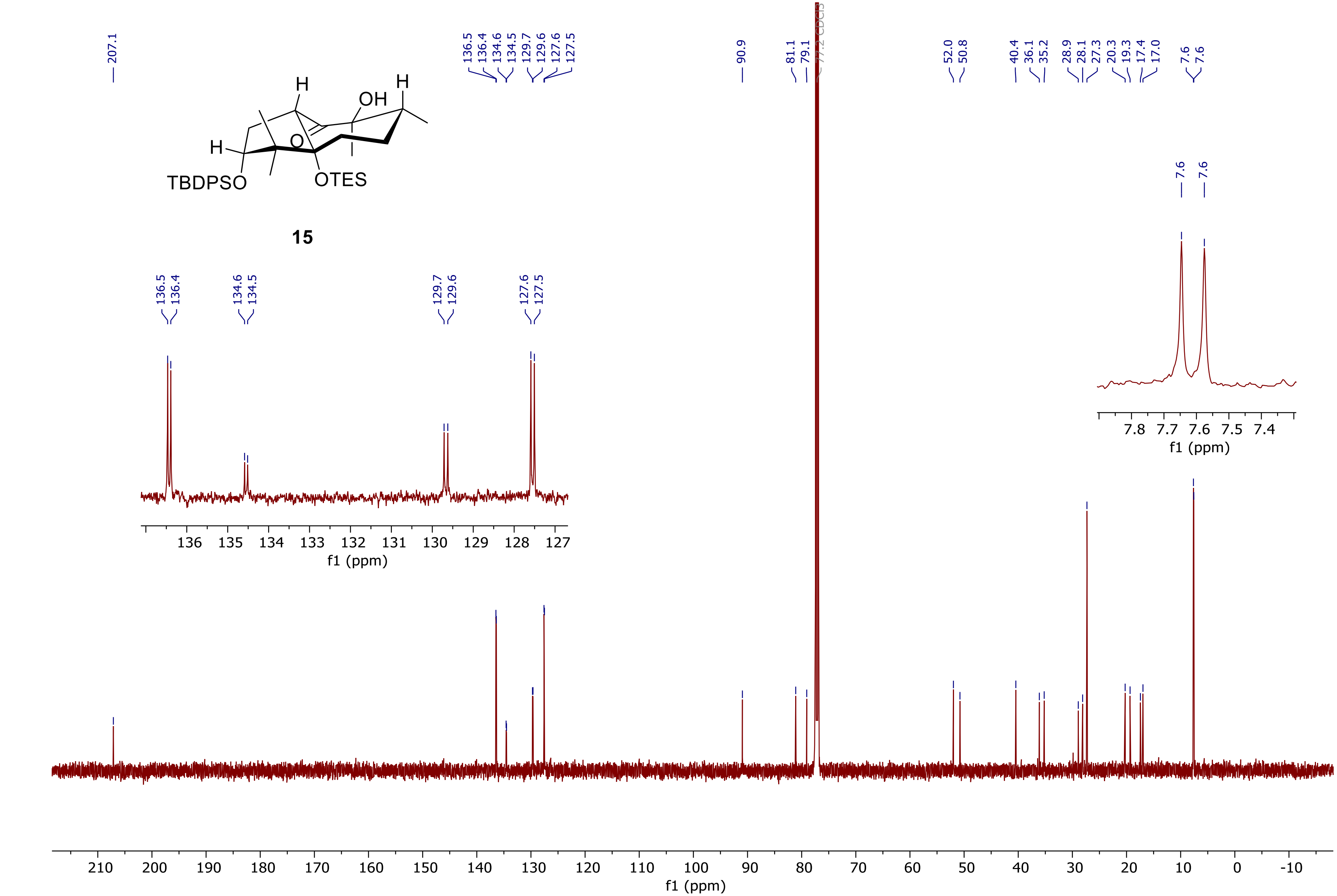

^{1}H NMR (500 MHz, $CDCl_3$)

SI-6

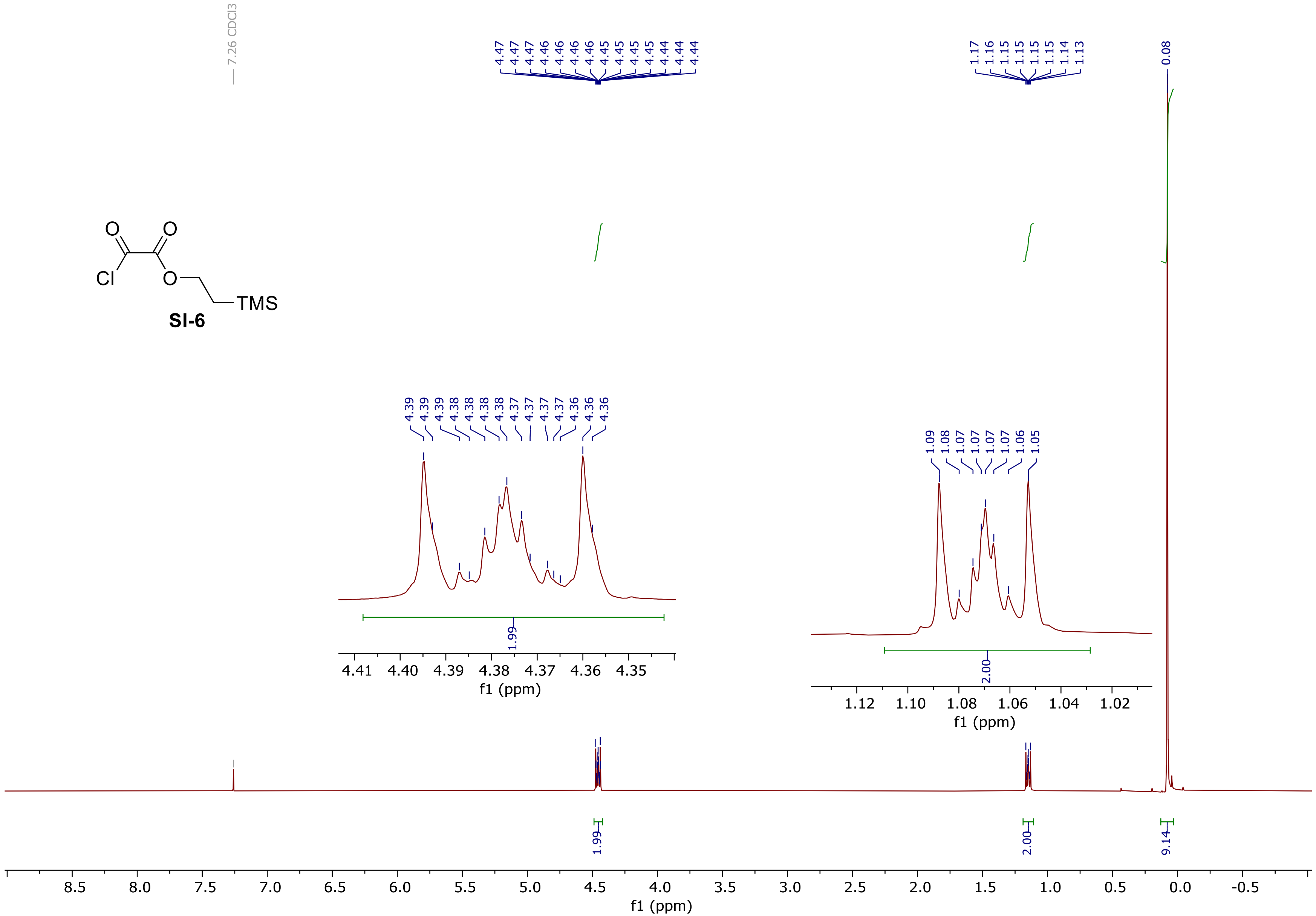

^{13}C NMR (126 MHz, $CDCl_3$)

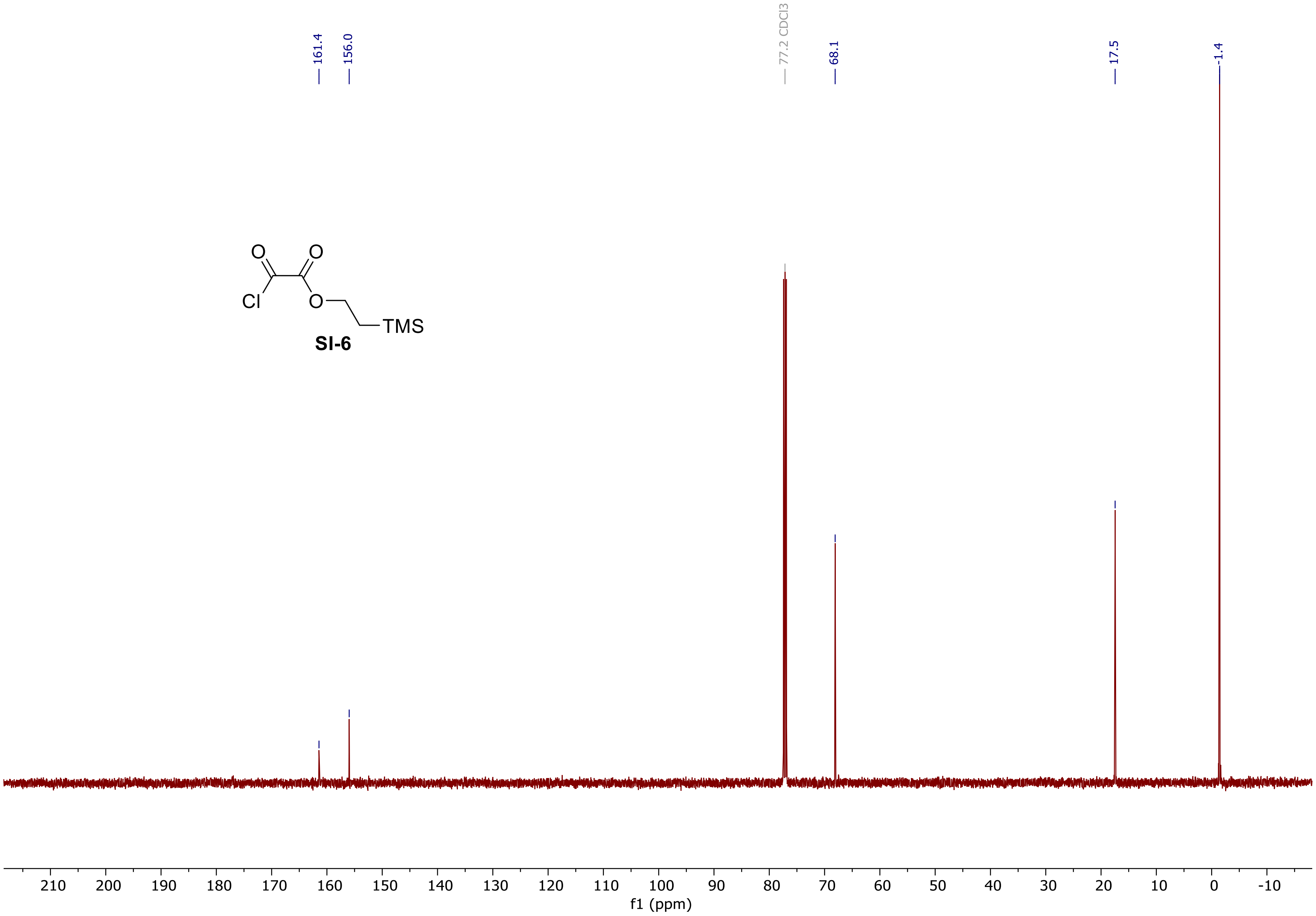

^1H NMR (500 MHz, $CDCl_3$)

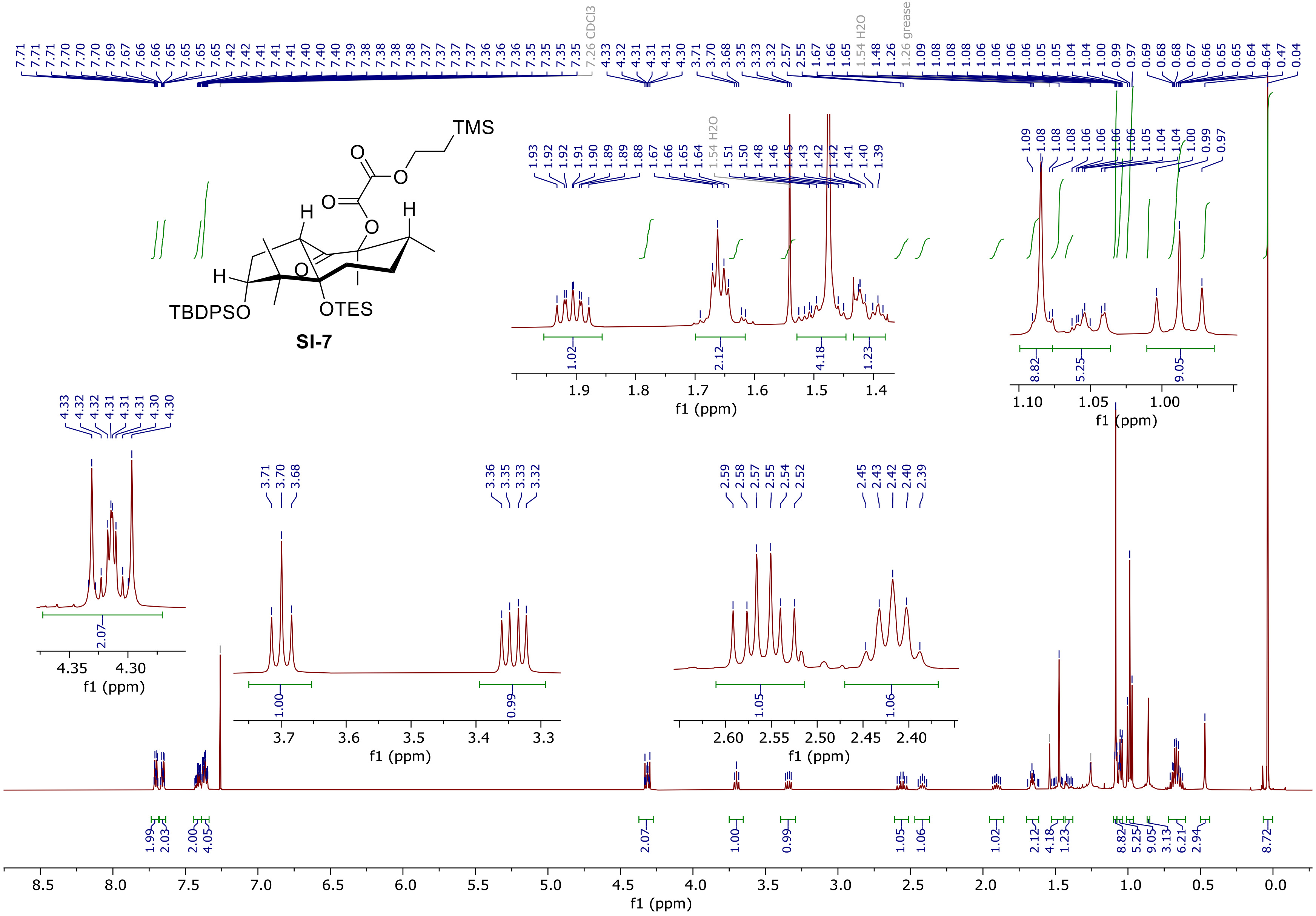

^{13}C NMR (126 MHz, $CDCl_3$)

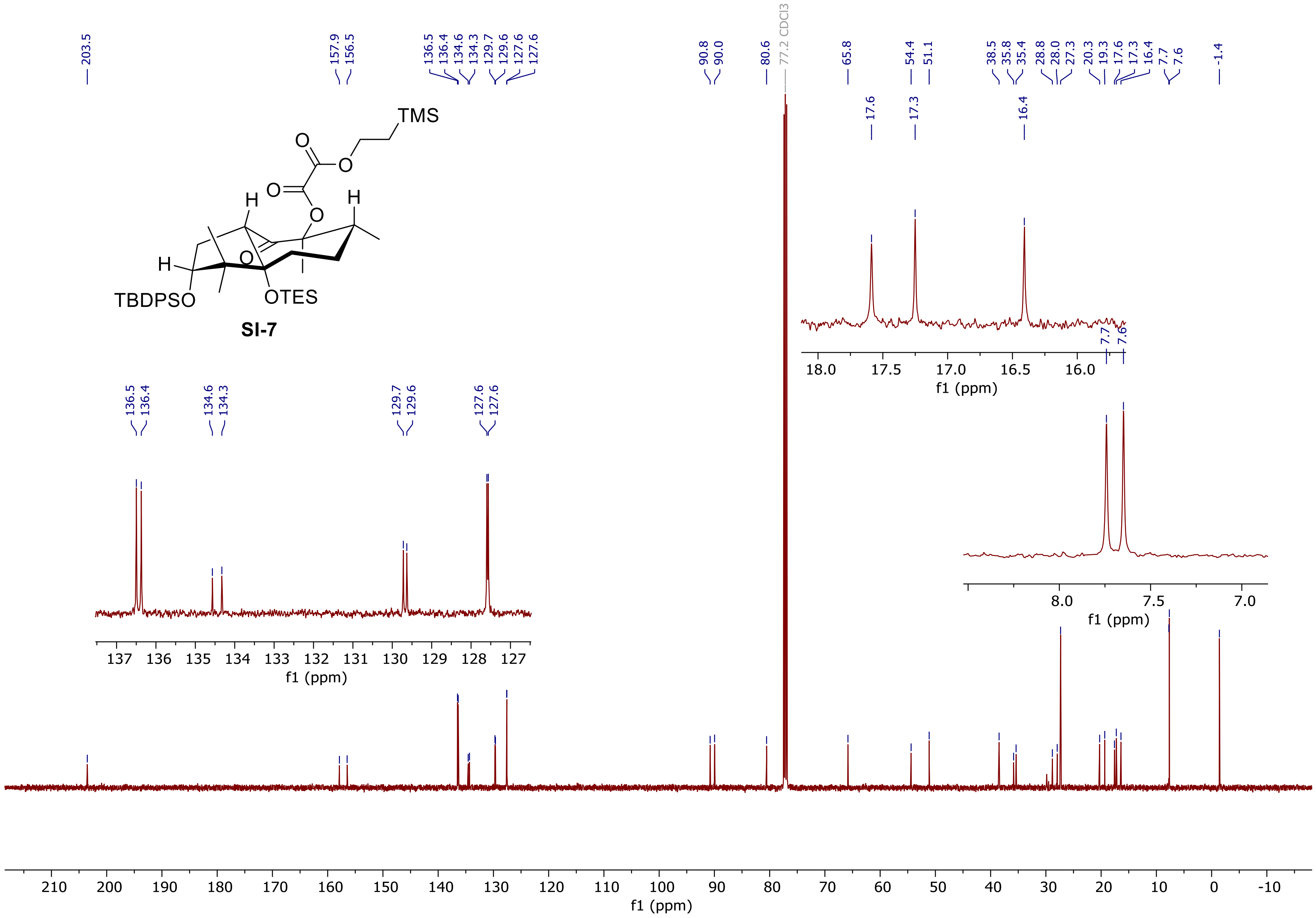

COSY (500 MHz, $CDCl_3$)

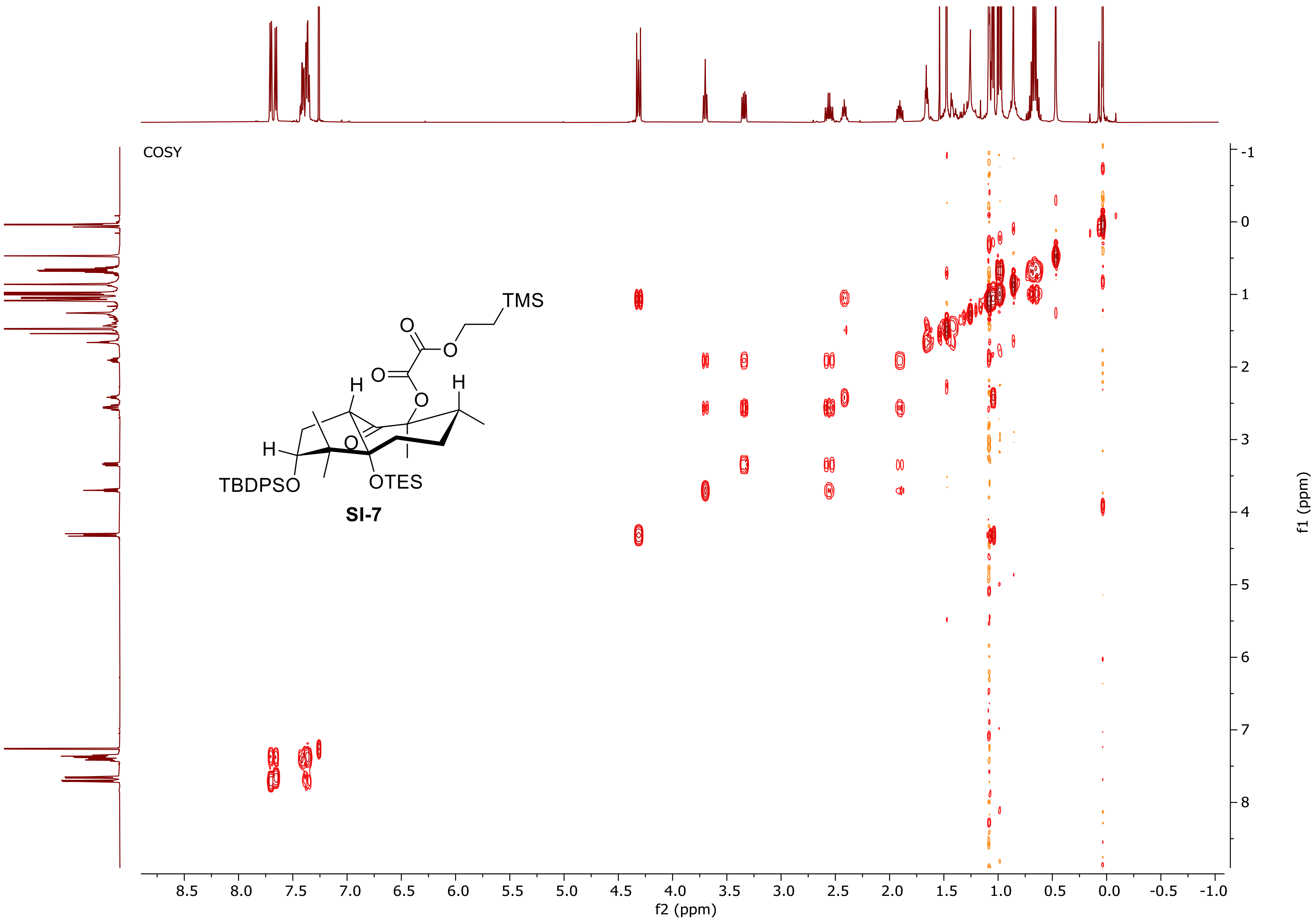

NOESY (500 MHz, $CDCl_3$)

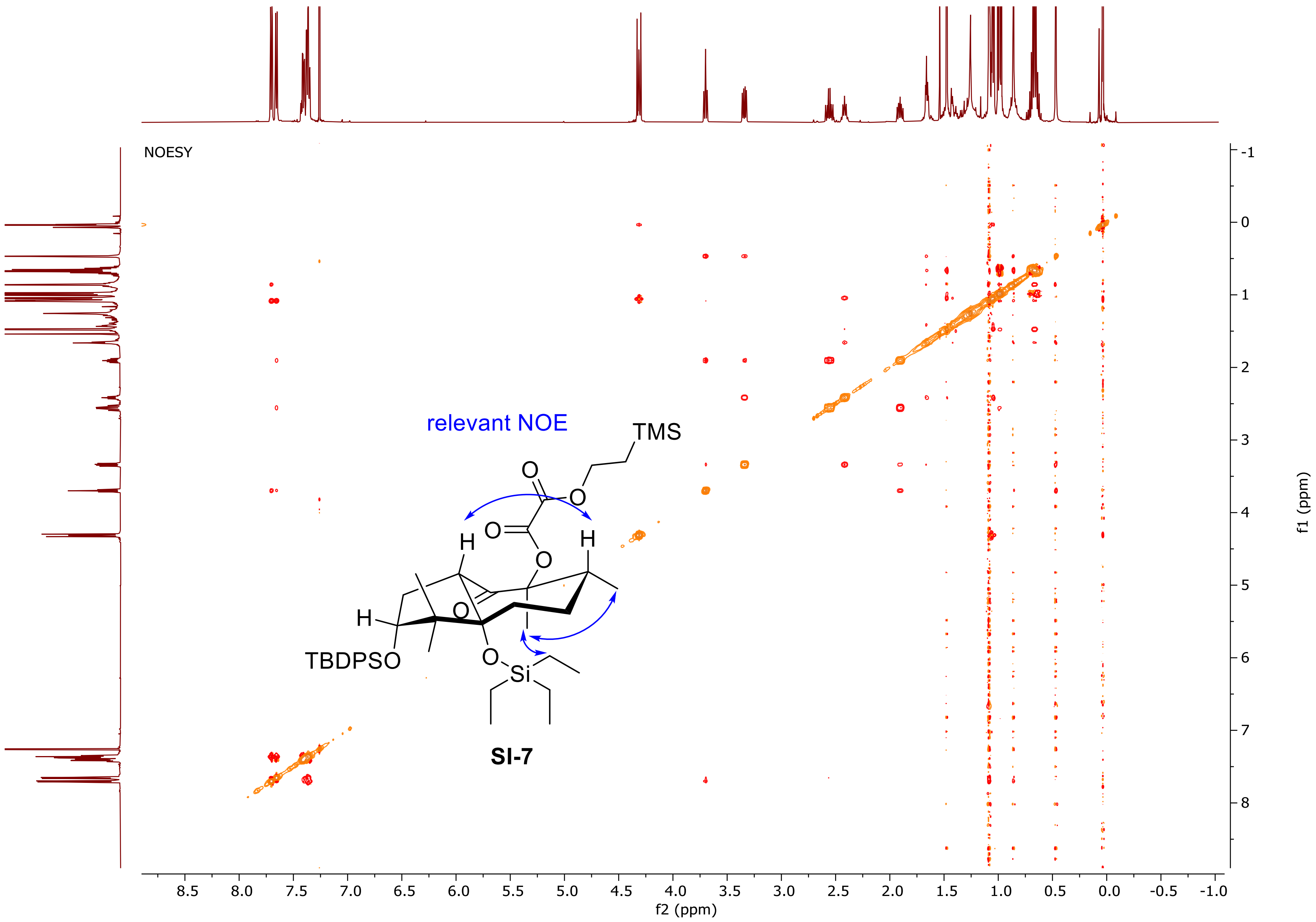

^{1}H NMR (400 MHz, $CDCl_3$)

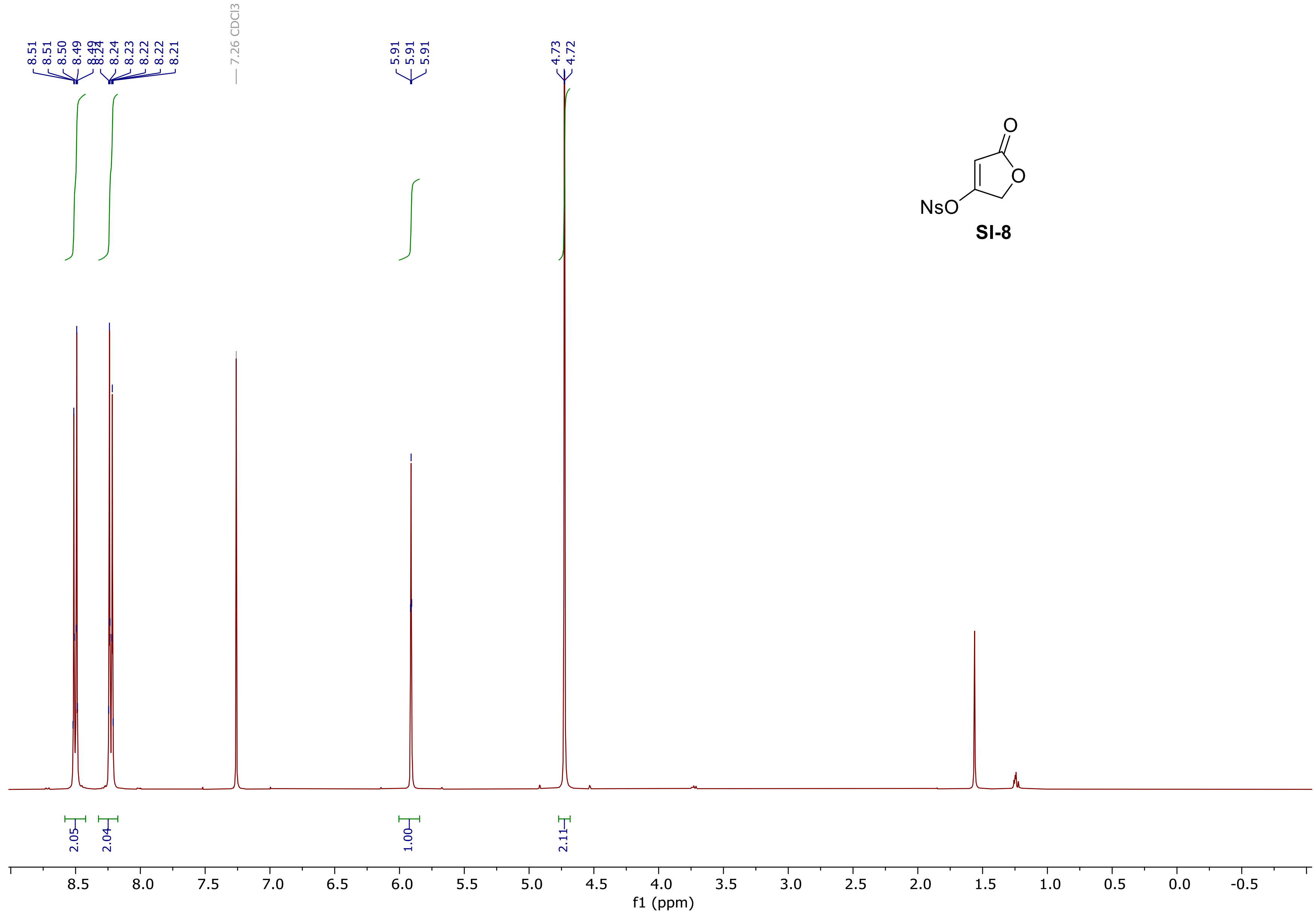

^{13}C NMR (101 MHz, $CDCl_3$)

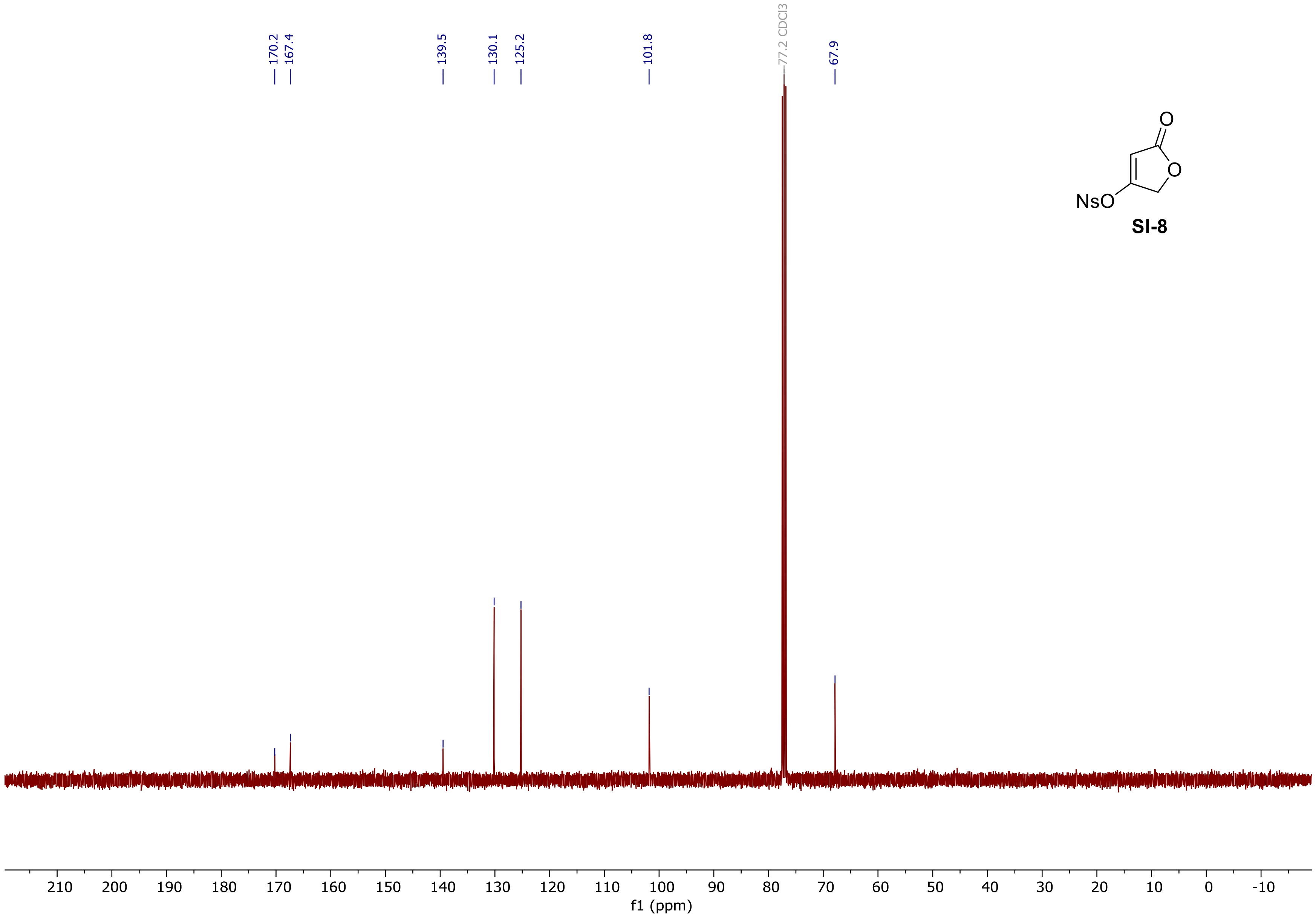

SI-8

^{1}H NMR (500 MHz, $CDCl_3$)

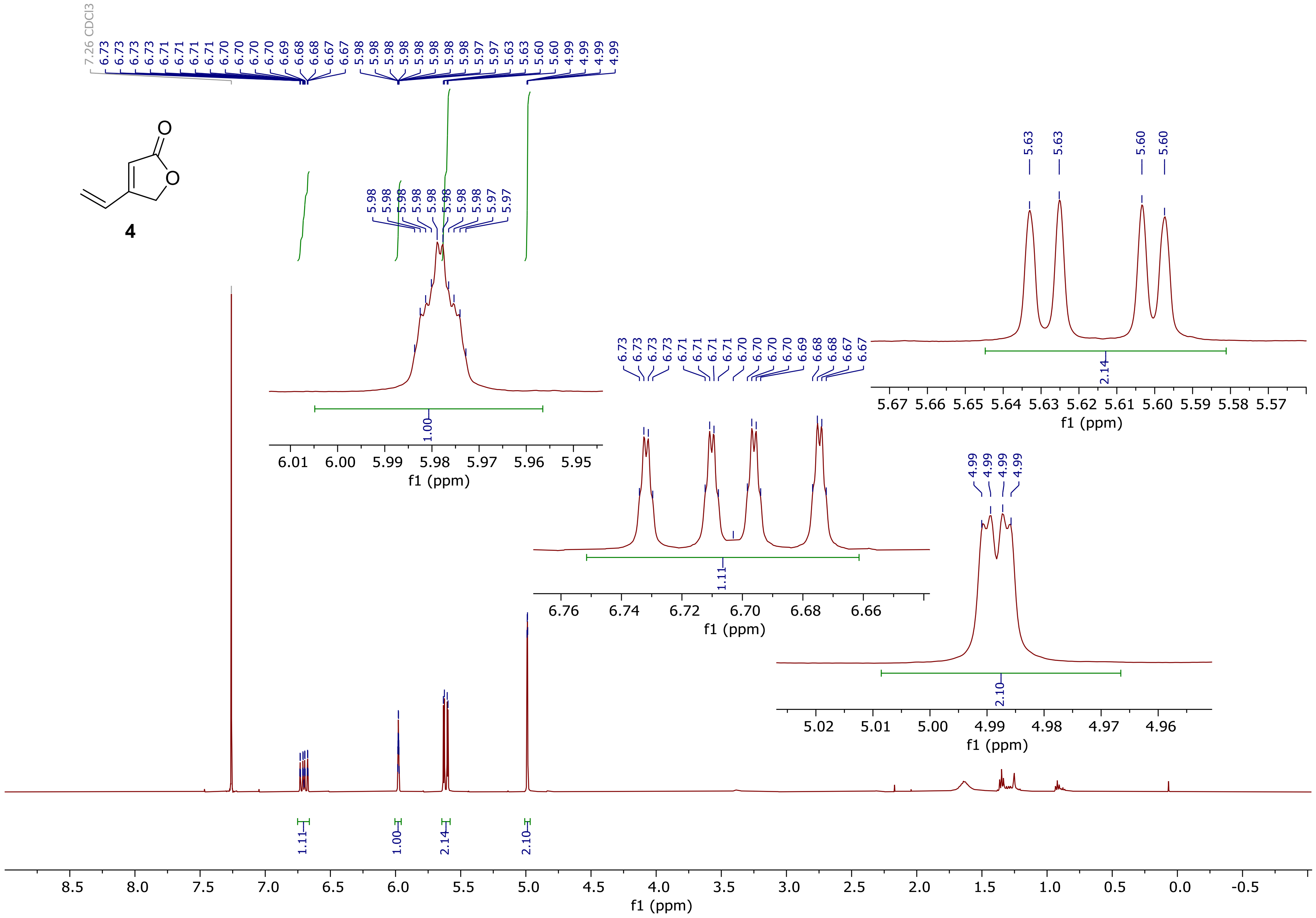

^{13}C NMR (126 MHz, $CDCl_3$)

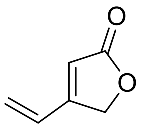

4

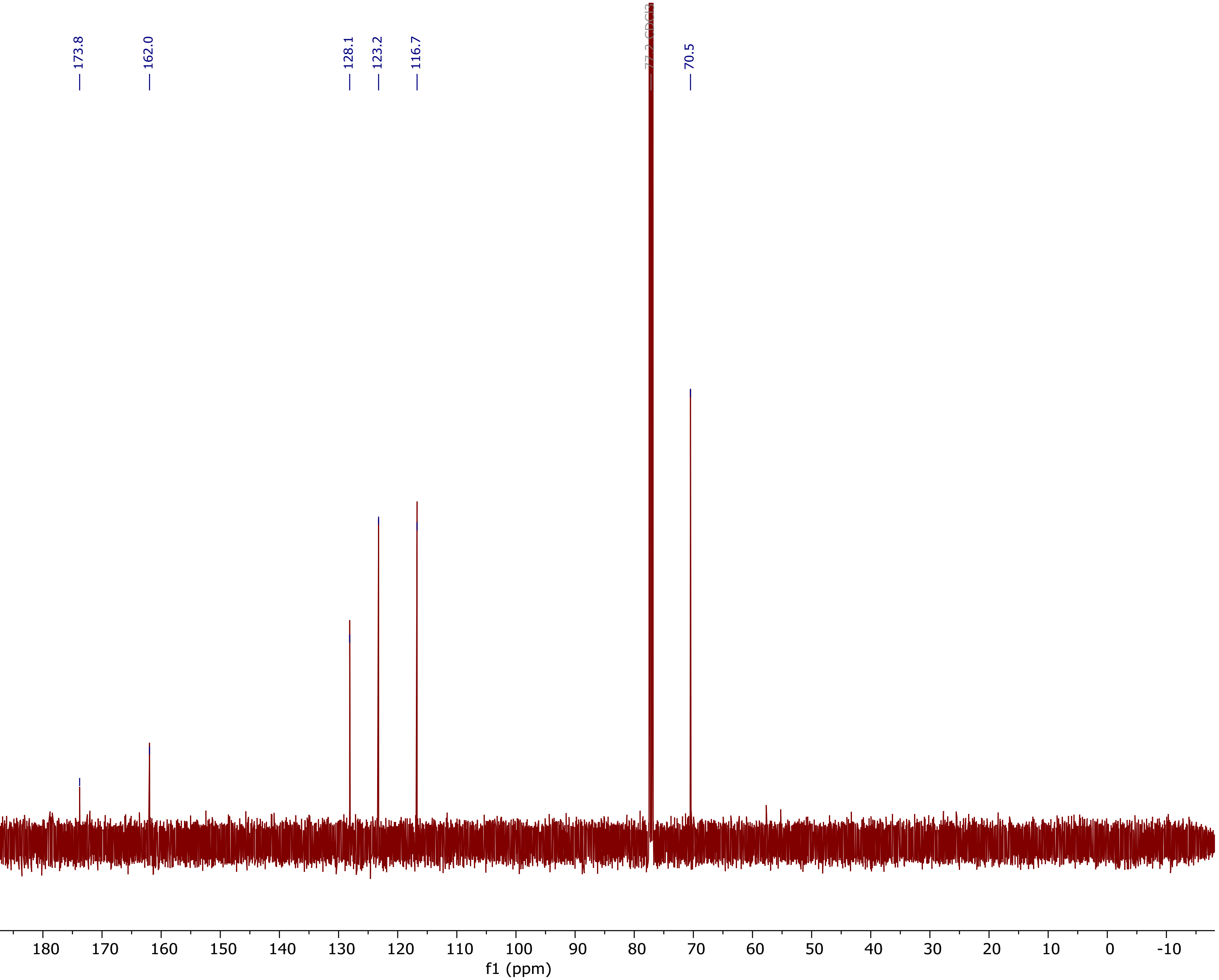

^{1}H NMR (500 MHz, CDCl$_3$)

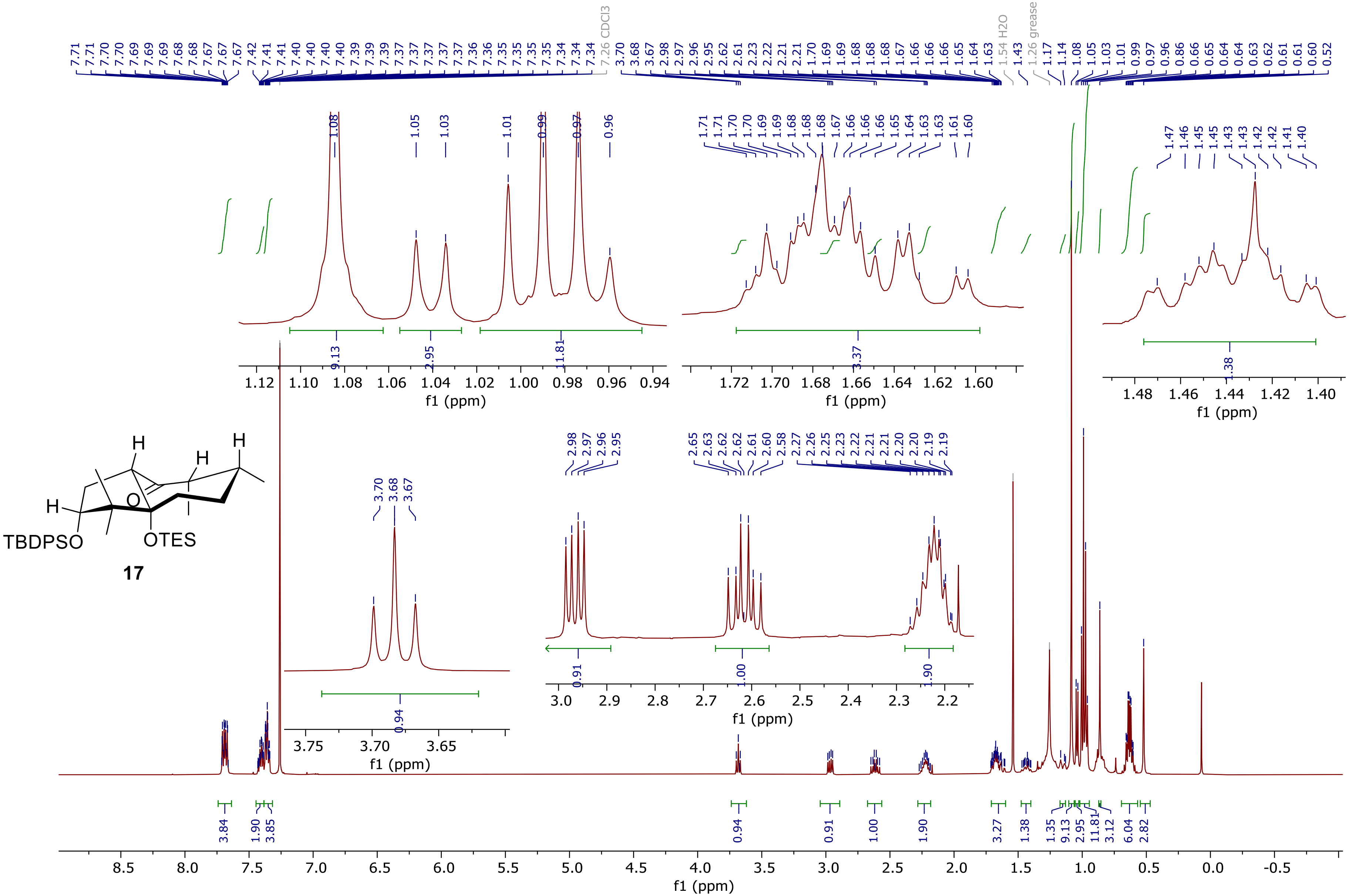

^{13}C NMR (126 MHz, $CDCl_3$)

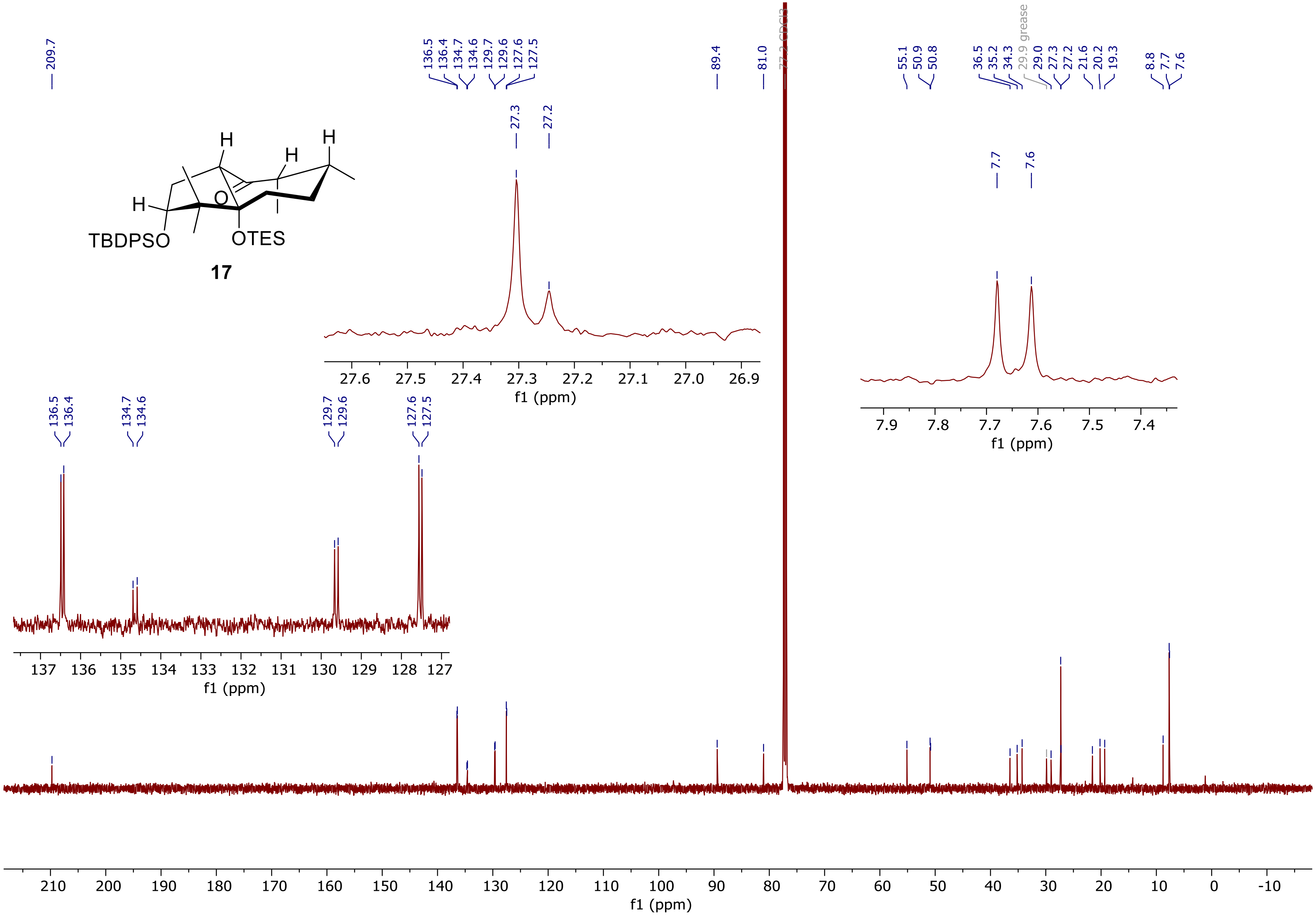

NOESY (500 MHz, $CDCl_3$)

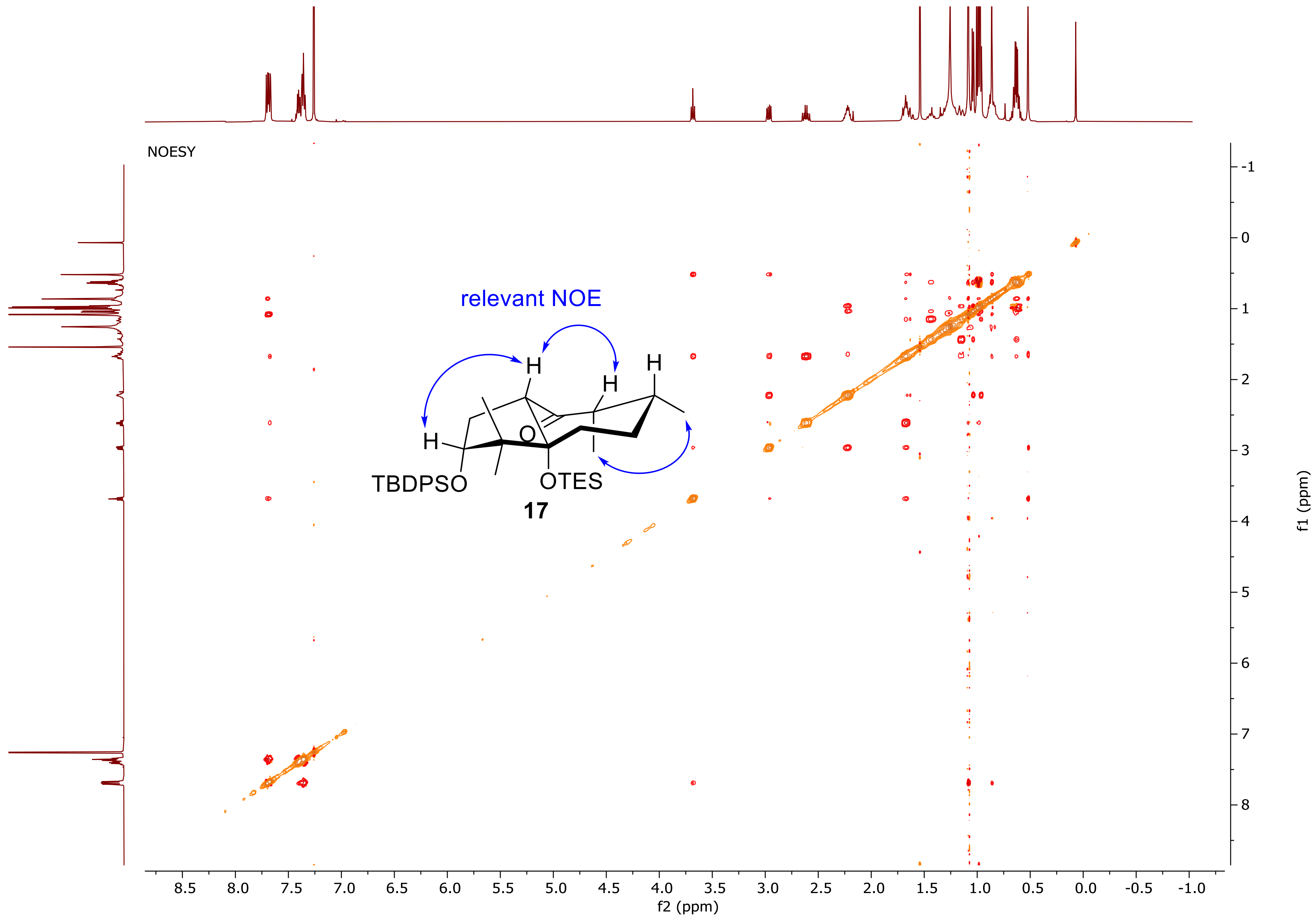

^{1}H NMR (500 MHz, $CDCl_3$)

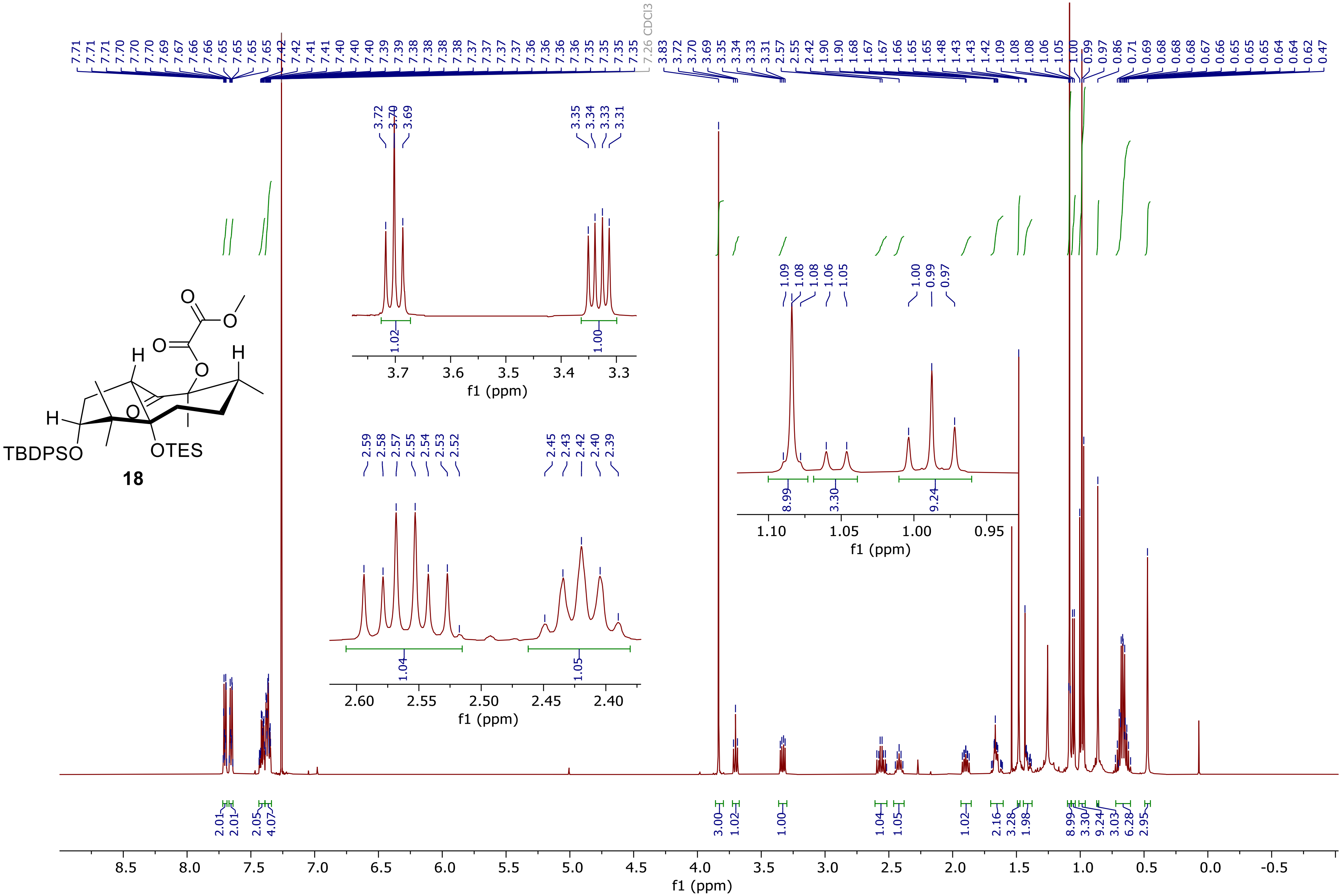

^{13}C NMR (126 MHz, $CDCl_3$)

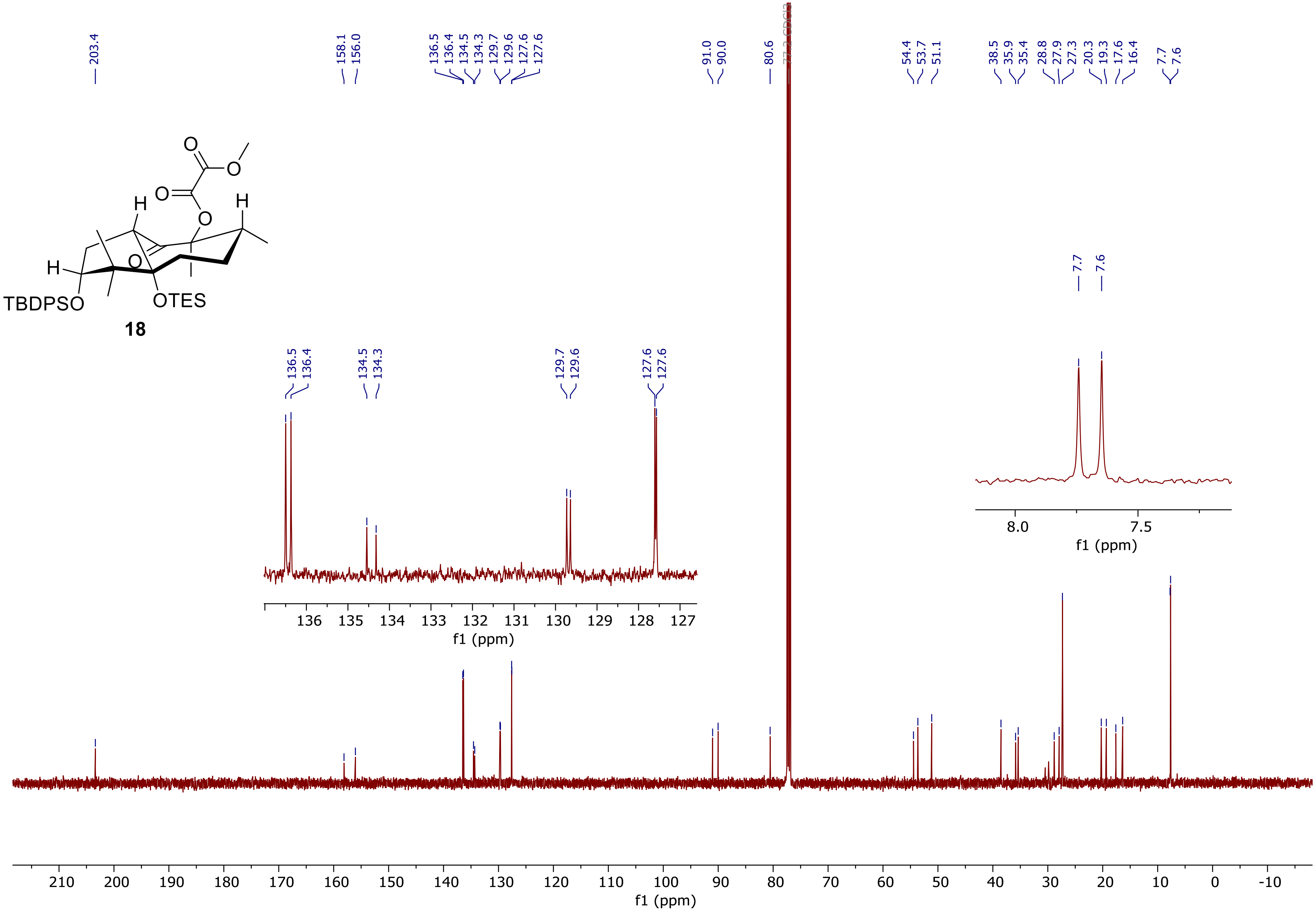

^1H NMR (500 MHz, $CDCl_3$)

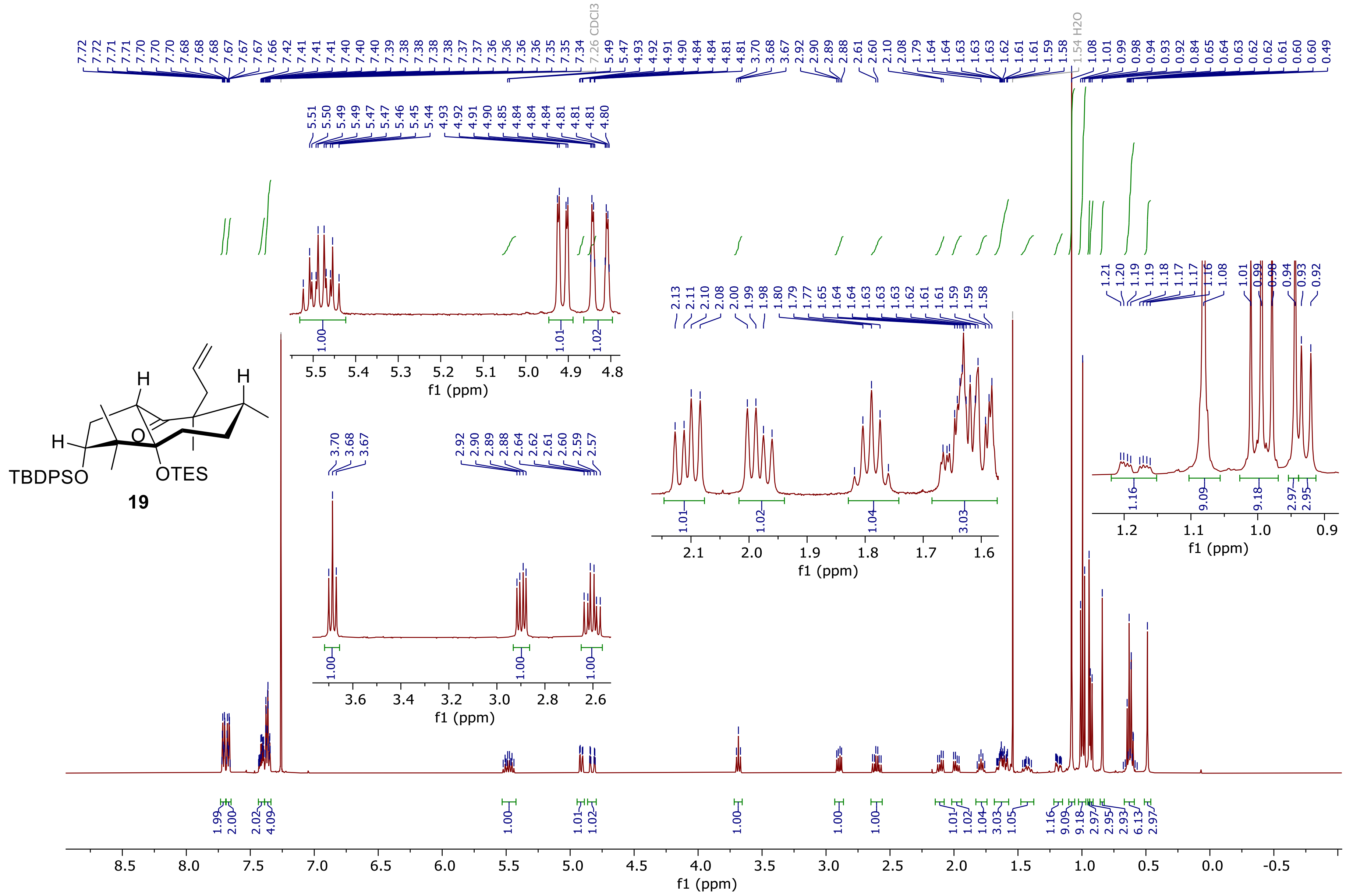

^{13}C NMR (126 MHz, $CDCl_3$)

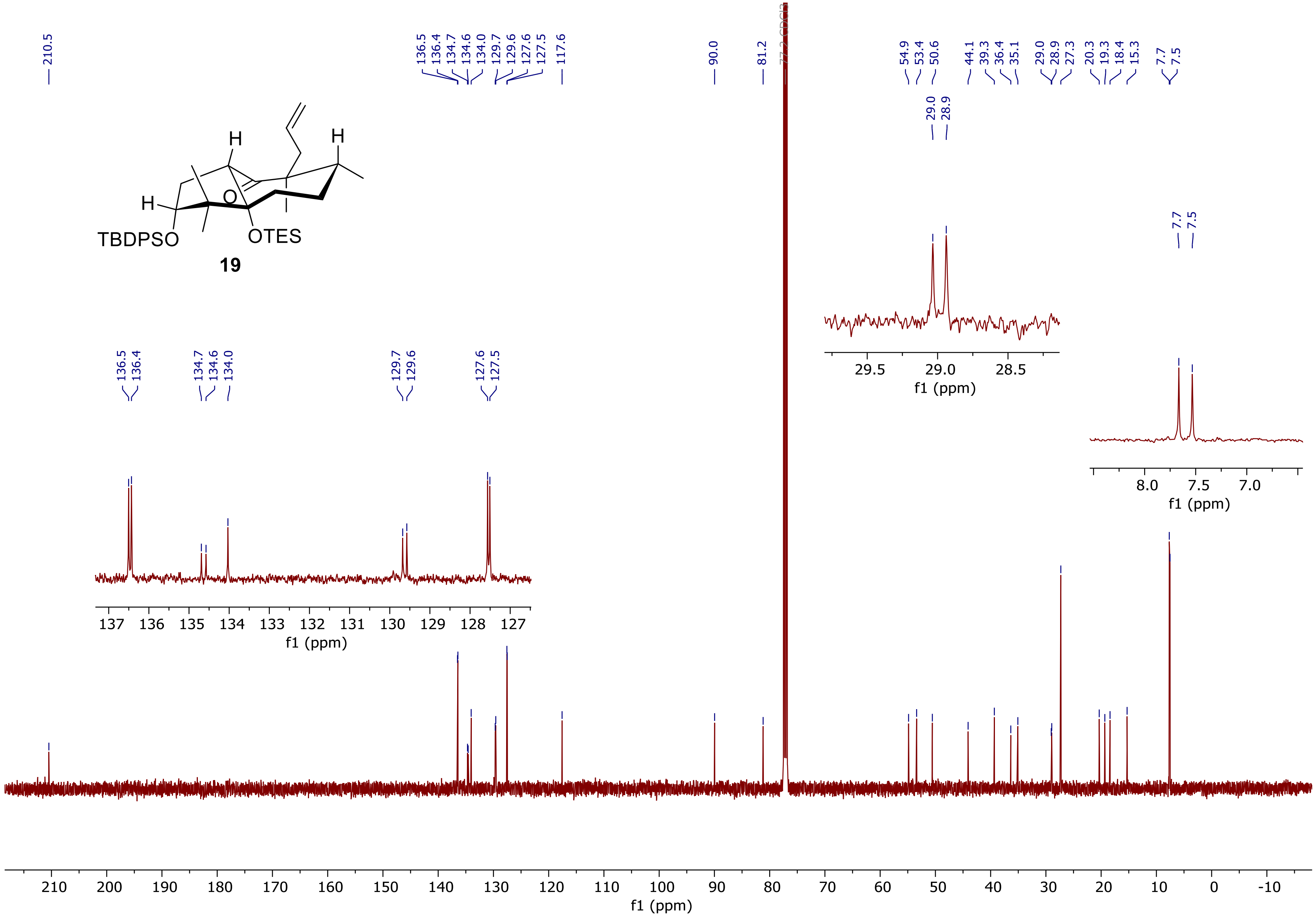

COSY (500 MHz, $CDCl_3$)

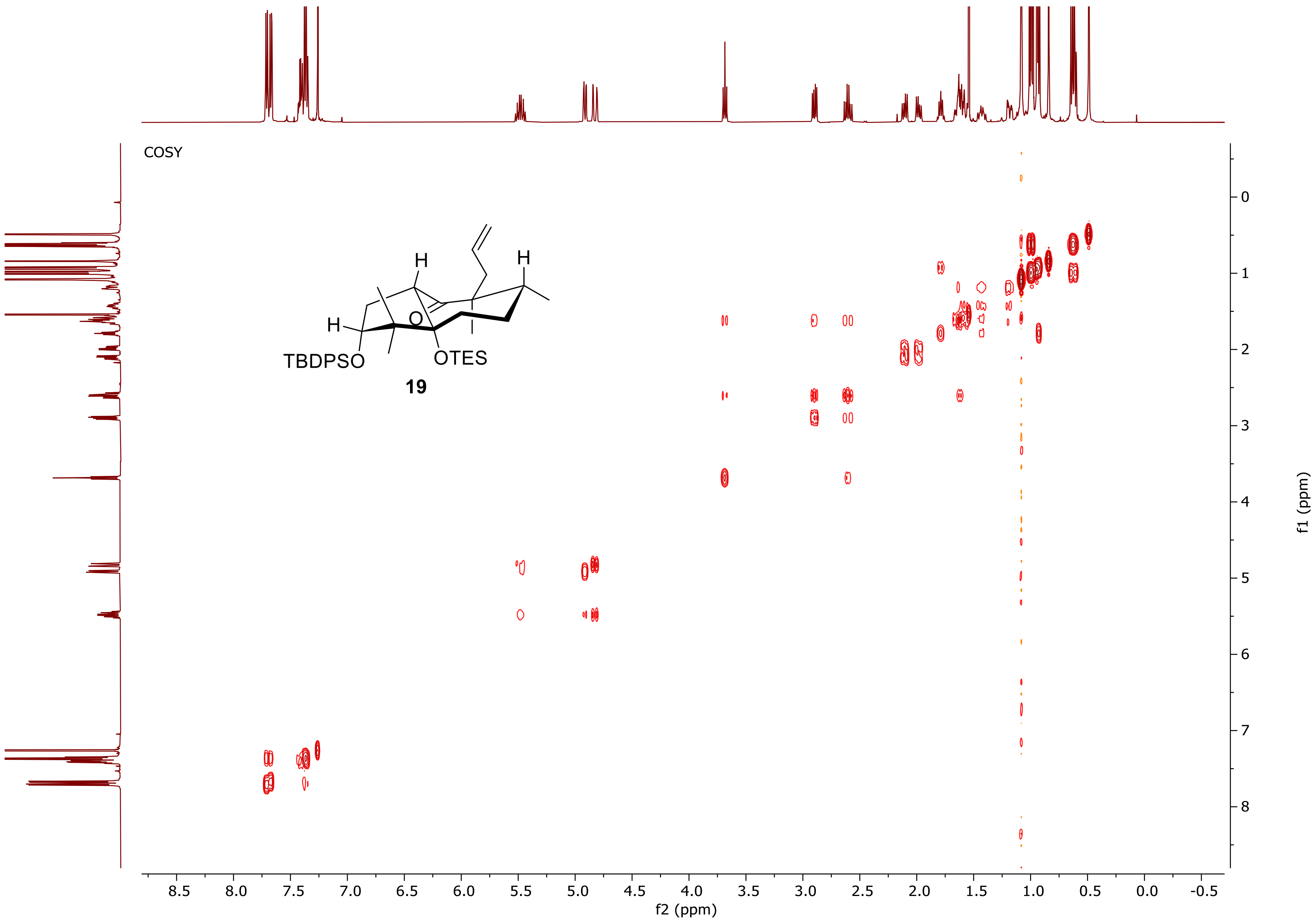

NOESY (500 MHz, $CDCl_3$)

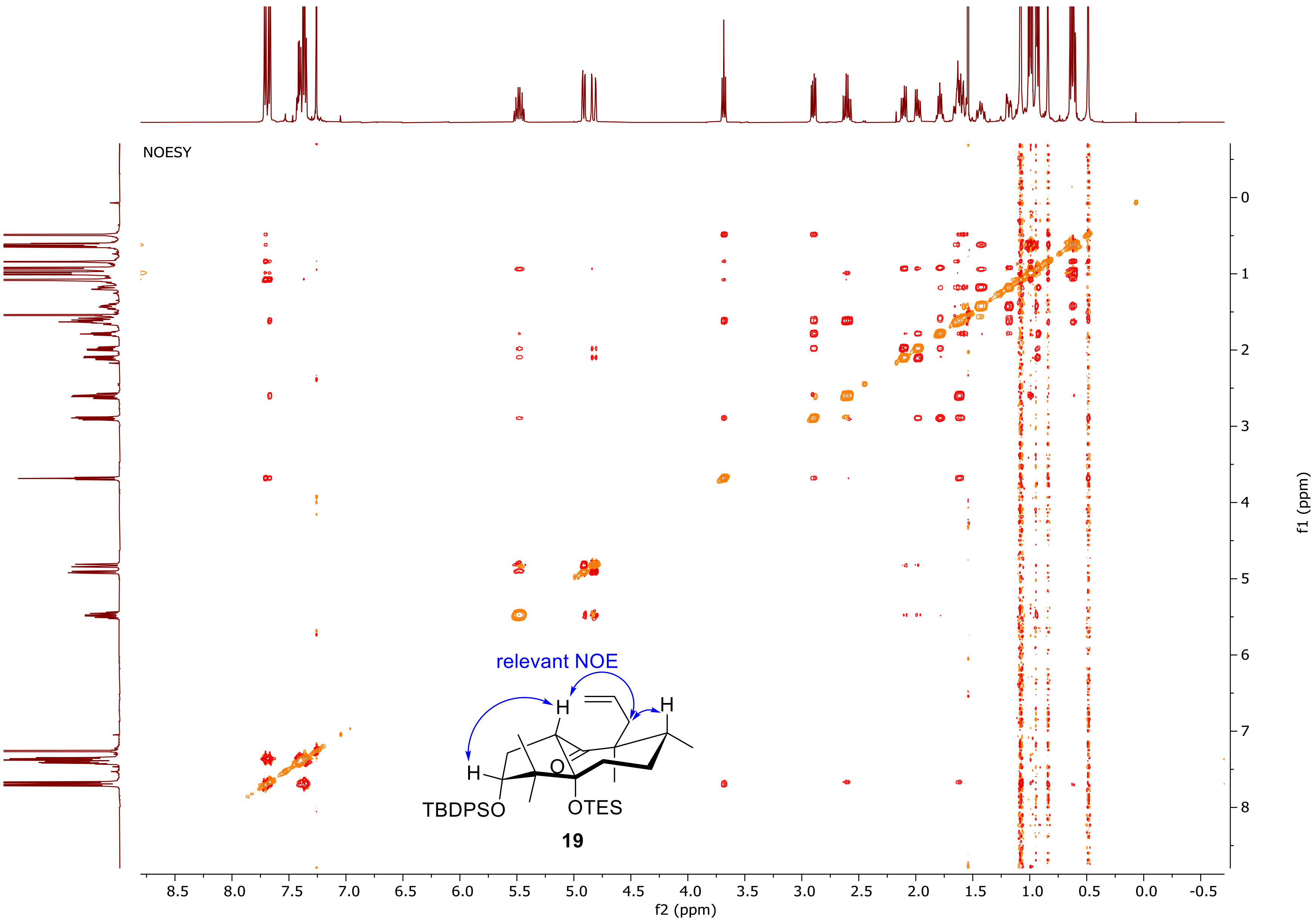

^{1}H NMR (500 MHz, $CDCl_3$)

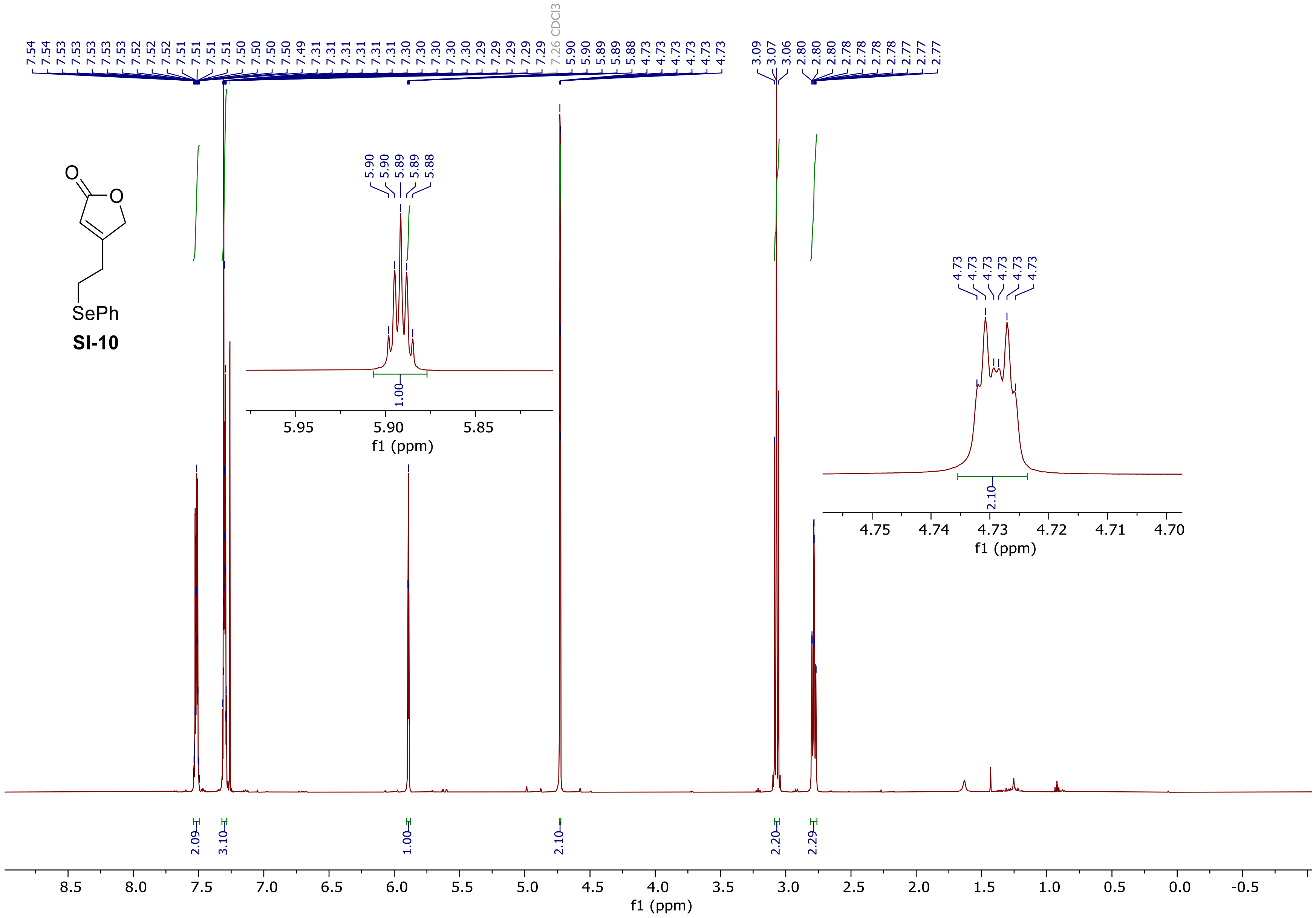

^{13}C NMR (126 MHz, $CDCl_3$)

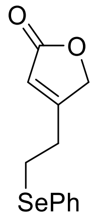

SI-10

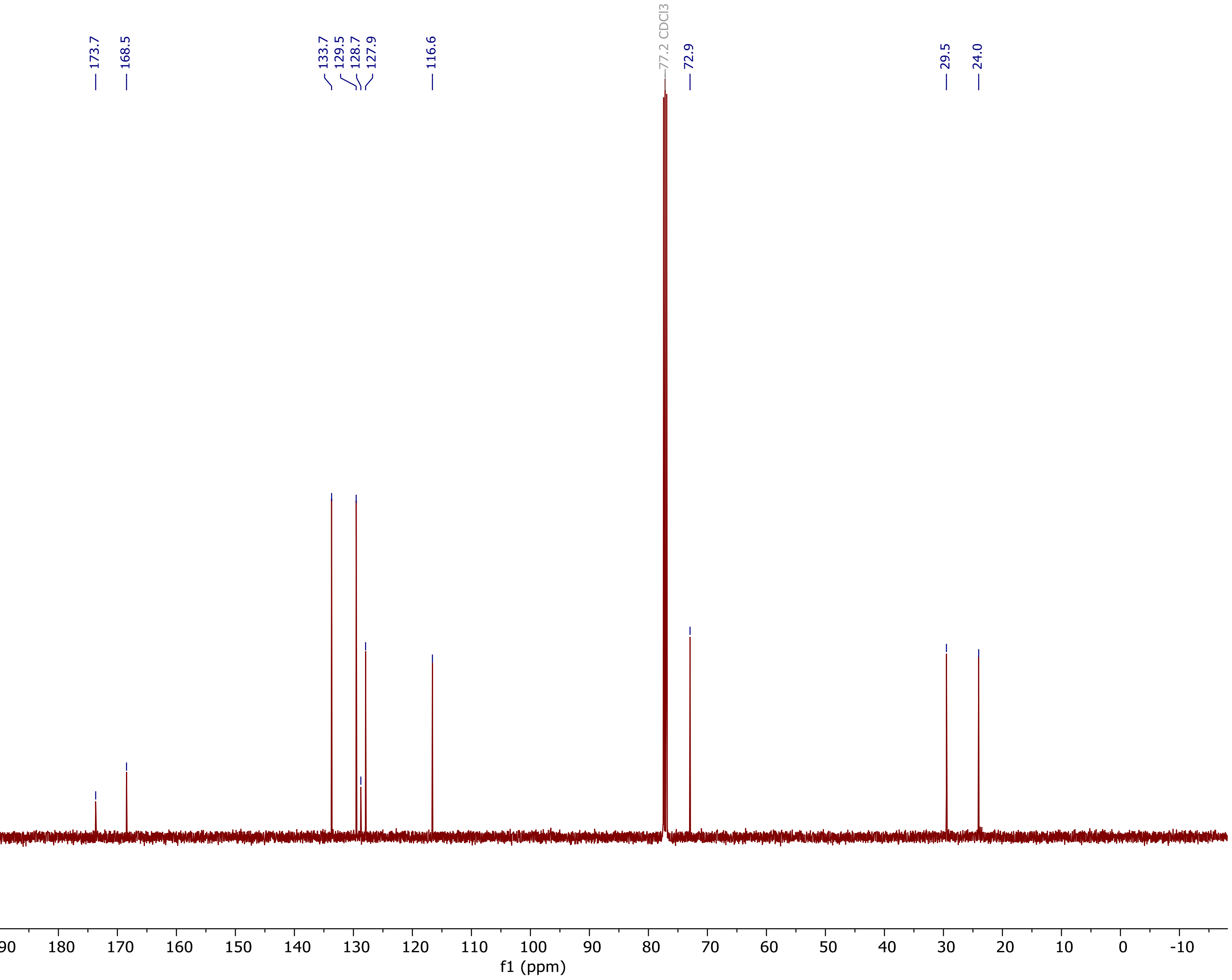

^1H NMR (500 MHz, C_6D_6)

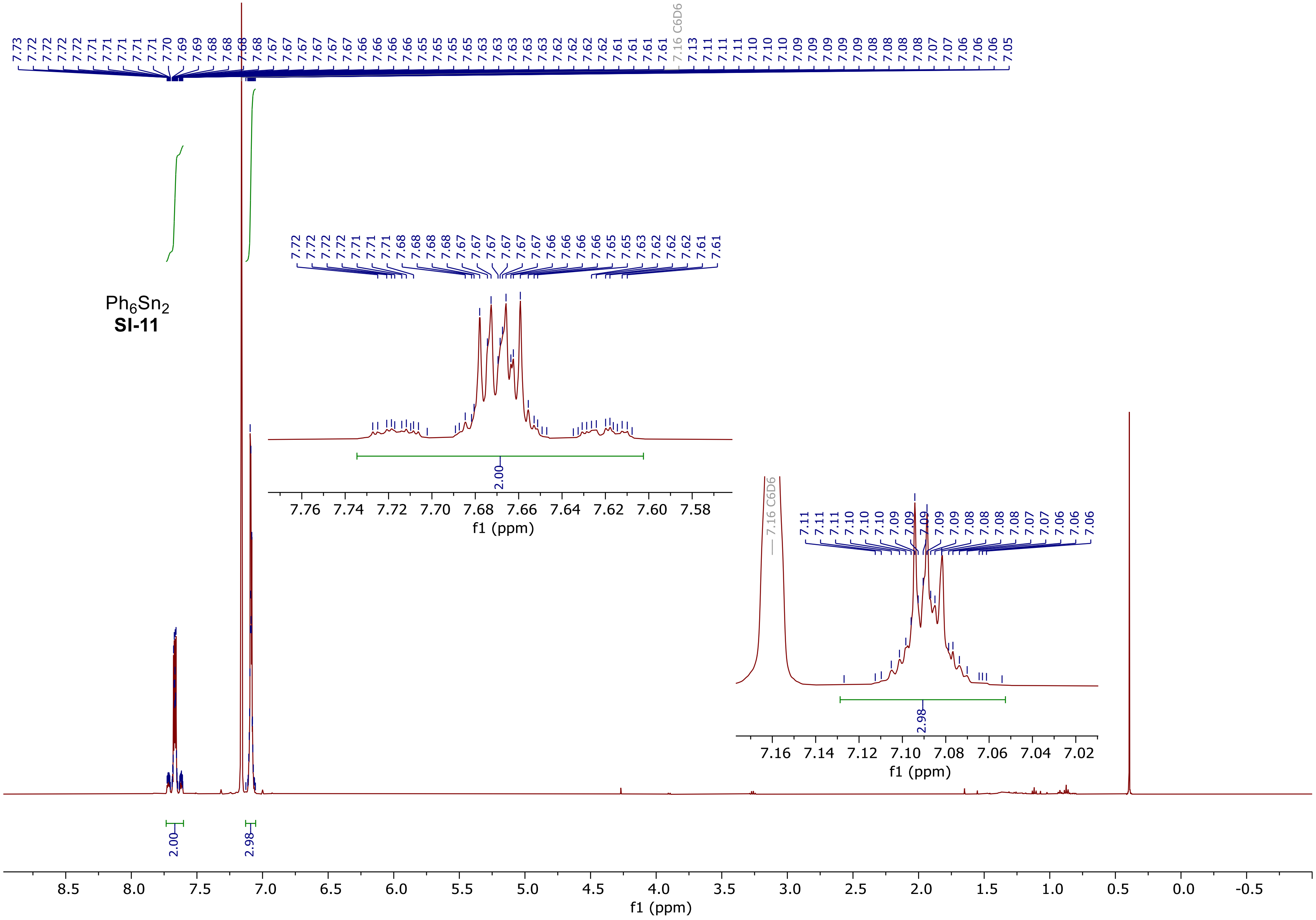

^{13}C NMR (126 MHz, C_6D_6)

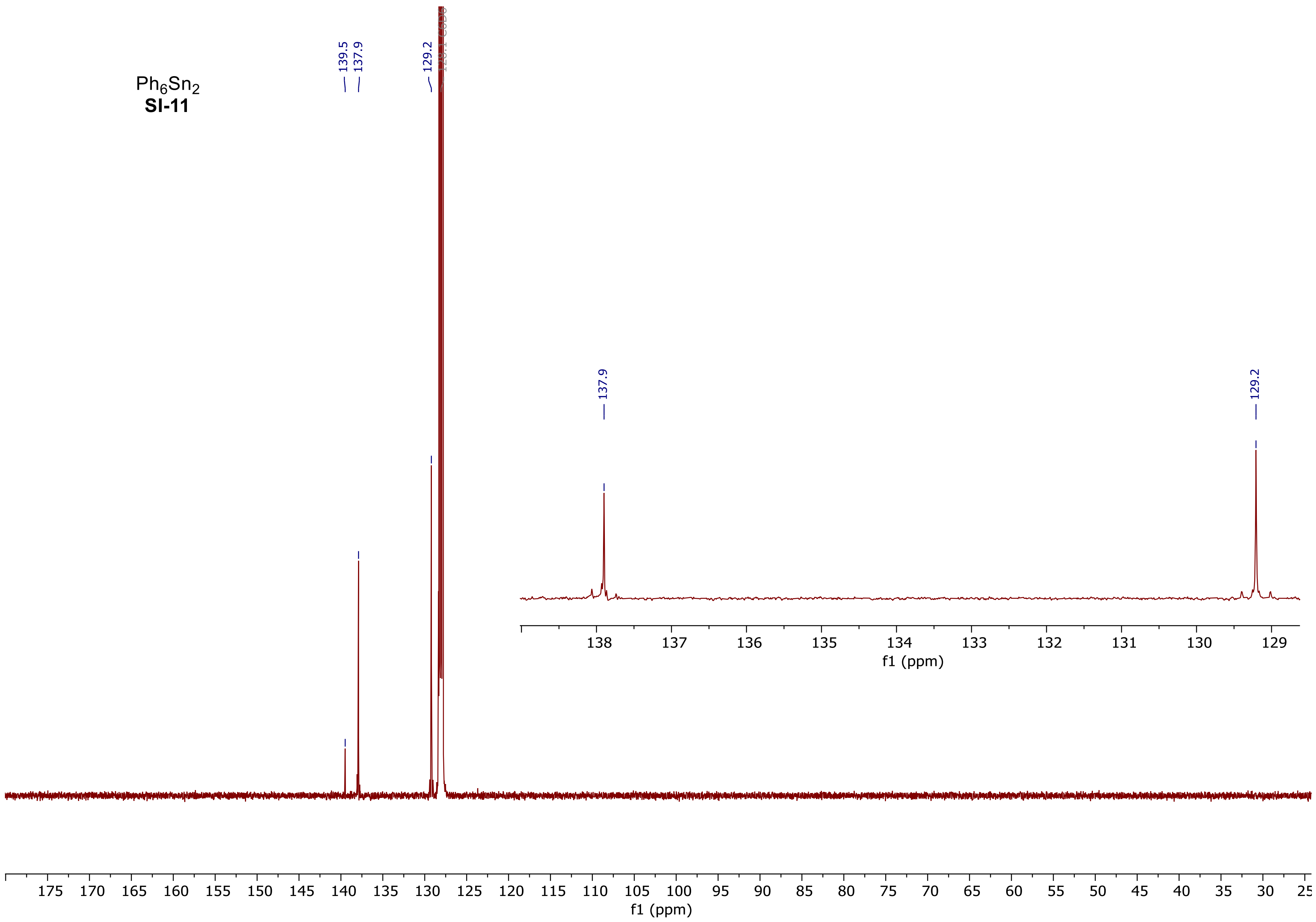

^{1}H NMR (500 MHz, $CDCl_3$)

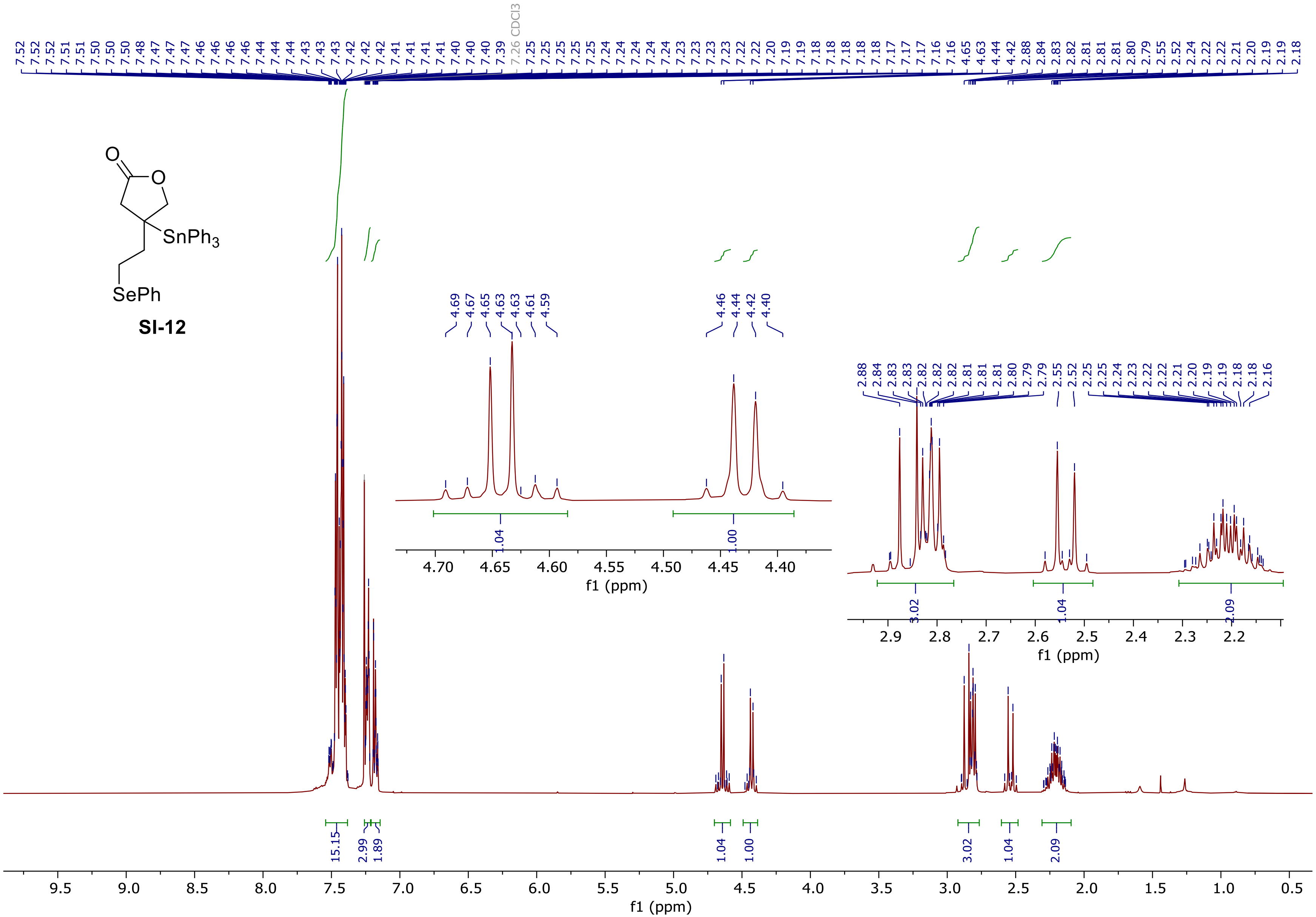

^{13}C NMR (126 MHz, $CDCl_3$)

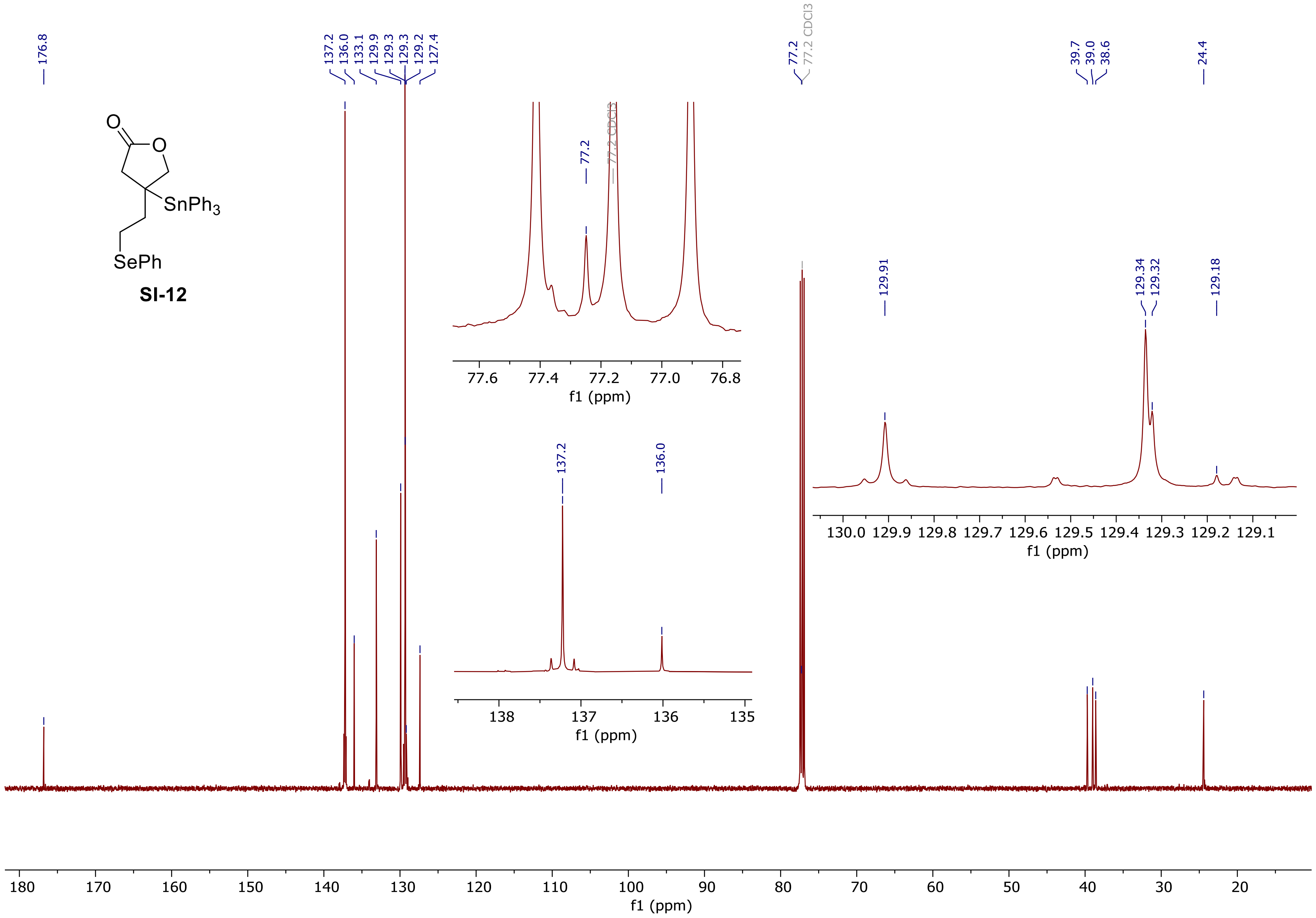

^{1}H NMR (500 MHz, $CDCl_3$)

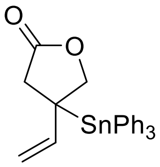

20

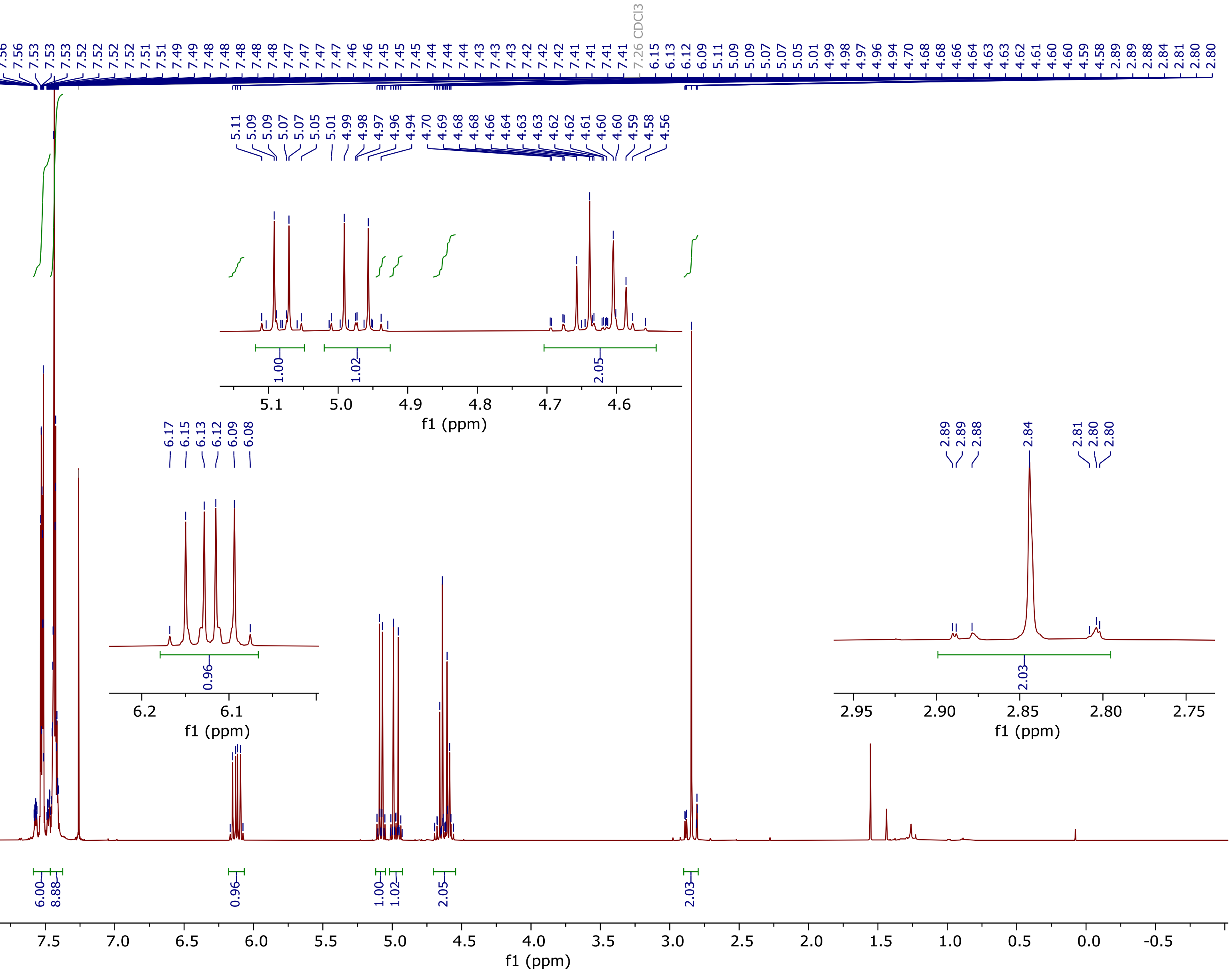

^{13}C NMR (126 MHz, $CDCl_3$)

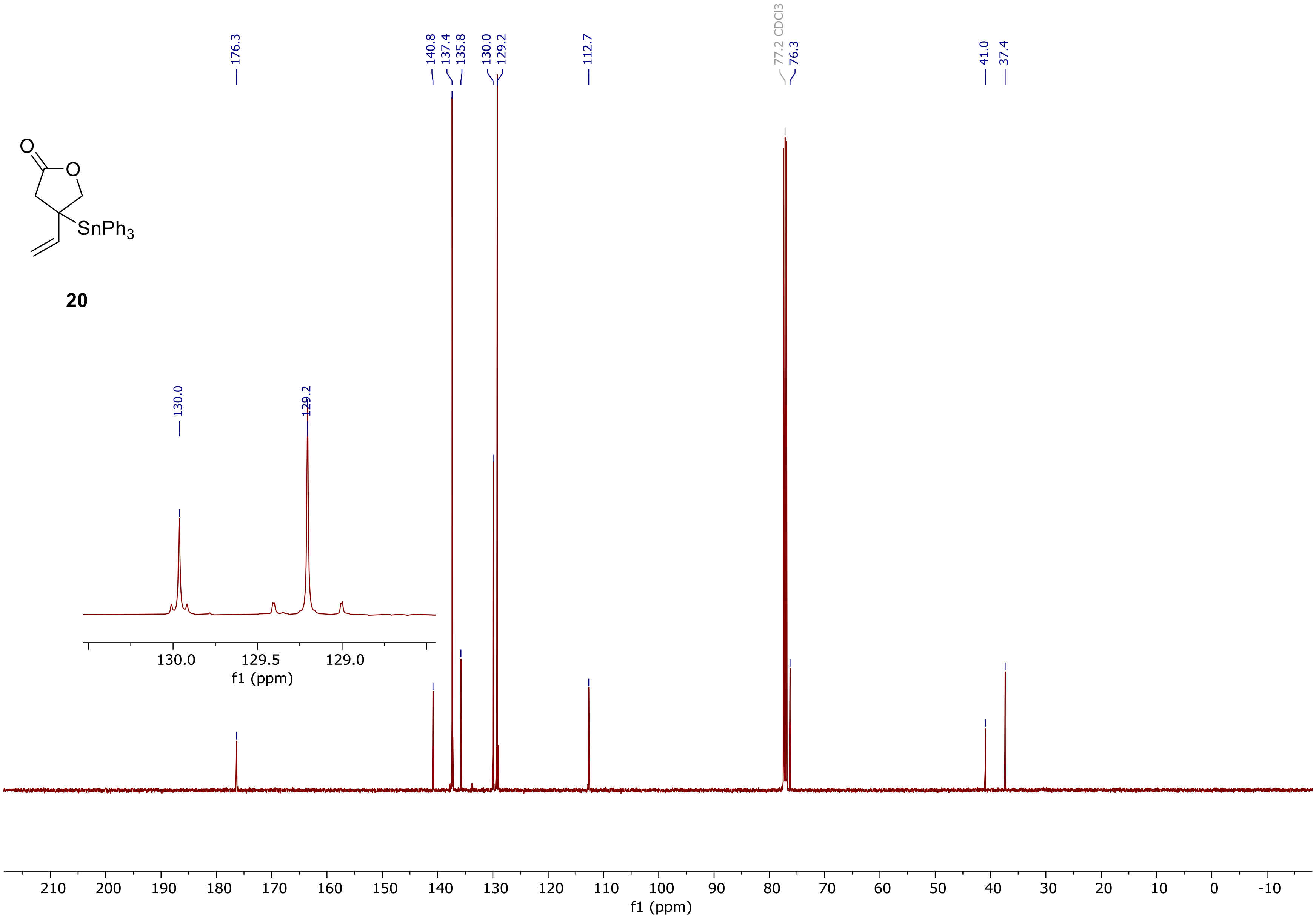

20

^{1}H NMR (500 MHz, $CDCl_3$)

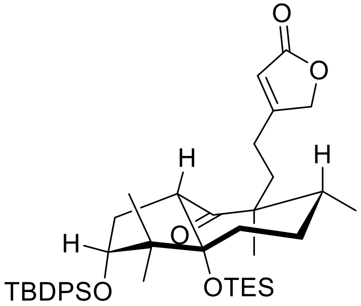

21

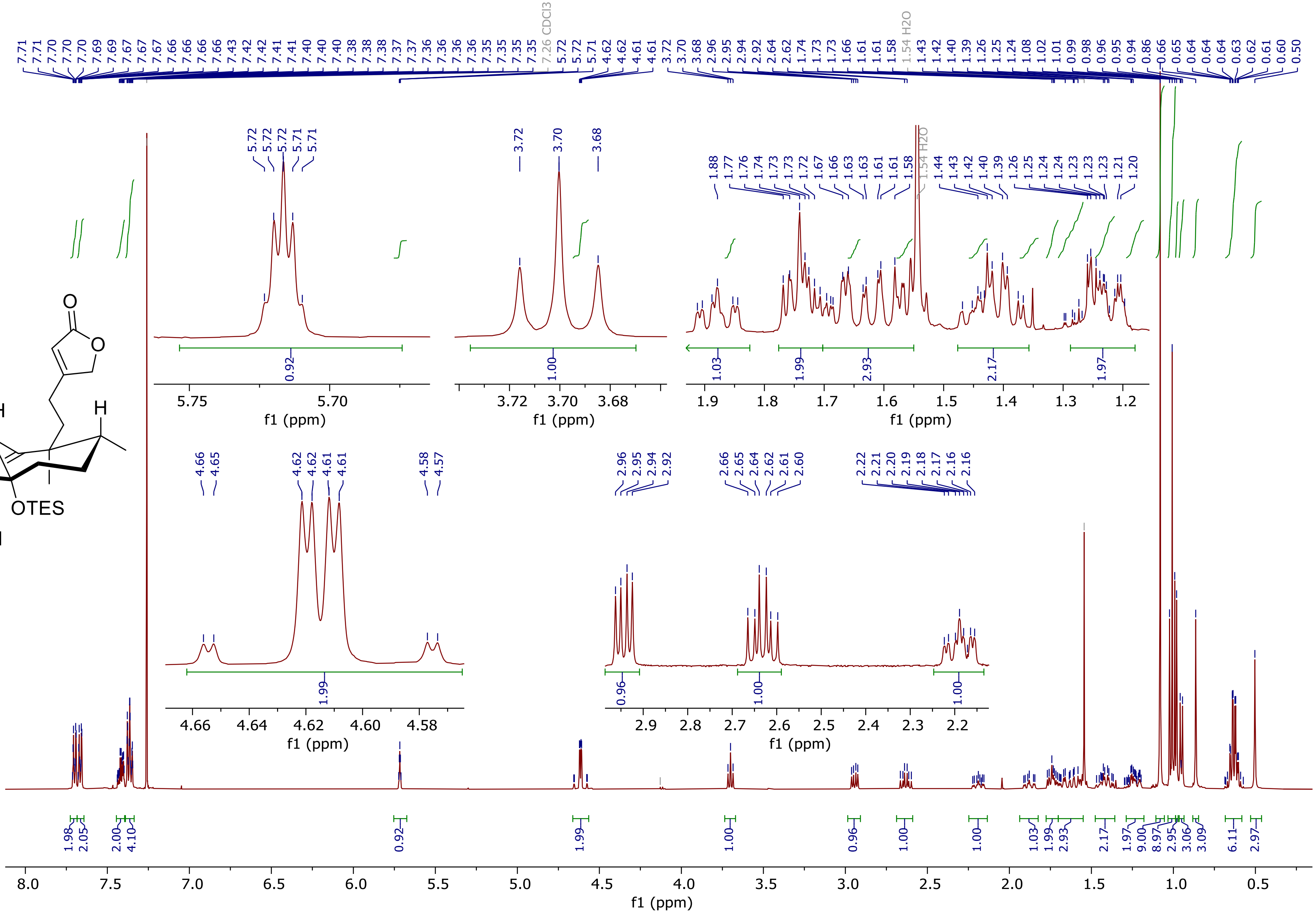

^{13}C NMR (126 MHz, $CDCl_3$)

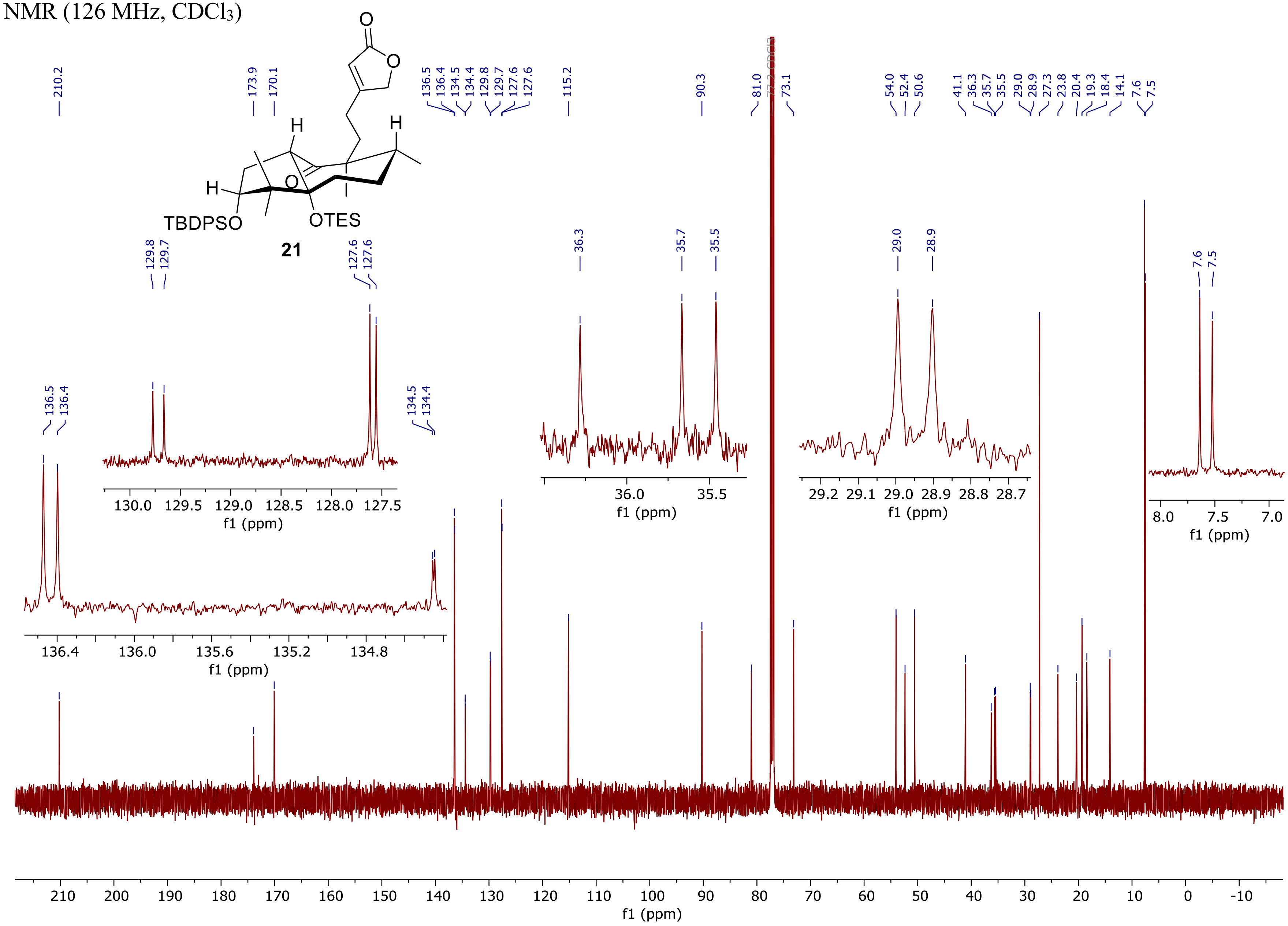

COSY (500 MHz, $CDCl_3$)

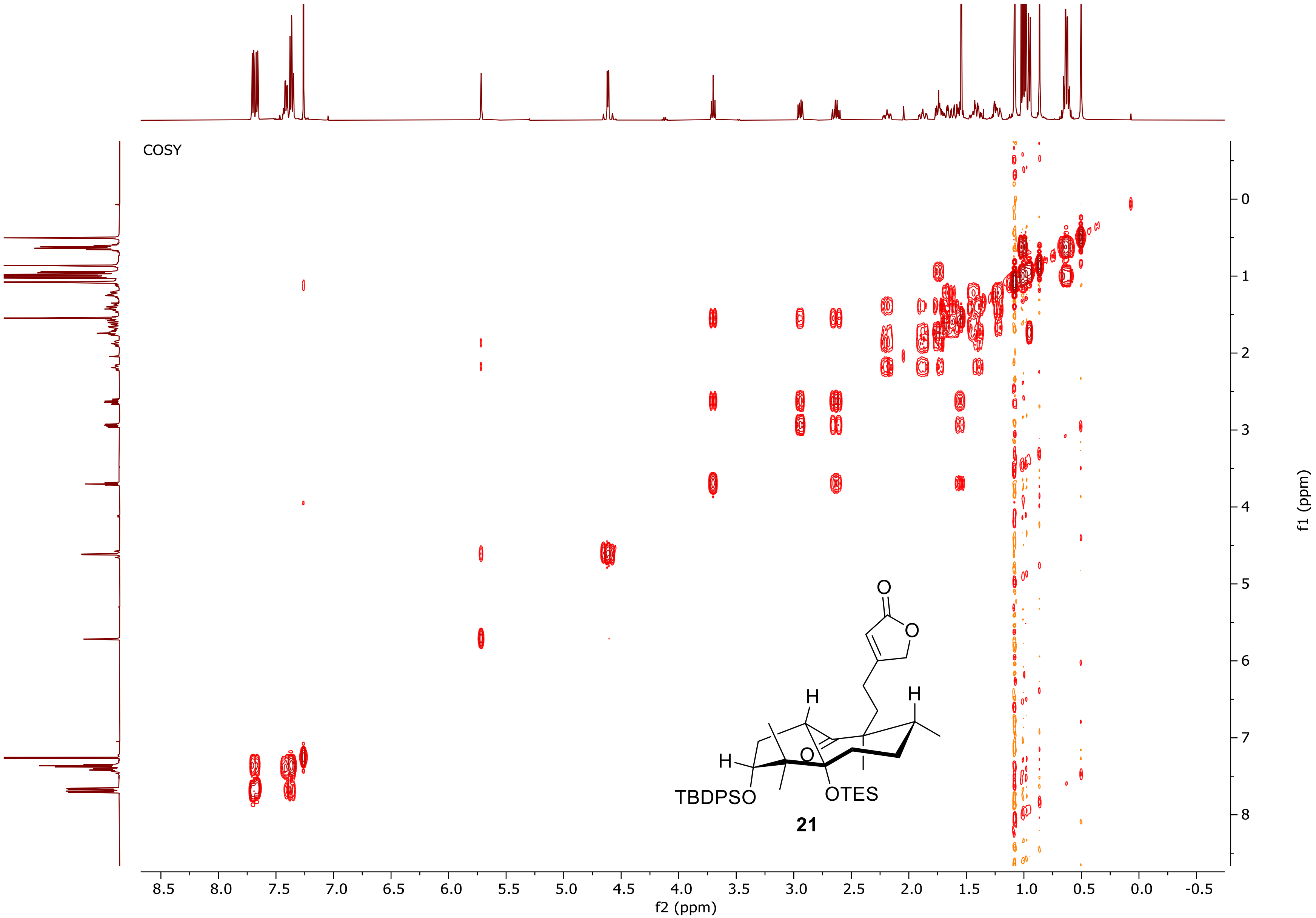

NOESY (500 MHz, $CDCl_3$)

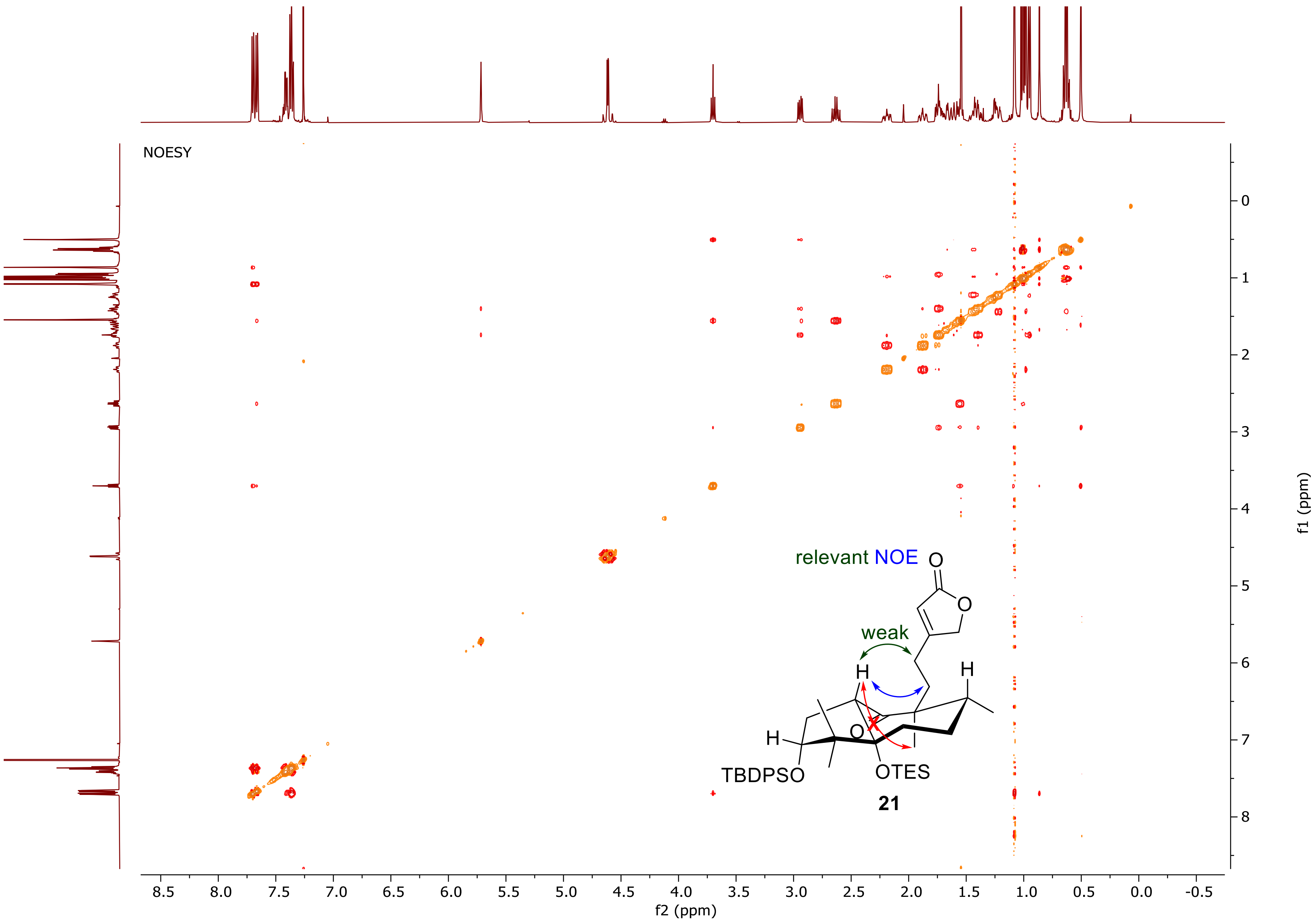

^{1}H NMR (500 MHz, $CDCl_3$)

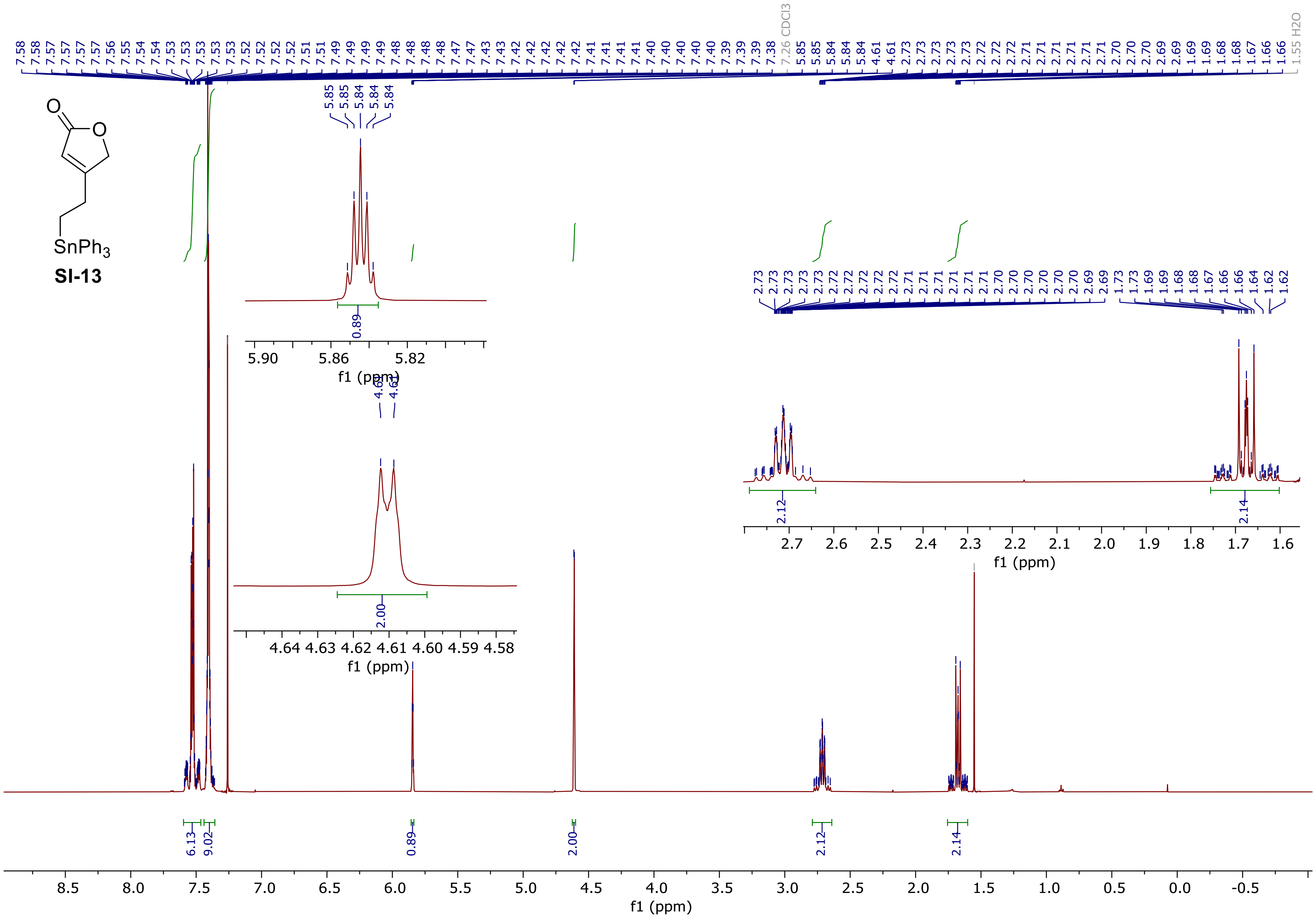

^{13}C NMR (126 MHz, $CDCl_3$)

SI-13

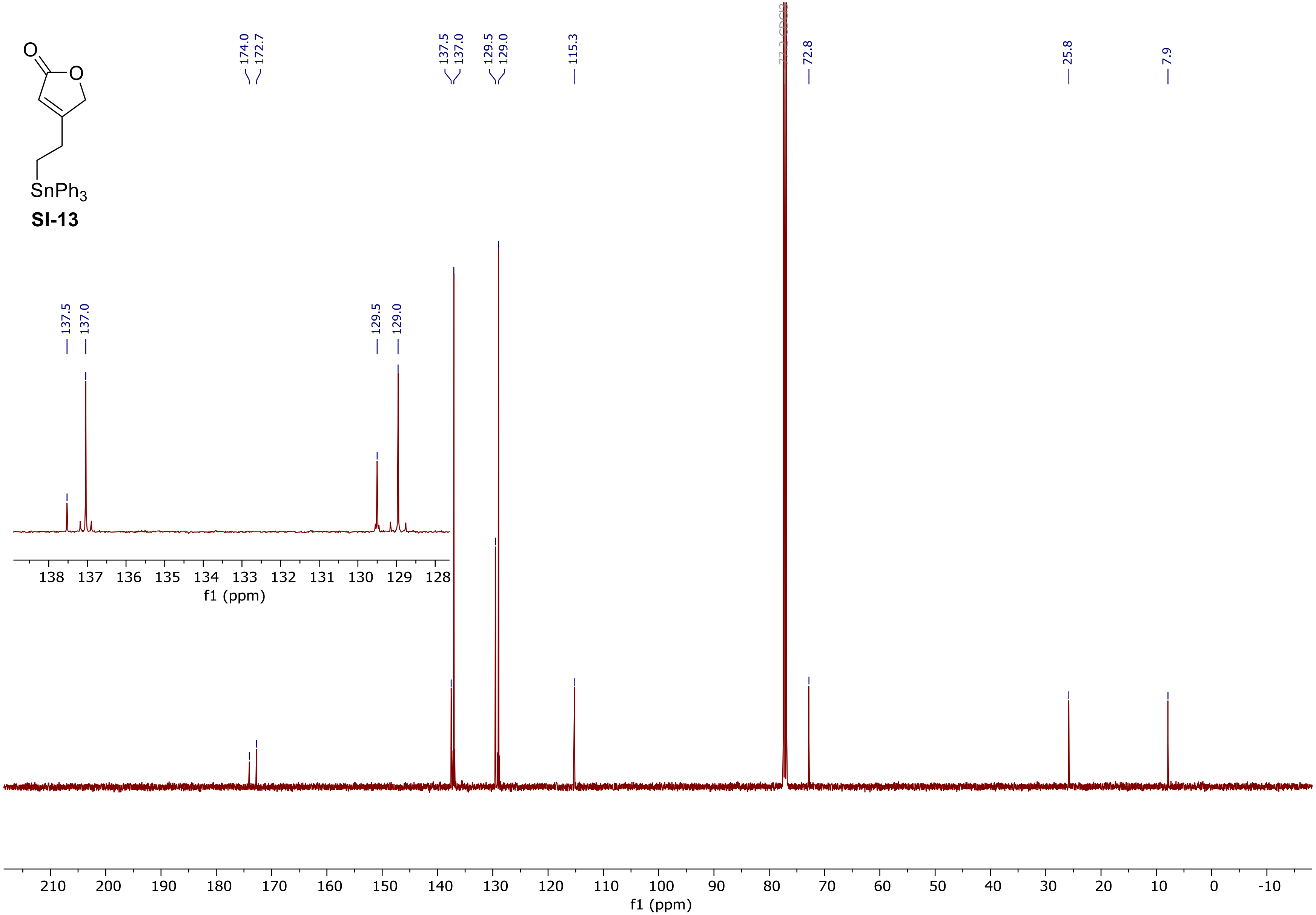

^{1}H NMR (500 MHz, $CDCl_3$)

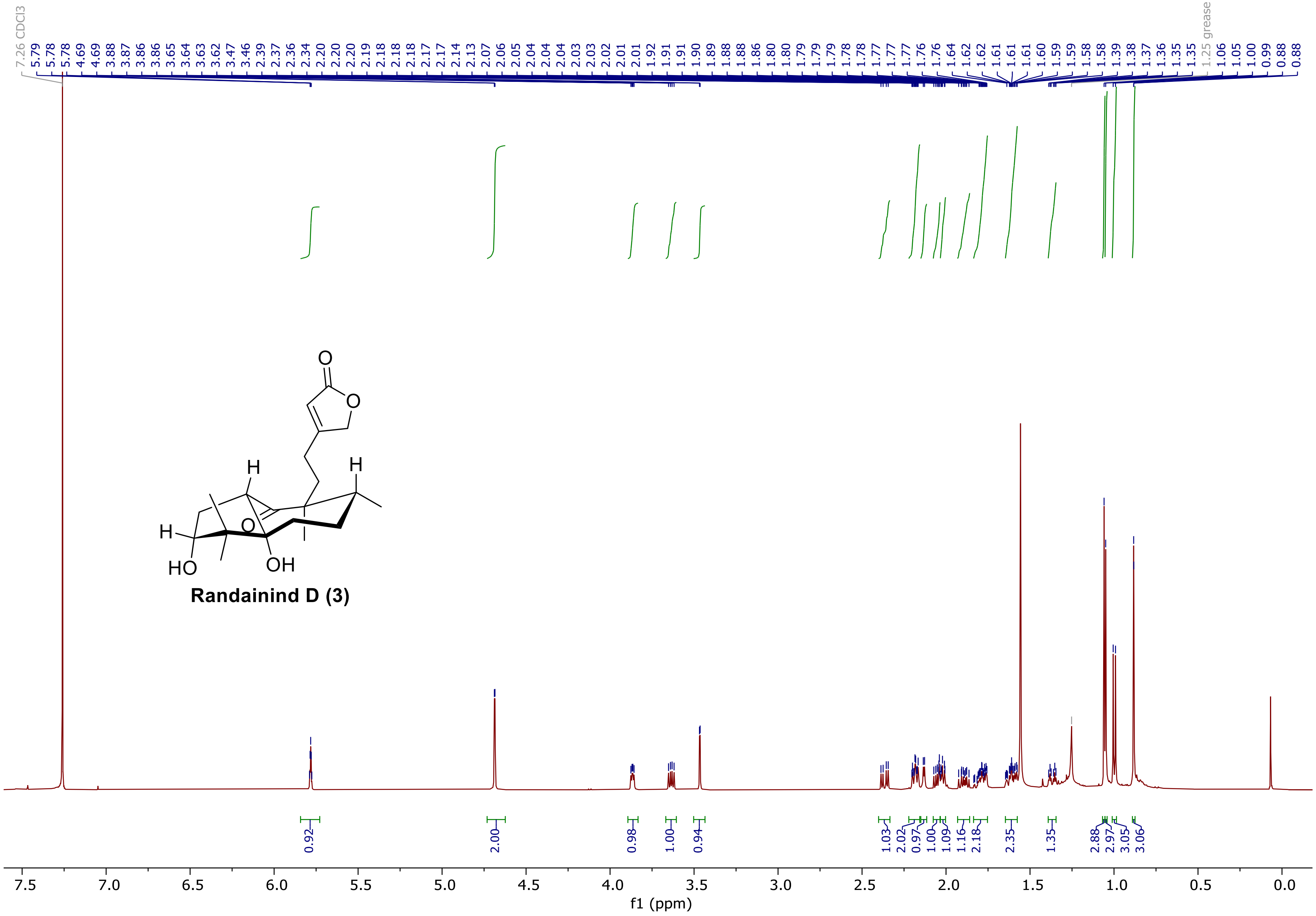

^{1}H NMR (500 MHz, $CDCl_3$)

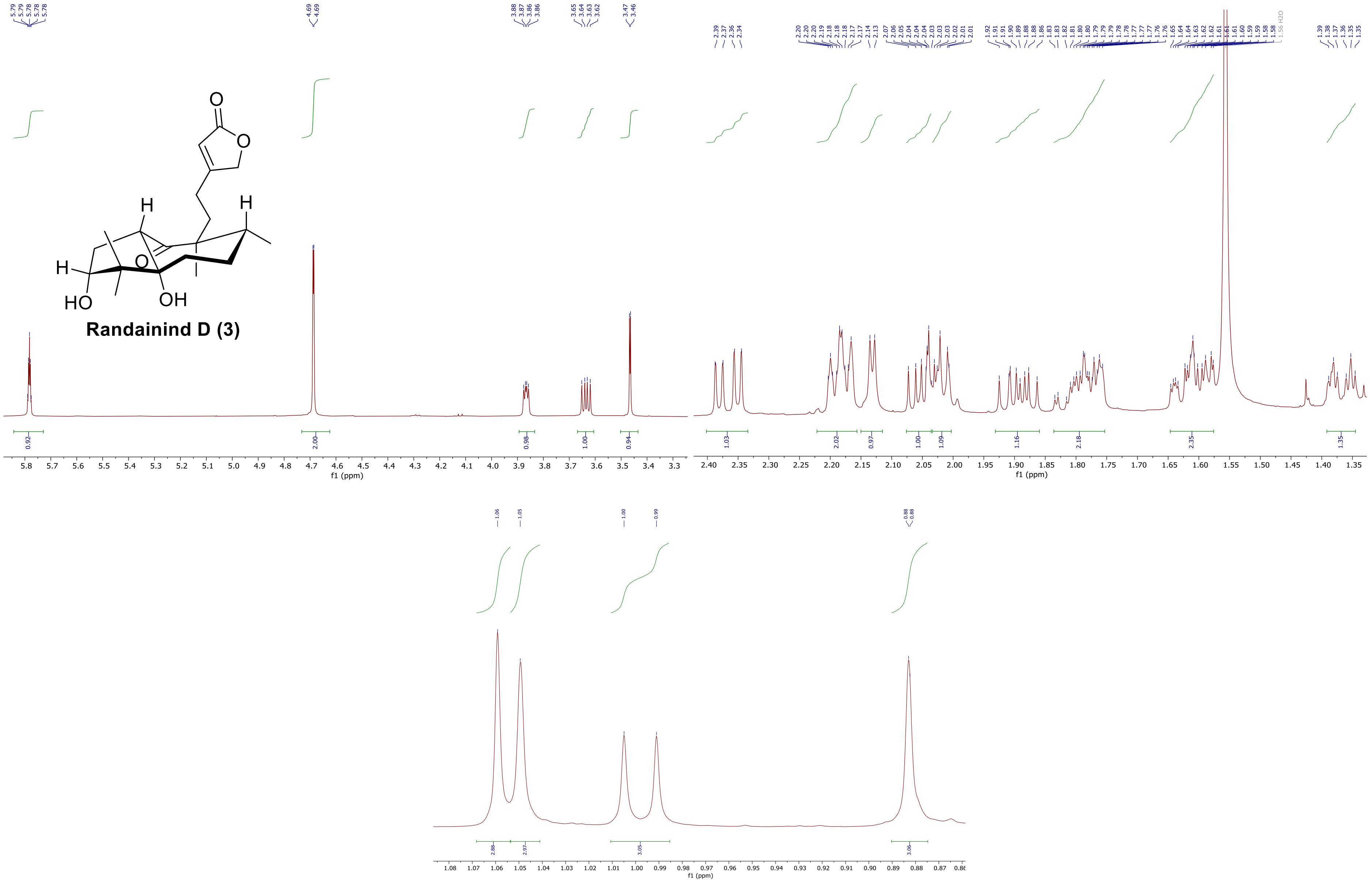

^{13}C NMR (126 MHz, $CDCl_3$)

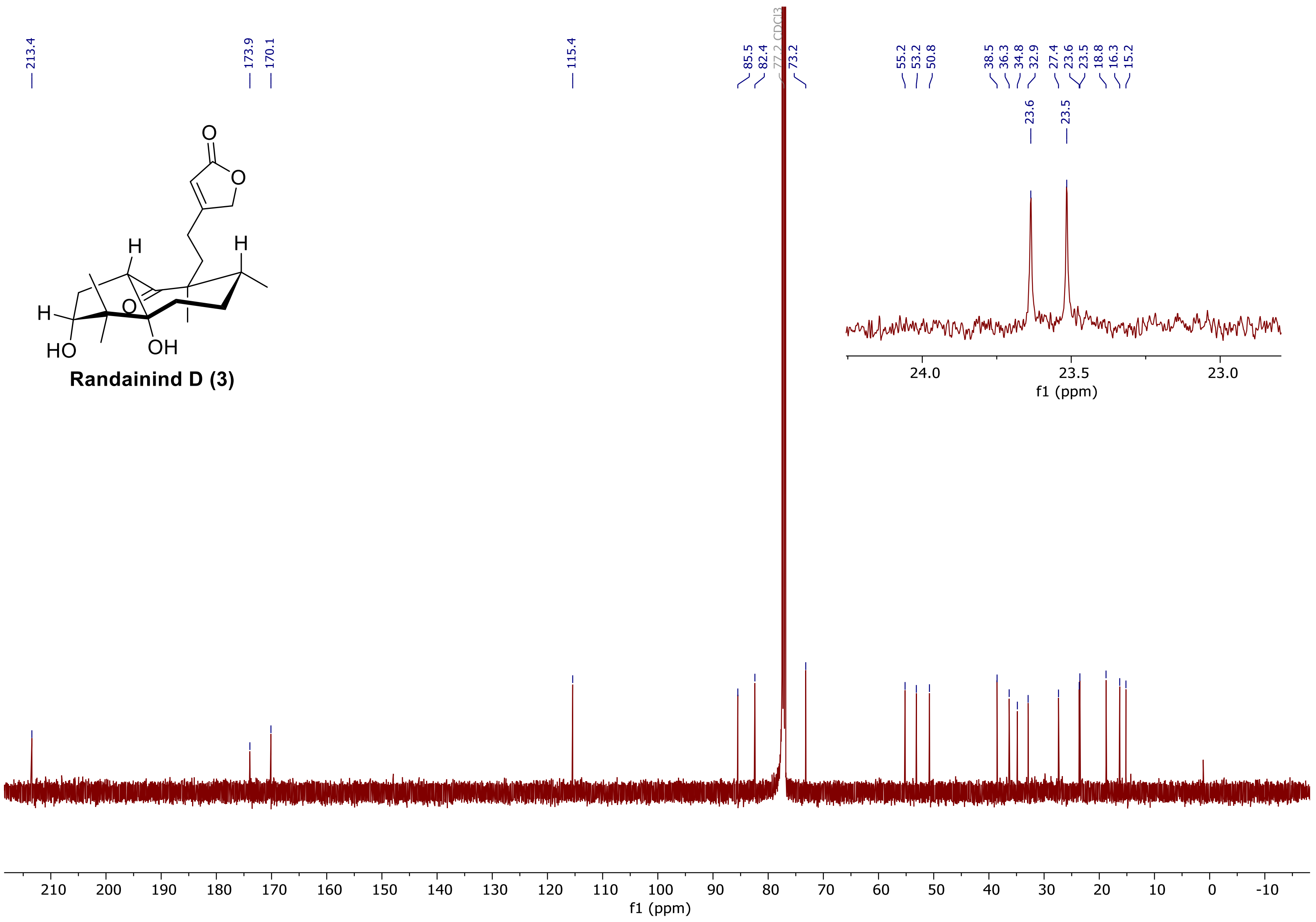

$CDCl_3$ peak at 77.16 ppm

^{13}C NMR (126 MHz, $CDCl_3$)

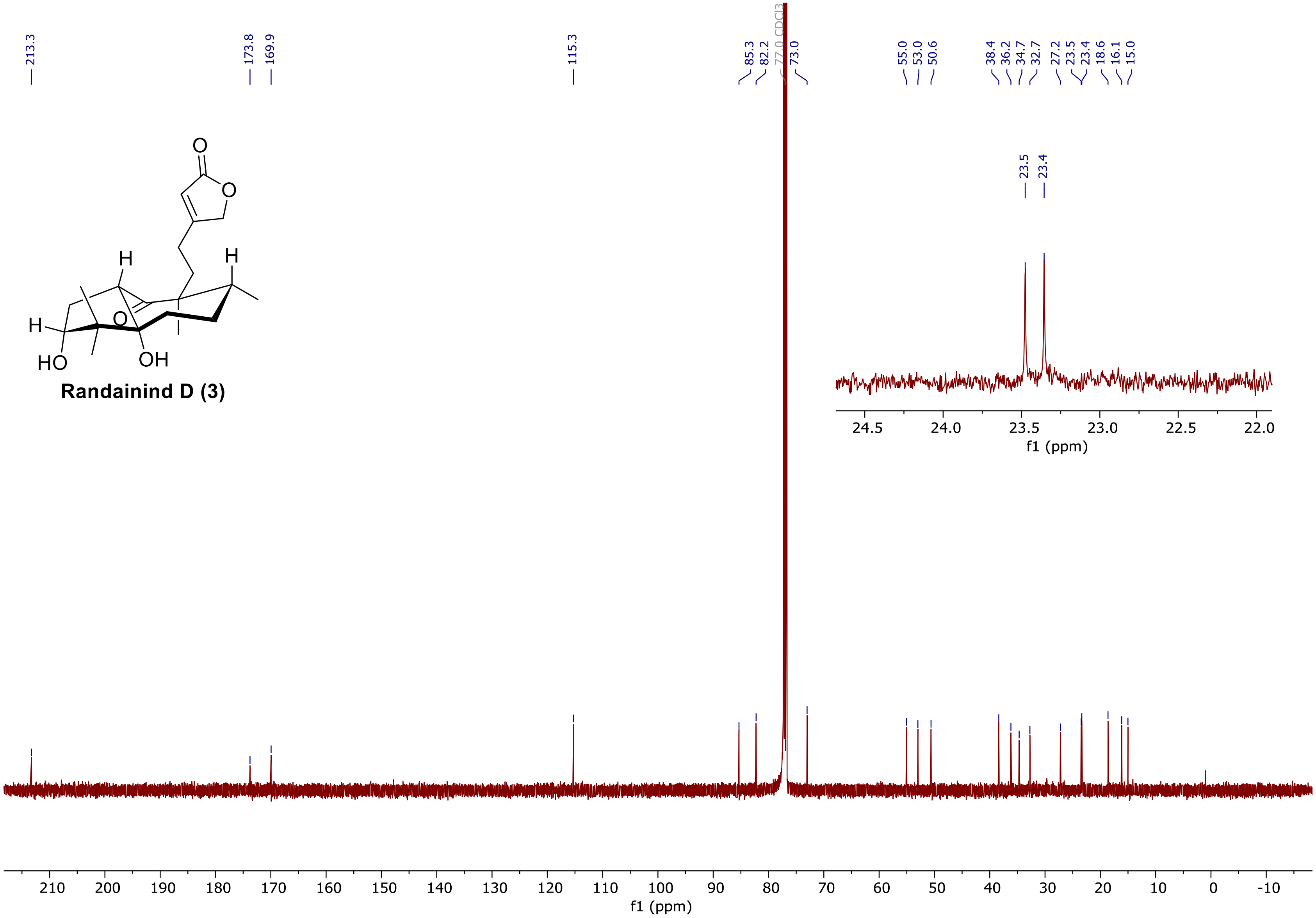

Randainind D (3)

$CDCl_3$ peak at 77.00 ppm

^{1}H NMR (500 MHz, $CDCl_3$)

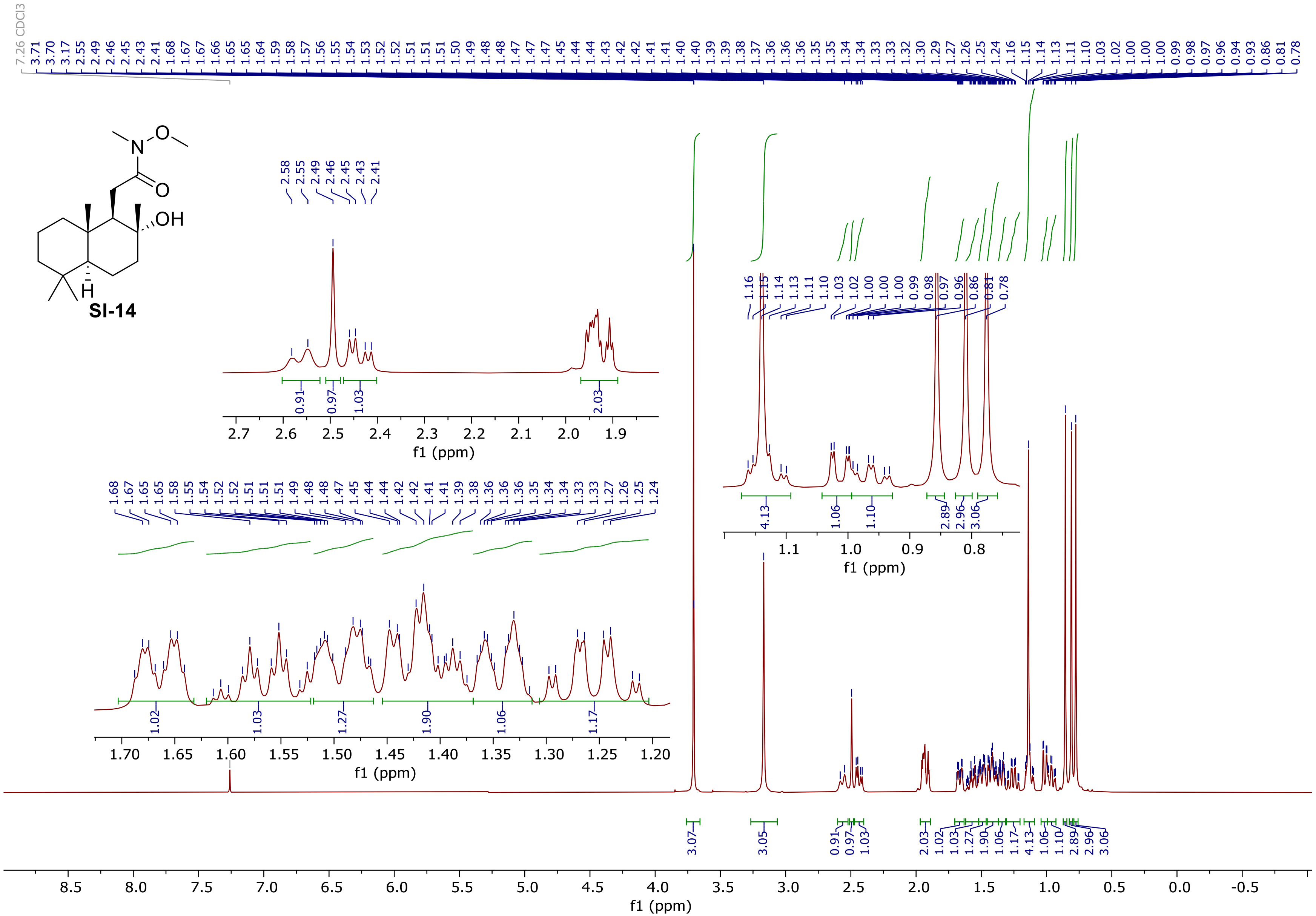

^{13}C NMR (126 MHz, $CDCl_3$)

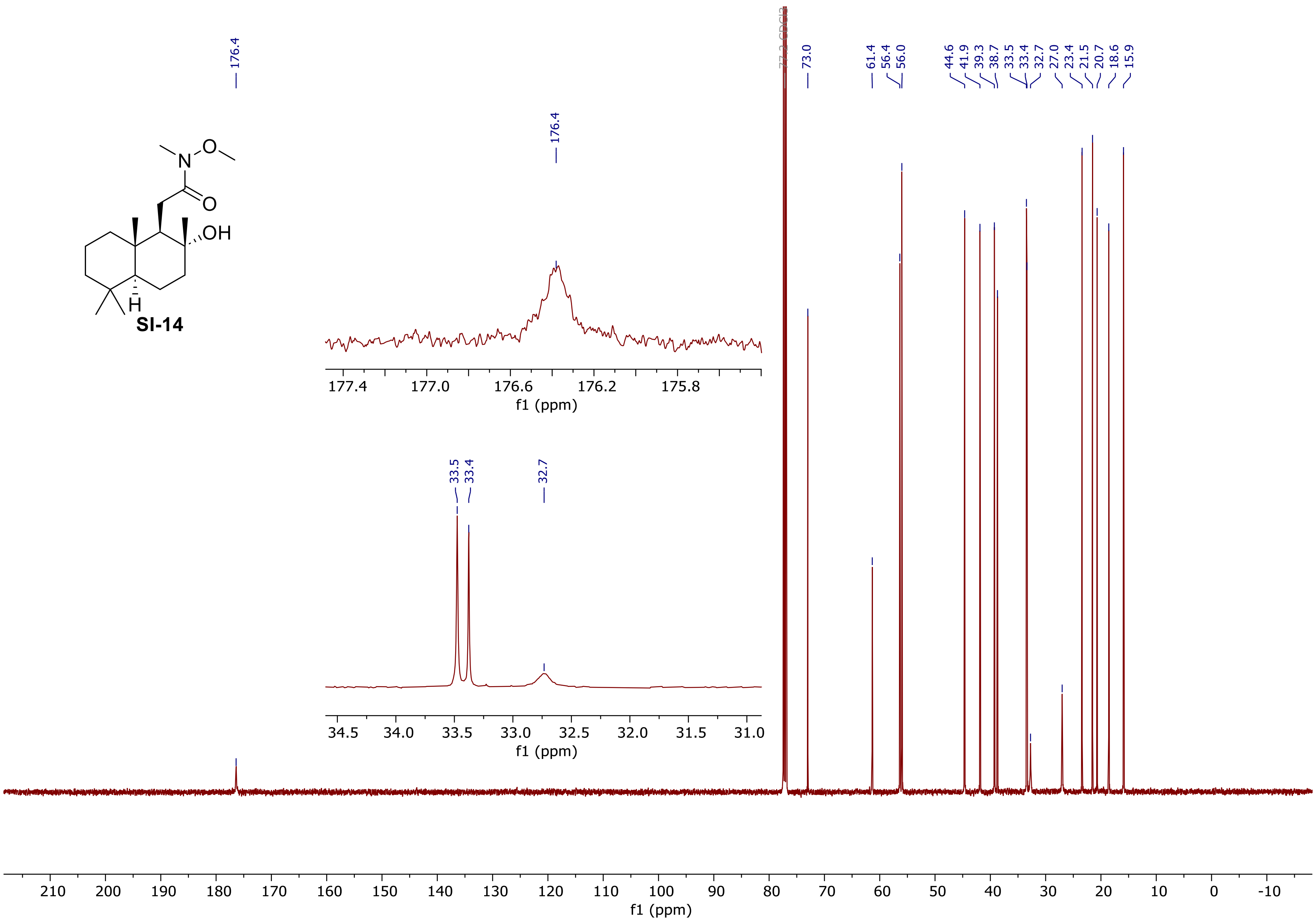

^{1}H NMR (500 MHz, $CDCl_3$)

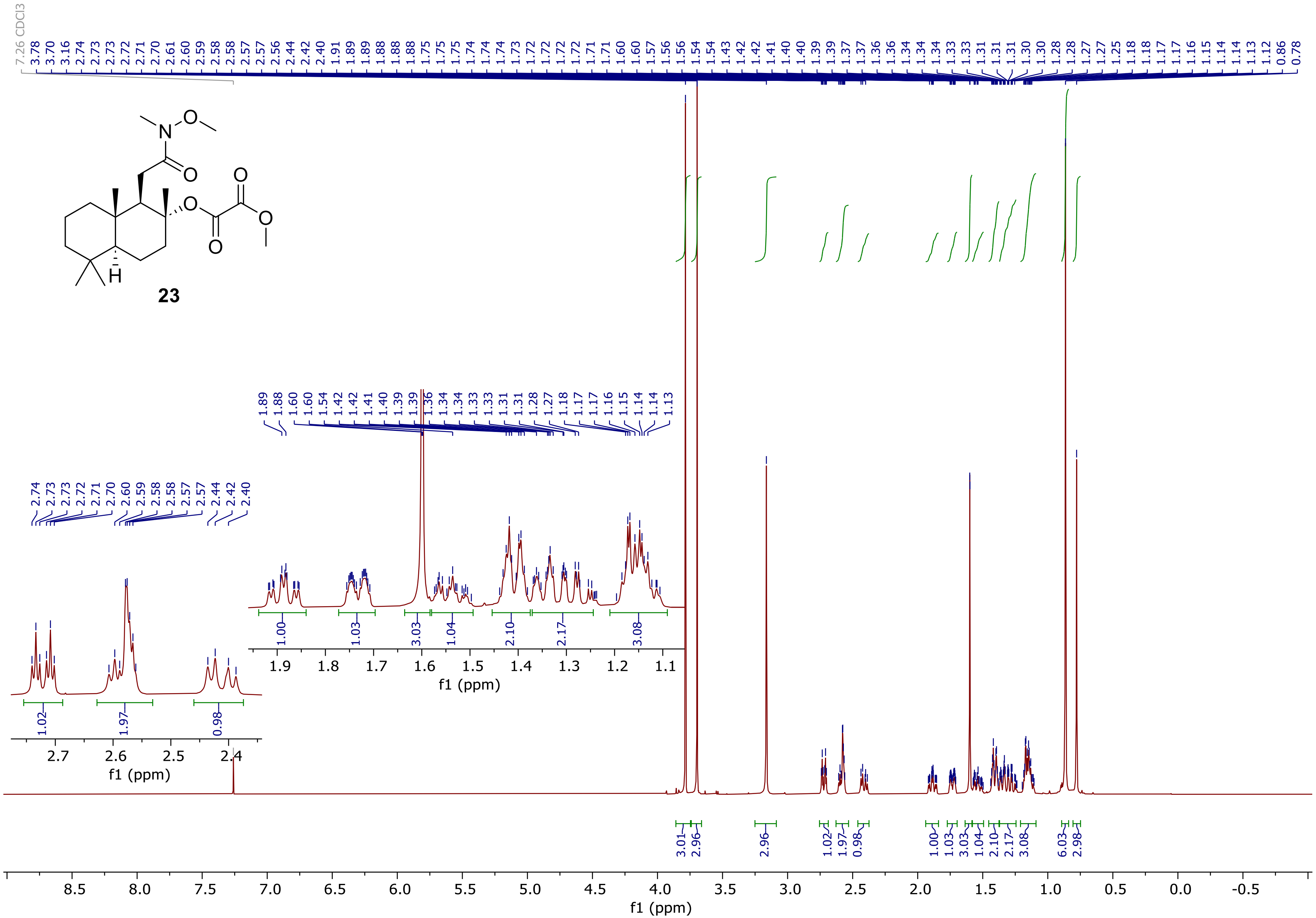

23

^{13}C NMR (126 MHz, $CDCl_3$)

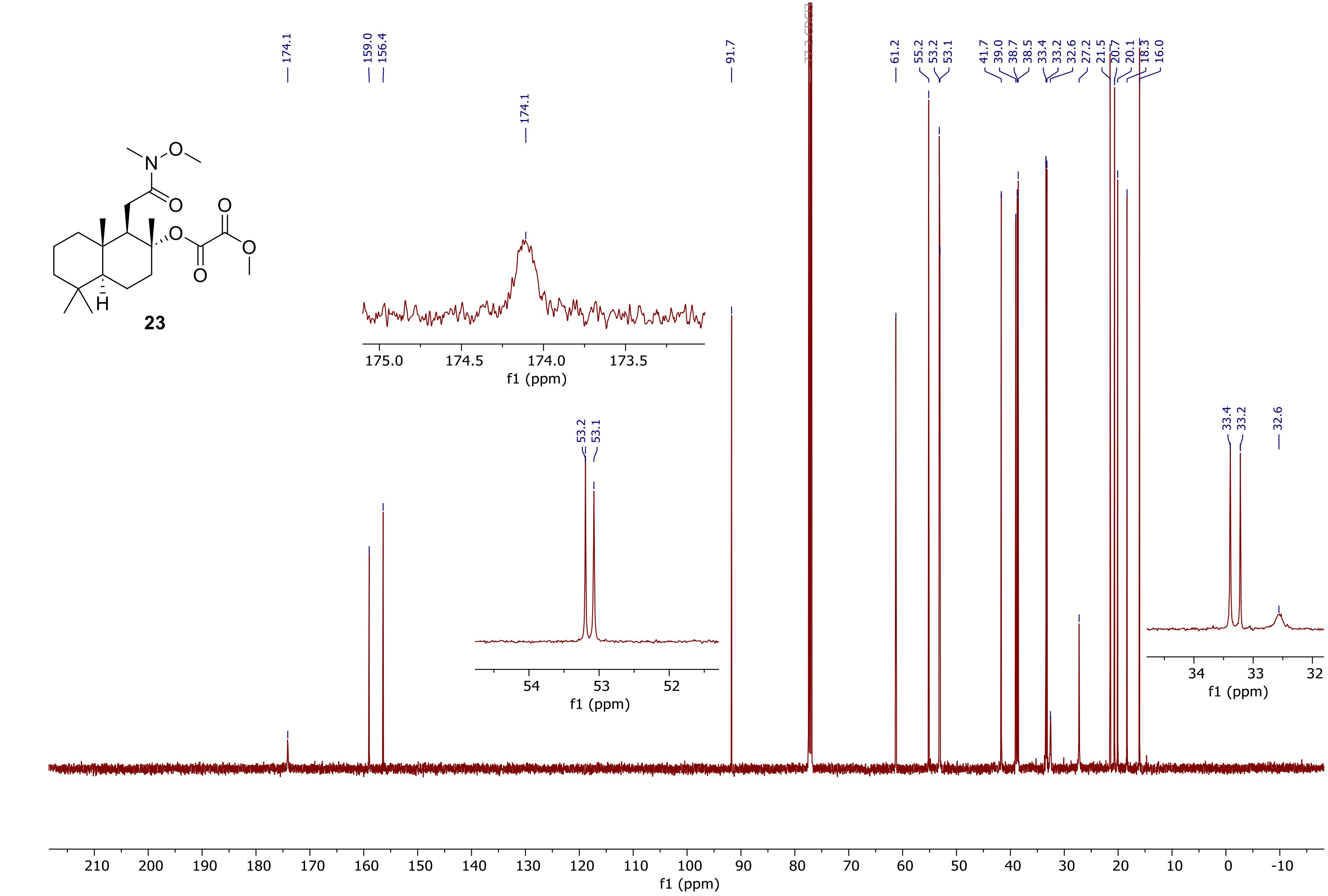

^{1}H NMR (500 MHz, $CDCl_3$)

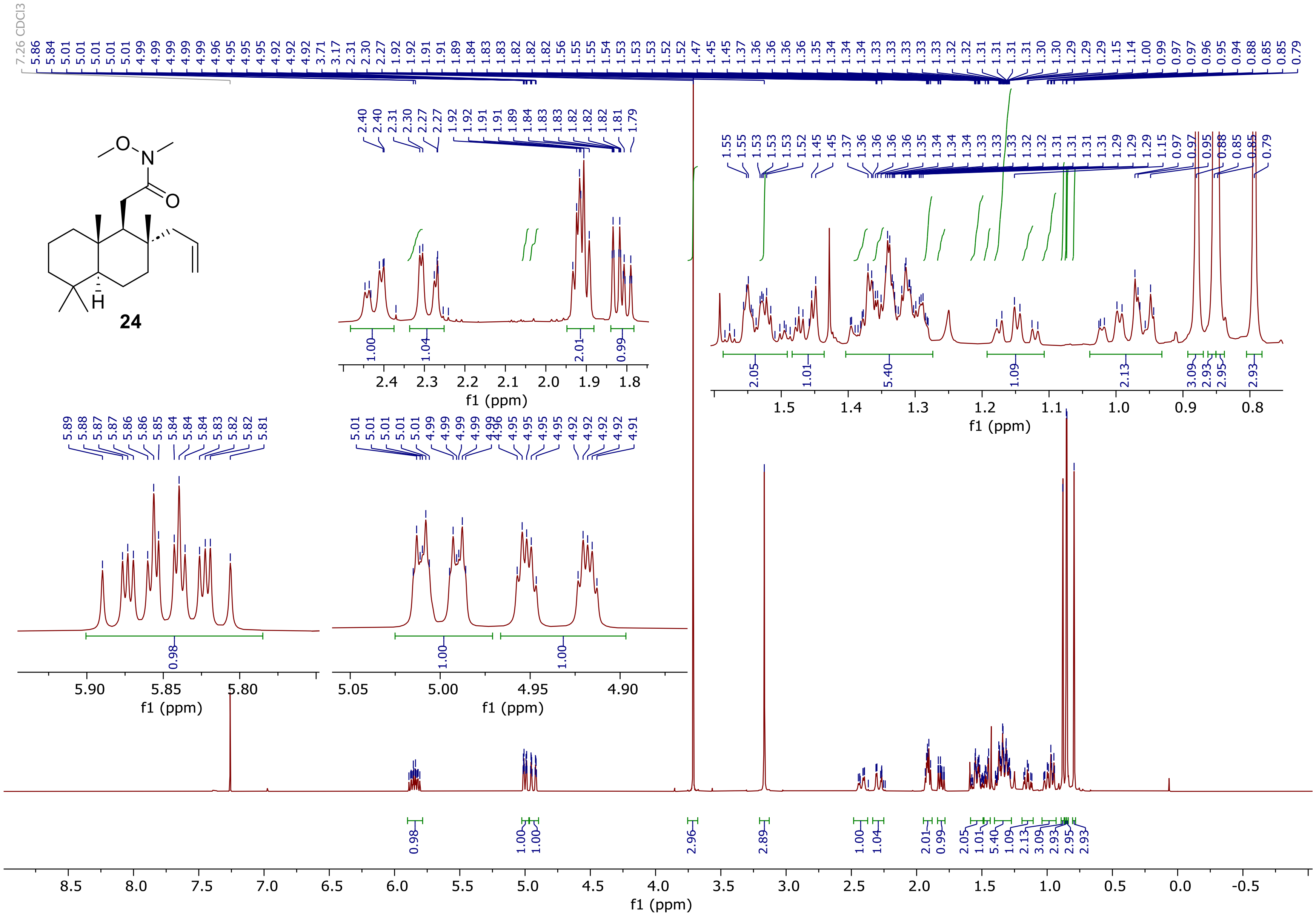

^{13}C NMR (126 MHz, $CDCl_3$)

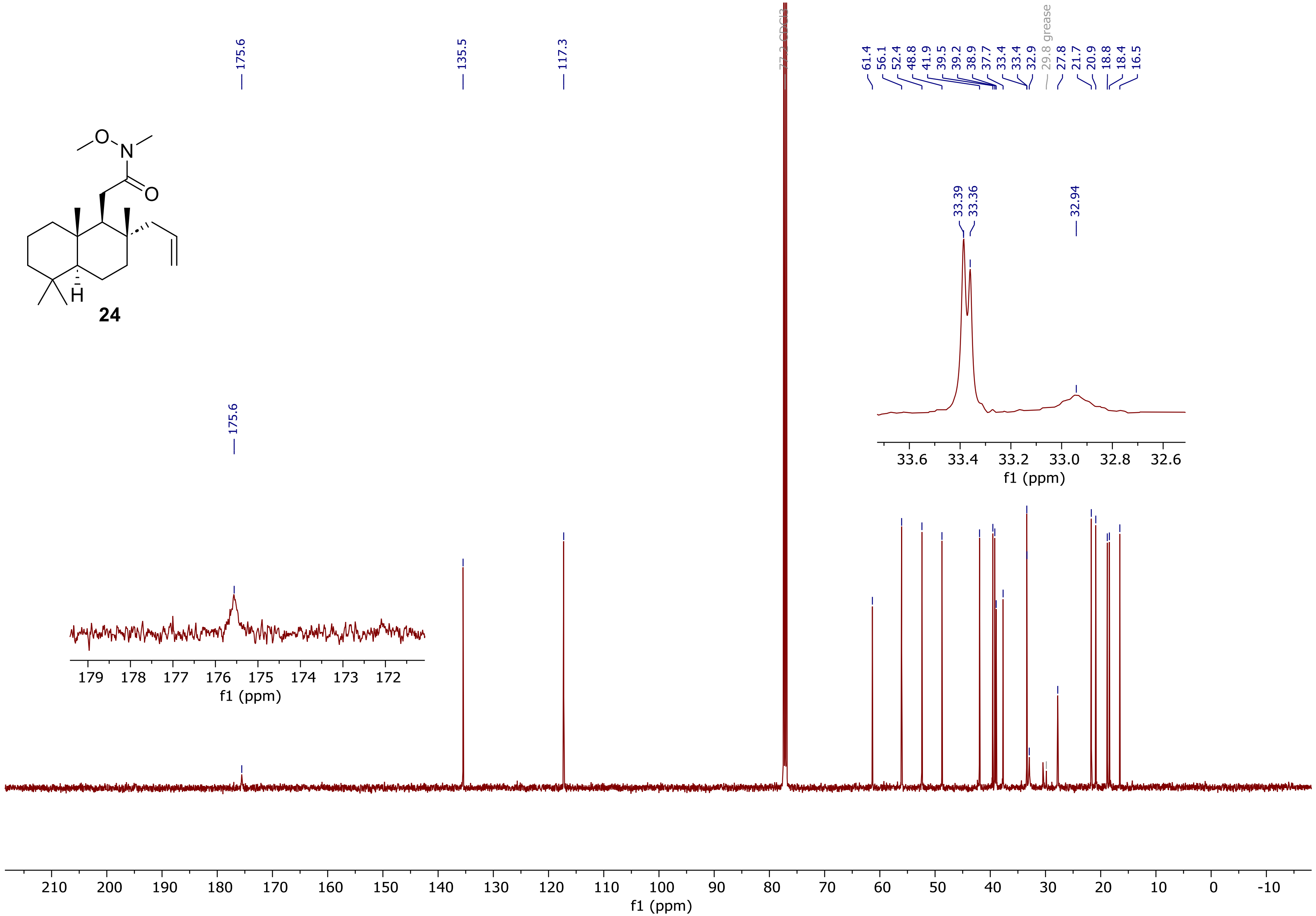

24

COSY (500 MHz, CDCl$_3$)

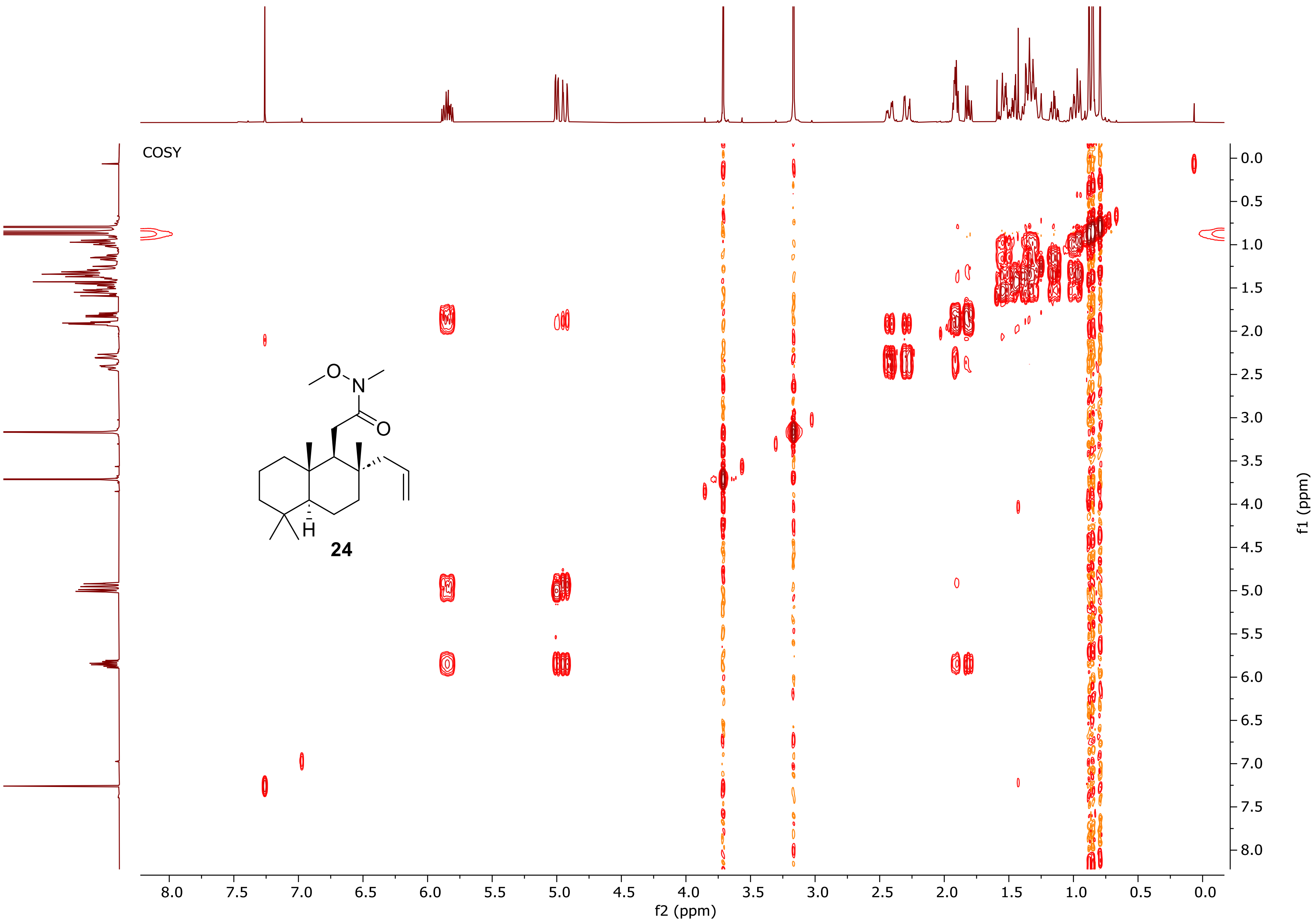

NOESY (500 MHz, $CDCl_3$)

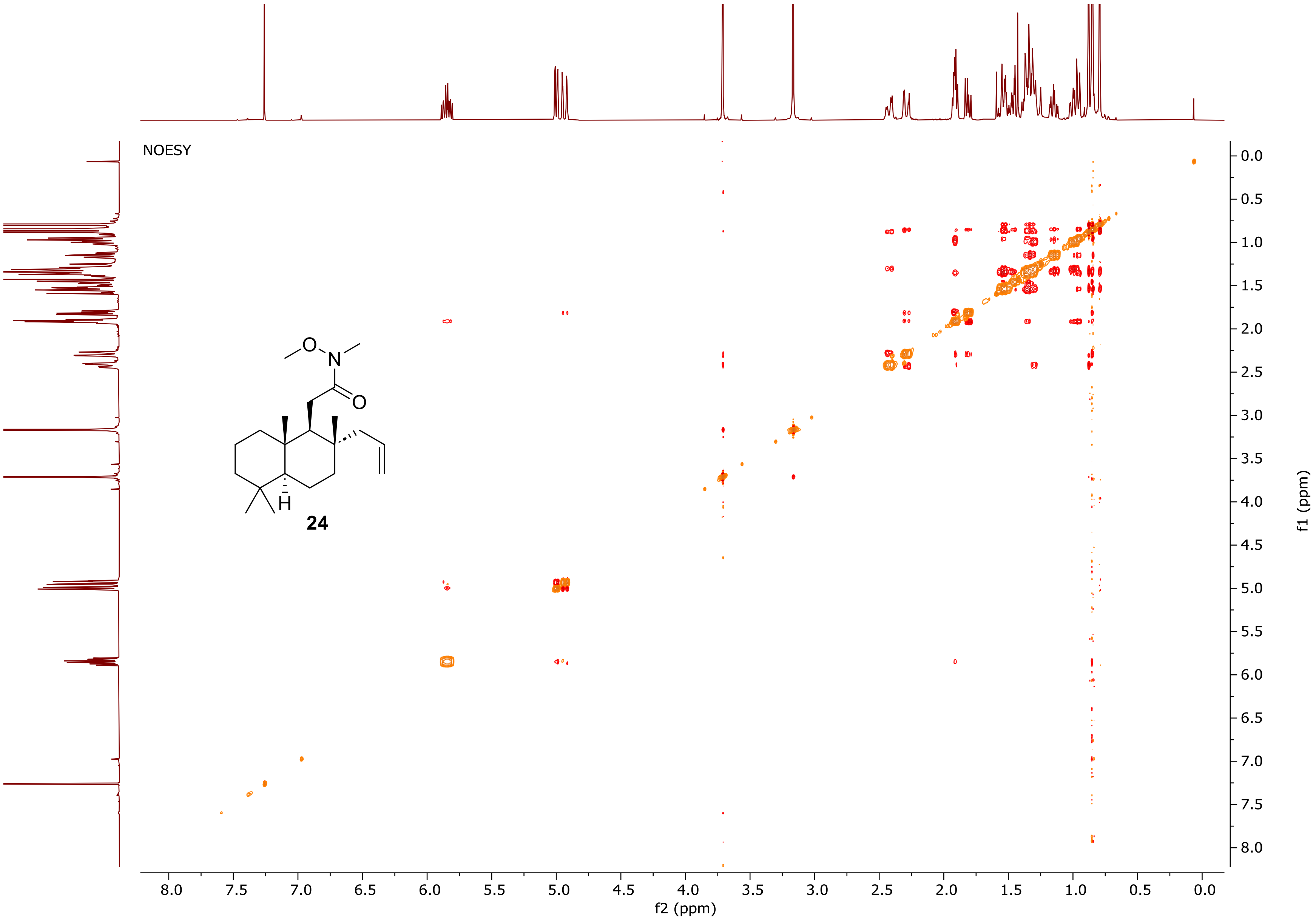

^{1}H NMR (500 MHz, $CDCl_3$)

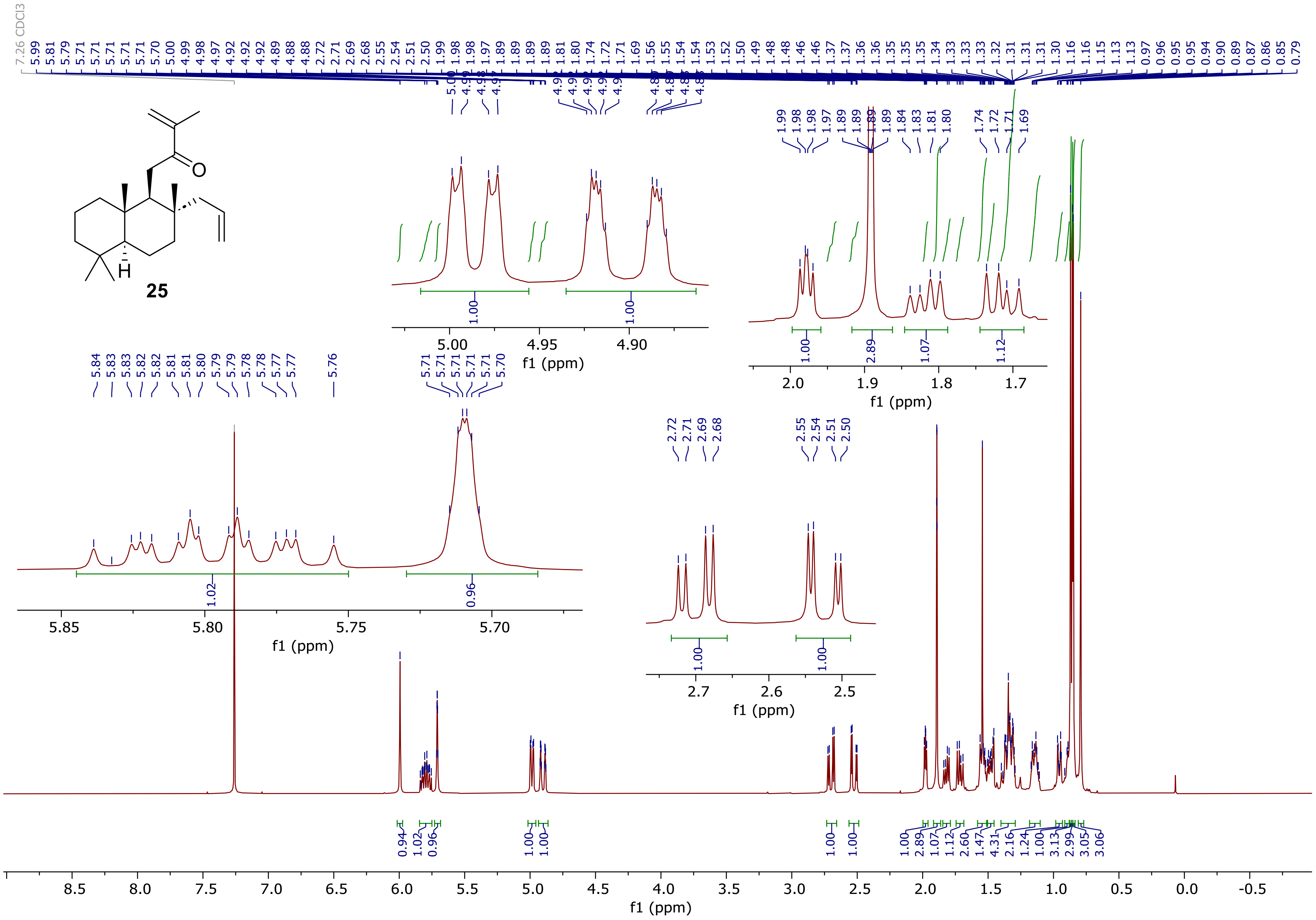

^{13}C NMR (126 MHz, $CDCl_3$)

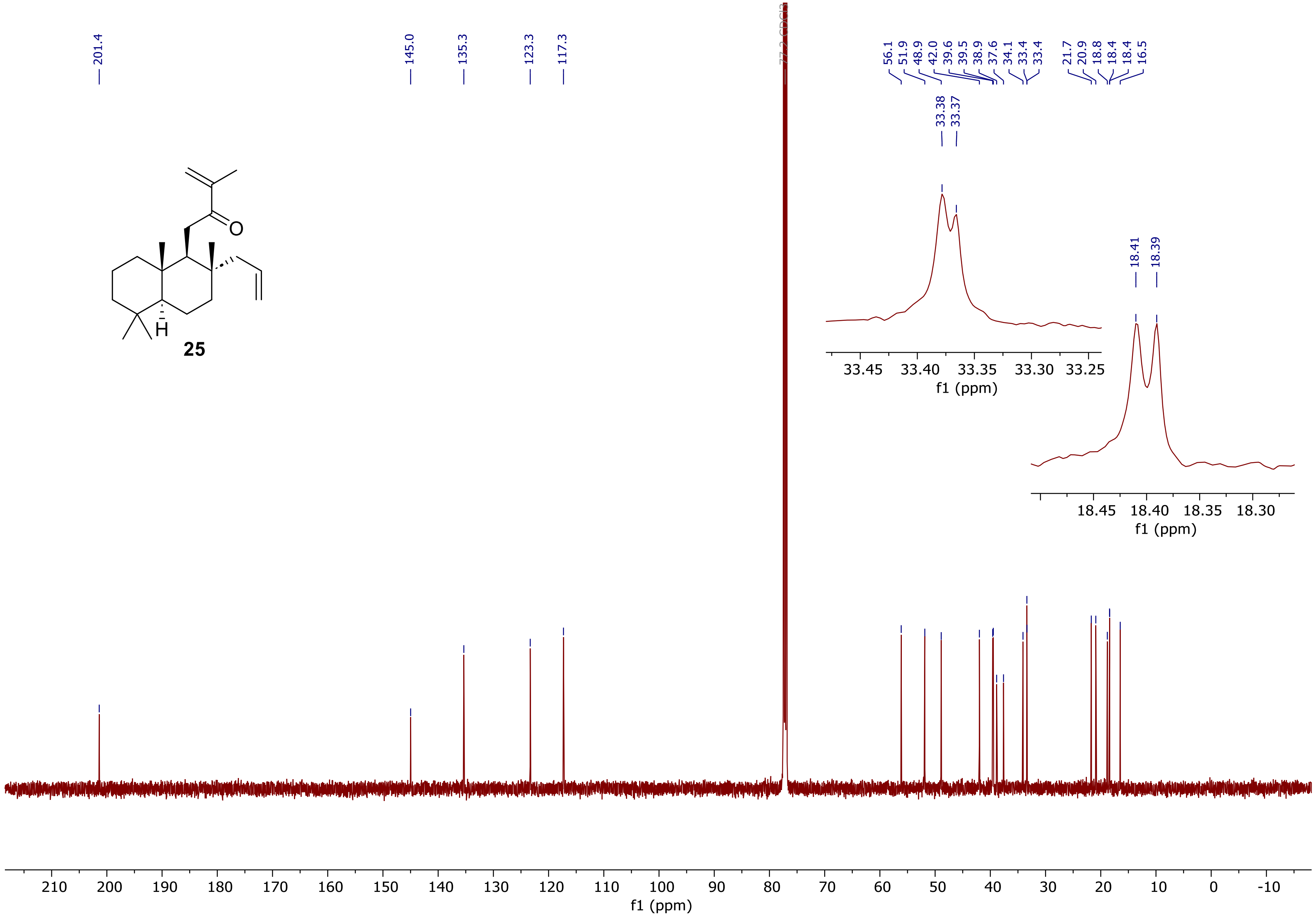

COSY (500 MHz, CDCl3)

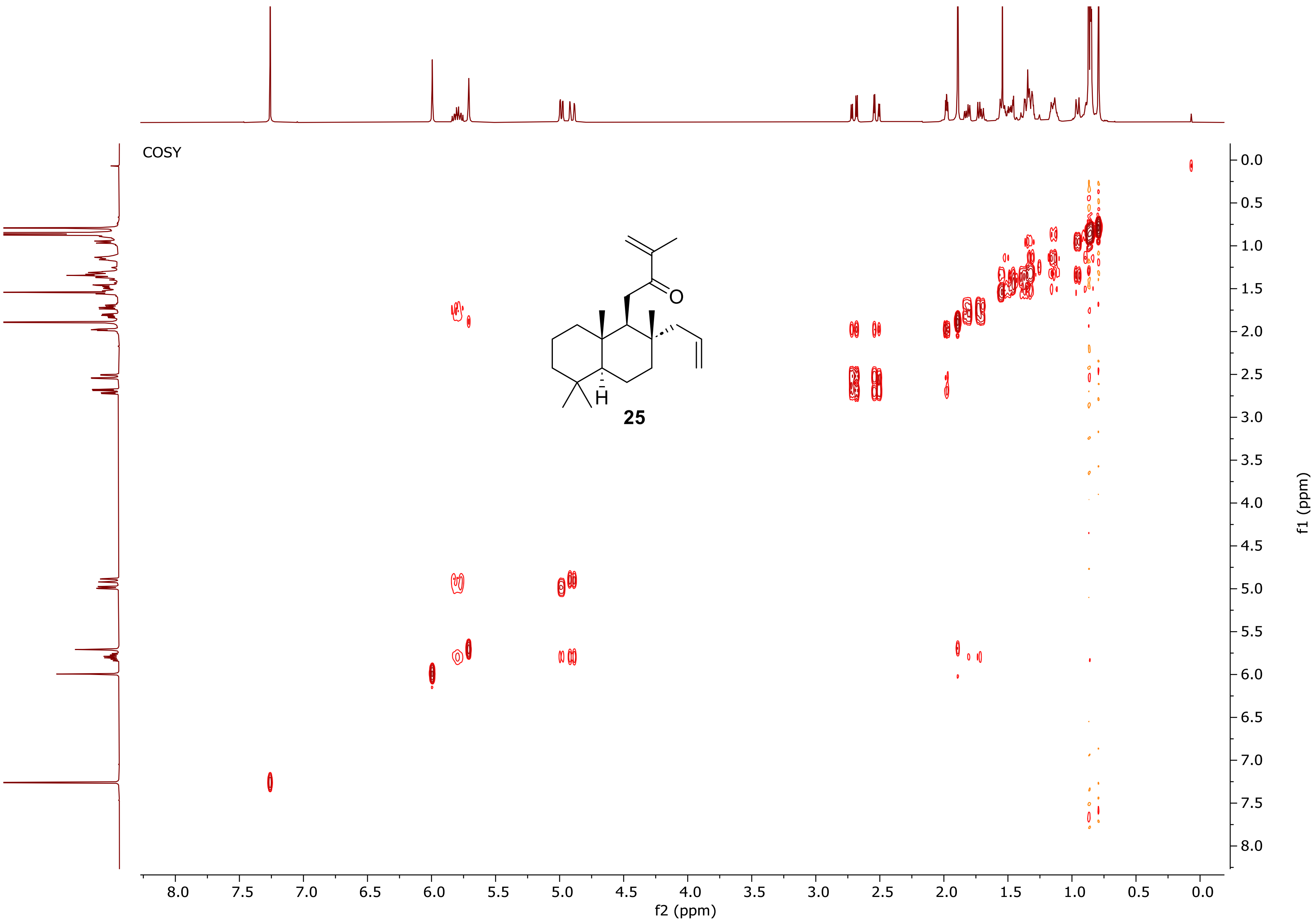

NOESY (500 MHz, $CDCl_3$)

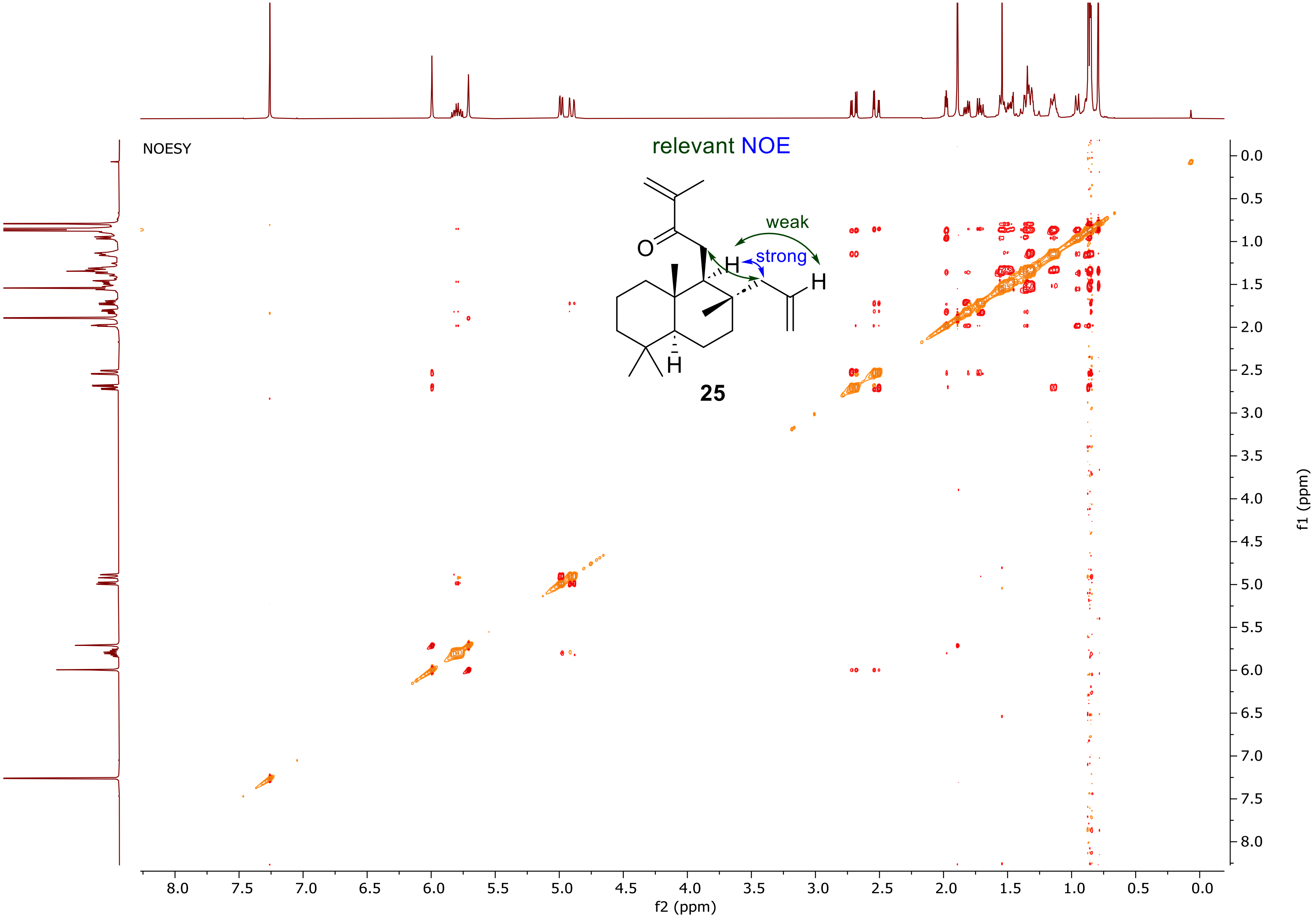

^{1}H NMR (500 MHz, $CDCl_3$)

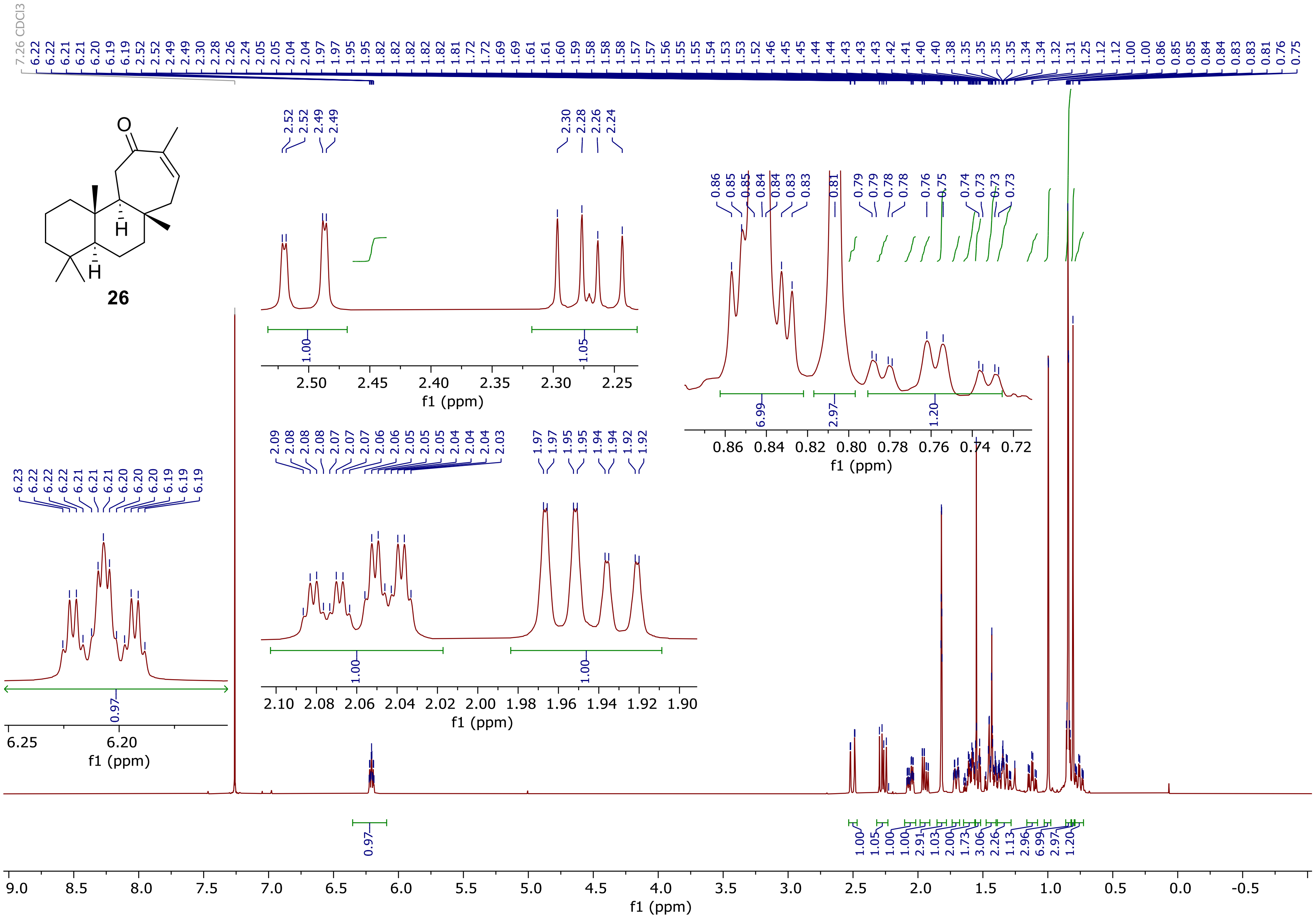

^{13}C NMR (126 MHz, $CDCl_3$)

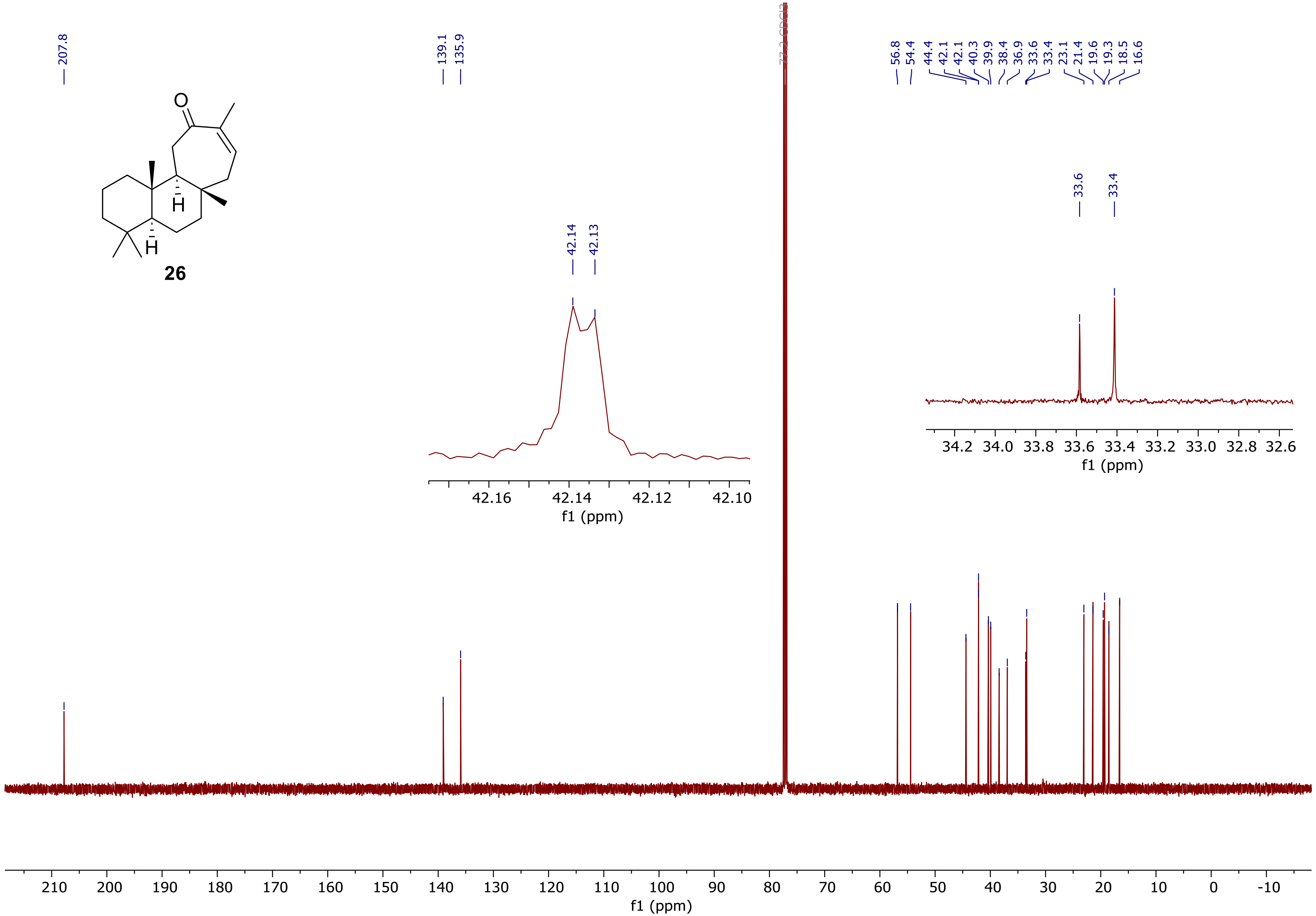

^{1}H NMR (600 MHz, C_6D_6)

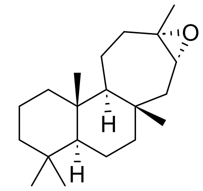

(+)-Barekoxide (21)
600 MHz

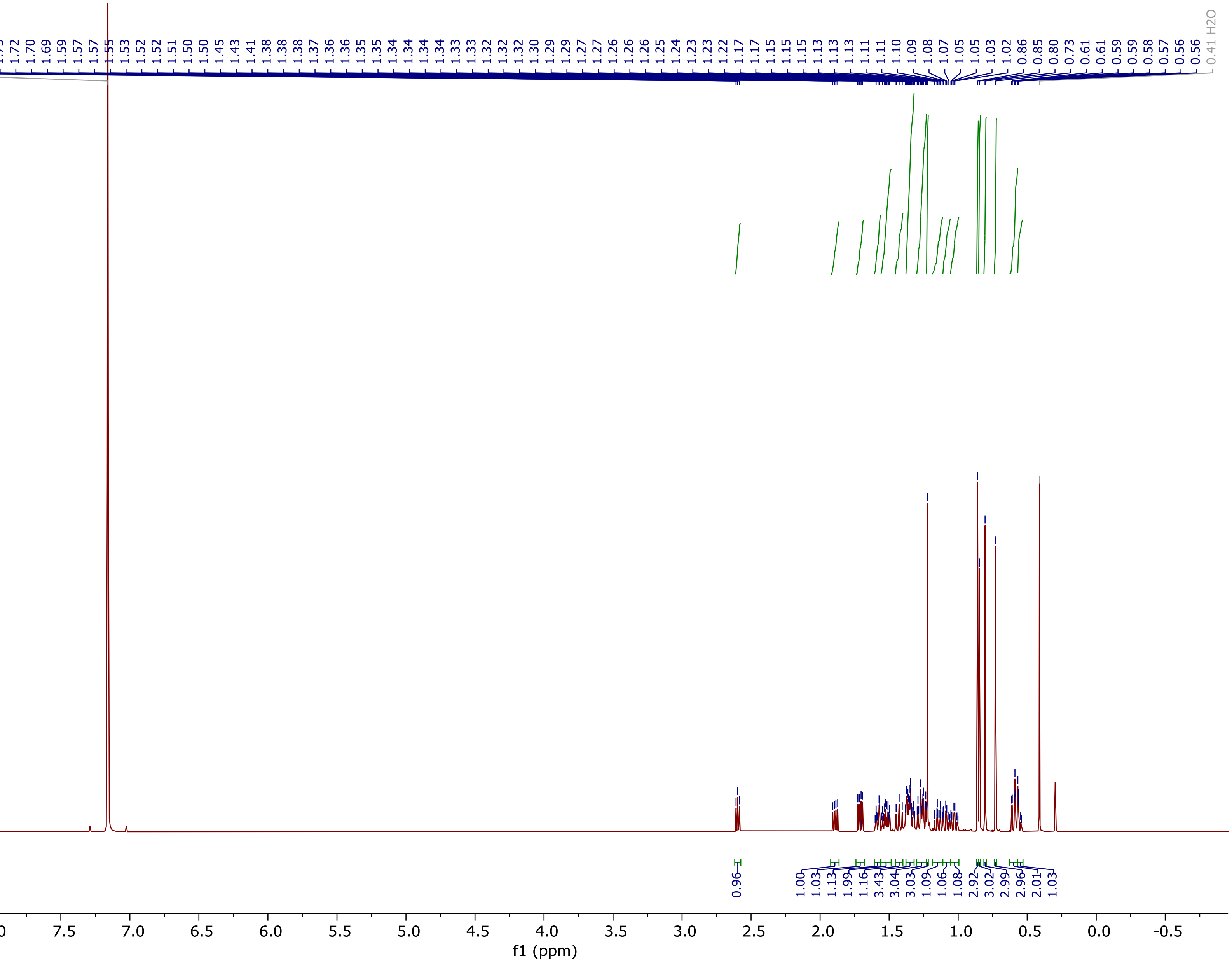

^{1}H NMR (600 MHz, C_6D_6)

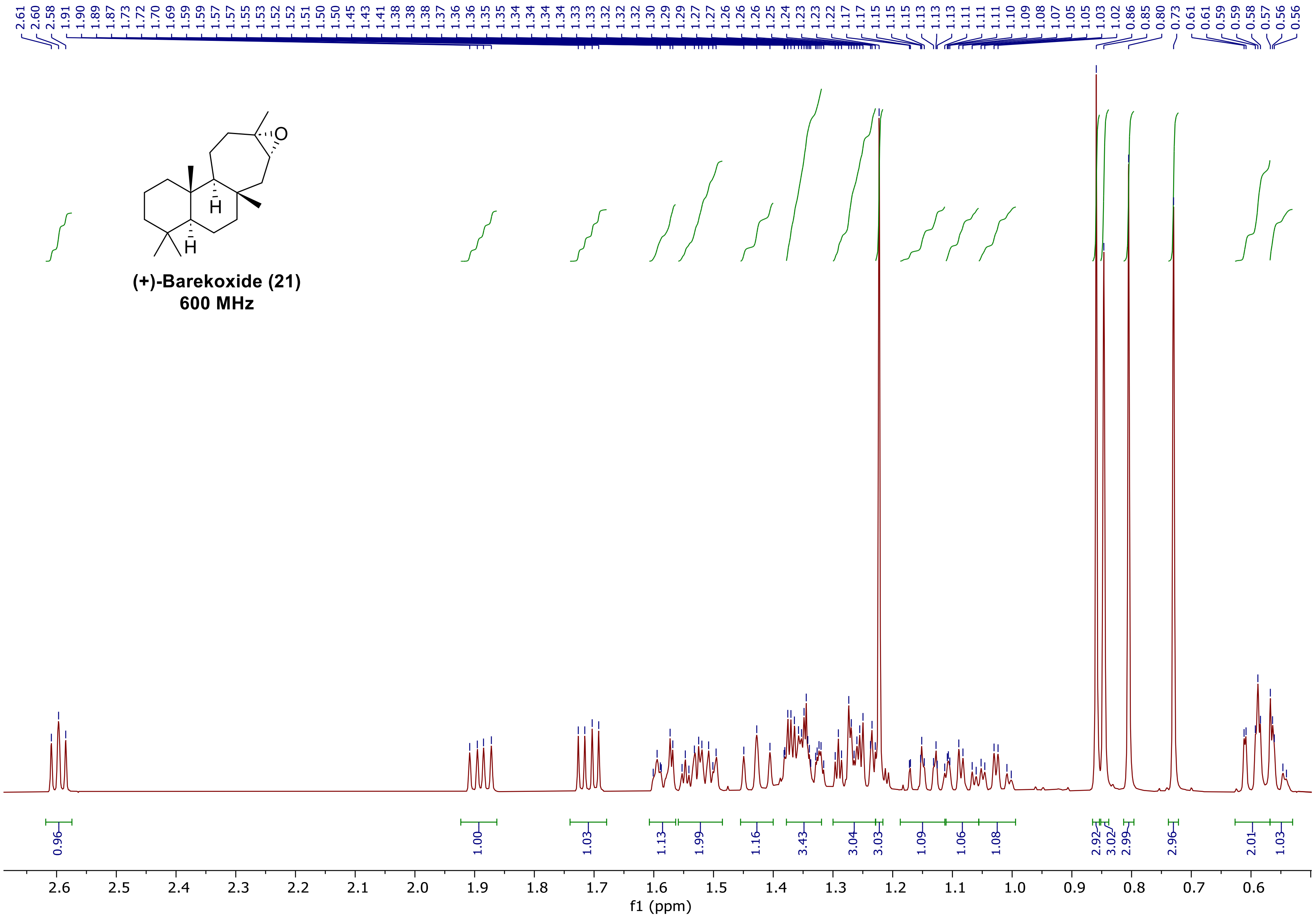

^{13}C NMR (151 MHz, C_6D_6)

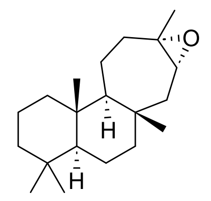

(+)-Barekoxide (21)

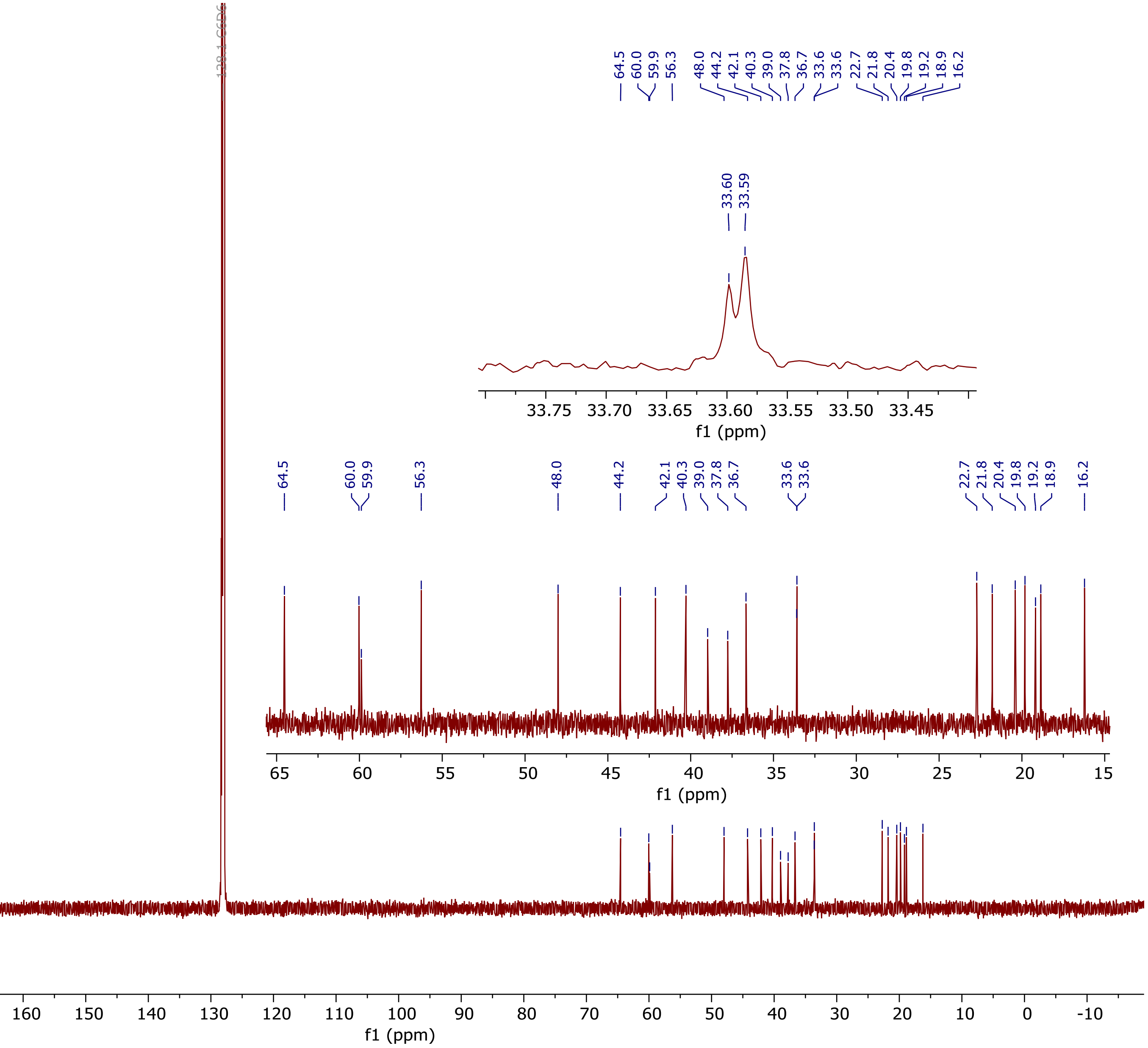

HSQC (600 MHz, C_6D_6)

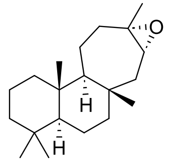

(+)-Barekoxide (21)

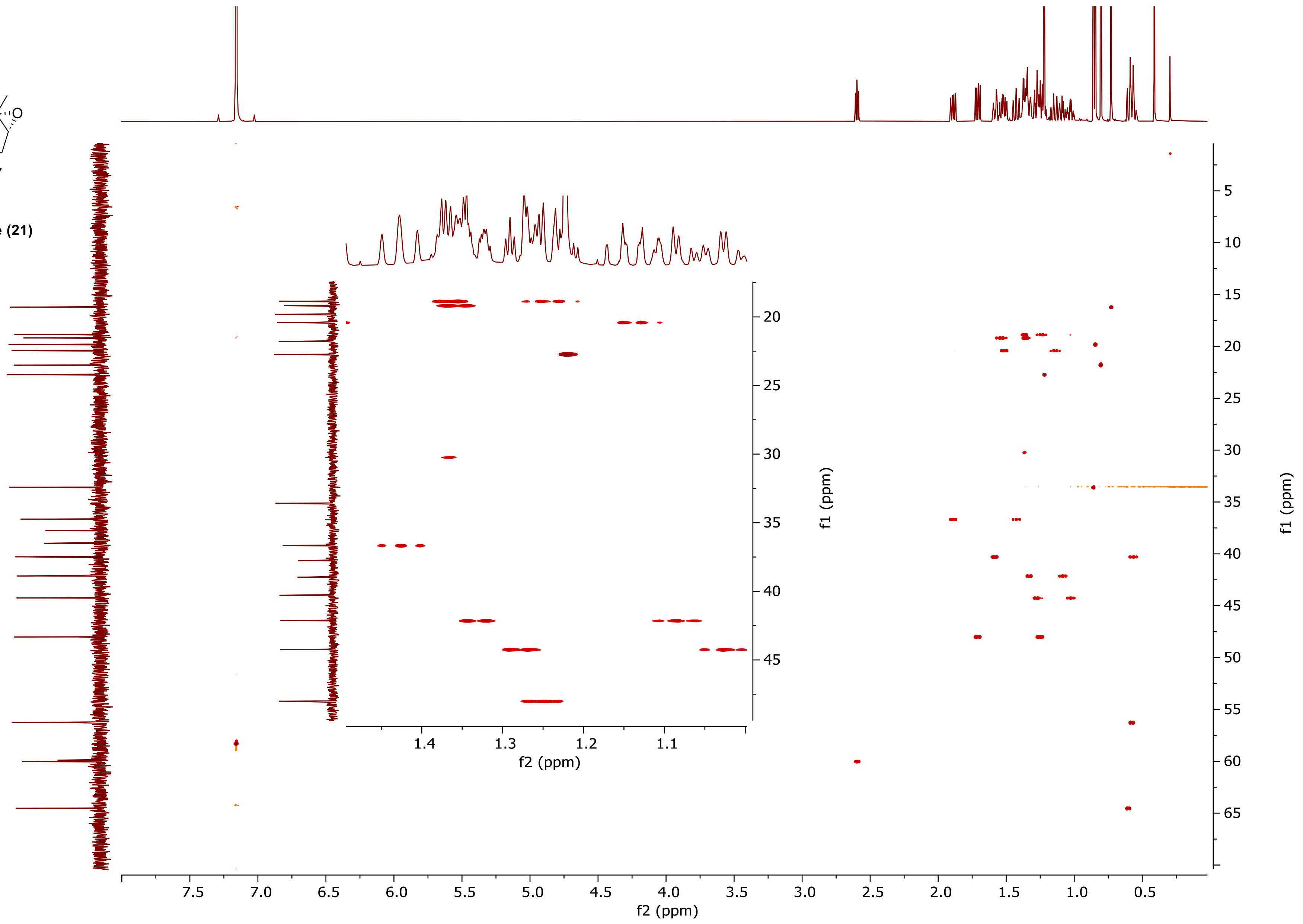

^{13}C NMR (151 MHz, C_6D_6) DEPT-135

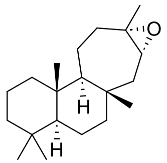

(+)-Barekoxide (21)

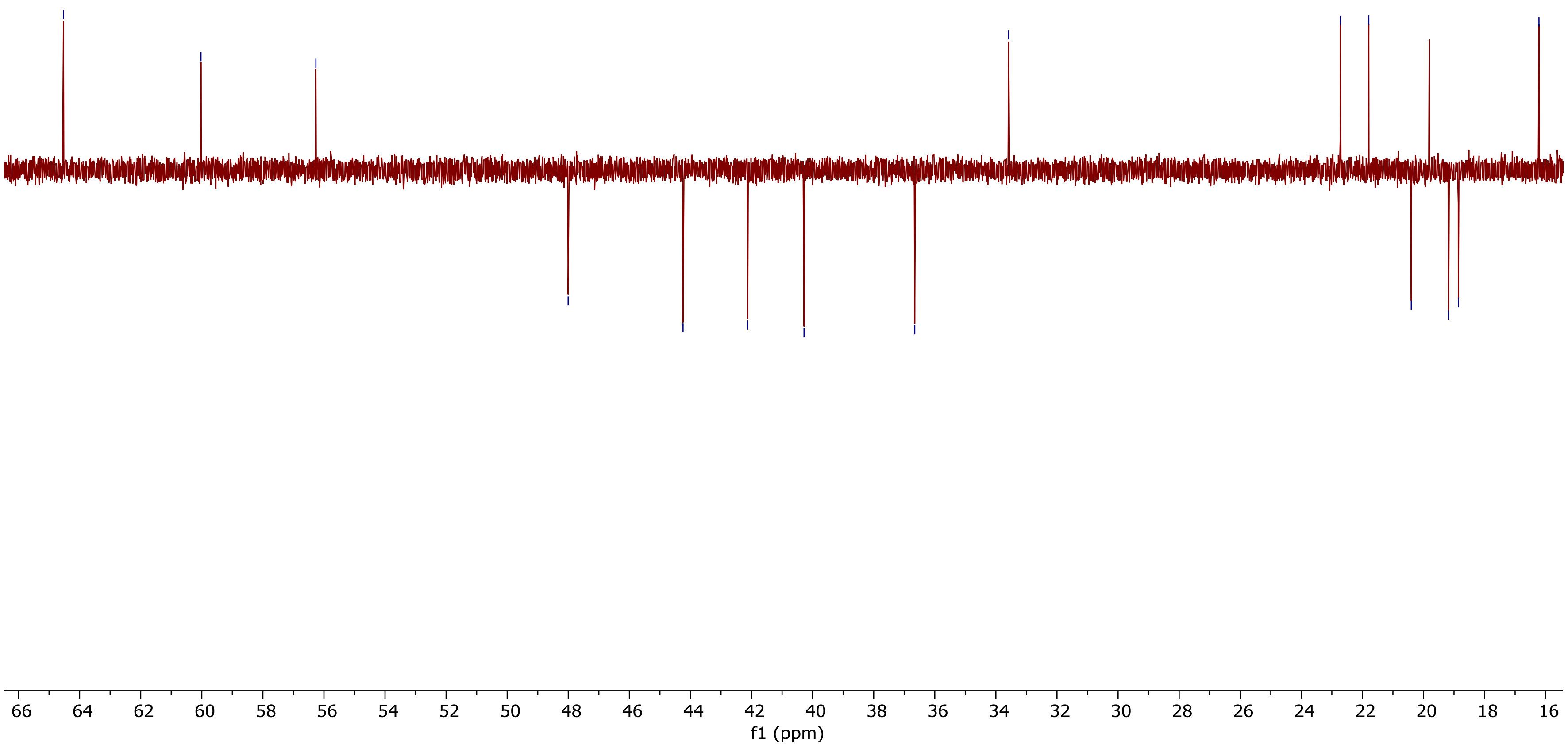

Supplement: Supplementary file 1 — ja4c02224_si_001.pdf [file ja4c02224_si_001.pdf]
